# Synthesis and Conformational Analysis of FR901464-Based RNA Splicing Modulators and Their Synergism in Drug-Resistant Cancers

**DOI:** 10.1021/acs.jmedchem.3c00733

**Published:** 2023-10-23

**Authors:** Jacob P. Beard, Robert K. Bressin, Paulo L. Markaj, John C. Schmitz, Kazunori Koide

**Affiliations:** † Department of Chemistry, University of Pittsburgh, 219 Parkman Avenue, Pittsburgh, Pennsylvania 15260, United States; ‡ Division of Hematology-Oncology, Department of Medicine, University of Pittsburgh School of Medicine, 5150 Centre Avenue, Pittsburgh, Pennsylvania 15232, United States; § Cancer Therapeutics Program, UPMC Hillman Cancer Center, 5117 Centre Avenue, Pittsburgh, Pennsylvania 15232, United States

## Abstract

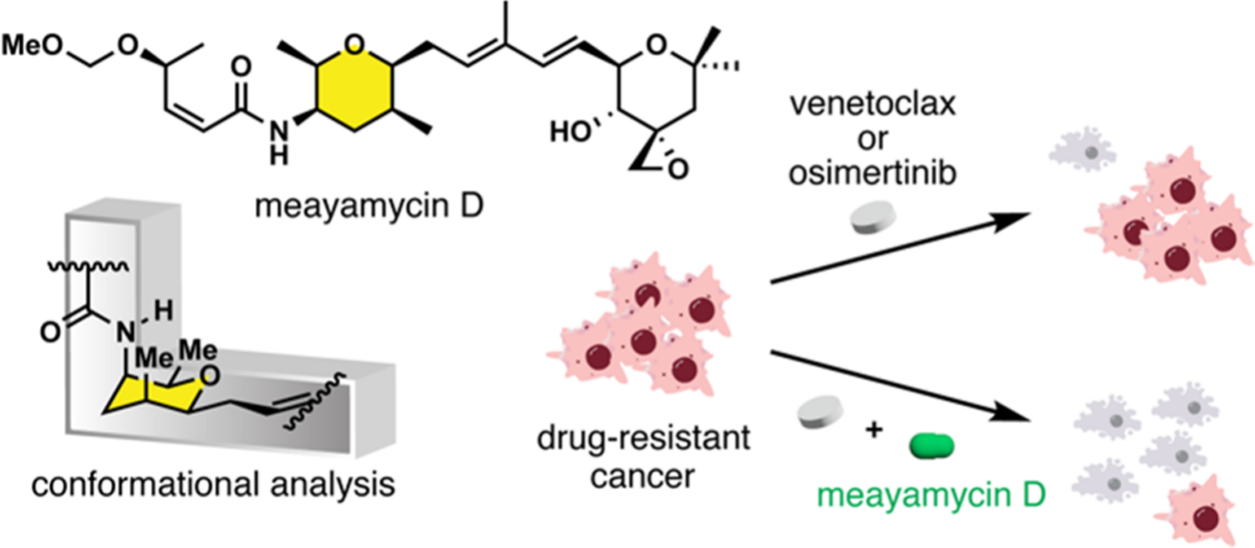

FR901464 is a cytotoxic natural product that binds splicing factor 3B subunit 1 (SF3B1) and PHD finger protein 5A (PHF5A), the components of the human spliceosome. The amide-containing tetrahydropyran ring binds SF3B1, and it remains unclear how the substituents on the ring contribute to the binding. Here, we synthesized meayamycin D, an analogue of FR901464, and three additional analogues to probe the conformation through methyl scanning. We discovered that the amide-containing tetrahydropyran ring assumes only one of the two possible chair conformations and that methylation of the nitrogen distorts the chair form, dramatically reducing cytotoxicity. Meayamycin D induced alternative splicing of *MCL-1*, showed strong synergism with venetoclax in drug-resistant lung cancer cells, and was cancer-specific over normal cells. Meayamycin D incorporates an alkyl ether and shows a long half-life in mouse plasma. The characteristics of meayamycin D may provide an approach to designing other bioactive L-shaped molecules.

## Introduction

**Figure 1:**
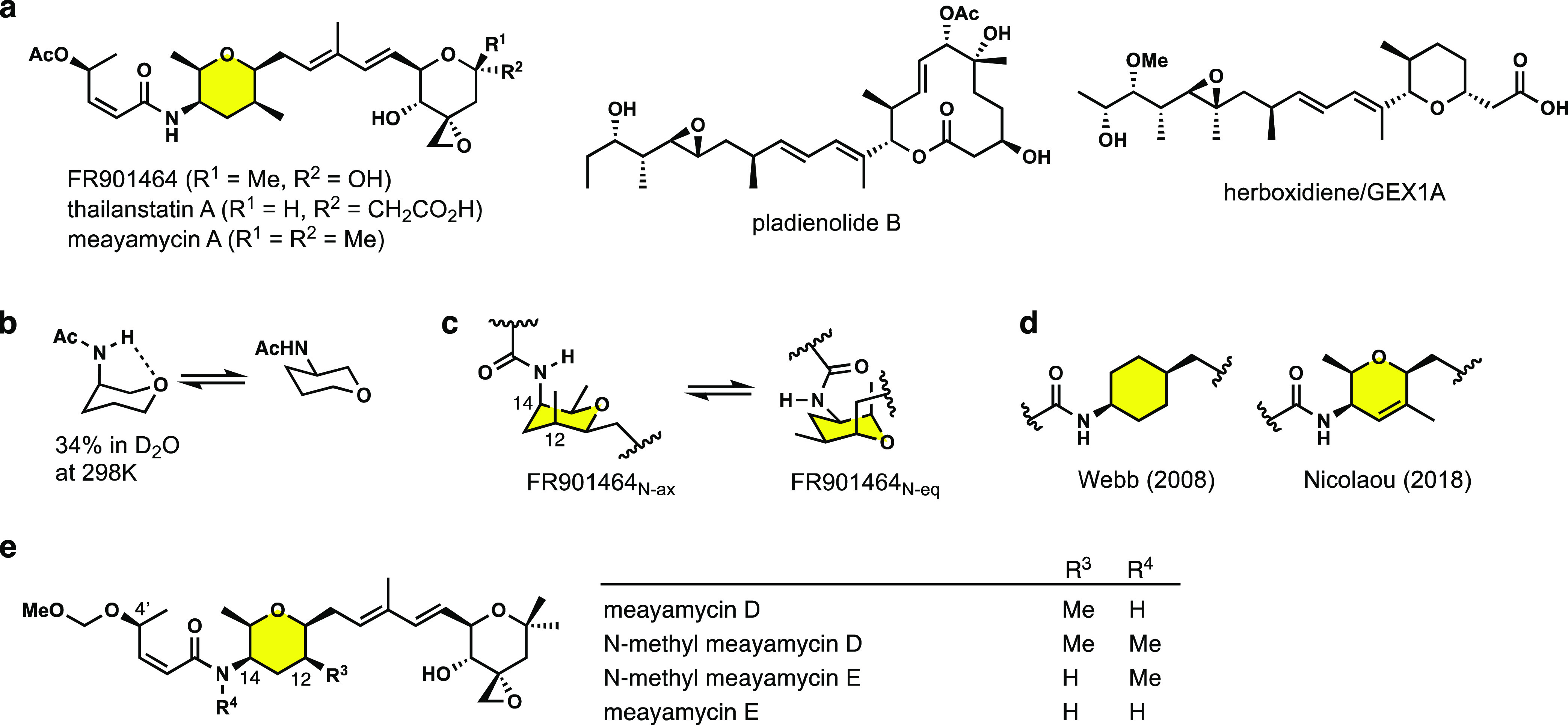
Structures of FR901464 and related compounds. (a) Structures of FR901464, thalianstatin A, meayamycin A, pladienolide B, and herboxidiene/GEX1A. (b) Vasella’s study. (c) Two chair conformations of the amide-containing fragment of FR901464. (d) Amide-containing six-membered ring analogues prepared by Nicolaou and Webb. (e) Structures of meayamycin D and analogues in this work.

Natural products FR901464,^1−3^ pladienolide,^4−6^ herboxidiene,^7,8^ and thailanstatin A^9,10^ (Figure 1a) modulate RNA splicing and exhibit potent anticancer activity. The tetrahydropyran rings of FR901464 bind splicing factor 3b subunit 1 (SF3B1) of the spliceosome.^11,12^ The great potency (GI_50_ 1–2 nM) has attracted synthetic chemists, culminating in total syntheses and several analogue development studies.^13−29^ Despite this, little is known about the atomic detail of the amide-containing tetrahydropyran ring in the SF3B1 active site. Prior to the discoveries of FR901464, a simplified variant of the amide fragment was studied by the Vasella group. They found that 34% of 3-acetamido-tetrahydropyran adopted the N-axial chair conformation in D_2_O at 298 K (Figure 1b).^30^ They proposed that an intramolecular hydrogen bond was formed between the N–H group with the ring oxygen, stabilizing the N-axial conformer.

We previously reported FR901464 analogues, meayamycins, and conducted a structure–activity relationship (SAR) study.^31^ However, it remained unclear which of the two chair conformers depicted in Figure 1c accounts for SF3B1 binding. The Webb group addressed the active conformation of the pyran ring of FR901464; their computational modeling placed the 3-acetamido-tetrahydropyran in the N-equatorial chair conformation (Figure 1d).^32^ In addition, the Nicolaou group found 3,6-dihydro-2*H*-pyran-containing analogues to be substantially less potent.^28^

In this paper, we describe the synthesis and conformational analysis of four new analogues to systematically probe the amide-containing six-membered ring conformation in meayamycin D (Figure 1e). Additionally, meayamycin D has activity comparable to meayamycin A in cancer cell lines and is significantly more metabolically stable. We show that meayamycin D has synergistic effects with venetoclax and osimertinib in lung cancer cell lines and reveal promising cancer specificity in colon cell lines with meayamycin D and meayamycin E. Our results provide insight into the active binding conformation of FR901464-related molecules and highlight *O-*alkyl ethers as compatible functional groups for future analogue developments.

## Results and Discussion

**Scheme 1:**
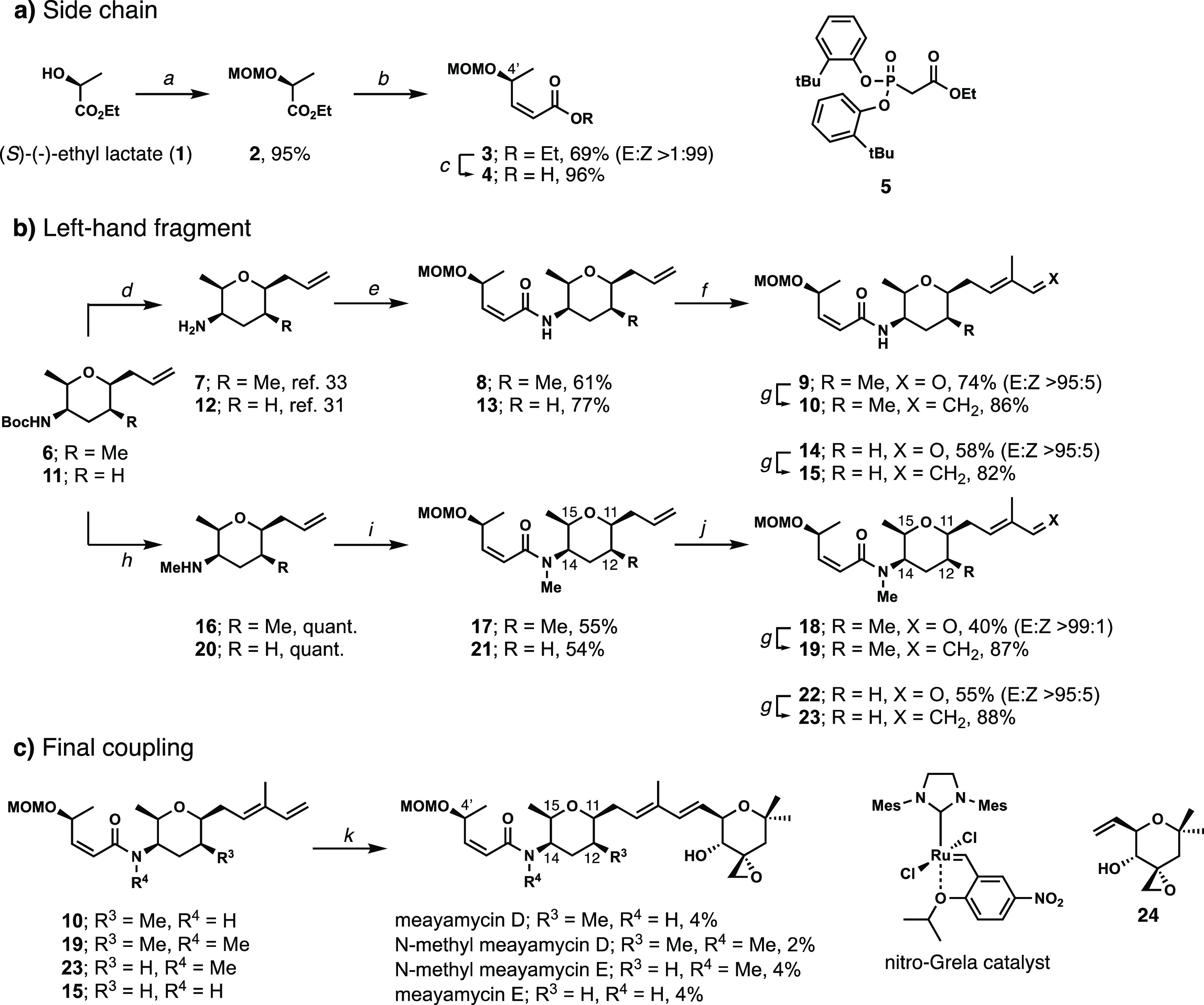
Synthesis of Meayamycin D and Three Other Analogues. Conditions: (a) CH_2_(OMe)_2_, AcCl, ZnBr_2_, Dichloromethane (DCM), 33 °C to Room Temperature (rt); (b) DIBALH, DCM, −78 °C; Then **5**, KO^*t*^Bu, Tetrahydrofuran (THF), −78 °C to rt; (c) NaOH, MeOH, 0 °C to rt; (d) Trifluoroacetic Acid (TFA)/DCM (1:9), 0 °C; (e) **4**, HATU, Diisopropylethylamine, DCM, 0 °C to rt; (f) Methacrolein, Nitro-Grela Catalyst, 40 °C; (g) Ph_3_PCH_3_Br, KO^*t*^Bu, THF, 0 °C; (h) LiAlH_4_, THF, Reflux; (i) **4**, HATU, Diisopropylethylamine, DCM, 0 °C to rt; (j) Methacrolein, Nitro-Grela Catalyst, 40–50 °C; (k) **24**, Nitro-Grela Catalyst, 1,2-Dichloroethane (DCE), 40–60 °C

Our first objective was to replace the esters or carbamates at the C4′-position with an ether group to prevent Z-to-E isomerization of the side chain during the amide coupling we previously reported.^33^ Specifically, methoxymethyl (MOM) ether was chosen as a suitable replacement, which proved to suppress undesired isomerization.^33^ We also anticipated that the MOM ether might be stable in plasma.^34^ Therefore, our first synthetic target was meayamycin D. Following the route developed for meayamycin B,^35^ (*S*)-(−)-ethyl lactate **1** was alkylated with in situ generated methoxymethyl chloride (the commercial supply of MOMCl was unstable during this study) to yield MOM ether **2** in 95% yield (Scheme 1a). Next, this compound was subjected to a sequential DIBALH reduction and Z-selective Horner–Wadsworth–Emmons reaction using Ando-type phosphonate **5**(36) and *t*-BuOK to afford Z-enoate **3** in 69% yield, with a *Z*/*E* ratio of >99:1. Enoate **3** was hydrolyzed to acid **4** in 96% yield, completing the synthesis of the side chain fragment.

Amine **6** was prepared using our previously reported method.^33^ Trifluoroacetic-acid-mediated Boc-deprotection followed by coupling with acid **4** using HATU formed amide **8** in 61% yield with retention of the olefin configuration (Scheme 1b, top). Next, this amide underwent olefin cross-metathesis with methacrolein, using nitro-Grela catalyst, to give aldehyde **9** in 74% yield. Finally, a Wittig olefination with Ph_3_P = CH_2_ afforded diene **10** in 86% yield.

We turned our attention toward synthesizing *N*-methyl meayamycin D to eliminate the intramolecular hydrogen bond (Scheme 1b, bottom). In the original report by Vasella, *N*-methylation of the 3-acetamido-tetrahydropyran yielded only the N-equatorial conformer.^30^ Therefore, we hypothesized that *N*-methyl meayamycin D might be constrained with the side chain in the equatorial conformation. The Boc-protected amine **6** was reduced with LiAlH_4_ to generate *N*-methyl amine **16** quantitatively. The coupling of amine **16** and acid **4** with HATU gave the tertiary amide **17** in 55% yield. Due to the amide bond’s slow rotation, the NMR spectrum of the tertiary amide presented signal peaks for two rotamers. Attempts to resolve these peaks by variable-temperature NMR were unsuccessful; therefore, one-dimensional nuclear Overhauser effect (NOE) experiments were used to confirm the presence of a single diastereomer (Figures S1 and S2).^37^ After confirming the structure, amide **17** was subjected to olefin cross-metathesis to give aldehyde **18** in 40% yield, which, upon Wittig olefination, delivered the *N*-methylated coupling partner **19** in 87% yield.

Analysis of the 15-H coupling constants in tetrahydropyrans **17**, **18**, and **19** led us to believe these compounds do not exist in a similar conformation to their non-*N*-methylated counterparts (**8**, **9**, and **10**). We asked whether removing the C12 methyl group enabled the tetrahydropyran ring to adopt the N-axial conformation more readily. Therefore, we synthesized a C12-desmethyl analogue (i.e., **23**) of diene **19**. We previously speculated that the hydrophobicity of the C12 methyl group might be important for binding.^31^ Based on this speculation, the *N*-methyl substituent might mimic the hydrophobicity of the C12 methyl group when oriented with the methyl group directed toward the C12 hydrogen and compensate for the direct loss of the C12 methyl group. The synthesis of *N*-methyl C12-desmethyl analogue **23** follows the same sequence as the *N*-methyl analogue **19** (Scheme 1b, bottom); Boc-protected amine^31^**11** was reduced with LiAlH_4_ to the corresponding *N*-methyl amine **20** quantitatively. Amine **20** was coupled with acid **4** using HATU to afford amide **21** in 54% yield. The additional NMR signals of comparable hydrogens between amides **21** and **17** were shifted almost identically, implying that amide **21** is similarly observed as rotamers. Amide **21** underwent olefin cross-metathesis and Wittig olefination giving aldehyde **22** and diene fragment **23** in 55 and 88% yields, respectively.

Finally, the C12-desmethyl analogue of meayamycin D (henceforth denoted as meayamycin E) was synthesized to investigate the effect on the diaxial interaction between the C12 and C14 positions. Our group previously synthesized and evaluated the morpholino carbamate analogue of meayamycin E.^31^ The synthesis follows the same general route as its *N*-methylated counterpart (Scheme 1b, top; see the Experimental Section for details). Employing nitro-Grela catalyst, olefin cross-metathesis of fragments **10**, **15**, **19**, and **23** with the right-hand fragment **24** was achieved to provide analogues meayamycin D, *N*-methyl meayamycin D, *N*-methyl meayamycin E, and meayamycin E in yields ranging between 2 and 4% after HPLC purification (see Scheme 1c for specific yields).

**Figure 2:**
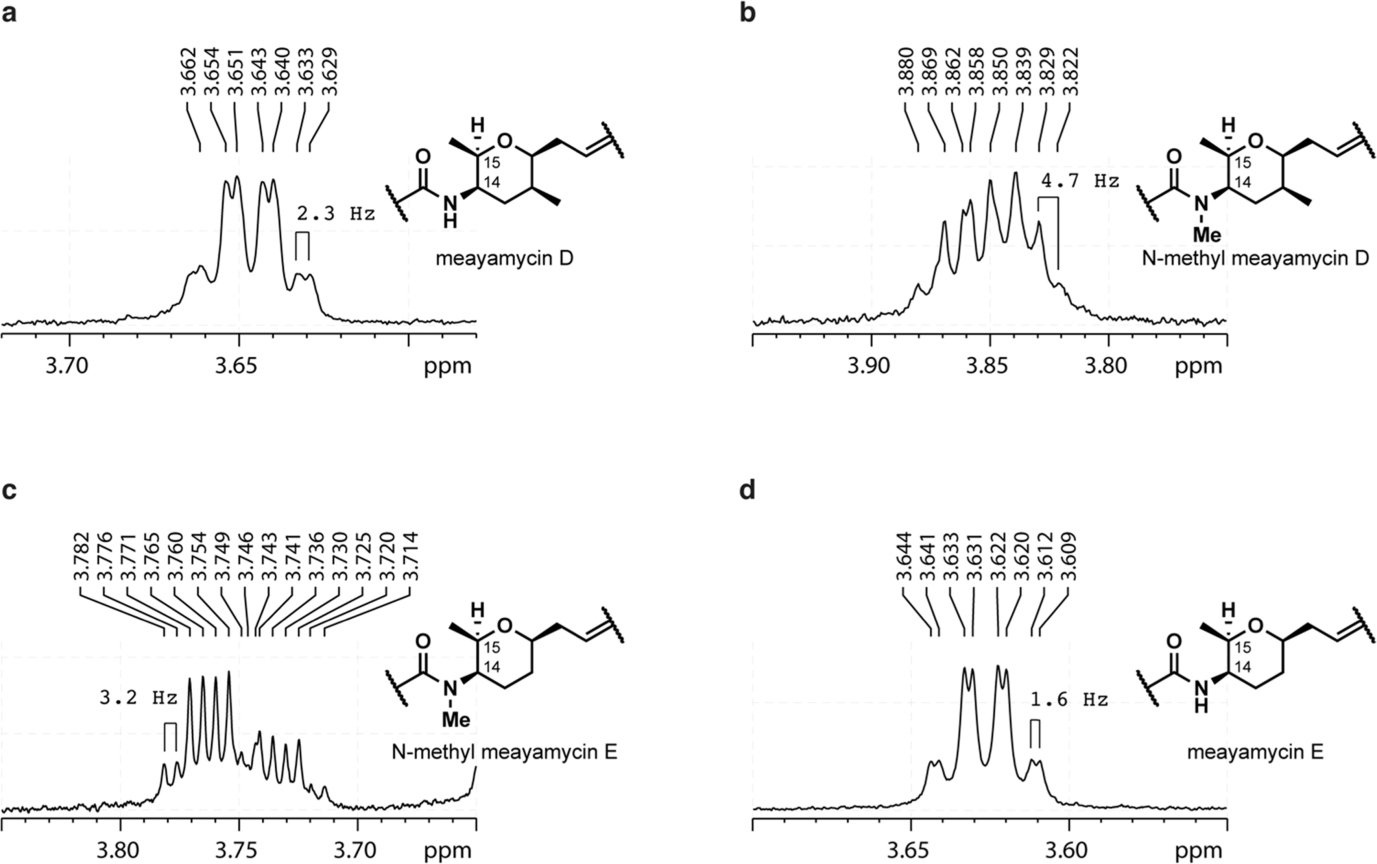
^1^H NMR coupling of (a) 15-H for compounds meayamycin D, (b) *N*-methyl meayamycin D, (c) *N*-methyl meayamycin E, and (d) meayamycin E. The spectra were taken at 600 MHz in CD_2_Cl_2_ or CDCl_3_.

With the representative compounds in hand, the ^3^*J*_15,14_ value was used to characterize the conformation of the pyran ring and compare its resemblance to the natural product ring conformation (Figure 2). The Karplus equation describes the relationship between the vicinal coupling constant (^3^*J*) observed in ^1^H NMR and the dihedral angle between those protons.^38,39^ In the ideal chair conformation of the tetrahydropyran, the dihedral angle between 14-H and 15-H is 60°. This correlates to a ^3^*J* value of ca. 2 Hz, according to the Karplus equation. In FR901464 and meayamycin A, the ^3^*J*_15,14_ values range from 2.0 to 2.1 Hz in CD_2_Cl_2_, indicating a chairlike conformation with a dihedral angle of ca. 60°. For meayamycin D, the ^3^*J*_15,14_ of 2.3 Hz in the same solvent indicates a chair conformation similar to FR901464 (Figures 2a and S3). Analysis of *N*-methyl meayamycin D revealed a stark difference; the major rotamer showed ^3^*J*_15,14_ of 4.7 Hz (Figure 2b). The Nicolaou group had previously observed ^3^*J*_15,14_ of 6.1 Hz for a boat conformer of an equivalent N-phthaloyl intermediate.^25^ Therefore, we propose that the amide-containing tetrahydropyran ring of *N*-methyl meayamycin D adopts a twisted-boat conformation with a dihedral angle close to ca. 40°. When the C12 methyl group is absent, the ^3^*J*_15,14_ was 3.2 Hz (Figure 2c), implying that the conformation of *N*-methyl meayamycin E is closer to those of FR901464 and meayamycin A. Meayamycin E resembled the parent compound, with the ^3^*J*_15,14_ of 1.6 Hz (Figure 2d). These results reveal that *N*-methylation of the amide induces a significant conformational change.

Next, we asked whether the twisted conformation in *N*-methyl meayamycin D stemmed from the disruption of the proposed hydrogen bond. In Vasella’s conformational studies, the N-equatorial conformer predominates in protic solvent due to the competing intermolecular hydrogen bond formed between solvent molecules and the substrate (see Figure 1b). However, unlike Vasella’s model system, the N-equatorial conformation in meayamycin D presents a 1,3-diaxial interaction between the C11 and C15 substituents. Therefore, compounds **8**, **13**, **17**, and **21** were analyzed in CD_3_OD to determine whether any analogues adopt a different conformation in a protic environment. In CD_3_OD, compounds **8** and **13** had similar ^3^*J*_15,14_ values of 2.3 and 1.8 Hz, respectively, indicating these compounds do not alter their chairlike conformation in the protic solvent (Figures S4, S5, S10, and S11). Similarly, *N*-methylated compounds **17** and **21** have almost identical ^3^*J*_15,14_ of 4.7 and 3.3 Hz in protic solvent (Figures S6–S9). These results indicate that the solvent does not significantly influence the pyran conformation.

**Figure 3:**
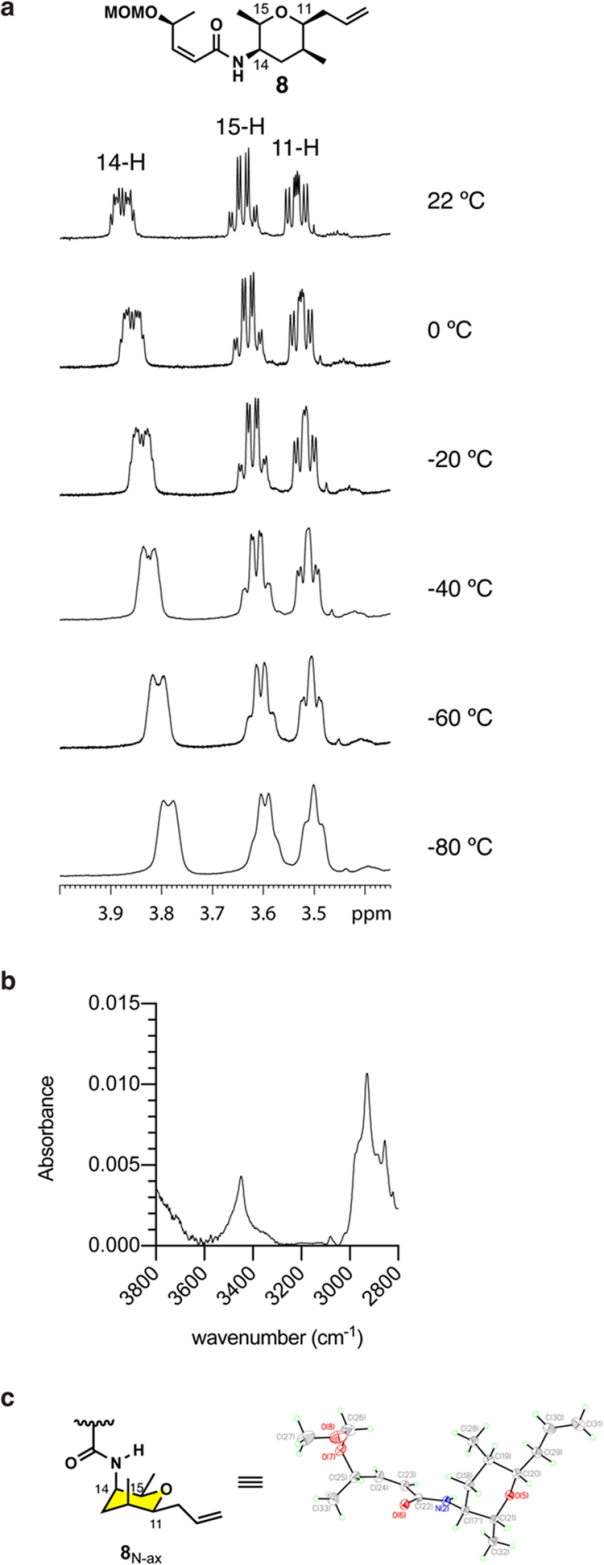
Spectroscopic analysis of amide **8**. (a) ^1^H NMR expansion from 3.4 to 4.0 ppm of amide **8** in CD_2_Cl_2_ (20 mM) from 22 to −80 °C. (b) IR spectrum of amide **8** from 2800 to 3800 cm^–1^ (5 mM in CCl_4_) at room temperature. (c) X-ray structure of amide **8**.

Next, we aimed to quantify the percentage of the secondary amide **8** in the N-axial and N-equatorial conformations. At room temperature, the observed coupling constants and chemical shifts are averages of all conformers. However, the ^1^H NMR signal for the hydrogen vicinal to the acetamide group (14-H in meayamycin D) is distinguishable at cryogenic temperatures.^30^ Therefore, we performed the ^1^H NMR study of amide **8** in CD_2_Cl_2_ from 21 to −81 °C (Figures 3a and S12). Surprisingly, the 14-H signal was not split at any of the temperatures scanned. Additionally, we performed IR experiments to characterize the ring conformation further. The Vasella group studied the conformation of 3-acetamido-pyrans by IR spectroscopy to show a splitting of the N–H band based on the distribution between the unbound state and intramolecular-bound state.^30^ We acquired the IR spectrum of amide **8** in CCl_4_ at 5 mM; CCl_4_ was chosen to mimic the hydrophobicity of the binding pocket^12^ and exclude the effects of solvation or aggregation. We observed only one peak for the N–H stretching at 3453 cm^–1^ (Figure 3b), indicating that amide **8** exists as a single conformer in CCl_4_. ^1^H NOE analysis of the 11-, 13-, and 15-Hs signifies that the sole conformation observed is the N-axial conformation (Figures S13–S15). To gain further insight, we performed DFT calculations of amide **8**. The lowest energy conformation located for amide **8** in dichloromethane placed the tetrahydropyran ring in the N-axial conformation (Figure S16). Additionally, the 14 H–15 H dihedral angle was calculated to be 57°, matching closely with the angle determined by ^1^H NMR coupling. Finally, an X-ray crystal structure of amide **8** was obtained and confirmed that the solid-state conformation of amide **8** is solely the N-axial conformation (Figure 3c). Taken together, this data indicates that the amide-containing ring of FR901464 is essentially “locked” in one chair conformation and does not require further rigidity to improve the binding affinity.

**Figure 4:**
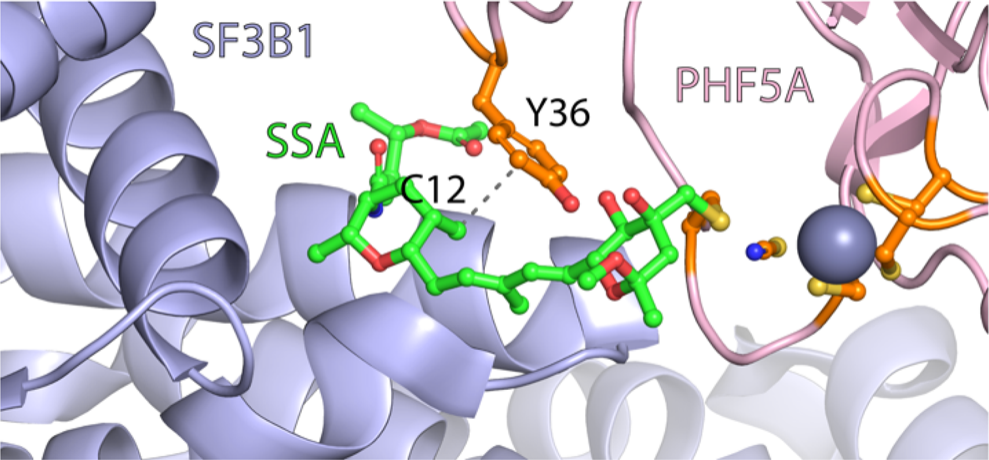
Proposed CH–π interaction between C12 methyl of spliceostatin A (SSA) and Y36 of PHF5A (PDB: 7B9C).

Meayamycin D, meayamycin E, and their *N*-methyl analogues were evaluated for their cytotoxicity against various cancer cell lines (Table 1 and Figure S17). Meayamycin D exhibited GI_50_ comparable to meayamycin A, ranging from 2.0 to 3.9 nM. Meayamycin E exhibited a slightly higher GI_50_, which may be caused by the loss of the CH-π interaction^40^ between the C12 methyl group and Y36 of PHF5A (Figure 4).^12^*N*-Methyl meayamycin D and *N*-methyl meayamycin E showed GI_50_ over 1000-fold greater than their non-*N*-methylated counterparts, highlighting a necessity for the C14–N-axial conformation. However, we cannot disregard the possibility that the substitution of the methyl group might remove a necessary hydrogen bonding interaction which could be more critical than the altered conformation of the *N*-methylated analogues. It is also possible that these *N*-methylated analogues are still weakly bound to SF3B1 and inhibit growth through alternative means as observed with other inactive SF3B1-inhibitors.^41^ Finally, the potent compounds were evaluated in normal colon epithelial cells (CCD-841CoN) and liver hepatocytes (FL83B). The GI_50_ values for meayamycin A and meayamycin D were slightly higher (ca. 2- to 4-fold) in normal cells. Meanwhile, there was a 3 to 6-fold difference in the GI_50_ for normal cells treated with meayamycin E. These results suggest meayamycins may be further tuned to selectivity target cancer cells and serve as an avenue for future drug development.

**Table 1:** Half-Maximal Growth Inhibition Concentrations (GI_50_) for Meayamycin A, Meayamycin D, and Analogues^a^

GI_50_ values of meayamycin and synthetic analogues (nM)					
cell line	meayamycin A	meayamycin D	meayamycin E	N-Me meayamycin D	N-Me meayamycin E
HCT116	0.7 ± 0.1	2.0 ± 0.3	10.5 ± 1.2	4100 ± 620	16000 ± 2500
SW48	0.9 ± 0.1	2.5 ± 0.4	10.7 ± 1.6	4600 ± 850	16000 ± 3000
A549	2.7 ± 0.9	3.9 ± 1.3	8.6 ± 2.6	7500 ± 1800	34000 ± 5300
DMS53	0.5 ± 0.1	2.7 ± 0.6	14.9 ± 2.2	6000 ± 1000	23000 ± 4700
DMS114	0.4 ± 0.1	2.3 ± 0.6	10.2 ± 1.6	4900 ± 960	8700 ± 1100
CCD-841CoN^b^	2.0 ± 0.4^d^	9.6 ± 3.2^c^	63.2 ± 11.5^d^		
FL83B^b^	0.9 ± 0.2	5.7 ± 0.4^e^	35.7 ± 5.0^c^		

a Values reported as mean ± standard deviation (SD) of *n* ≥ 3 individual experiments (see Figure S17 for growth inhibition curves).

b Normal cells.

c *p* < 0.05.

d *p* < 0.01.

e *p* < 0.001, significance between HCT116 or SW48 and normal cells.

**Figure 5:**
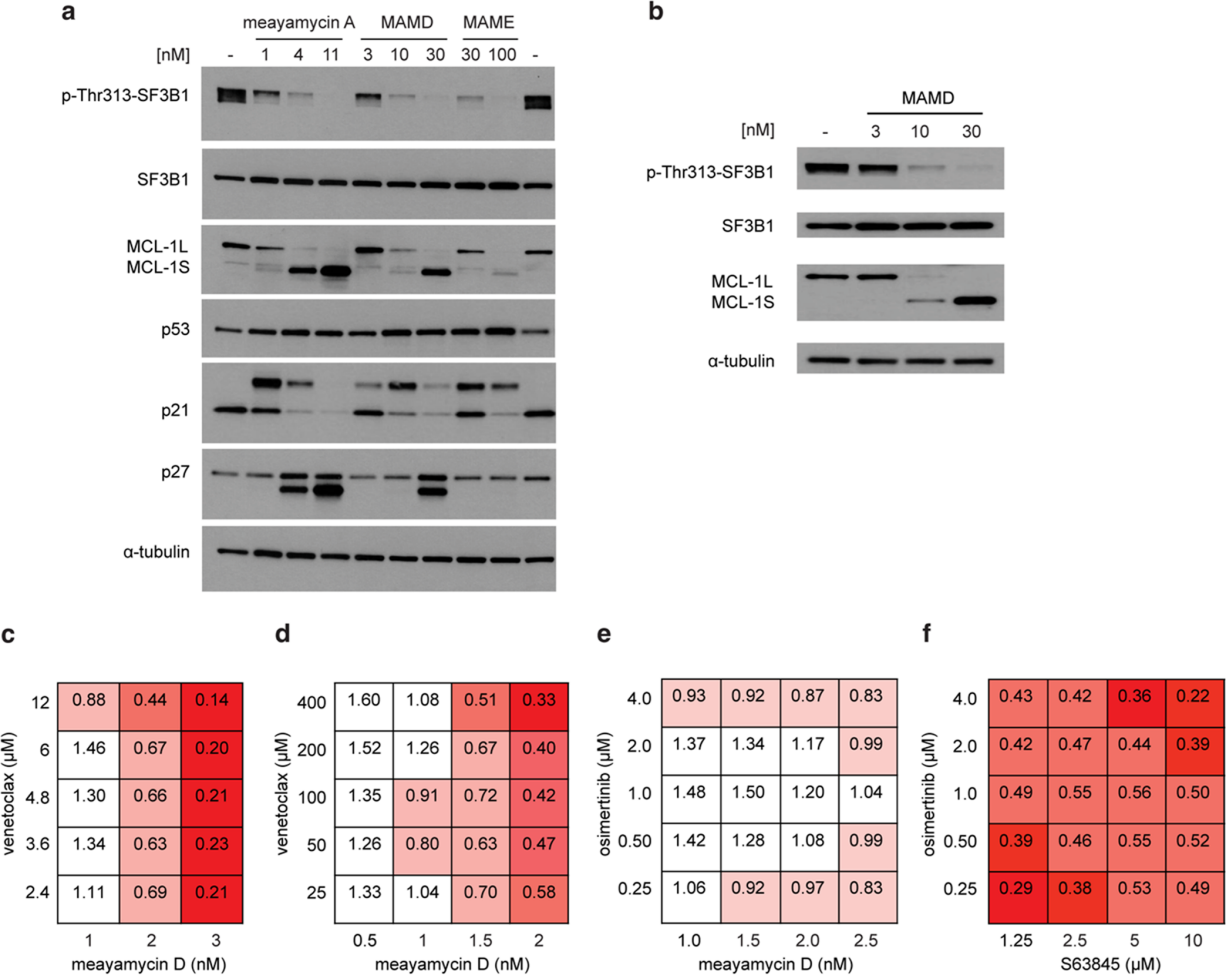
Western blot analysis of (a) HCT116 treated with meayamycin A, meayamycin D (MAMD), or meayamycin E (MAME) and (b) DMS114 cells treated with meayamycin D at the indicated nM concentrations. Synergistic antiproliferative activity (combination index values) of meayamycin D and venetoclax in DMS114 cells (c) and DMS53 cells (d) (*n* = 3). Synergy with the combination of osimertinib and meayamycin D (e) and osimertinib and S63845 (f) in HCC827AR cells (*n* = 3).

We evaluated the ability of meayamycin D and meayamycin E to alter RNA splicing, leading to differential protein expression. We observed a dose-dependent decrease in phosphorylation of Thr313 on SF3B1 (Figure 5a). Since SF3B1 is phosphorylated in the early stages of splicing, this result suggests that the compound is binding SF3B1 in the early stage of splicing.^42^ Meayamycin D and meayamycin E dose dependently decreased the expression of myeloid cell leukemia-1 (MCL-1L; long isoform) with a new band resulting from the alternatively spliced MCL-1S (short isoform). We previously showed that the protein and mRNA levels of MCL-1L and MCL-1S are correlated, indicating that the Western blot analysis of MCL-1 in the current study reflects their RNA transcripts.^43,44^ These meayamycin analogues also resulted in p27 truncation, previously reported by Yoshida et al.^11^ Similar results were observed in DMS114 cells (Figure 5b). Interestingly, we observed an alternatively spliced form of p21 (Figure S18). This larger p21 isoform was recently reported by the Lunec group, which demonstrated that when cells were treated with E7107 (pladienolide analogue), p21^L^ retained an intron and lost its nuclear localization signal, resulting in a loss of CDK inhibitory activity.^45^ In addition, they observed alternative splicing of p53 with the pladienolide analogue E7107, which we did not observe with the meayamycins. Further studies are warranted to validate alternative splicing of p21. Taken together, these results indicate that these new analogues affect alternative splicing in the same manner as meayamycin A.

Cancer cells can innately resist anticancer drugs by overexpressing antiapoptotic proteins such as BCL-2 and MCL-1L.^46^ Therefore, inhibiting both proteins kills cancer cells more effectively than inhibiting only one of them.^47^ We investigated the combination of venetoclax (FDA-approved BCL-2 inhibitor) and meayamycin D in both venetoclax-resistant small-cell lung cancer (SCLC) DMS114 cells and venetoclax-sensitive SCLC DMS53 cells. The Chou–Talalay method defines the additive effect as the combination index (CI) = 1, synergism as CI < 1, and antagonism as CI > 1.^48,49^Figure 5c,d shows that the drug combination was strongly synergistic (CI < 0.5).

Drug resistance poses a therapeutic challenge, especially in patients with advanced EGFR-mutated NSCLC. Despite FDA approval of a third-generation mutant-EGFR inhibitor (osimertinib), acquired resistance occurs in an EGFR-dependent and -independent manner.^50^ One of the EGFR-independent mechanisms may involve RNA splicing.^51^ Patients with high levels of alternative splicing events have poorer overall survival.^51^ Further, siRNA knockdown of *PHF5A*, which is overexpressed in NSCLC, reduced cell proliferation and induced apoptosis.^52^ Given these findings, we evaluated the combination of meayamycin D and osimertinib in osimertinib-resistant NSCLC HCC827AR cells. This drug combination was synergistic (CI = 0.8, Figure 5e) in these resistant cells. In addition, the MCL-1 inhibitor S63845 was strongly synergistic with osimertinib as previously demonstrated (Figure 5f).^53^

**Figure 6:**
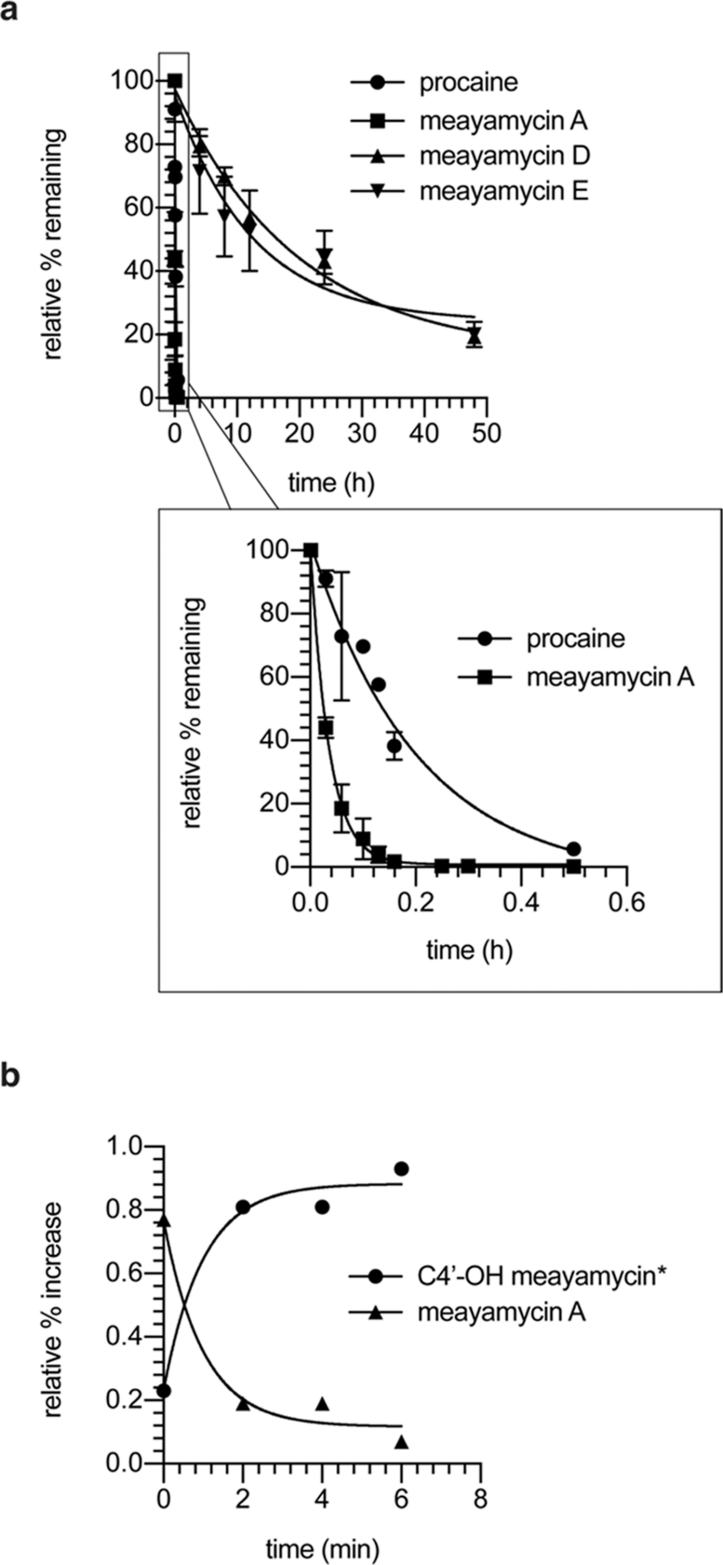
(a) In vitro plasma stability of meayamycin A, meayamycin D, and meayamycin E in mouse CD1 plasma over 48 h. *n* = 2. (b) Degradation of meayamycin A over 6 min. *The curve was determined by the appearance of species with *m*/*z* 464.3070 corresponding to C4′–OH meayamycin (calcd for [M + H]^+^ C_26_H_42_NO_6_ 464.3007).

Finally, we assessed the half-lives of meayamycin D and meayamycin E in mouse plasma using procaine as a positive control (Figure 6a). Plasma contains many nonspecific esterase enzymes that will rapidly cleave the acetyl group of meayamycin A, affecting its ADME properties. As expected, the hydrolysis of meayamycin A was nearly complete after 10 min (*t*_1/2_ = 1–2 min). Meanwhile, meayamycin D and meayamycin E were significantly more stable, with a half-life of 13 and 8 h, respectively. Furthermore, in the crude extracts of plasma treated with meayamycin A, we detected a mass peak corresponding to the acetate-hydrolysis product at the C4′ position. We also observed the time-dependent increase of this species (Figure 6b) that mirrors the decreasing signal of meayamycin A. While the rapid cleavage of the C4′-acetyl in meayamycin A is not surprising, it suggests that other FR901464 analogues such as spliceostatin A are also metabolically labile at that position. At the time of submission, there were no metabolic studies reported for spliceostatin A. However, our results suggest that spliceostatin A and other acetate-containing analogues are likely susceptible to nonspecific esterase enzymes. When analyzing the fraction of meamyamycin D and meayamycin E after 48 h, we did not detect the corresponding hydrolysis product, indicating the greater stability of MOM ether at the C4′-position in mouse plasma.

## Conclusions

In conclusion, we designed and synthesized four analogues to investigate the six-membered ring conformation of meayamycin. We acknowledge that the Pena group published the cocrystal structure of spliceostatin A-SF3B1-PHF5A during this study;^12^ however, their paper was not available during the period of the current study for the design of these analogues. Furthermore, our results substantiate the finding that the bound conformation of spliceostatin occurs in the N-axial conformation. Our results suggest that *N*-methylation induces twisting of the ring conformation and removing the C12 methyl group from *N*-methyl meayamycin D does not restore potency, highlighting a necessity for the N–H bond. Conformational analysis suggests that the amide-containing pyran of the natural product exists as a single conformer with the C14–N bond in the axial position. Additionally, structural information from the crystal structure provides insight into the reduced potency of the *N*-methyl analogues because of their deviated conformations. These results provide evidence for the N-axial chair conformation as the binding conformation and suggest that the amide N–H is essential for both the ring conformation and intracellular modulation of alternative splicing. Two new compounds emerge from this study highlighting linear alkyl ethers as suitable substitutions at the C4′ position while also revealing metabolic labilities of other FR901464 analogues. These new compounds exhibit potency comparable to meayamycin A and improved metabolic stability, the series of which we have named meayamycin D and meayamycin E. Meayamycin D and meayamycin E show modest but promising cancer selectivity against colon cancer cell lines, the generality of which must be further explored. Finally, the 1,2,4,5-all cis substituted tetrahydropyran with a secondary amide at the 4-position may be an L-shaped scaffold that medicinal chemists sought after to present substituents at the 1 and 4 positions with rigidity.^54^

## Experimental Section

### Chemistry

All reactions were carried out with freshly distilled solvents under anhydrous conditions unless otherwise noted. All flasks used for carrying out reactions were dried in an oven at 80 °C prior to use. Unless otherwise stated, all reactions that required heating used an oil bath as the heating source with a thermometer submerged in the bath to monitor the temperature. Unless specifically stated, the temperature of a water bath during the evaporation of organic solvents using a rotary evaporator was about 35 ± 5 °C. THF was distilled over Na metal and benzophenone. DCM was distilled over calcium hydride. MeCN was distilled over calcium hydride and stored over 3 Å molecular sieves. Yields refer to chromatographically and spectroscopically (^1^H NMR) homogeneous materials unless otherwise stated. All reactions were monitored by thin-layer chromatography (TLC) carried out on 0.25 mm Merck silica gel plates (60F-254) using UV light (254 nm) for visualization or anisaldehyde in ethanol or 0.2% ninhydrin in ethanol or 2.4% phosphomolybdic acid/1.4% phosphoric acid/5% sulfuric acid w/v in water as developing agents and heat for visualization. Silica gel (230–400 mesh) was used for flash column chromatography. NMR spectra were recorded on a Bruker ADVANCE spectrometer at 300, 400, 500, 600, or 700 MHz. The chemical shifts are given in parts per million (ppm) on a delta (δ) scale. The solvent peak was used as a reference value for ^1^H NMR: C*H*Cl_3_ = 7.26 ppm, C*H*_2_Cl_2_ = 5.32 ppm, C*H*_3_OH = 3.31 ppm, for ^13^C{^1^H} NMR: CDCl_3_ = 77.16 ppm, CD_2_Cl_2_ = 53.84 ppm. The following abbreviations are used to indicate the multiplicities: s = singlet; d = doublet; t = triplet; q = quartet; m = multiplet; br = broad. High-resolution mass spectra were recorded on a Thermo Scientific Q Exactive Orbitrap. The purity of all final compounds was determined to be >95% by liquid chromatography–mass spectrometry. Infrared (IR) spectra were collected on a PerkinElmer FT-IR Spectrum Two UATR spectrometer or Thermo Scientific Nicolet iS50R FT-IR spectrometer. Optical rotation was obtained on a Jasco P-2000 Digital Polarimeter, and [α]_D_^T^ values are given in deg × cm^3^ × g^–1^ × dm^–1^; concentrations, *c*, are listed in g × 100 × mL^–1^.

#### Ethyl-(*S*)-2-(Methoxymethoxy)propanoate (**2**)

A three-neck, 500 mL round-bottom flask equipped with a reflux condenser, an addition funnel, and a thermometer was purged with nitrogen, placed in a water bath at 23 °C, and treated with dimethoxymethane (100 mL, 1.11 mol, 1.0 equiv) and zinc bromide (6.4 mg, 28 μmol, 2.5 mol %). Acetyl chloride (81.0 mL, 1.11 mol, 1.0 equiv) was added dropwise over the course of 1 h to ensure the internal temperature did not exceed 35 °C. After 3 h at 35 °C, ethyl-(*S*)-lactate **1** (88.2 mL, 0.757 mol, 0.67 equiv) and diisopropylethylamine (168 mL, 0.946 mol, 0.83 equiv) were added. After 13 h at 23 °C, the mixture was poured into aqueous satd. NH_4_Cl (750 mL) and extracted with EtOAc (2 × 250 mL). The organic layers were combined, washed with water (1 × 250 mL) and brine (1 × 250 mL), dried over anhydrous Na_2_SO_4_, filtered, and concentrated under reduced pressure to afford ethyl (*S*)-2-(methoxymethoxy)propanoate **2** (117 g, 95% yield) as a pale-yellow oil. Data for ethyl (*S*)-2-(methoxymethoxy)propanoate **2** were consistent with those reported in the literature.^55^

#### Ethyl-2-(Bis(2-(*tert*-butyl)phenoxy)phosphoryl)acetate (**5**)

The preparation of ethyl 2-(bis(2-(*tert*-butyl)phenoxy)phosphoryl)acetate **5** followed the reported procedure.^56^

#### Ethyl-(*S*,*Z*)-4-(Methoxymethoxy)pent-2-enoate (**3**)

A 500 mL round-bottom flask equipped with an addition funnel and ethyl (*S*)-2-(methoxymethoxy)propanoate **2** (5.66 g, 34.9 mmol) was purged with nitrogen gas three times and then charged with DCM (87 mL). The reaction was cooled to −78 °C, and DIBALH (1.0 M in hexanes, 52 mL, 1.5 equiv) was added dropwise down the side of the flask over 1 h at −78 °C with an addition funnel. After stirring for 2 h at −78 °C, a separate 250 mL round-bottom flask with ethyl 2-(bis(2-(*tert*-butyl)phenoxy)phosphoryl)acetate **5** (16.6 g, 38.4 mmol, 1.1 equiv) was purged with nitrogen gas three times, and then charged with THF (48 mL) and cooled to 0 °C. Potassium *tert*-butoxide (4.19 g, 36.6 mmol, 1.05 equiv) was added in one portion to the flask with ethyl (*S*)-2-(methoxymethoxy)propanoate **2** at 0 °C. After 45 min at 0 °C, the solution of ethyl 2-(bis(2-(*tert*-butyl)phenoxy)phosphoryl)acetate **5** and potassium *tert*-butoxide was added to the addition funnel of the flask with ethyl (*S*)-2-(methoxymethoxy)propanoate **2** via cannula and added dropwise at −78 °C. The reaction mixture was slowly warmed to 23 °C. After 22 h at the same temperature, the reaction mixture was quenched with aqueous 1 M sodium citrate (150 mL) and stirred. After 21 h, the organic solvent was removed under reduced pressure, and the residue was extracted with EtOAc/hexanes (1:4, 2 × 120 mL) and washed with brine (1 × 120 mL) using a separatory funnel. The combined organic layers were dried over anhydrous Na_2_SO_4_, filtered, and concentrated under reduced pressure. The crude material was purified by flash chromatography (2–10% EtOAc in hexanes) on silica gel (350 mL) to afford ethyl (*S*,*Z*)-4-(methoxymethoxy)pent-2-enoate **3** (4.51 g, 69% yield, 1:99 E:Z) as a colorless oil. *R*_f_ = 0.32 (10% EtOAc/hexanes); IR (neat): *v*_max_ = 2980, 2937, 2889, 1718 (C=O), 1648, 1408, 1189, 1096, 1028, 920, 822 cm^–1^; [α]_D_^24^ −40.9 (c 1.0, CH_2_Cl_2_); ^1^H NMR (400 MHz, 293 K, CDCl_3_) δ 6.18 (dd, *J* = 11.7, 8.1 Hz, 1H), 5.78 (dd, *J* = 11.7, 1.2 Hz, 1H), 5.36–5.27 (m, 1H), 4.65 (d, *J* = 6.7 Hz, 1H), 4.62 (d, *J* = 6.7 Hz, 1H), 4.17 (q, *J* = 7.1 Hz, 2H), 3.36 (s, 3H), 1.35–1.24 (overlapping d and t, 6H); ^13^C NMR (100 MHz, 293 K, CDCl_3_) δ 165.9, 151.7, 119.8, 95.3, 69.8, 60.5, 55.6, 20.8, 14.4; HRMS (ESI^+^) calcd for C_9_H_16_O_4_Na [M + Na]^+^ 211.0941, found 211.0939.

#### (*S*,*Z*)-4-(Methoxymethoxy)pent-2-enoic Acid (**4**)

A 100 mL round-bottom flask with ethyl (*S*,*Z*)-4-(methoxymethoxy)pent-2-enoate **3** (3.33 g, 17.7 mmol), open to air, in methanol (9.31 mL) was cooled to 0 °C. Aqueous 1.0 M NaOH (44.2 mL, 2.5 equiv) was added dropwise at 0 °C. The resulting mixture was warmed to 23 °C. After stirring for 2.5 h at the same temperature, the mixture was acidified with aqueous 4 M HCl to approximately pH 4. The resulting solution was extracted with EtOAc (4 × 50 mL) using a separatory funnel. The combined organic layers were dried over anhydrous Na_2_SO_4_, filtered, and concentrated under reduced pressure to afford (*S*,*Z*)-4-(methoxymethoxy)pent-2-enoic acid **4** (2.73 g, 96% yield) as a colorless oil. *R*_f_ = 0.46 (1% AcOH in EtOAc); IR (neat): *v*_max_ = 3126, 2981, 2936, 2892, 1699 (C=O), 1644, 1435, 1097, 1028, 920, 830 cm^–1^; [α]_D_^24^ −37.6 (c 1.0, CH_2_Cl_2_); ^1^H NMR (400 MHz, 293 K, CDCl_3_) δ 6.32 (dd, *J* = 11.8, 8.1 Hz, 1H), 5.81 (dd, *J* = 11.8, 1.3 Hz, 1H), 5.33–5.23 (m, 1H), 4.65 (app s, 2H), 3.38 (s, 3H), 1.32 (d, *J* = 6.5 Hz, 3H); ^13^C NMR (100 MHz, 293 K, CDCl_3_) δ 170.7, 154.2, 119.0, 95.4, 70.2, 55.7, 20.7; HRMS (ESI^–^) calcd for C_7_H_11_O_4_ [M – H]^−^ 159.0652, found 159.0700.

#### *tert*-Butyl-((2R,3R,5S,6S)-6-allyl-2,5-dimethyltetrahydro-2*H*-pyran-3-yl)carbamate (**6**)

The preparation of *tert*-butyl ((2*R*,3*R*,5*S*,6*S*)-6-allyl-2,5-dimethyltetrahydro-2*H*-pyran-3-yl)carbamate **6** followed the reported procedure.^33^

#### (2*R*,3*R*,5*S*,6*S*)-6-Allyl-2,5-dimethyltetrahydro-2*H*-pyran-3-amine (**7**)

The preparation of (2*R*,3*R*,5*S*,6*S*)-6-allyl-2,5-dimethyltetrahydro-2*H*-pyran-3-amine **7** followed the reported procedure.^33^

#### (*S*,*Z*)-*N*-((2*R*,3*R*,5*S*,6*S*)-6-Allyl-2,5-dimethyltetrahydro-2*H*-pyran-3-yl)-4-(methoxymethoxy)pent-2-enamide (**8**)

A 25 mL round-bottom flask with (2*R*,3*R*,5*S*,6*S*)-6-allyl-2,5-dimethyltetrahydro-2*H*-pyran-3-amine **7** (196 mg, 1.16 mmol) was purged with nitrogen and then charged with DCM (3.5 mL), (*S*,*Z*)-4-(methoxymethoxy)pent-2-enoic acid **4** (186 mg, 1.16 mmol, 1.0 equiv), and diisopropylethylamine (570 μL, 3.48 mmol, 3.0 equiv) at 23 °C. The resulting solution was cooled to 0 °C, and HATU (441 mg, 1.16 mmol, 1.0 equiv) was added at 0 °C. After 1 h at 0 °C, the mixture was warmed to 23 °C. After 18 h, the reaction mixture was quenched with aqueous satd. NH_4_Cl (5 mL) and extracted with EtOAc (2 × 10 mL) using a separatory funnel. The combined organic layers were dried over anhydrous Na_2_SO_4_, filtered, and concentrated under reduced pressure. The crude material was purified by flash chromatography (10–30% EtOAc in hexanes) on silica gel (70 mL) to afford (*S*,*Z*)-*N*-((2*R*,3*R*,5*S*,6*S*)-6-allyl-2,5-dimethyltetrahydro-2*H*-pyran-3-yl)-4-(methoxymethoxy)pent-2-enamide **8** (221 mg, 61% yield) as a white solid. mp = 61.0–63.4 °C; *R*_f_ = 0.41 (40% EtOAc/hexanes); IR (film): *v*_max_ = 3436, 3054, 2982, 2936, 1668 (C=O), 1642, 1503, 1266, 1032, 738 cm^–1^; [α]_D_^24^ −95.2 (c 1.0, CH_2_Cl_2_); ^1^H NMR (500 MHz, 293 K, CDCl_3_) δ 5.97 (dd, *J* = 11.6, 8.2 Hz, 1H), 5.84–5.74 (m, 2H), 5.70 (dd, *J* = 11.7, 1.2 Hz, 1H), 5.44–5.36 (m, 1H), 5.12 (dq, *J* = 17, 1.6 Hz, 1H), 5.05 (br d, *J* = 10.2 Hz, 1H), 4.67 (d, *J* = 6.7 Hz, 1H), 4.62 (d, *J* = 6.7 Hz, 1H), 3.96–3.90 (m, 1H), 3.66 (qd, *J* = 6.4, 2.1 Hz, 1H), 3.54 (ddd, *J* = 7.3, 7.3, 2.8 Hz, 1H), 3.37 (s, 3H), 2.38–2.29 (m, 1H), 2.17–2.09 (m, 1H), 1.94 (t, *J* = 3.6 Hz, 2H), 1.82–1.74 (m, 1H), 1.33 (d, *J* = 6.5 Hz, 3H), 1.14 (d, *J* = 6.5 Hz, 3H), 1.02 (d, *J* = 7.4 Hz, 3H); ^13^C NMR (100 MHz, 293 K, CDCl_3_) δ 165.2, 147.3, 134.8, 122.4, 116.9, 95.2, 80.9, 76.1, 69.7, 55.6, 47.2, 37.5, 36.0, 29.0, 21.1, 18.0, 15.2; HRMS (ESI+) calcd for C_17_H_30_NO_4_ [M + H]^+^ 312.2169, found 312.2179.

#### (*S*,*Z*)-*N*-((2*R*,3*R*,5*S*,6*S*)-2,5-Dimethyl-6-((*E*)-3-methyl-4-oxobut-2-en-1-yl)tetrahydro-2H-pyran-3-yl)-4-(methoxymethoxy)pent-2-enamide (**9**)

A 2 mL sealed tube with (*S*,*Z*)-*N*-((2*R*,3*R*,5*S*,6*S*)-6-allyl-2,5-dimethyltetrahydro-2*H*-pyran-3-yl)-4-(methoxymethoxy)pent-2-enamide **8** (100 mg, 0.321 mmol) was placed under a flow of argon gas and then charged with methacrolein (638 μL, 77.71 mmol, 24 equiv) and nitro-Grela catalyst (11 mg, 0.016 mmol, 5 mol %). The sealed tube was capped. After 23 h at 40 °C (external temperature), the reaction was cooled to 23 °C. The crude contents were transferred to a separate 10 mL pear-shaped flask, concentrated under reduced pressure, and purified by flash chromatography (20–60% EtOAc in hexanes) on silica gel (50 mL) to afford (*S*,*Z*)-*N*-((2*R*,3*R*,5*S*,6*S*)-2,5-dimethyl-6-((*E*)-3-methyl-4-oxobut-2-en-1-yl)tetrahydro-2*H*-pyran-3-yl)-4-(methoxymethoxy)pent-2-enamide **9** (84 mg, 74% yield, 95:5 E:Z) as a light-brown oil. *R*_f_ = 0.23 (40% EtOAc/hexanes); IR (film): *v*_max_ = 3344, 2975, 2931, 2885, 2855, 1680 (C=O), 1672 (C=O), 1639, 1519, 1098, 1068, 1033 cm^–1^; [α]_D_^24^ −76.6 (c 0.5, CH_2_Cl_2_); ^1^H NMR (400 MHz, 293 K, CDCl_3_) δ 9.34 (s, 1H), 6.58–6.49 (m, 1H), 5.99 (dd, *J* = 11.6, 8.2 Hz, 1H), 5.78 (d, *J* = 10.3 Hz, 1H), 5.72 (dd, *J* = 11.6, 1.1 Hz, 1H), 5.45–5.33 (m, 1H), 4.68 (d, *J* = 6.7 Hz, 1H), 4.62 (d, *J* = 6.7 Hz, 1H), 4.00–3.91 (m, 1H), 3.75–3.60 (m, 2H), 3.37 (s, 3H), 2.65–2.51 (m, 1H), 2.48–2.34 (m, 1H), 2.02–1.94 (m, 2H), 1.90–1.79 (m, 1H), 1.77 (s, 3H), 1.33 (d, *J* = 6.4 Hz, 3H), 1.15 (d, *J* = 6.4 Hz, 3H), 1.06 (d, *J* = 7.4 Hz, 3H); ^13^C NMR (100 MHz, 293 K, CDCl_3_) δ 195.2, 165.2, 150.5, 147.4, 140.7, 122.3, 95.1, 79.9, 76.3, 69.7, 55.5, 47.0, 35.8, 32.9, 29.6, 21.1, 17.9, 15.3, 9.6; HRMS (ESI+) calcd for C_19_H_32_NO_5_ [M + H]^+^ 354.2275, found 354.2284.

#### (*S*,*Z*)-*N*-((2R,3R,5S,6S)-2,5-Dimethyl-6-((*E*)-3-methylpenta-2,4-dien-1-yl)tetrahydro-2*H*-pyran-3-yl)-4-(methoxymethoxy)pent-2-enamide (**10**)

A 25 mL round-bottom flask with methyltriphenylphosphonium bromide (503 mg, 1.38 mmol, 2.5 equiv) was purged with nitrogen gas three times, charged with THF (4.6 mL), and cooled to 0 °C. Potassium *tert*-butoxide (145 mg, 1.27 mmol, 2.3 equiv) was added at 0 °C. After 25 min at 0 °C, (*S*,*Z*)-*N*-((2*R*,3*R*,5*S*,6*S*)-2,5-dimethyl-6-((*E*)-3-methyl-4-oxobut-2-en-1-yl)tetrahydro-2*H*-pyran-3-yl)-4-(methoxymethoxy)pent-2-enamide **9** (195 mg, 0.551 mmol) in THF (1 mL) was added, rinsed with THF (0.5 mL), and the mixture was warmed to 23 °C. After 2 h at 23 °C, the reaction was quenched with aqueous satd. NH_4_Cl (7 mL). The mixture was separated, and the aqueous layer was extracted with EtOAc (3 × 10 mL) using a separatory funnel. The combined organic layers were washed with brine (1 × 10 mL), dried over anhydrous Na_2_SO_4_, filtered, and concentrated under reduced pressure. The crude material was purified by flash chromatography (10–30% EtOAc in hexanes) on silica gel (40 mL) to afford (*S*,*Z*)-*N*-((2*R*,3*R*,5*S*,6*S*)-2,5-dimethyl-6-((*E*)-3-methylpenta-2,4-dien-1-yl)tetrahydro-2*H*-pyran-3-yl)-4-(methoxymethoxy)pent-2-enamide **10** (166 mg, 86% yield) as a brown oil. *R*_f_ = 0.45 (40% EtOAc/hexanes); IR (neat): *v*_max_ = 3342, 2931, 2980, 1663 (C=O), 1631, 1505, 1097, 1062, 1030 cm^–1^; [α]_D_^24^ −79.4 (c 1.0, CH_2_Cl_2_); ^1^H NMR (400 MHz, 293 K, CDCl_3_) δ 6.35 (dd, *J* = 17.3, 10.7 Hz, 1H), 5.97 (dd, *J* = 11.6, 8.2 Hz, 1H), 5.79 (d, *J* = 9.2 Hz, 1H), 5.70 (dd, *J* = 11.6, 1.0 Hz, 1H), 5.46 (t, *J* = 7.2 Hz, 1H), 5.43–5.35 (m, 1H), 5.11 (d, *J* = 17.4 Hz, 1H), 4.96 (d, *J* = 10.6 Hz, 1H), 4.68 (d, *J* = 6.7 Hz, 1H), 4.62 (d, *J* = 6.6 Hz, 1H), 3.98–3.89 (m, 1H), 3.66 (qd, *J* = 6.4, 2.1 Hz, 1H), 3.54 (ddd, *J* = 7.3, 7.3, 2.8 Hz, 1H), 3.37 (s, 3H), 2.46–2.34 (m, 1H), 2.31–2.19 (m, 1H), 2.00–1.99 (m, 2H), 1.86–1.72 (m, 1H), 1.76 (s, 3H), 1.33 (d, *J* = 6.4 Hz, 3H), 1.14 (d, *J* = 6.4 Hz, 3H), 1.02 (d, *J* = 7.4 Hz, 3H); ^13^C NMR (100 MHz, 293 K, CDCl_3_) δ 165.2, 147.3, 141.4, 135.8, 128.2, 122.4, 111.3, 95.2, 81.0, 76.2, 69.8, 55.5, 47.2, 36.0, 32.0, 29.1, 21.1, 18.0, 15.3, 12.1; HRMS (ESI^+^) calcd for C_20_H_34_NO_4_ [M + H]^+^ 352.2488, found 352.2489.

#### (3*R*,4*R*,5*R*)-7,7-Dimethyl-5-vinyl-1,6-dioxaspiro[2.5]octan-4-ol (**24**)

The preparation of (3*R*,4*R*,5*R*)-7,7-dimethyl-5-vinyl-1,6-dioxaspiro[2.5]octan-4-ol **24** followed the reported procedure.^57^

##### Meayamycin D

A 2 mL sealed tube was treated with (*S*,*Z*)-*N*-((2*R*,3*R*,5*S*,6*S*)-2,5-dimethyl-6-((*E*)-3-methylpenta-2,4-dien-1-yl)tetrahydro-2*H*-pyran-3-yl)-4-(methoxymethoxy)pent-2-enamide **10** (56.6 mg, 0.161 mmol) in DCE (302 μL). (3*R*,4*R*,5*R*)-7,7-Dimethyl-5-vinyl-1,6-dioxaspiro[2.5]octan-4-ol **24** in DCE (148 μL, 100 mg/mL solution, 0.5 equiv) and nitro-Grela catalyst (5.4 mg, 8.0 μmol, 5 mol %) were added to the sealed tube at 23 °C, and the sealed tube was purged with argon. The sealed tube was heated to 50 °C. After 2 h at 50 °C, additional right-hand fragment **24** in DCE (148 μL, 100 mg/mL solution, 0.5 equiv) was added. After an additional 2 h at 50 °C, right-hand fragment **24** in DCE (148 μL, 100 mg/mL, 0.5 equiv) and nitro-Grela catalyst (5.4 mg, 8.0 μmol, 5 mol %) were added. After an additional 4 h at 50 °C, the reaction was cooled to 23 °C and concentrated under reduced pressure. The crude material was purified by flash chromatography (20–70% EtOAc in hexanes) on silica gel (7 mL) to afford a complex mixture, which was further purified by preparative TLC (60% EtOAc in hexanes). Unreacted (*S*,*Z*)-*N*-((2*R*,3*R*,5*S*,6*S*)-2,5-dimethyl-6-((*E*)-3-methylpenta-2,4-dien-1-yl)tetrahydro-2*H*-pyran-3-yl)-4-(methoxymethoxy)pent-2-enamide **10** and right-hand fragment **24** were resubmitted to the reaction conditions and purified by preparative TLC (60% EtOAc in hexanes). The product mixtures were combined and dissolved in DCM (5 mL), and charcoal (1.5 g, 50× by weight) was added. After 3 h, the mixture was filtered through Celite and the filtrate was concentrated under reduced pressure, and further purified by HPLC to afford meayamycin D (3.5 mg*, 4% yield) as a white solid. *R*_f_ = 0.25 (60% EtOAc/hexanes); IR (neat): *v*_max_ = 3367, 2922, 2854, 1662 (C=O), 1513, 1460, 1375, 1266, 1110, 1033 cm^–1^; ^1^H NMR (600 MHz, 293 K, CD_2_Cl_2_) δ 6.34 (d, *J* = 15.7 Hz, 1H), 5.93 (dd, *J* = 11.5, 8.2 Hz, 1H), 5.80 (d, *J* = 9.1 Hz, 1H), 5.72 (app d, *J* = 11.5 Hz, 1H), 5.64 (dd, *J* = 15.7, 6.6 Hz, 1H), 5.52 (t, *J* = 6.9 Hz, 1H), 5.38–5.28 (m, 1H), 4.62 (d, *J* = 6.7 Hz, 1H), 4.56 (d, *J* = 6.7 Hz, 1H), 3.96 (dd, *J* = 9.5, 6.7 Hz, 1H), 3.91–3.85 (m, 1H), 3.65 (qd, *J* = 6.5, 2.3 Hz, 1H), 3.53 (ddd, *J* = 7.3, 7.3, 2.6 Hz, 1H), 3.48 (dd, *J* = 10.0, 10.0 Hz, 1H), 3.33 (s, 3H), 2.96 (d, *J* = 4.7 Hz, 1H), 2.46 (d, *J* = 4.7 Hz, 1H), 2.40–2.32 (m, 1H), 2.25–2.19 (m, 1H), 2.17 (d, *J* = 14.3 Hz, 1 H), 1.96–1.87 (m, 2H), 1.81–1.74 (m, 1H), 1.78 (s, 3H), 1.60 (d, *J* = 10.4 Hz, 1H), 1.40 (d, *J* = 14.3 Hz, 1H), 1.36 (s, 3H), 1.26 (d, *J* = 6.4 Hz, 3H), 1.23 (s, 3H), 1.10 (d, *J* = 6.4 Hz, 3H), 1.01 (d, *J* = 7.3 Hz, 3H); ^13^C NMR (150 MHz, 293 K, CD_2_Cl_2_) δ 165.2, 147.0, 137.8, 129.5, 125.9, 122.9, 95.2, 81.2, 76.3, 74.9, 73.0, 69.5, 68.6, 57.8, 55.5, 47.8, 47.4, 43.1, 36.2, 32.4, 31.1, 31.0, 29.6, 23.7, 21.1, 18.0, 15.4, 12.8; HRMS (ESI^+^) calcd for C_28_H_46_NO_7_ [M + H]^+^ 508.3269, found 508.3258. *Yield was calculated using Beer–Lambert law (*A* = ε × *c* × *l*) and the UV absorbance reading at 235 nm on a Shimadzu UV-1280 with molar absorptivity (ε) value as reported.^1^

#### (2*R*,3*R*,5*S*,6*S*)-6-Allyl-*N*,2,5-trimethyltetrahydro-2*H*-pyran-3-amine (**16**)

A 50 mL round-bottomed flask with a reflux condenser was purged with nitrogen gas three times, charged with THF (10 mL), and cooled to 0 °C. Lithium aluminum hydride (80 mg, 2.10 mmol, 3 equiv) was added at 0 °C. After 10 min, *tert*-butyl ((2*R*,3*R*,5*S*,6*S*)-6-allyl-2,5-dimethyltetrahydro-2*H*-pyran-3-yl)carbamate **6** (189 mg, 0.700 mmol) was added dropwise in a solution of THF (3 mL) and the reaction was heated to reflux in an oil bath. After 4 h at reflux, the reaction mixture was removed from the oil bath, cooled to 0 °C, and quenched with dropwise addition of EtOAc (2 mL). Aqueous 1.0 M NaOH was added dropwise at 0 °C until the precipitation of a white solid ceased. The reaction mixture was filtered through Celite and diluted with satd. aqueous sodium bicarbonate (10 mL). The aqueous layer was extracted with DCM (3 × 10 mL) using a separatory funnel. The combined organic layers were dried over anhydrous Na_2_SO_4_, filtered, and concentrated under reduced pressure to give (2*R*,3*R*,5*S*,6*S*)-6-allyl-*N*,2,5-trimethyltetrahydro-2*H*-pyran-3-amine **16** (115 mg) as a gold oil which was used directly without further purification. For characterization, a small portion was dissolved in 1 N HCl, and EtOAc (5 mL) was added to the solution. The layers were separated, and the aqueous layer was basified by the addition of solid NaHCO_3_. The resulting mixture was extracted with a mixture of 10% MeOH/DCM (2 × 5 mL), dried over anhydrous Na_2_SO_4_, filtered, and concentrated under reduced pressure to afford (2*R*,3*R*,5*S*,6*S*)-6-allyl-*N*,2,5-trimethyltetrahydro-2*H*-pyran-3-amine **16**. *R*_f_ = 0.22 (10% MeOH/DCM); IR (neat): *v*_max_ = 3377, 2931, 2850, 1642, 1447, 1372, 1319, 1127, 1076, 912 cm^–1^; [α]_D_^25^ −28.2 (c 1.0, CH_2_Cl_2_); ^1^H NMR (300 MHz, 293 K, 1% CD_3_OD in CDCl_3_) δ 5.81 (dddd, *J* = 17.1, 10.2, 7.7, 6.1 Hz, 1H), 5.16–4.96 (m, 2H), 3.62 (qd, *J* = 6.5, 2.3 Hz, 1H), 3.50 (ddd, *J* = 7.2, 7.2, 2.7 Hz, 1H), 2.46 (s, 3H), 2.44–2.39 (m, 1H), 2.39–2.28 (m, 1H), 2.16–2.05 (m, 2H), 1.74–1.56 (m, 2H), 1.25 (d, *J* = 6.6 Hz, 3H), 1.09 (d, *J* = 7.3 Hz, 3H); ^13^C NMR (100 MHz, 293 K, 1% CD_3_OD in CDCl_3_) δ 135.4, 116.5, 81.0, 77.7, 57.3, 37.6, 34.9, 32.9, 29.6, 18.3, 14.4; HRMS (ESI^+^) calcd for C_11_H_22_NO [M + H]^+^ 184.1696, found 184.1698.

#### (*S*,*Z*)-*N*-((2*R*,3*R*,5*S*,6*S*)-6-Allyl-2,5-dimethyltetrahydro-2*H*-pyran-3-yl)-4-(methoxymethoxy)-*N*-methylpent-2-enamide (**17**)

A 25 mL round-bottom flask with (2*R*,3*R*,5*S*,6*S*)-6-allyl-*N*,2,5-trimethyltetrahydro-2*H*-pyran-3-amine **16** (1.16 g, 6.33 mmol) was purged with nitrogen and then charged with DCM (20 mL), (*S*,*Z*)-4-(methoxymethoxy)pent-2-enoic acid **4** (726 mg, 4.53 mmol, 0.7 equiv), and diisopropylethylamine (3.29 mL, 19.0 mmol, 3.0 equiv) at 23 °C. The resulting solution was cooled to 0 °C. After 10 min at 0 °C, HATU (2.41 g, 6.33 mmol, 1.0 equiv) was added at 0 °C. The mixture was warmed to 23 °C. After 20 h, the reaction mixture was quenched with aqueous satd. NH_4_Cl (15 mL) and extracted with DCM (3 × 15 mL) using a separatory funnel. The combined organic layers were washed with brine (1 × 15 mL), dried over anhydrous Na_2_SO_4_, filtered, and concentrated under reduced pressure. The crude material was purified by flash chromatography (10–30% EtOAc in hexanes) on silica gel (70 mL) to afford (*S*,*Z*)-*N*-((2*R*,3*R*,5*S*,6*S*)-6-allyl-2,5-dimethyltetrahydro-2*H*-pyran-3-yl)-4-(methoxymethoxy)-*N*-methylpent-2-enamide **17** (815 mg, 55% yield, 66:34 *s*-cis:*s*-trans amides) as a pale-yellow oil. The *s*-cis amide was defined by selective one-dimensional (1D) NOESY of the N-Me signal (see Supporting Information, S62 and S63). *R*_f_ = 0.26 (40% EtOAc/hexanes); IR (neat): *v*_max_ = 2978, 2931, 2882, 2850, 1627 (C=O), 1442, 1321, 1096, 1028, 917 cm^–1^; [α]_D_^24^ −104.0 (c 1.0, CH_2_Cl_2_); ^1^H NMR (600 MHz, 293 K, CDCl_3_) δ 6.13 (d, *J* = 11.7 Hz, 1H), 6.04 (d, *J* = 11.7 Hz, 0.5H), 5.92–5.81 (m, 3H), 5.13–5.08 (m, 1.5H), 5.07–5.02 (m, 1.5H), 4.93–4.87 (m, 1H), 4.81–4.74 (m, 0.5H), 4.67 (d, *J* = 6.7 Hz, 1H), 4.64 (d, *J* = 6.6 Hz, 0.5H), 4.61–4.55 (m, 2.5H), 3.88 (qd, *J* = 6.8, 4.9 Hz, 1H), 3.89–3.83 (m, 1H), 3.72–3.65 (m, 1.5H), 3.35 (s, 3H), 3.33 (s, 1.5H), 3.14 (s, 3H), 3.07 (s, 3H), 2.34–2.26 (m, 1.5H), 2.23–2.15 (m, 1.5H), 2.00–1.86 (m, 3H), 1.74–1.67 (m, 1.5H), 1.34 (d, *J* = 6.4 Hz, 1.5H), 1.32 (d, *J* = 6.5 Hz, 3H), 1.16 (d, *J =* 6.1 Hz, 1.5H), 1.15 (d, *J* = 6.7 Hz, 3H), 0.98 (d, *J* = 7.3 Hz, 1.5H), 0.97 (d, *J* = 7.1 Hz, 3H); ^13^C NMR (100 MHz, 293 K, CDCl_3_) δ 167.9, 167.7, 143.3, 143.2, 135.6, 135.5, 122.9, 122.7, 116.8, 116.6, 95.0, 94.9, 79.2, 79.0, 73.5, 73.3, 70.7, 70.0, 55.44, 55.35, 50.3, 36.3, 36.1, 33.8, 33.1, 32.3, 31.2, 28.7, 28.6, 21.2, 21.1, 17.4, 17.2, 15.9, 15.7; HRMS (ESI^+^) calcd for C_18_H_32_NO_4_ [M + H]^+^ 326.2326, found 326.2341. Due to the frequent overlap of unresolved rotamer peaks, multiplicities and coupling constants for this molecule are not calculated for every signal. The signals for both rotamers are reported.

#### (*S*,*Z*)-*N*-((2*R*,3*R*,5*S*,6*S*)-2,5-Dimethyl-6-((*E*)-3-methyl-4-oxobut-2-en-1-yl)tetrahydro-2H-pyran-3-yl)-4-(methoxymethoxy)-*N*-methylpent-2-enamide (**18**)

A 5 mL sealed tube with (*S*,*Z*)-*N*-((2*R*,3*R*,5*S*,6*S*)-6-allyl-2,5-dimethyltetrahydro-2*H*-pyran-3-yl)-4-(methoxymethoxy)-*N*-methylpent-2-enamide **17** (527 mg, 1.62 mmol) was placed under a flow of argon gas and then charged with methacrolein (3.21 mL, 38.9 mmol, 24 equiv) and nitro-Grela catalyst (54 mg, 81 μmol, 5 mol %). The sealed tube was capped and heated in an oil bath until the external temperature reached 40 °C. After 14 h at 40 °C, the reaction mixture was cooled to 23 °C. The crude contents were transferred to a separate 10 mL pear-shaped flask, concentrated under reduced pressure, and purified by flash chromatography (20–65% EtOAc in hexanes) on silica gel (50 mL) to afford (*S*,*Z*)-*N*-((2*R*,3*R*,5*S*,6*S*)-2,5-dimethyl-6-((*E*)-3-methyl-4-oxobut-2-en-1-yl)tetrahydro-2*H*-pyran-3-yl)-4-(methoxymethoxy)-*N*-methylpent-2-enamide **18** (239 mg, 40% yield, 99:1 E:Z, 68:32 *s*-cis:*s*-trans amides) as a dark-brown oil. The *s*-cis amide was defined by selective 1D NOESY of (*S*,*Z*)-*N*-((2*R*,3*R*,5*S*,6*S*)-6-allyl-2,5-dimethyltetrahydro-2*H*-pyran-3-yl)-4-(methoxymethoxy)-*N*-methylpent-2-enamide **17**. *R*_f_ = 0.26 (60% EtOAc/hexanes); IR (film): *v*_max_ = 2968, 2930, 2884, 1684 (C=O), 1623 (C=O), 1442, 1321, 1096, 1028 cm^–1^; [α]_D_^23^ −80.1 (c 0.5, CH_2_Cl_2_); ^1^H NMR (600 MHz, 293 K, CDCl_3_) δ 9.435 (s, 0.5H), 9.428 (s, 1H), 6.68–6.58 (m, 1.5H), 6.13 (d, *J* = 11.6 Hz, 1H), 6.04 (d, *J* = 11.6 Hz, 0.5H), 5.89 (dd, *J* = 11.6, 8.5 Hz, 1H), 5.86 (dd, *J* = 11.6, 8.6 Hz, 0.5H), 4.94–4.87 (m, 1H), 4.81–4.74 (m, 0.5H), 4.70–4.62 (m, 2.5H), 4.62–4.55 (m, 1.5H), 3.95–3.87 (m, 2H), 3.87–3.81 (m, 1.5H), 3.35 (s, 3H), 3.33 (s, 1.5H), 3.12 (s, 3H), 3.05 (s, 1.5H), 2.59–2.50 (m, 1.5H), 2.50–2.41 (m, 1.5H), 2.09–1.99 (m, 1.5H), 1.95–1.84 (m, 1.5H), 1.76 (s, 4.5H), 1.75–1.66 (m, 1.5H), 1.34 (d, *J* = 6.4 Hz, 1.5H), 1.32 (d, *J* = 6.4 Hz, 3H), 1.18–1.10 (m, 4.5H), 1.04–0.96 (m, 4.5H); ^13^C NMR (150 MHz, 293 K, CDCl_3_) δ 195.3, 195.2, 167.9, 167.7, 151.6, 151.3, 143.5, 143.4, 140.5, 140.4, 122.7, 122.5, 95.0, 94.9, 78.0, 77.8, 73.2, 73.0, 70.6, 69.9, 55.5, 55.4, 50.3, 33.6. 32.3, 31.4, 31.3, 31.1, 31.0, 29.8, 29.0, 28.9, 21.2, 21.1, 17.2, 17.0, 16.1, 16.05, 15.95, 9.6; HRMS (ESI+) calcd for C_20_H_34_NO_5_ [M + H]^+^ 368.2432, found 368.2440. Due to the frequent overlap of unresolved rotamer peaks, multiplicities and coupling constants for this molecule are not calculated for every signal. The signals for both rotamers are reported.

#### (*S*,*Z*)-*N*-((2*R*,3*R*,5*S*,6*S*)-2,5-Dimethyl-6-((*E*)-3-methylpenta-2,4-dien-1-yl)tetrahydro-2*H*-pyran-3-yl)-4-(methoxymethoxy)-*N*-methylpent-2-enamide (**19**)

A 25 mL round-bottom flask with methyltriphenylphosphonium bromide (582 mg, 1.63 mmol, 2.5 equiv) was purged with nitrogen gas three times, charged with THF (5 mL), and cooled to 0 °C. Potassium *tert*-butoxide (168 mg, 1.45 mmol, 2.3 equiv) was added at 0 °C. After 30 min at 0 °C, (*S*,*Z*)-*N*-((2*R*,3*R*,5*S*,6*S*)-2,5-dimethyl-6-((*E*)-3-methyl-4-oxobut-2-en-1-yl)tetrahydro-2*H*-pyran-3-yl)-4-(methoxymethoxy)-*N*-methylpent-2-enamide **18** (239 mg, 0.651 mmol) in THF (1.5 mL) was added, rinsed with additional THF (1 mL), and the mixture was warmed to 23 °C. After 2.5 h at 23 °C, the reaction mixture was quenched with aqueous satd. NH_4_Cl (10 mL). The mixture was concentrated under reduced pressure to remove residual THF. EtOAc (5 mL) was added to the crude mixture, and the layers were separated. The aqueous layer was extracted with EtOAc (2 × 10 mL) using a separatory funnel. The combined organic layers were washed with brine (1 × 10 mL), dried over anhydrous Na_2_SO_4_, filtered, and concentrated under reduced pressure. The crude material was purified by flash chromatography (10–25% EtOAc in hexanes) on silica gel (30 mL) to afford (*S*,*Z*)-*N*-((2*R*,3*R*,5*S*,6*S*)-2,5-dimethyl-6-((*E*)-3-methylpenta-2,4-dien-1-yl)tetrahydro-2*H*-pyran-3-yl)-4-(methoxymethoxy)-*N*-methylpent-2-enamide **19** (207 mg, 87% yield, 66:34 *s*-cis:*s*-trans amides) as a brown oil. The *s*-cis amide was defined by selective 1D NOESY of (*S*,*Z*)-*N*-((2*R*,3*R*,5*S*,6*S*)-6-allyl-2,5-dimethyltetrahydro-2*H*-pyran-3-yl)-4-(methoxymethoxy)-*N*-methylpent-2-enamide **17**. *R*_f_ = 0.33 (40% EtOAc/hexanes); IR (neat): *v*_max_ = 2928, 2886, 2854, 1627 (C=O), 1442, 1321, 1097, 1030, 919 cm^–1^; [α]_D_^23^ −81.8 (c 1.0, CH_2_Cl_2_); ^1^H NMR (600 MHz, 293 K, CDCl_3_) δ 6.385 (dd, *J* = 17.2, 10.8 Hz, 0.5H), 6.380 (dd, *J* = 17.3, 10.6, 1H), 6.13 (d, *J* = 11.8 Hz, 1H), 6.04 (d, *J* = 11.8 Hz, 0.5H), 5.88 (dd, *J* = 11.6, 8.5 Hz, 1H), 5.84 (dd, *J* = 11.6, 8.5 Hz, 0.5H), 5.59–5.52 (m, 1.5H), 5.11 (d, *J* = 17.5 Hz, 0.5H), 5.10 (d, *J* = 17.4 Hz, 1H), 4.95 (d, *J* = 10.6 Hz, 0.5H), 4.94 (d, *J* = 10.5 Hz, 1H), 4.93–4.87 (m, 1H), 4.81–4.74 (m, 0.5H), 4.67 (d, *J* = 6.7 Hz, 1H), 4.65 (d, *J* = 6.7 Hz, 0.5H), 4.61–4.55 (m, 2.5H), 3.91–3.82 (m, 2H), 3.71–3.66 (m, 1.5H), 3.35 (s, 3H), 3.33 (s, 1.5H), 3.14 (s, 3H), 3.07 (s, 3H), 2.40–2.27 (m, 3H), 2.00–1.85 (m, 3H), 1.76 (s, 4.5H), 1.76–1.68 (m, 1.5H), 1.34 (d, *J* = 6.4 Hz, 1.5H), 1.32 (d, *J* = 6.4 Hz, 3H), 1.18–1.12 (m, 4.5H), 0.99 (d, *J* = 7.4 Hz, 1.5H), 0.97 (d, *J* = 7.1 Hz, 3H); ^13^C NMR (150 MHz, 293 K, CDCl_3_) δ 167.9, 167.8, 143.3, 143.2, 141.54, 141.50, 135.5, 135.4, 129.2, 129.1, 122.9, 122.7, 111.1, 111.0, 95.0, 94.9, 79.4, 79.1, 73.6, 73.4, 70.6, 70.0, 55.4, 55.3, 50.3, 33.8, 33.2, 32.4, 32.1, 31.2, 30.9, 30.6, 29.8, 29.5, 28.7, 28.6, 22.8, 21.2, 21.1, 17.5, 17.3, 15.9, 15.8, 14.3, 12.1; HRMS (ESI^+^) calcd for C_21_H_36_NO_4_ [M + H]^+^ 366.2639, found 366.2623. Due to the frequent overlap of unresolved rotamer peaks, multiplicities and coupling constants for this molecule are not calculated for every signal. The signals for both rotamers are reported.

##### *N*-Methyl Meayamycin D

A 10 mL round-bottom flask with (*S*,*Z*)-*N*-((2*R*,3*R*,5*S*,6*S*)-2,5-dimethyl-6-((*E*)-3-methylpenta-2,4-dien-1-yl)tetrahydro-2*H*-pyran-3-yl)-4-(methoxymethoxy)-*N*-methylpent-2-enamide **19** (100 mg, 0.275 mmol) open to air was charged with DCE (500 μL), nitro-Grela catalyst (9 mg, 14 μmol, 5 mol %), and (3*R*,4*R*,5*R*)-7,7-dimethyl-5-vinyl-1,6-dioxaspiro[2.5]octan-4-ol **24** in DCE (200 μL, 100 mg/mL solution). After addition, the reaction was heated to 60 °C. After 3 h, right-hand fragment **24** in DCE (150 μL, 100 mg/mL solution) was added. After another 3 h, right-hand fragment **24** in DCE (150 μL, 100 mg/mL solution) was added. After another 3 h, right-hand fragment **24** in DCE (250 μL, 100 mg/mL solution) was added. After a total of 24 h at 60 °C, nitro-Grela catalyst (15 mg) was added. After an additional 24 h, the reaction mixture was removed from heat and concentrated. The crude material was dissolved in DCM (50 mL), and charcoal (7 g, 50× by weight) was added. The mixture was stirred for 6 h at 23 °C, filtered, and concentrated under reduced pressure. The crude material was purified by flash chromatography (40–80% EtOAc in hexanes) on silica gel (15 mL), followed by further HPLC purification, to afford *N*-methyl meayamycin D (2.6 mg*, 2% yield) as a colorless oil. *R*_f_ = 0.25 (60% EtOAc/hexanes); IR (neat): *v*_max_ = 3440, 2925, 2855, 1620 (C=O), 1524, 1454, 1262, 1093, 1035, 913, 800, 732 cm^–1^; ^1^H NMR (600 MHz, 293 K, CDCl_3_) δ 6.40 (d, *J* = 15.7 Hz, 1.5H), 6.13 (d, *J* = 11.7 Hz, 1H), 6.04 (d, *J* = 11.7 Hz, 0.5H), 5.88 (dd, *J* = 11.6, 8.5 Hz, 1H), 5.84 (dd, *J* = 11.6, 8.5 Hz, 0.5H), 5.69–5.62 (m, 1.5H), 5.61–5.55 (m, 1.5H), 4.93–4.87 (m, 1H), 4.81–4.75 (m, 0.5H), 4.67 (d, *J* = 6.7 Hz, 1H), 4.64 (d, *J* = 6.7 Hz, 0.5H), 4.59 (d, *J* = 6.5 Hz, 1.5H), 4.59–4.54 (m, 1H), 4.01 (dd, *J* = 9.5, 6.9 Hz, 1.5H), 3.89–3.81 (m, 2H), 3.70–3.61 (m, 1.5H), 3.54 (dd, *J* = 9.8, 9.8 Hz, 1.5H), 3.35 (s, 3H), 3.33 (s, 1.5H), 3.13 (s, 3H), 3.06 (s, 1.5H), 3.03 (d, *J* = 4.7 Hz, 1.5H), 2.50 (d, *J* = 4.7 Hz, 1.5H), 2.39–2.32 (m, 1.5H), 2.32–2.24 (m, 1.5H), 2.20 (d, *J* = 14.3 Hz, 1.5H), 1.98–1.86 (m, 3H), 1.77 (s, 4.5H), 1.74–1.66 (m, 1.5H), 1.60 (d, *J* = 10.0 Hz, 1H), 1.42 (d, *J* = 14.3 Hz, 1.5H), 1.40 (s, 4.5H), 1.34 (d, *J* = 6.4 Hz, 1.5H), 1.32 (d, *J* = 6.4 Hz, 3H), 1.29 (s, 4.5H), 1.18–1.11 (m, 4.5H), 0.98 (d, *J* = 7.3 Hz, 1.5H), 0.96 (d, *J* = 7.1 Hz, 3H); ^13^C NMR (175 MHz, 293 K, CDCl_3_) δ 167.9, 167.7, 143.3, 143.1, 138.5, 134.4, 130.0, 124.7, 122.9, 122.7, 95.0, 94.9, 79.3, 79.1, 74.9, 73.5, 73.0, 70.7, 70.0, 68.3, 57.6, 55.5, 50.3, 47.7, 43.0, 33.8, 32.3, 31.2, 30.9, 29.8, 28.6, 23.7, 22.8, 21.2, 21.1, 17.5, 17.3, 15.9, 15.8, 14.3, 12.8; HRMS (ESI^+^) calcd for C_29_H_48_NO_7_ [*M+H*]^+^ 522.3425, found 522.3411. Due to frequent overlap of unresolved rotamer peaks, multiplicities and coupling constants for this molecule are not calculated for every signal. The signals for both rotamers are reported. *Yield was calculated using Beer–Lambert law (*A* = ε × *c* × *l*) and the UV absorbance reading at 235 nm on a Shimadzu UV-1280 with molar absorptivity (ε) value as reported.^1^

#### *tert*-Butyl-((2*R*,3*R*,6*R*)-6-allyl-2-methyltetrahydro-2*H*-pyran-3-yl)carbamate (**11**)

The preparation of *tert*-butyl ((2*R*,3*R*,6*R*)-6-allyl-2-methyltetrahydro-2*H*-pyran-3-yl)carbamate **11** followed the reported procedure.^31^

#### (2*R*,3*R*,6*R*)-6-Allyl-*N*,2-dimethyltetrahydro-2*H*-pyran-3-amine (**20**)

A 100 mL round-bottom flask with a reflux condenser was purged with nitrogen gas three times, charged with THF (25 mL), and cooled to 0 °C. Lithium aluminum hydride (208 mg, 5.32 mmol, 3 equiv) was added at 0 °C. After 30 min, *tert*-butyl-((2*R*,3*R*,6*R*)-6-allyl-2-methyltetrahydro-2*H*-pyran-3-yl)carbamate **11** (453 mg, 1.77 mmol) was added dropwise in a solution of THF (10 mL) and the reaction was heated to reflux in an oil bath. After 17 h at reflux, the reaction mixture was removed from the oil bath, cooled to 0 °C, and quenched with dropwise addition of EtOAc (5 mL). Aqueous NaOH (1.0 M) was added dropwise at 0 °C until the precipitation of a white solid ceased. The reaction mixture was filtered through Celite and diluted with satd. aqueous sodium bicarbonate (10 mL). The aqueous layer was extracted with DCM (3 × 15 mL) using a separatory funnel. The combined organic layers were dried over anhydrous Na_2_SO_4_, filtered, and concentrated under reduced pressure to give (2*R*,3*R*,6*R*)-6-allyl-*N*,2-dimethyltetrahydro-2*H*-pyran-3-amine **20** (292 mg) as a gold oil which was used directly without further purification. For characterization, a small portion was dissolved in 1 N HCl and EtOAc (5 mL) was added to the solution. The layers were separated, and the aqueous layer was basified by the addition of solid NaHCO_3_. The resulting aqueous mixture was extracted with a mixture of 10% MeOH/DCM (2 × 5 mL), dried over anhydrous Na_2_SO_4_, filtered, and concentrated under reduced pressure to afford (2*R*,3*R*,6*R*)-6-allyl-*N*,2-dimethyltetrahydro-2*H*-pyran-3-amine **20**. *R*_f_ = 0.34 (10% MeOH/DCM); IR (neat): *v*_max_ = 3354, 2932, 2850, 1642, 1446, 1369, 1328, 1131, 1073, 911 cm^–1^; [α]_D_^25^ −23.6 (c 1.0, CH_2_Cl_2_); ^1^H NMR (400 MHz, 293 K, 1% CD_3_OD in CDCl_3_) δ 5.82 (dddd, *J* = 16.8, 9.2, 7.0, 7.0 Hz, 1H), 5.12–4.97 (m, 2H), 3.69–3.51 (m, 1H), 3.45–3.27 (m, 1H), 2.41 (s, 3H), 2.39 (s, 1H), 2.37–2.26 (m, 1H), 2.22–2.05 (m, 2H), 1.52–1.25 (m, 4H), 1.21 (d, *J* = 6.5 Hz, 3H); ^13^C NMR (100 MHz, 293 K, 1% CD_3_OD in CDCl_3_) δ 135.1, 116.7, 77.9, 76.6, 56.2, 41.0, 34.1, 26.4, 25.2, 18.5; HRMS (ESI^+^) calcd for C_10_H_20_NO [M + H]^+^ 170.1539, found 170.1541.

#### (*S*,*Z*)-*N*-((2*R*,3*R*,6*R*)-6-Allyl-2-methyltetrahydro-2*H*-pyran-3-yl)-4-(methoxymethoxy)-*N*-methylpent-2-enamide (**21**)

A 25 mL round-bottom flask with (2*R*,3*R*,6*R*)-6-allyl-*N*,2-dimethyltetrahydro-2*H*-pyran-3-amine **20** (247 mg, 1.35 mmol) was purged with nitrogen and then charged with MeCN (3 mL), (*S*,*Z*)-4-(methoxymethoxy)pent-2-enoic acid **4** (325 mg, 2.03 mmol, 1.5 equiv), and diisopropylethylamine (0.84 mL, 4.74 mmol, 3.5 equiv) at 23 °C. The resulting solution was cooled to 0 °C, and HATU (812 mg, 2.03 mmol, 1.5 equiv) was added at 0 °C, and the mixture was warmed to 23 °C. After 23 h, the reaction mixture was quenched with aqueous satd. NH_4_Cl (10 mL) and extracted with DCM (3 × 10 mL) using a separatory funnel. The combined organic layers were washed with brine (1 × 10 mL), dried over anhydrous Na_2_SO_4_, filtered, and concentrated under reduced pressure. The crude material was purified by flash chromatography (10–25% EtOAc in hexanes) on silica gel (35 mL) to afford (*S*,*Z*)-*N*-((2*R*,3*R*,6*R*)-6-allyl-2-methyltetrahydro-2*H*-pyran-3-yl)-4-(methoxymethoxy)-*N*-methylpent-2-enamide **21** (238 mg, 54% yield, 66:34 *s*-cis:*s*-trans amides) as a pale-yellow oil. The *s*-cis amide was defined by selective 1D NOESY of the N-Me signal (see Supporting Information, S84 and S85). *R*_f_ = 0.26 (40% EtOAc/hexanes); IR (film): *v*_max_ = 2977, 2932, 2853, 1627 (C=O), 1441, 1315, 1157, 1097, 1072, 1030 cm^–1^; [α]_D_^25^ −61.4 (c 1.0, CH_2_Cl_2_); ^1^H NMR (600 MHz, 293 K, 1% CD_3_OD in CDCl_3_) δ 6.16 (d, *J* = 11.7 Hz, 1H), 6.00 (d, *J* = 11.7 Hz, 0.5H), 5.86–5.78 (m, 3H), 5.11–5.03 (m, 3H), 4.84–4.77 (m, 1H), 4.74–4.68 (m, 0.5H), 4.68 (d, *J* = 6.6 Hz, 1H), 4.64 (d, *J* = 6.7 Hz, 0.5H), 4.58 (d, *J* = 6.7 Hz, 1H), 4.57 (d, *J* = 6.5 Hz, 0.5H), 4.54–4.50 (m, 1H), 3.76 (qd, *J* = 6.5, 3.2 Hz, 1H), 3.73 (qd, *J* = 6.5, 3.4 Hz, 0.5H), 3.66–3.62 (m, 0.5H), 3.51–3.43 (m, 1.5H), 3.34 (s, 3H), 3.33 (s, 1.5H), 3.24 (s, 3H), 3.20 (s, 1.5H), 2.39–2.31 (m, 1.5H), 2.28–2.19 (m, 1.5H), 2.04–1.90 (m, 3H), 1.78–1.53 (m, 5H), 1.33 (d, *J* = 6.4 Hz, 1.5H), 1.31 (d, *J* = 6.4 Hz, 3H), 1.17 (d, *J* = 6.5 Hz, 1.5H), 1.16 (d, *J* = 6.5 Hz, 3H); ^13^C NMR (150 MHz, 293 K, CDCl_3_) δ 168.1, 167.9, 142.7, 141.9, 134.6, 134.4, 117.3, 117.1, 95.0, 94.7, 77.50, 77.48, 75.6, 75.5, 70.7, 69.8, 55.45, 55.41, 53.2, 47.6, 40.99, 40.87, 35.3, 32.2, 29.4, 28.1, 27.1, 26.8, 21.2, 21.1, 18.2; HRMS (ESI^+^) calcd for C_17_H_30_NO_4_ [M + H]^+^ 312.2169, found 312.2165. Due to the frequent overlap of unresolved rotamer peaks, multiplicities and coupling constants for this molecule are not calculated for every signal. The signals for both rotamers are reported.

#### (*S*,*Z*)-4-(Methoxymethoxy)-*N*-methyl-*N*-((2*R*,3*R*,6*R*)-2-methyl-6-((*E*)-3-methyl-4-oxobut-2-en-1-yl)tetrahydro-2*H*-pyran-3-yl)pent-2-enamide (**22**)

A 5 mL sealed tube with (*S*,*Z*)-*N*-((2*R*,3*R*,6*R*)-6-allyl-2-methyltetrahydro-2*H*-pyran-3-yl)-4-(methoxymethoxy)-*N*-methylpent-2-enamide **21** (238 mg, 0.731 mmol) was placed under a flow of argon gas and then charged with methacrolein (1.89 mL, 18.3 mmol, 24 equiv) and nitro-Grela catalyst (26 mg, 45 μmol, 5 mol %). The sealed tube was capped and heated in an oil bath until the external temperature reached 50 °C. After 20 h at 50 °C, the reaction mixture was cooled to 23 °C. The crude contents were transferred to a separate 10 mL pear-shaped flask, concentrated under reduced pressure, and the resulting material was purified by flash chromatography (20–70% EtOAc in hexanes) on silica gel (35 mL) to afford (*S*,*Z*)-4-(methoxymethoxy)-*N*-methyl-*N*-((2*R*,3*R*,6*R*)-2-methyl-6-((*E*)-3-methyl-4-oxobut-2-en-1-yl)tetrahydro-2*H*-pyran-3-yl)pent-2-enamide **22** (148 mg, 55% yield, 95:5 E:Z, 69:31 *s*-cis:*s*-trans amides) as a light-brown oil. The *s*-cis amide was defined by selective 1D NOESY of (*S*,*Z*)-*N*-((2*R*,3*R*,6*R*)-6-allyl-2-methyltetrahydro-2*H*-pyran-3-yl)-4-(methoxymethoxy)-*N*-methylpent-2-enamide **21**. *R*_f_ = 0.14 (60% EtOAc/hexanes); IR (neat): *v*_max_ = 2976, 2933, 2888, 2855, 1684 (C=O), 1625 (C=O), 1442, 1317, 1157, 1097, 1074, 1030 cm^–1^; [α]_D_^25^ −78.2 (c 1.0, CH_2_Cl_2_); ^1^H NMR (600 MHz, 293 K, 1% CD_3_OD in CDCl_3_) δ 9.43 (s, 0.5H), 9.42 (s, 1H), 6.62–6.57 (m, 1.5H), 6.16 (dd, *J* = 11.6, 0.8 Hz, 1H), 6.00 (d, *J* = 11.6 Hz, 0.5H), 5.84 (dd, *J* = 11.6, 8.6 Hz, 1H), 5.83 (dd, *J* = 11.6, 8.6 Hz, 0.5H), 4.83–4.77 (m, 1H), 4.74–4.68 (m, 0.5H), 4.68 (d, *J* = 6.7 Hz, 1H), 4.65 (d, *J* = 6.7 Hz, 0.5H), 4.58 (d, *J* = 6.7 Hz, 1.5H), 4.57–4.53 (m, 1H), 3.80 (qd, *J* = 6.5, 3.4 Hz, 1H), 3.77 (qd, *J* = 6.5, 3.3 Hz, 0.5H), 3.70–3.66 (m, 0.5H), 3.64–3.56 (m, 1.5H), 3.34 (s, 3H), 3.33 (s, 1.5H), 3.24 (s, 3H), 3.21 (s, 1.5H), 2.60–2.54 (m, 3H), 2.07–1.94 (m, 3H), 1.77–1.74 (m, 4H), 1.68–1.56 (m, 3H), 1.34 (d, *J* = 6.4 Hz, 1.5H), 1.31 (d, *J* = 6.4 Hz, 3H), 1.19 (d, *J* = 6.5 Hz, 1.5H), 1.18 (d, *J* = 6.5 Hz, 3H); ^13^C NMR (150 MHz, 293 K, CDCl_3_) δ 195.2, 195.1, 168.2, 167.9, 150.1, 149.8, 143.0, 142.1, 141.00, 140.94, 123.5, 122.9, 95.0, 94.7, 76.72, 76.70, 75.9, 75.8, 70.7, 69.8, 55.5, 55.4, 53.0, 47.4, 36.0, 35.9, 35.2, 32.2, 31.7, 29.3, 28.0, 27.7, 27.5, 21.2, 21.1, 18.1, 9.6; HRMS (ESI^+^) calcd for C_29_H_32_NO_5_ [M + H]^+^ 354.2275, found 354.2283. Due to the frequent overlap of unresolved rotamer peaks, multiplicities and coupling constants for this molecule are not calculated for every signal. The signals for both rotamers are reported.

#### (*S*,*Z*)-4-(Methoxymethoxy)-*N*-methyl-*N*-((2*R*,3*R*,6*R*)-2-methyl-6-((*E*)-3-methylpenta-2,4-dien-1-yl)tetrahydro-2*H*-pyran-3-yl)pent-2-enamide (**23**)

A 25 mL round-bottom flask with methyltriphenylphosphonium bromide (411 mg, 1.13 mmol, 3.5 equiv) was purged with nitrogen gas three times, charged with THF (3.7 mL), and cooled to 0 °C. Potassium *tert*-butoxide (118 mg, 1.03 mmol, 3.2 equiv) was added at 0 °C. After 30 min at 0 °C, (*S*,*Z*)-4-(methoxymethoxy)-*N*-methyl-*N*-((2*R*,3*R*,6*R*)-2-methyl-6-((*E*)-3-methyl-4-oxobut-2-en-1-yl)tetrahydro-2*H*-pyran-3-yl)pent-2-enamide **22** (114 mg, 0.322 mmol) in THF (1.5 mL) was added, and the mixture was warmed to 23 °C. After 1.5 h at 23 °C, the reaction was quenched with aqueous satd. NH_4_Cl (5 mL). The mixture was concentrated under reduced pressure to remove residual THF. EtOAc (5 mL) was added to the crude mixture, and the layers were separated. The aqueous layer was extracted with EtOAc (2 × 5 mL) using a separatory funnel. The combined organic layers were washed with brine (1 × 5 mL), dried over anhydrous Na_2_SO_4_, filtered, and concentrated under reduced pressure. The crude material was purified by flash chromatography (5–30% EtOAc in hexanes) on silica gel (20 mL) to afford (*S*,*Z*)-4-(methoxymethoxy)-*N*-methyl-*N*-((2*R*,3*R*,6*R*)-2-methyl-6-((*E*)-3-methylpenta-2,4-dien-1-yl)tetrahydro-2*H*-pyran-3-yl)pent-2-enamide **23** (98 mg, 88% yield, 67:33 *s*-cis:*s*-trans amides) as a brown oil. The *s*-cis amide was defined by selective 1D NOESY of (*S*,*Z*)-*N*-((2*R*,3*R*,6*R*)-6-allyl-2-methyltetrahydro-2*H*-pyran-3-yl)-4-(methoxymethoxy)-*N*-methylpent-2-enamide **21**. *R*_f_ = 0.23 (40% EtOAc/hexanes); IR (neat): *v*_max_ = 2977, 2932, 2894, 2855, 1628 (C=O), 1442, 1315, 1157, 1098, 1073, 1032 cm^–1^; [α]_D_^25^ −68.2 (c 1.0, CH_2_Cl_2_); ^1^H NMR (600 MHz, 293 K, 1% CD_3_OD in CDCl_3_) δ 6.38 (dd, *J* = 17.3, 10.6 Hz, 0.5H), 6.37 (dd, *J* = 17.4, 10.7 Hz, 1H), 6.16 (d, *J* = 11.6 Hz, 1H), 6.00 (d, *J* = 11.7 Hz, 0.5H), 5.86–5.78 (m, 1.5H), 5.53 (t, *J* = 7.3 Hz, 1.5H), 5.11 (d, *J* = 17.3 Hz, 0.5H), 5.10 (d, *J* = 17.3 Hz, 1H), 4.96 (d, *J* = 10.6 Hz, 0.5H), 4.95 (d, *J* = 10.7 Hz, 1H), 4.84–4.77 (m, 1H), 4.74–4.68 (m, 0.5H), 4.68 (d, *J* = 6.7 Hz, 1H), 4.65 (d, *J* = 6.6 Hz, 0.5H), 4.58 (d, *J* = 6.6 Hz, 1.5H), 4.54–4.50 (m, 1H), 3.77 (qd, *J* = 6.5, 3.4 Hz, 1H), 3.74 (qd, *J* = 6.5, 3.4 Hz, 0.5H), 3.67–3.62 (m, 0.5H), 3.34 (s, 3H), 3.33 (s, 1.5H), 3.24 (s, 3H), 3.21 (s, 3H), 2.48–2.39 (m, 1.5H), 2.39–2.30 (m, 1.5H), 2.03–1.90 (m, 3H), 1.74 (s, 4H), 1.66–1.51 (m, 5H), 1.34 (d, *J* = 6.4 Hz, 0.5H), 1.31 (d, *J* = 6.4 Hz, 1H), 1.32 (d, *J* = 6.4 Hz, 3H), 1.18 (d, *J* = 6.4 Hz, 0.5H), 1.17 (d, *J* = 6.5 Hz, 1H); ^13^C NMR (150 MHz, 293 K, CDCl_3_) δ 168.1, 167.9, 142.7, 141.9, 141.43, 141.39, 136.1, 136.0, 128.2, 128.0, 123.6, 123.1, 111.4, 111.3, 95.0, 94.7, 77.8, 75.6, 75.5, 70.8, 69.8, 55.5, 55.4, 53.2, 47.6, 35.4, 35.3, 35.2, 32.2, 29.4, 28.1, 27.3, 27.1, 21.2, 21.1, 18.2, 12.1; HRMS (ESI+) calcd for C_20_H_34_NO_4_ [M + H]^+^ 352.2482, found 352.2490. Due to the frequent overlap of unresolved rotamer peaks, multiplicities and coupling constants for this molecule are not calculated for every signal. The signals for both rotamers are reported.

##### *N*-Methyl Meayamycin E

A 2 mL sealed tube was treated with (*S*,*Z*)-4-(methoxymethoxy)-*N*-methyl-*N*-((2*R*,3*R*,6*R*)-2-methyl-6-((*E*)-3-methylpenta-2,4-dien-1-yl)tetrahydro-2*H*-pyran-3-yl)pent-2-enamide **23** (75 mg, 21 μmol) in DCE (400 μL). (3*R*,4*R*,5*R*)-7,7-Dimethyl-5-vinyl-1,6-dioxaspiro[2.5]octan-4-ol **24** in DCE (196 μL, 100 mg/mL solution, 0.5 equiv), *p*-benzoquinone (6.9 mg, 64 μmol, 30 mol %), and nitro-Grela catalyst (9.6 mg, 14 μmol, 6.7 mol %) were added to the sealed tube at 23 °C. The sealed tube was purged with argon and heated to 50 °C. After 2 h at 50 °C, additional right-hand fragment **24** in DCE (196 μL, 100 mg/mL solution, 0.5 equiv) and nitro-Grela catalyst (9.6 mg, 14 μmol, 6.7 mol %) were added. After an additional 2 h at 50 °C, right-hand fragment **24** in DCE (196 μL, 100 mg/mL, 0.5 equiv) and nitro-Grela catalyst (9.6 mg, 14 μmol, 6.7 mol %) were added. After an additional 4 h at 50 °C, the reaction was cooled to 23 °C and filtered through a plug of silica (80% EtOAc in hexanes). Charcoal (1.6 g, 10× by weight) was added and the crude solution was stirred at 50 °C. After 3 h at 50 °C, the mixture was filtered, concentrated under reduced pressure, and purified by flash chromatography (20–80% EtOAc in hexanes) on silica gel (20 mL) to afford a complex mixture, which was further purified by preparative TLC (3:5:12 *i*-PrOH:EtOAc:hexanes). Unreacted (*S*,*Z*)-4-(methoxymethoxy)-*N*-methyl-*N*-((2*R*,3*R*,6*R*)-2-methyl-6-((*E*)-3-methylpenta-2,4-dien-1-yl)tetrahydro-2*H*-pyran-3-yl)pent-2-enamide **23** and right-hand fragment **24** were resubmitted to the reaction conditions and purified by preparative TLC (3:5:12 *i*-PrOH:EtOAc:hexanes). The product mixtures were combined and further purified by HPLC to afford *N*-methyl meayamycin E (4 mg*, 4% yield) as a colorless oil. *R*_f_ = 0.13 (60% EtOAc/hexanes); IR (neat): *v*_max_ = 3406, 2978, 2925, 2850, 1623 (C=O), 1442, 1324, 1206, 1158, 1098, 1031 cm^–1^; ^1^H NMR (600 MHz, 293 K, CD_2_Cl_2_) δ 6.36 (d, *J* = 15.6 Hz, 1.5H), 6.14 (dd, *J* = 11.6, 0.8 Hz, 1H), 6.00 (d, *J* = 11.6 Hz, 0.5H), 5.79 (dd, *J* = 11.8, 8.6 Hz, 1H), 5.76 (dd, *J* = 11.6, 8.5 Hz, 0.5H), 5.69–5.63 (m, 1.5H), 5.59 (t, *J* = 7.5 Hz, 1.5H), 4.78–4.71 (m, 1H), 4.67–4.59 (m, 0.5H), 4.64 (d, *J* = 6.6 Hz, 1H), 4.61 (d, *J* = 6.6 Hz, 0.5H), 4.55 (d, *J* = 6.6 Hz, 1H), 4.54 (d, *J* = 6.6 Hz, 0.5H), 4.47–4.50 (m, 1H), 3.97 (dd, *J* = 9.5, 6.7 Hz, 1.5H), 3.76 (qd, *J* = 6.5, 3.2 Hz, 1H), 3.73 (qd, *J* = 6.5, 3.3 Hz, 0.5H), 3.67–3.61 (m, 0.5H), 3.52–3.42 (m, 1.5H), 3.48 (dd, *J* = 9.9, 9.9 Hz, 1.5H), 3.31 (s, 3H), 3.30 (s, 1.5H), 3.23 (s, 3H), 3.17 (s, 1.5H), 2.96 (d, *J* = 4.8 Hz, 1.5H), 2.46 (d, *J* = 4.7 Hz, 1.5H), 2.44–2.37 (m, 1.5H), 2.37–2.29 (m, 1.5H), 2.17 (d, *J* = 14.3 Hz, 1.5H), 2.04–1.95 (m, 1.5H), 1.95–1.87 (m, 1.5H), 1.77 (s, 4.5H), 1.65–1.55 (m, 4H), 1.40 (d, *J* = 14.3 Hz, 3H), 1.37 (s, 4.5H), 1.31–1.22 (m, 9H), 1.17–1.12 (m, 4.5H); ^13^C NMR (150 MHz, 293 K, CD_2_Cl_2_) δ 168.2, 168.0, 142.2, 141.6, 137.84, 137.79, 135.2, 129.3, 129.1, 126.0, 125.9, 124.2, 123.9, 95.1, 94.9, 78.1, 75.9, 75.8, 75.0, 73.1, 70.6, 69.9, 68.7, 57.8, 55.43, 55.41, 47.82, 47.76, 43.1, 35.8, 35.7, 35.4, 32.2, 31.2, 30.1, 29.6, 28.3, 27.7, 27.5, 23.8, 21.3, 21.1, 18.3, 18.2, 12.8; HRMS (ESI^+^) calcd for C_28_H_46_NO_7_ [M + H]^+^ 508.3269, found 508.3272. Due to frequent overlap of unresolved rotamer peaks, multiplicities and coupling constants for this molecule are not calculated for every signal. The signals for both rotamers are reported. *Yield was calculated using Beer–Lambert law (*A* = ε × *c* × *l*) and the UV absorbance reading at 235 nm on a Shimadzu UV-1280 with molar absorptivity (ε) value as reported.^1^

#### (2*R*,3*R*,6*R*)-6-Allyl-2-methyltetrahydro-2*H*-pyran-3-amine (**12**)

The preparation of (2*R*,3*R*,6*R*)-6-allyl-2-methyltetrahydro-2*H*-pyran-3-amine **12** followed the reported procedure.^31^

#### (*S*,*Z*)-*N*-((2*R*,3*R*,6*R*)-6-Allyl-2-methyltetrahydro-2*H*-pyran-3-yl)-4-(methoxymethoxy)pent-2-enamide (**13**)

A 25 mL round-bottom flask with (2*R*,3*R*,6*R*)-6-allyl-2-methyltetrahydro-2*H*-pyran-3-amine **12** (383 mg, 1.50 mmol) was purged with nitrogen and then charged with DCM (5 mL), (*S*,*Z*)-4-(methoxymethoxy)pent-2-enoic acid **4** (241 mg, 1.50 mmol, 1.0 equiv), and diisopropylethylamine (734 μL, 4.50 mmol, 3.0 equiv) at 23 °C. The resulting solution was cooled to 0 °C, and HATU (570 mg, 1.50 mmol, 1.0 equiv) was added at 0 °C. The solution was slowly warmed to 23 °C. After 20 h, the reaction mixture was quenched with aqueous satd. NH_4_Cl (5 mL) and extracted with DCM (3 × 10 mL) using a separatory funnel. The combined organic layers were dried over anhydrous Na_2_SO_4_, filtered, and concentrated under reduced pressure. The crude material was purified by flash chromatography (10–35% EtOAc in hexanes) on silica gel (60 mL) to afford (*S*,*Z*)-*N*-((2*R*,3*R*,6*R*)-6-allyl-2-methyltetrahydro-2*H*-pyran-3-yl)-4-(methoxymethoxy)pent-2-enamide **13** (342 mg, 77% yield) as a white solid. *R*_f_ = 0.39 (40% EtOAc/hexanes); IR (film): *v*_max_ = 3313, 2977, 2933, 2857, 1662 (C=O), 1630, 1525, 1443, 1156, 1098, 1077, 1032 cm^–1^; [α]_D_^25^ −85.5 (c 1.0, CH_2_Cl_2_); ^1^H NMR (400 MHz, 293 K, CDCl_3_) δ 6.10 (d, *J* = 8.7 Hz, 1H), 5.98 (dd, *J* = 11.7, 8.3 Hz, 1H), 5.83 (dddd, *J* = 17.2, 10.2, 7.2, 7.2 Hz, 1H), 5.79 (dd, *J* = 11.7, 1.1 Hz, 1H), 5.35–5.26 (m, 1H), 5.13–5.03 (m, 2H), 4.69 (d, *J* = 6.7 Hz, 1H), 4.65 (d, *J* = 6.7 Hz, 1H), 3.99–3.93 (m, 1H), 3.65 (qd, *J* = 6.4, 1.6 Hz, 1H), 3.44 (dddd, *J* = 11.3, 6.4, 6.4, 2.3 Hz, 1H), 3.38 (s, 3H), 2.38–2.28 (m, 1H), 2.23–2.13 (m, 1H), 2.02–1.93 (m, 1H), 1.86–1.79 (m, 1H), 1.60–1.51 (m, 1H), 1.38–1.25 (m, 1H), 1.33 (d, *J* = 6.4 Hz, 3H), 1.13 (d, *J* = 6.4 Hz, 3H); ^13^C NMR (100 MHz, 293 K, CDCl_3_) δ 165.4, 146.5, 134.6, 122.6, 117.1, 95.4, 78.0, 75.2, 70.1, 55.6, 47.0, 40.8, 29.0, 26.0, 21.2, 18.3; HRMS (ESI^+^) calcd for C_16_H_28_NO_4_ [M + H]^+^ 298.2018, found 298.2020.

#### (*S*,*Z*)-4-(Methoxymethoxy)-*N*-((2*R*,3*R*,6*R*)-2-methyl-6-((*E*)-3-methyl-4-oxobut-2-en-1-yl)tetrahydro-2*H*-pyran-3-yl)pent-2-enamide (**14**)

A 5 mL sealed tube with (*S*,*Z*)-*N*-((2*R*,3*R*,6*R*)-6-allyl-2-methyltetrahydro-2*H*-pyran-3-yl)-4-(methoxymethoxy)pent-2-enamide **13** (121 mg, 0.403 mmol) was placed under a flow of argon gas and then charged with methacrolein (1.0 mL, 9.71 mmol, 24 equiv) and nitro-Grela catalyst (14 mg, 0.020 mmol, 5 mol %). The sealed tube was capped and heated in an oil bath until the external temperature reached 50 °C. After 22 h at 50 °C, the reaction mixture was cooled to 23 °C. The crude contents were transferred to a separate 10 mL pear-shaped flask, concentrated under reduced pressure, and purified by flash chromatography (20-70% EtOAc in hexanes) on silica gel (20 mL) to afford (*S*,*Z*)-4-(methoxymethoxy)-*N*-((2*R*,3*R*,6*R*)-2-methyl-6-((*E*)-3-methyl-4-oxobut-2-en-1-yl)tetrahydro-2*H*-pyran-3-yl)pent-2-enamide **14** (80 mg, 58% yield, 95:5 E:Z) as a light-brown oil. *R*_f_ = 0.30 (60% EtOAc/hexanes); IR (film): *v*_max_ = 3322, 2977, 2932, 2888, 2858, 1668 (C=O), 1634 (C=O), 1525, 1156, 1097, 1072, 1031 cm^–1^; [α]_D_^25^ −58.3 (c 1.0, CH_2_Cl_2_); ^1^H NMR (400 MHz, 293 K, CDCl_3_) δ 9.43 (s, 1H), 6.60–6.53 (m, 1H), 6.10 (d, *J* = 8.5 Hz, 1H), 5.97 (dd, *J* = 11.6, 8.3 Hz, 1H), 5.79 (dd, *J* = 11.7, 1.0 Hz, 1H), 5.32–5.22 (m, 1H), 4.68 (d, *J* = 6.7 Hz, 1H), 4.62 (d, *J* = 6.8 Hz, 1H), 4.02–3.95 (m, 1H), 3.67 (qd, *J* = 6.5, 1.7 Hz, 1H), 3.56 (dddd, *J* = 11.6, 6.0, 6.0, 2.3 Hz, 1H), 3.37 (s, 3H), 2.59–2.45 (m, 2H), 2.05–1.95 (m, 1H), 1.82–1.70 (m, 1H), 1.75 (s, 3H), 1.62–1.53 (m, 1H), 1.46–1.36 (m, 1H), 1.33 (d, *J* = 6.4 Hz, 3H), 1.13 (d, *J* = 6.4 Hz, 3H); ^13^C NMR (150 MHz, 293 K, CDCl_3_) δ 195.1, 165.5, 150.0, 146.4, 140.8, 122.5, 95.3, 77.1, 75.4, 70.1, 55.5, 46.7, 35.8, 28.9, 26.5, 21.1, 18.1, 9.5; HRMS (ESI^+^) calcd for C_18_H_30_NO_5_ [M + H]^+^ 340.2119, found 340.2113.

#### (*S*,*Z*)-4-(Methoxymethoxy)-*N*-((2*R*,3*R*,6*R*)-2-methyl-6-((*E*)-3-methylpenta-2,4-dien-1-yl)tetrahydro-2*H*-pyran-3-yl)pent-2-enamide (**15**)

A 25 mL round-bottom flask with methyltriphenylphosphonium bromide (302 mg, 0.828 mmol, 3.5 equiv) was purged with nitrogen gas three times, charged with THF (2.8 mL), and cooled to 0 °C. Potassium *tert*-butoxide (81 mg, 0.71 mmol, 3.0 equiv) was added at 0 °C. After 30 min at 0 °C, (*S*,*Z*)-4-(methoxymethoxy)-*N*-((2*R*,3*R*,6*R*)-2-methyl-6-((*E*)-3-methyl-4-oxobut-2-en-1-yl)tetrahydro-2*H*-pyran-3-yl)pent-2-enamide **14** (80 mg, 0.24 mmol) in THF (0.5 mL) was added and rinsed with THF (1 mL) at 0 °C. After 1 h at 0 °C, the reaction mixture was quenched with aqueous satd. NH_4_Cl (5 mL). The mixture was concentrated under reduced pressure to remove residual THF. EtOAc (5 mL) was added to the crude mixture, and the layers were separated. The aqueous layer was extracted with EtOAc (2 × 5 mL) using a separatory funnel. The combined organic layers were washed with brine (1 × 10 mL), dried over anhydrous Na_2_SO_4_, filtered, and concentrated under reduced pressure. The crude material was purified by flash chromatography (10–40% EtOAc in hexanes) on silica gel (20 mL) to afford (*S*,*Z*)-4-(methoxymethoxy)-*N*-((2*R*,3*R*,6*R*)-2-methyl-6-((*E*)-3-methylpenta-2,4-dien-1-yl)tetrahydro-2*H*-pyran-3-yl)pent-2-enamide **15** (66 mg, 82% yield) as a brown oil. *R*_f_ = 0.40 (40% EtOAc/hexanes); IR (neat): *v*_max_ = 3313, 2977, 2932, 2857, 1663 (C=O), 1629, 1526, 1157, 1099, 1072, 1034 cm^–1^; [α]_D_^25^ −59.0 (c 1.0, CH_2_Cl_2_); ^1^H NMR (600 MHz, 293 K, CDCl_3_) δ 6.37 (dd, *J* = 17.3, 10.7 Hz, 1H), 6.14 (d, *J* = 8.8 Hz, 1H), 5.96 (dd, *J* = 11.6, 8.3 Hz, 1H), 5.78 (dd, *J* = 11.6, 1.0 Hz, 1H), 5.50 (t, *J* = 7.3 Hz, 1H), 5.33–5.26 (m, 1H), 5.11 (d, *J* = 17.3 Hz, 1H), 4.96 (d, *J* = 10.7 Hz, 1H), 4.68 (d, *J* = 6.7 Hz, 1H), 4.64 (d, *J* = 6.7 Hz, 1H), 3.98–3.92 (m, 1H), 3.64 (qd, *J* = 6.4, 1.5 Hz, 1H), 3.42 (dddd, *J* = 11.4, 6.6, 6.6, 2.2 Hz, 1H), 3.37 (s, 3H), 2.43–2.35 (m, 1H), 2.33–2.25 (m, 1H), 1.98–1.92 (m, 1H), 1.77–1.67 (m, 1H), 1.74 (s, 3H), 1.58–1.53 (m, 1H), 1.35–1.27 (m, 1H), 1.32 (d, *J* = 6.5 Hz, 3H), 1.12 (d, *J* = 6.4 Hz, 3H); ^13^C NMR (150 MHz, 293 K, CDCl_3_) δ 165.5, 146.4, 141.4, 136.0, 128.1, 122.6, 111.4, 95.3, 78.3, 75.2, 70.1, 55.6, 47.0, 35.3, 29.0, 26.2, 21.2, 18.3, 12.1; HRMS (ESI^+^) calcd for C_19_H_32_NO_4_ [M + H]^+^ 338.2326, found 338.2318.

##### Meayamycin E

A 2 mL sealed tube was treated with (*S*,*Z*)-4-(methoxymethoxy)-*N*-((2*R*,3*R*,6*R*)-2-methyl-6-((*E*)-3-methylpenta-2,4-dien-1-yl)tetrahydro-2*H*-pyran-3-yl)pent-2-enamide **15** (13 mg, 39 μmol) in DCE (210 μL). (3*R*,4*R*,5*R*)-7,7-Dimethyl-5-vinyl-1,6-dioxaspiro[2.5]octan-4-ol **24** in DCE (36 μL, 100 mg/mL solution, 0.5 equiv), *p*-benzoquinone (1.3 mg, 12 μmol, 30 mol %), and nitro-Grela catalyst (2.6 mg, 3.9 μmol, 10 mol %) were added to the sealed tube at 23 °C. The sealed tube was purged with argon and heated to 50 °C. After 2 h at 50 °C, additional right-hand fragment **24** in DCE (36 μL, 100 mg/mL solution, 0.5 equiv) was added. After an additional 2 h at 50 °C, right-hand fragment **24** in DCE (36 μL, 100 mg/mL, 0.5 equiv) and nitro-Grela catalyst (2.6 mg, 3.9 μmol, 10 mol %) were added. After an additional 4 h at 50 °C, the reaction mixture was cooled to 23 °C and filtered through a plug of silica (80% EtOAc in hexanes). The crude material was concentrated under reduced pressure and purified by flash chromatography (20–80% EtOAc in hexanes) on silica gel (1 mL) to afford a complex mixture, which was further purified by preparative TLC (60% EtOAc in hexanes). To remove excess ruthenium, the compound was further purified by HPLC to afford meayamycin E (0.58 mg*, 3% yield) as a white solid. *R*_f_ = 0.21 (60% EtOAc/hexanes); IR (neat): *v*_max_ = 3331, 2970, 2917, 2849, 1665 (C=O), 1630, 1525, 1448, 1371, 1223, 1156, 1099, 1033 cm^–1^; ^1^H NMR (600 MHz, 293 K, CD_2_Cl_2_) δ 6.35 (d, *J* = 15.7 Hz, 1H), 6.12 (d, *J* = 9 Hz, 1H), 5.92 (dd, *J* = 11.6, 8.3 Hz, 1H), 5.79 (dd, *J* = 11.6, 1.0 Hz, 1H), 5.64 (dd, *J* = 15.7, 6.7 Hz, 1H), 5.56 (t, *J* = 7.3 Hz, 1H), 5.30–5.27 (m, 1H), 4.63 (d, *J* = 6.7 Hz, 1H), 4.59 (d, *J* = 6.7 Hz, 1H), 3.96 (dd, *J* = 9.5, 7.1 Hz, 1H), 3.93–3.88 (m, 1H), 3.63 (qd, *J* = 6.2, 1.5 Hz, 1H), 3.48 (dd, *J* = 10.0, 10.0 Hz, 1H), 3.42 (dddd, *J* = 11.6, 6.5, 6.5, 2.3 Hz, 1H), 3.33 (s, 3H), 2.96 (d, *J* = 4.7 Hz, 1H), 2.46 (d, *J* = 4.7 Hz, 1H), 2.39–2.32 (m, 1H), 2.32–2.25 (m, 1H), 2.17 (d, *J* = 14.3 Hz, 1 H), 1.93–1.88 (m, 1H), 1.78 (s, 3H), 1.75–1.68 (m, 1H), 1.61 (d, *J* = 10.4 Hz, 1H), 1.54–1.50 (m, 1H), 1.40 (d, *J* = 14.3 Hz, 1H), 1.36 (s, 3H), 1.36–1.28 (m, 1H), 1.28–1.20 (m, 9H), 1.08 (d, *J* = 6.4 Hz, 3H); ^13^C NMR (150 MHz, 293 K, CD_2_Cl_2_) δ 165.5, 146.3, 137.8, 135.1, 129.3, 125.9, 123.0, 95.3, 78.5, 75.4, 74.9, 73.0, 69.8, 68.6, 57.8, 55.5, 47.8, 47.1, 43.1, 35.5, 31.3, 30.0, 29.3, 26.4, 23.7, 21.2, 18.3, 12.7; HRMS (ESI^+^) calcd for C_27_H_44_NO_7_ [M + H]^+^ 494.3112, found 494.3113. *Yield was calculated using Beer–Lambert law (*A* = ε × *c* × *l*) and the UV absorbance reading at 235 nm on a Shimadzu UV-1280 with molar absorptivity (ε) value as reported.^1^

### In Vitro Plasma Stability

Mouse CD1 plasma K2 EDTA (Innovative Research) was prepared in a 2 mL microcentrifuge tube. The compounds were prepared separately as 1 mM solutions in 10% DMSO and added to the plasma as 100× dilutions to give 700 μL at a concentration of compound, 10 μM procaine, meayamycin (meayamycin A), meayamycin D, or meayamycin E, in 0.1% DMSO. The mixture was vortexed for 10 s, capped, and placed in a shaking incubator (Corning) for 48 h at 125 rpm at 37 °C. At the indicated times an aliquot (70 μL) of the mixture was taken and added to an equal volume of ice-cold MeCN and centrifuged for 15 min at 14,000 relative centrifugal force (rcf) at 4 °C. The supernatant was collected and frozen at −80 °C until sample analysis. The samples were analyzed by LC-MS using Fmoc-l-phenylalanine as an internal standard at a concentration of 25 μM. A standard curve was prepared separately in a matrix-matched solution by 2-fold serial dilution from 10 μM. The decomposition was determined by comparing the ratio of analyte to Fmoc-l-phenylalanine with the ratio of the analyte to Fmoc-l-phenylalanine in the first data point.

### Growth Inhibition Assay

All cell lines were obtained from ATCC (Manassas, VA) and maintained in RPMI-1640 media, Waymouth media (DMS53 and DMS114 cells), or F-12K media (FL83B cells) + 10% fetal bovine serum. The cells were mycoplasma-free as determined by the e-Myco PLUS mycoplasma PCR detection kit (Bulldog Bio, Portsmouth, NH). The cells were plated in 96-well plates at an initial density of 1500 or 5000 cells per well in culture media (100 μL) and were incubated for 24 h prior to compound addition. The compounds were prepared separately as 10 mM in 100% DMSO or 10 μM in 100% DMSO. Serial dilution in sterile water gave 10× dilutions that were added directly to the cells as 100× dilutions to give the desired concentration of compound, 0.1 nM to 30 μM meayamycin A, meayamycin D, *N*-methyl meayamycin D, meayamycin E, or *N*-methyl meayamycin E, in 0.1–0.3% DMSO. The cells were then incubated for additional 72 h. Cell proliferation was measured by using the commercial 3-(4,5-dimethylthiazol-2-yl)-5-(3-carboxymethoxyphenyl)-2-(4-sulfophenyl)-2*H*-tetrazolium (MTS) dye or 4-[3-(4-iodophenyl)-2-(4-nitrophenyl)-2*H*-5-tetrazolio]-1,3-benzenesulfonate (WST-1) dye reduction assay. The absorbance at 490 nm (MTS) or 450 nm (WST-1) was measured with a Modulus II Microplate Multimode Reader (Promega) or Tecan Infinite M1000 PRO Multimode Reader Evaluation of the compounds was performed in duplicate at each concentration. GraphPad Prism 9.4.0 was used to construct dose–response curves and calculate the GI_50_ values.

### Immunoblot Analysis

Cells were treated with various concentrations of meayamycin analogues for 8 h and then lysed using RIPA buffer (10 mM Tris, pH 7.5, 150 mM NaCl, 1 mM EDTA, 0.1% SDS, 1% IGEPAL, 0.5% sodium deoxycholate) containing phosphatase and protease inhibitors. Approximately 20 μg protein from each cell lysate was resolved on SDS-PAGE gels (Cat#5671084; Bio-Rad). Proteins were transferred onto nitrocellulose membranes followed by 1 h incubation at room temperature in blocking solution (1× TBS, 0.1% Tween-20, 5% milk powder). Membranes were incubated overnight at 4 °C with the following antibodies: antiphospho-SF3B1(#25009; Cell Signaling), anti-SF3B1 (#14434; Cell Signaling), anti-MCL-1 (#5453; Cell Signaling), anti-p27 (#3686; Cell Signaling), anti-p21 (#2947; Cell Signaling), anti-p53 (sc-126; Santa Cruz Biotechnology; Dallas, TX), and anti-α-tubulin (#2125; Cell Signaling). Proteins were detected using SuperSignal West Pico substrate (Pierce; Rockford, IL).

### Combination Index Analysis

Cells were plated in 96-well plates at an initial density of 1500 or 5000 cells per well in RPMI-1640 medium (100 μL) and were incubated for 24 h prior to compound addition. The compounds were prepared separately as 10 mM in 100% DMSO or 10 μM in 100% DMSO. Serial dilution in sterile water gave 10× dilutions that were added directly to the cells alone or in combinations as 100× dilutions to give the desired concentration of compound: 0.5–3 nM meayamycin D, 1.25–10 μM S63845, 25 nM to 8 μM venetoclax, and 0.25–4 μM osimertinib. The cells were then incubated for additional 72 h. Cell proliferation was measured by using the commercial WST-1 dye reduction assay. To determine whether drug combinations were synergistic, we used CompuSyn software to calculate the combination index (CI) using nonconstant drug ratios.^48^ A CI of less than 1.0 was considered to be indicative of synergism, and this interaction was further classified as strong synergism (CI < 0.3), synergism (CI of 0.3–0.7), and slight to moderate synergism (CI of 0.7–0.9).

## Supporting Information Available

Molecular formula strings (CSV)
X-ray crystallographic data (CIF)
Selective 1D NOE spectrum of compounds **8**, **17**, and **21**; DFT calculations; ^1^H and ^13^C{^1^H} NMR spectrum of synthesized compounds; cytotoxicity assay data; RT-PCR; LC–MS traces; and X-ray structure of compound **8** (CCDC: 2294804) (PDF)

## Supplementary Material

jm3c00733_si_001.csv

jm3c00733_si_002.cif

## Abbreviations

BCL-2: B-cell lymphoma 2
CI: combination index
GI50: half-maximal growth inhibition
HATU: 1-[bis(dimethylamino)methylene]-1*H*-1,2,3-triazolo[4,5-*b*]pyridinium 3-oxid hexafluorophosphate
IGEPAL: octylphenoxy poly(ethyleneoxy) ethanol
MCL-1L: myeloid cell leukemia-1 long isoform
MCL-1S: myeloid cell leukemia-1 short isoform
sd: standard deviation
MTS: 3-(4,5-dimethylthiazol-2-yl)-5-(3-car-boxymethoxyphenyl)-2-(4-sulfophenyl)-2*H*-tetrazolium
PHF5A: PHD finger protein 5A
rcf: relative centrifugal force
SCLC: small-cell lung cancer
SF3B1: splicing factor 3B subunit 1
TBS: tris-buffered saline
WST-1: 4-[3-(4-iodophenyl)-2-(4-nitrophenyl)-2*H*-5-tetrazolio]-1,3-benzenesulfonate
