# Supplementary material for: Synthesis and Conformational Analysis of FR901464-Based RNA Splicing Modulators and Their Synergism in Drug-Resistant Cancers
Source: J Med Chem. 2023 Oct 23;66(21):14497–512. doi: 10.1021/acs.jmedchem.3c00733 (PMC10641826; doi:10.1021/acs.jmedchem.3c00733)
Supplement: Supplementary file 3 — jm3c00733_si_003.pdf [file jm3c00733_si_003.pdf]

Supporting Information

for

Synthesis and conformational analysis of FR901464-based RNA splicing modulators and their synergism in drug-resistant cancers

Jacob P. Beard[1], Robert K. Bressin[1], Paulo L. Markaj[1], John C. Schmitz[2,3]*, and Kazunori Koide[1]*

[1]Department of Chemistry, University of Pittsburgh

219 Parkman Avenue, Pittsburgh, Pennsylvania 15260, United States

[2]Division of Hematology-Oncology, Department of Medicine, University of Pittsburgh School of Medicine

5150 Centre Avenue, Pittsburgh, Pennsylvania 15232, United States

[3]Cancer Therapeutics Program, UPMC Hillman Cancer Center

5117 Centre Ave, Pittsburgh, Pennsylvania 15232, United States

schmitzjc@upmc.edu; koide@pitt.edu

Table of Contents

Figure S1–2. Selective 1D NOESY spectra of amide **17** rotamers S2

Figure S3. ^{1}H NMR spectra of meayamycin analogs S3

Figure S4–11.^{1}H NMR spectra of amide **8**, **13**, **17**, and **21** in CD_3OD S4

Figure S12. Cryogenic ^{1}H NMR of amide **8** S12

Figure S13–15. Selective 1D NOESY spectra of amide **8** S13

Figure S16. DFT structure of amide **8** ……………………………………………………..S15

Figure S17. Cell cytotoxicity assays S17

Figure S18. RT-PCR...S20

LCMS Traces S21

NMR spectra S27

Selective 1D NOESY spectra of amide rotamers

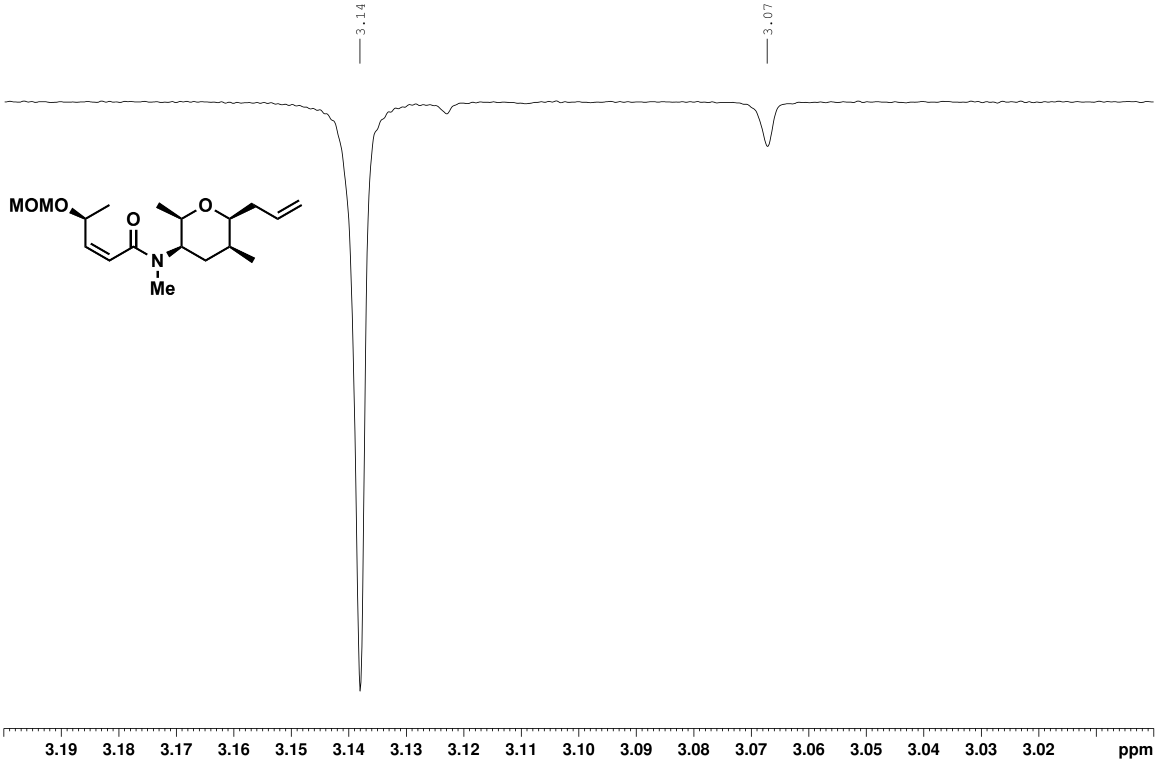

Figure S1. Selective 1D NOESY spectrum of amide **17** (600 MHz, $CDCl_3$, 293K, major rotamer)

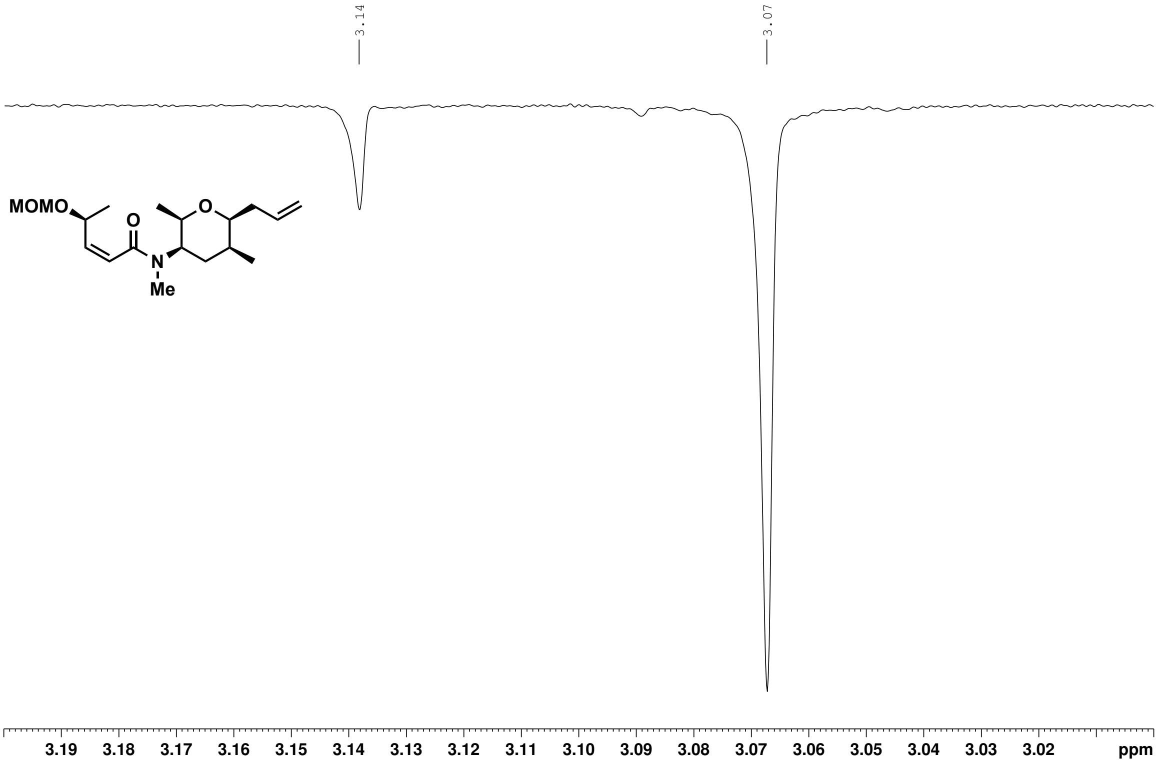

Figure S2. Selective 1D NOESY spectrum of amide **17** (600 MHz, $CDCl_3$, 293K, minor rotamer)

Stacked ^{1}H NMR of meayamycin analogs

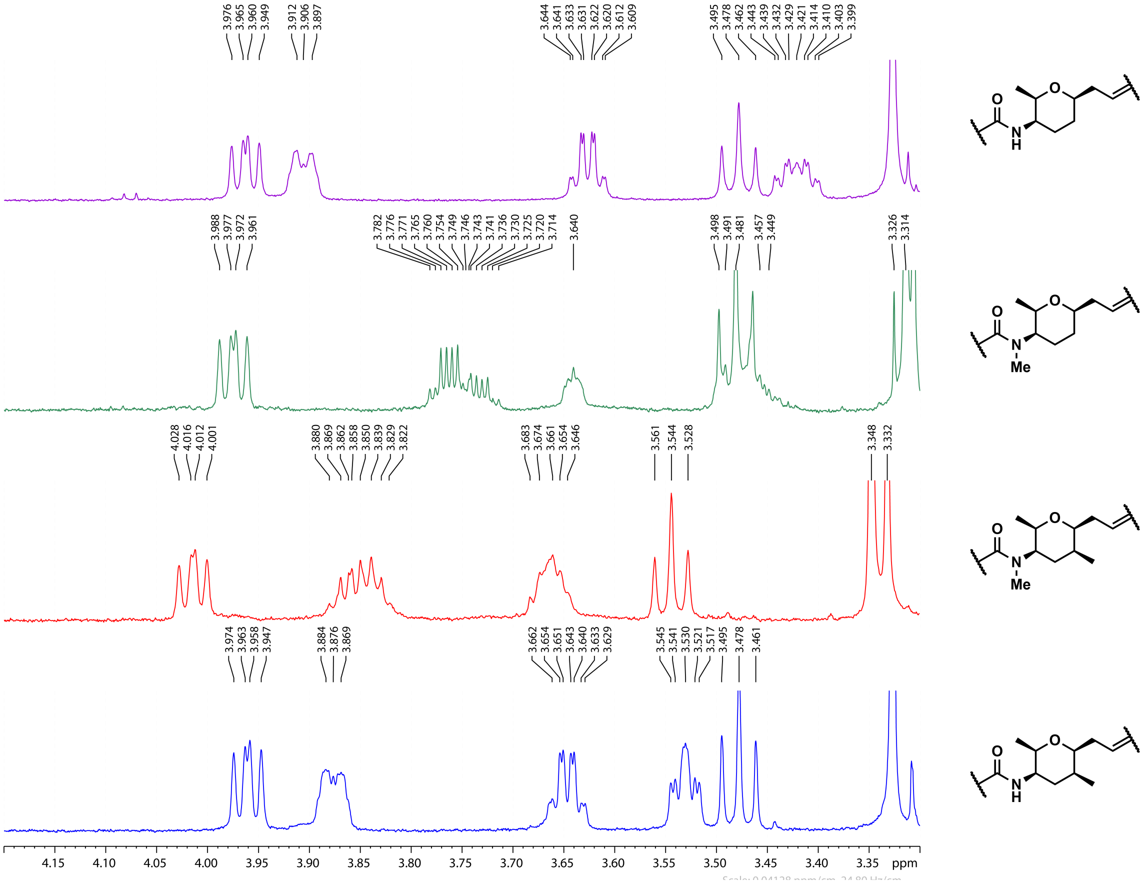

Figure S3. Expanded regions of ^{1}H NMR spectrum of (top to bottom) meayamycin E (600 MHz, CD_2Cl_2, 293K), N-methyl meayamycin E (600 MHz, CD_2Cl_2, 293K), N-methyl meayamycin D (600 MHz, $CDCl_3$, 293K), and meayamycin D (600 MHz, CD_2Cl_2, 293K)

^{1}H NMR spectra in CD_3OD for conformational analysis

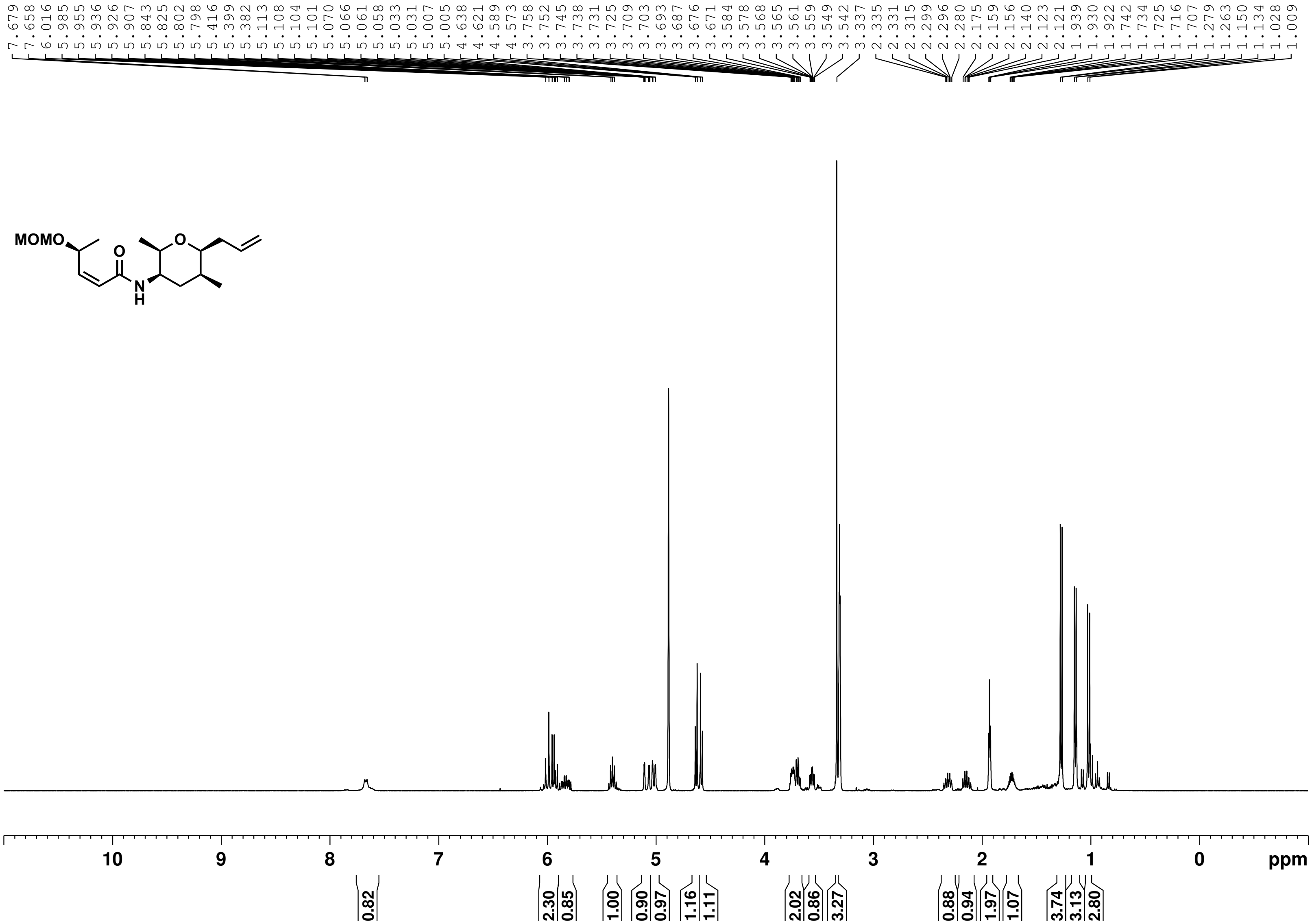

Figure S4. ^{1}H NMR spectrum of amide **8** (400 MHz, CD_3OD, 293K)

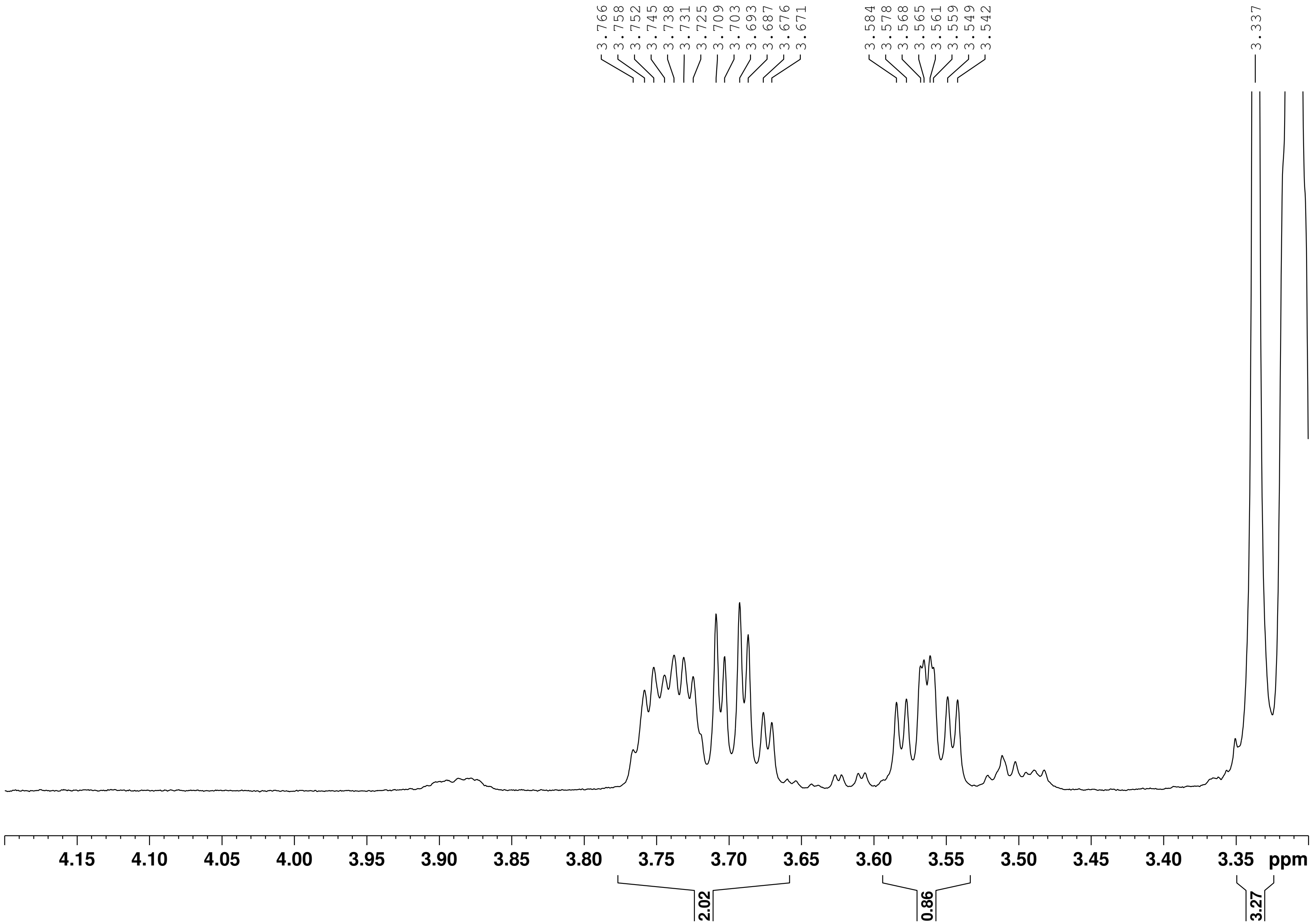

Figure S5. ^{1}H NMR expanded spectrum of amide **8** (400 MHz, CD_3OD, 293K)

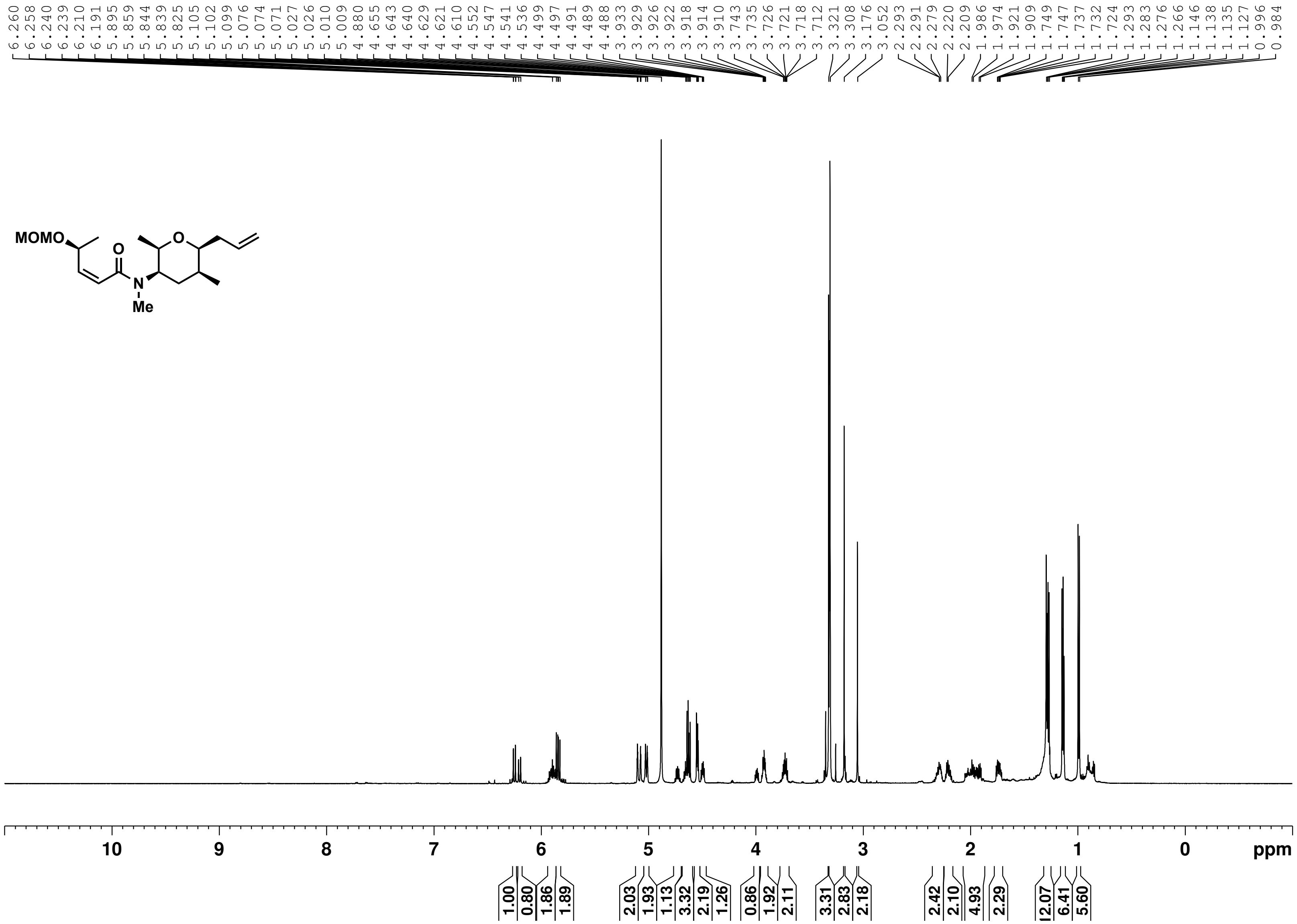

Figure S6. ^{1}H NMR spectrum of amide **17** (600 MHz, CD_3OD, 293K)

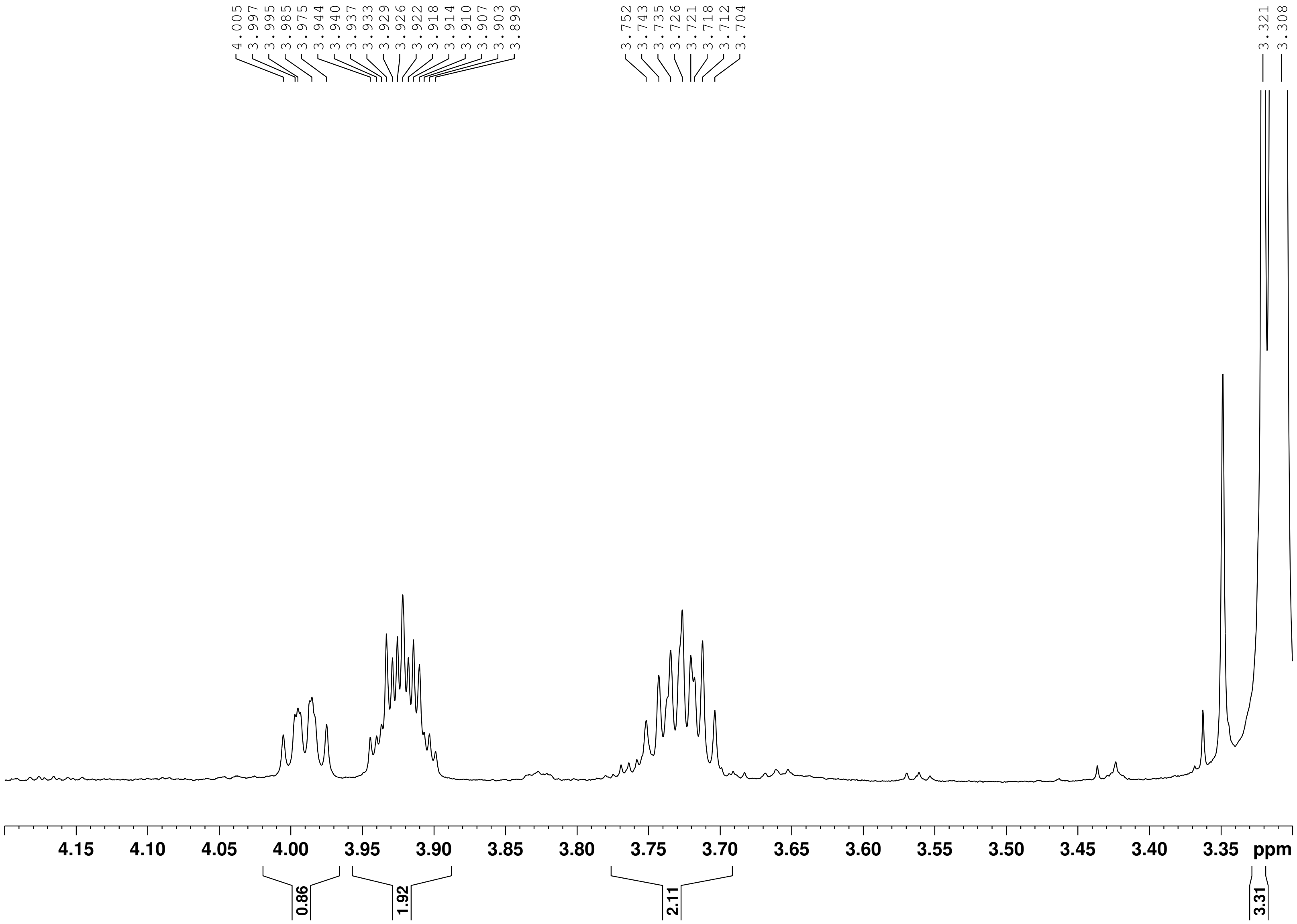

Figure S7. ^{1}H NMR expanded spectrum of amide **17** (600 MHz, CD_3OD, 293K)

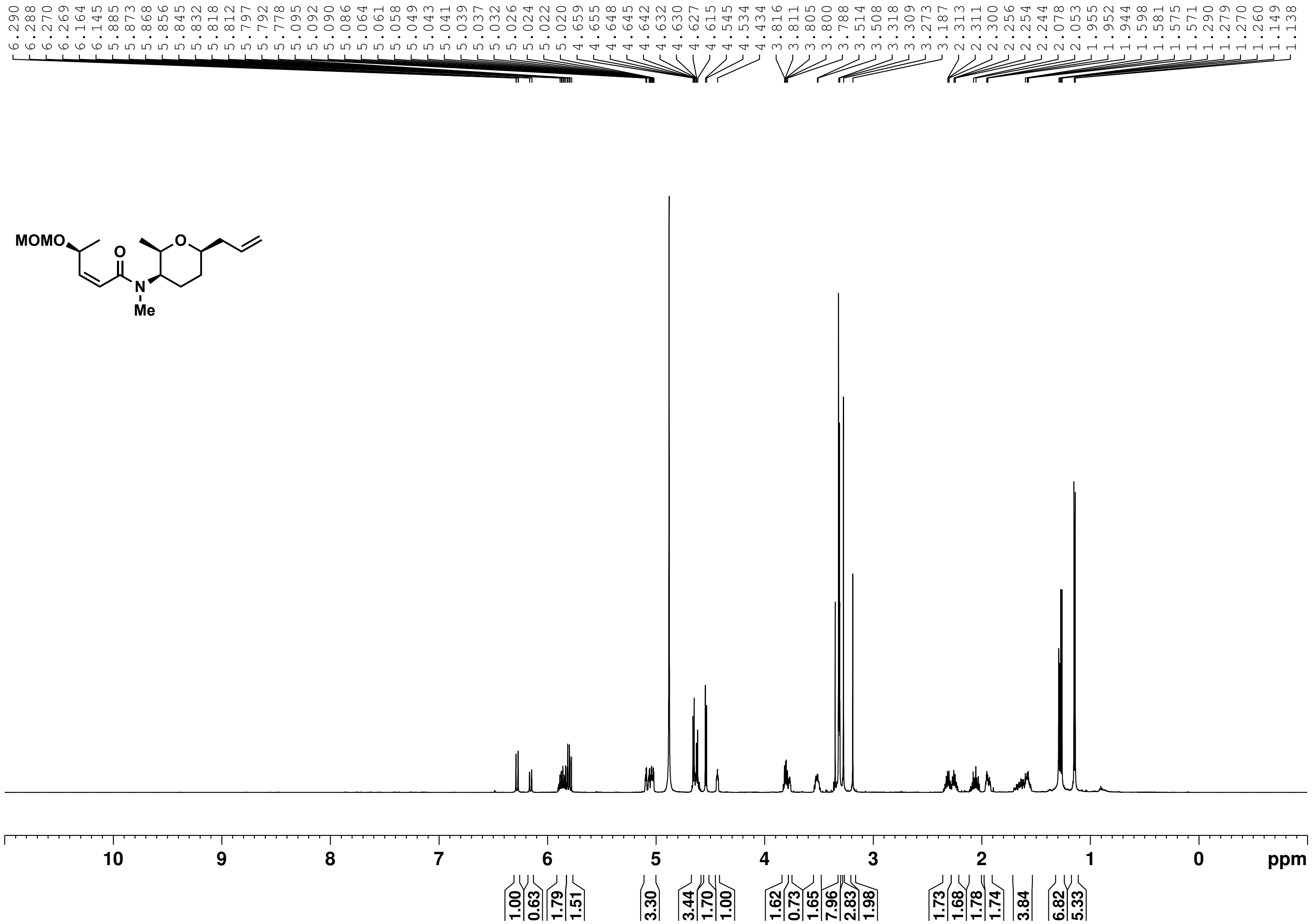

Figure S8. ^{1}H NMR spectrum of amide **21** (500 MHz, CD_3OD, 293K)

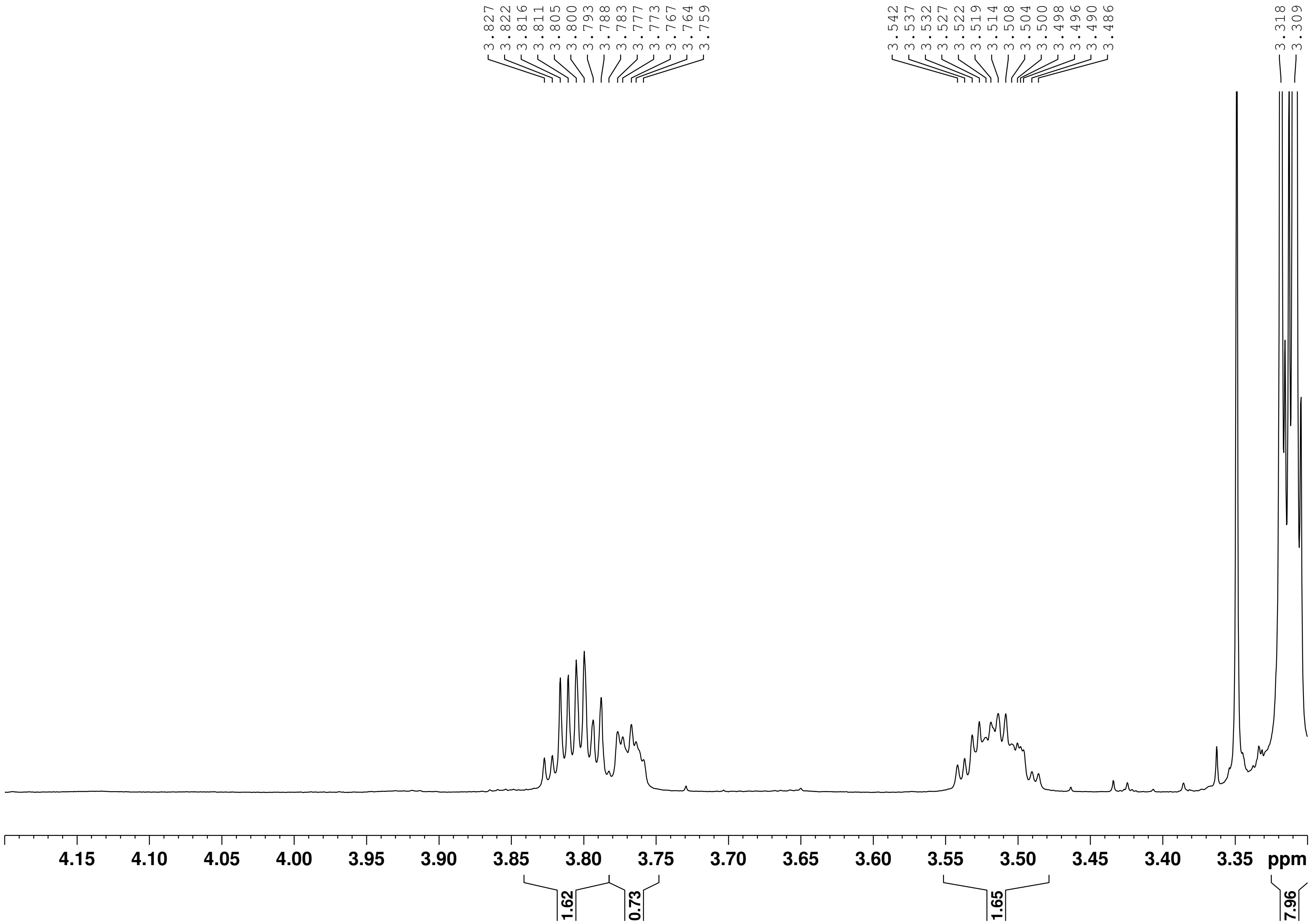

Figure S9. ^{1}H NMR expanded spectrum of amide **21** (500 MHz, CD_3OD, 293K)

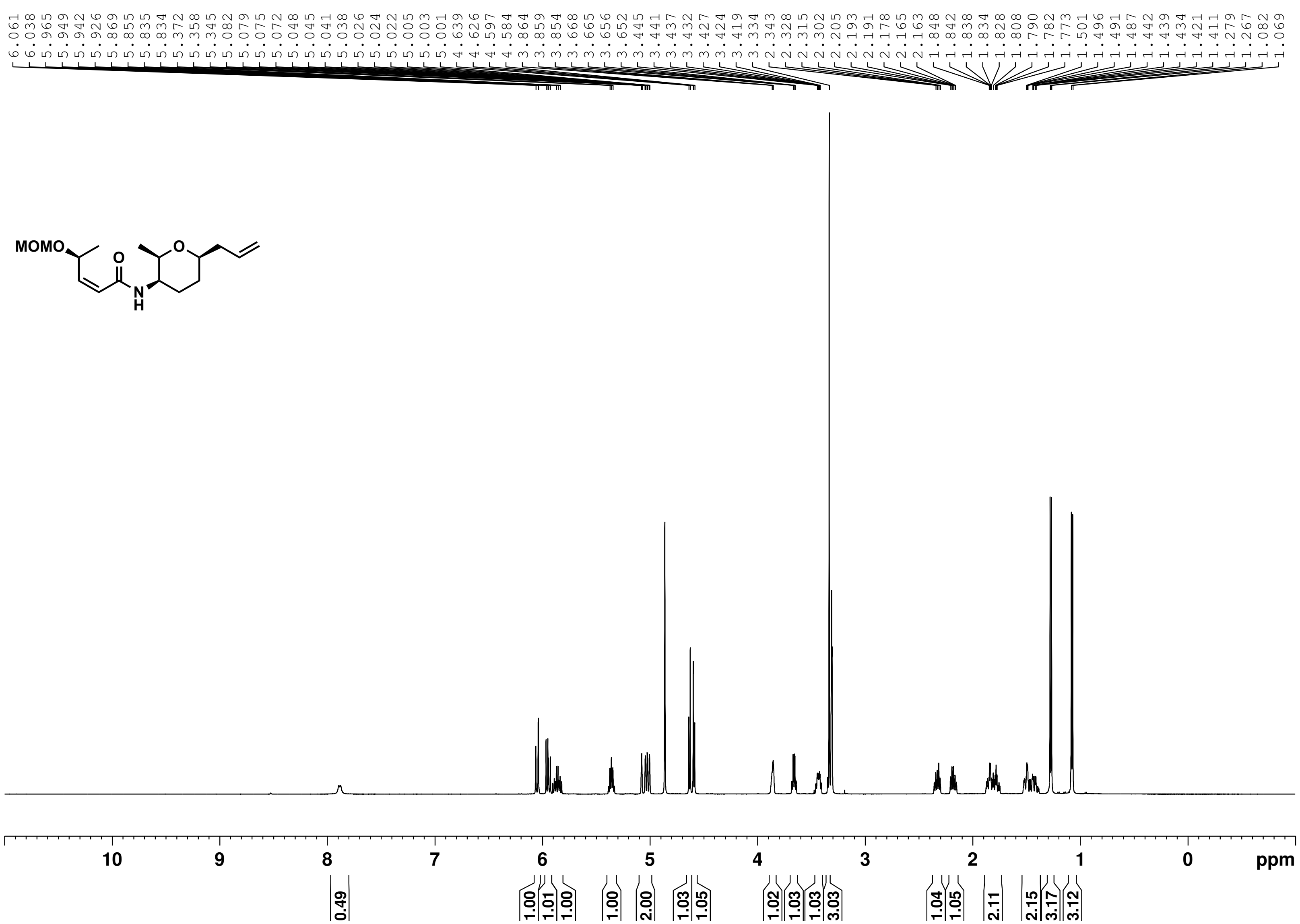

Figure S10. ^{1}H NMR spectrum of amide **13** (500 MHz, CD_3OD, 293K)

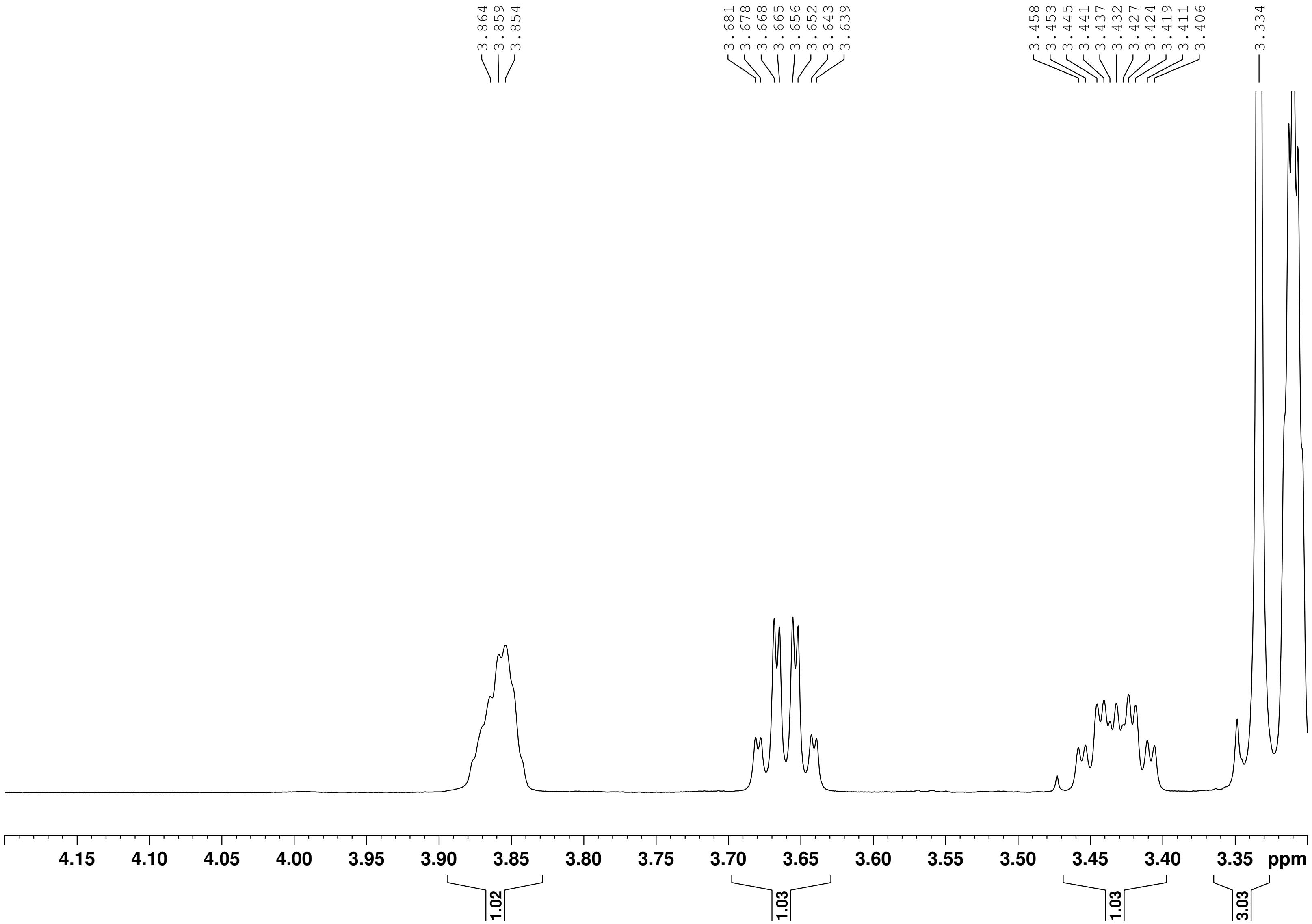

Figure S11. ^{1}H NMR expanded spectrum of amide **13** (500 MHz, CD_3OD, 293K)

Cryogenic ^{1}H NMR of amide 8

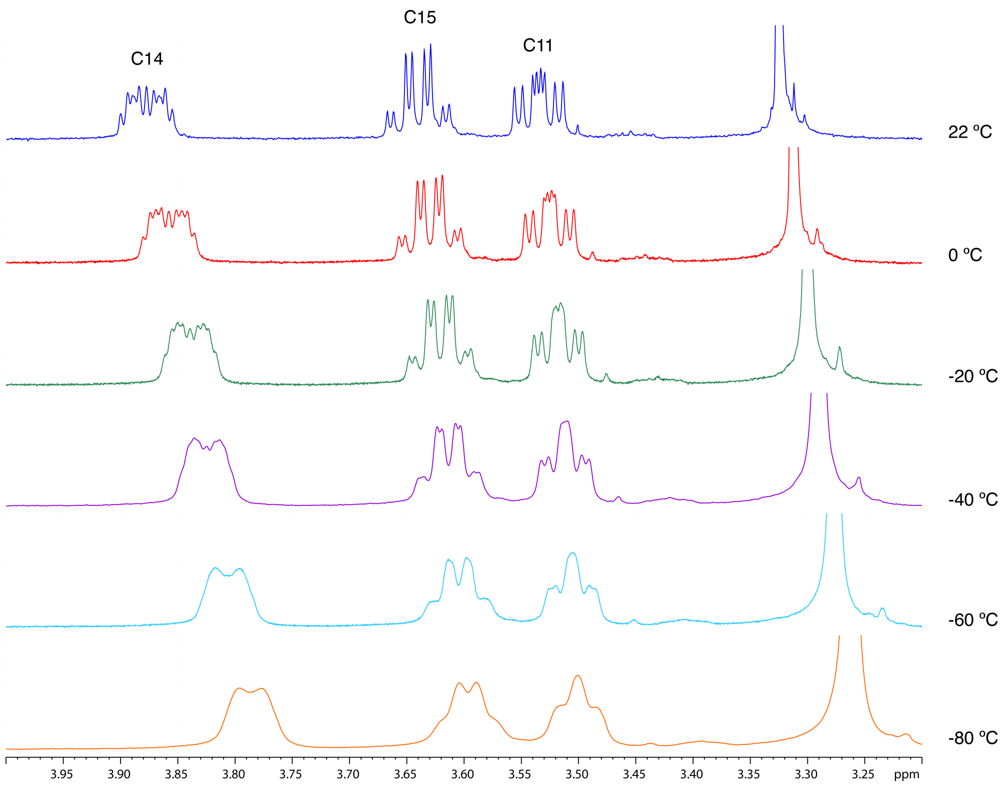

Figure S12. Cryogenic NMR analysis. Expanded regions of ^{1}H NMR spectrum of amide **8** (400 MHz, CD_2Cl_2 (20 mM), 22 to -80 °C)

Selective 1D NOESY spectra of amide 8

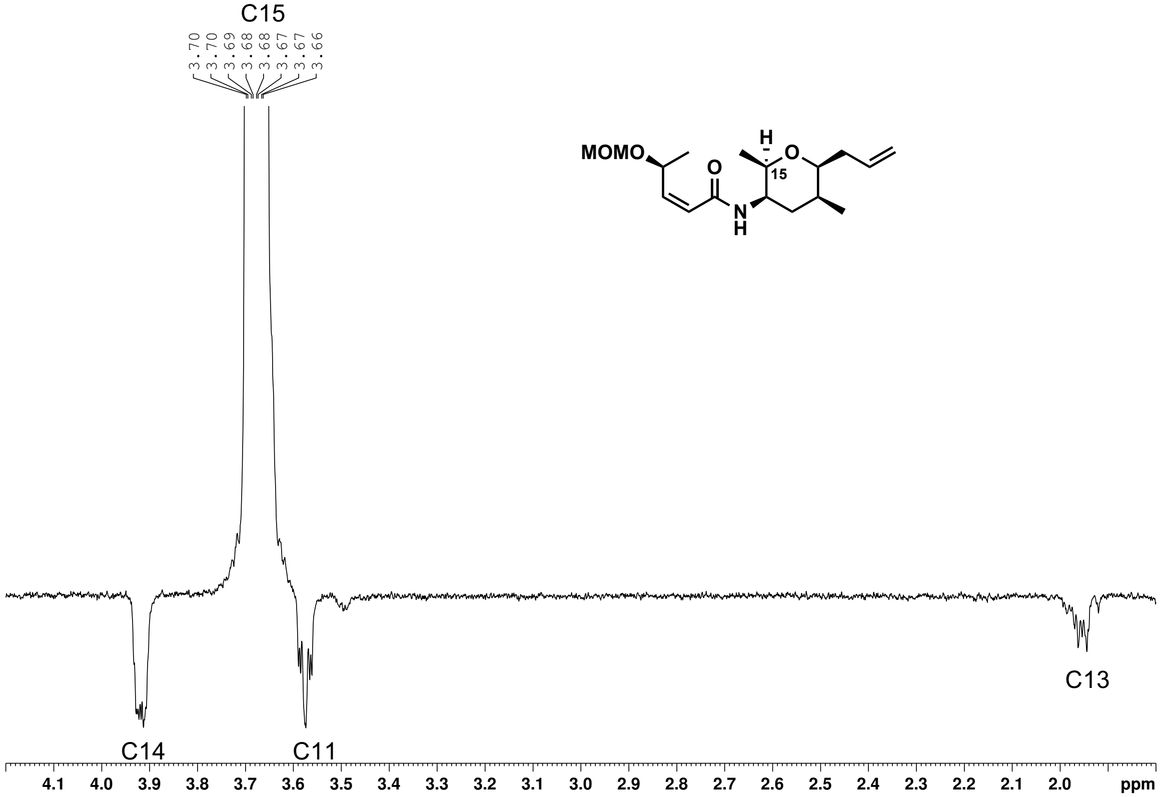

Figure S13. Selective 1D NOESY spectrum of amide **8** (600 MHz, CD_2Cl_2, 293K)

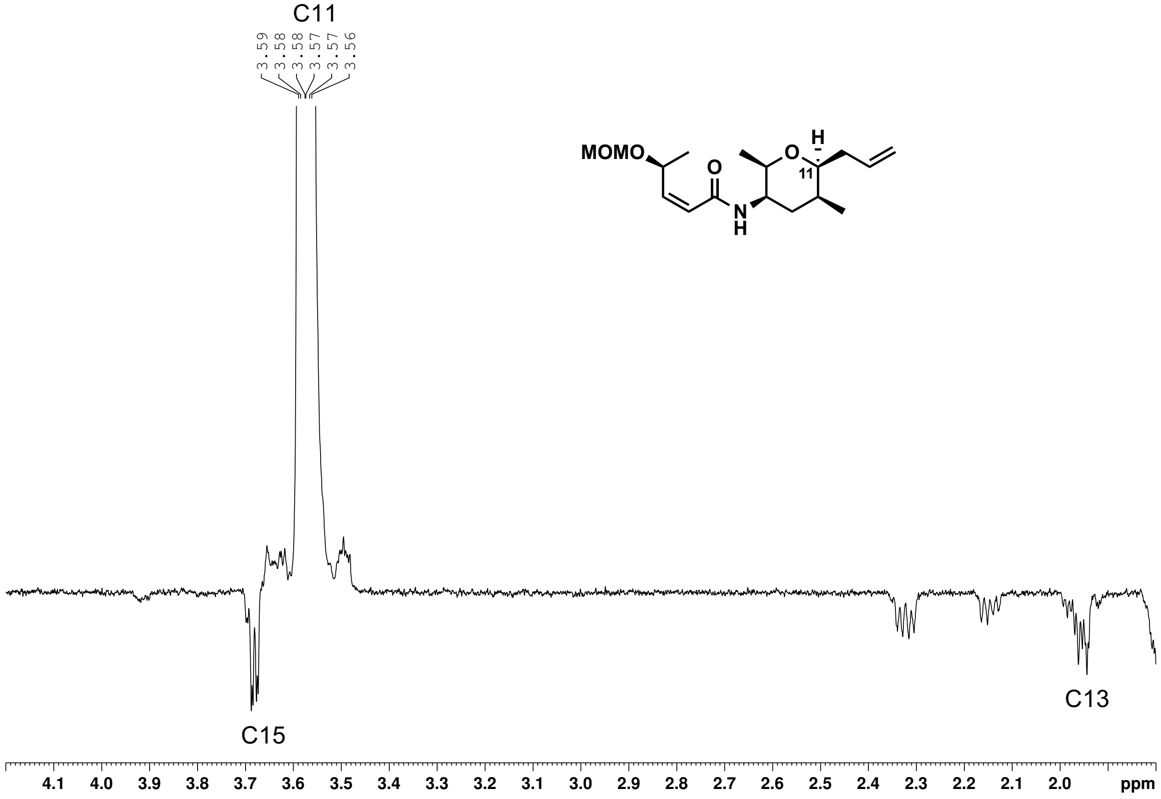

Figure S14. Selective 1D NOESY spectrum of amide **8** (600 MHz, CD_2Cl_2, 293K)

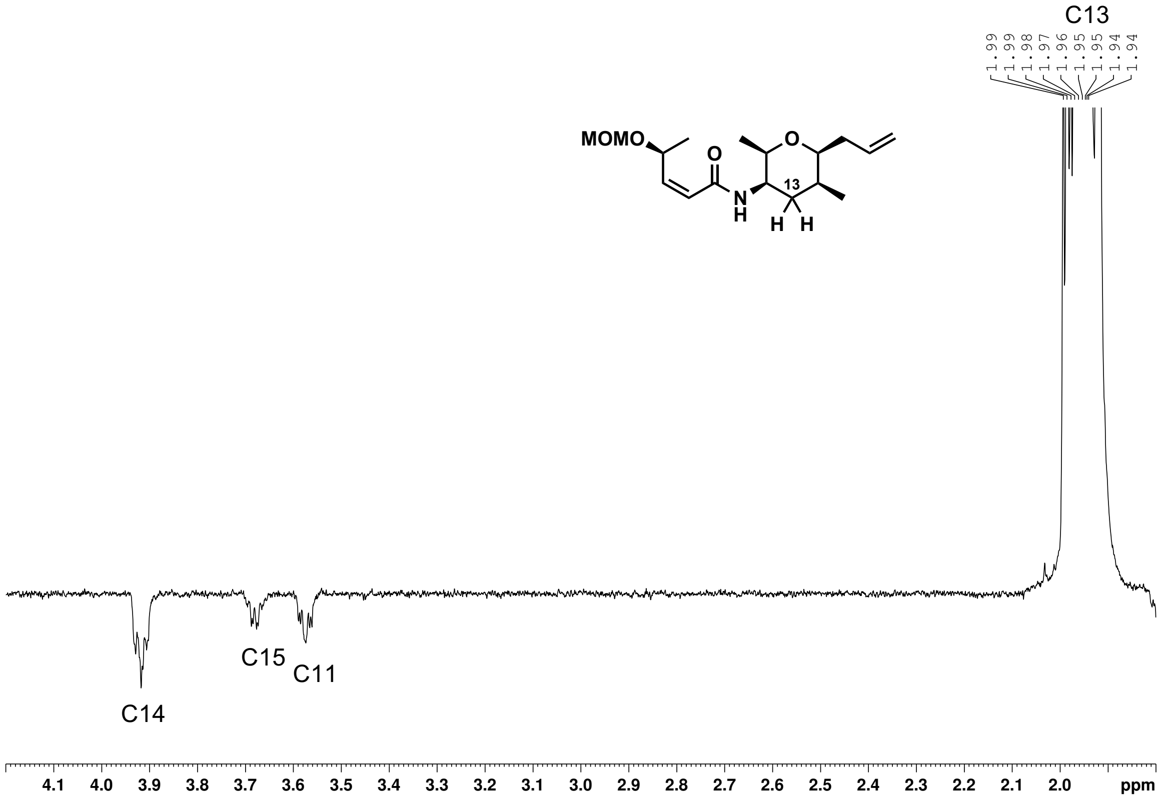

Figure S15. Selective 1D NOESY spectrum of amide **8** (600 MHz, CD_2Cl_2, 293K)

Figure S16. DFT structure of amide **8** in CH_2Cl_2, optimized at B3LYP/6-31G(d)

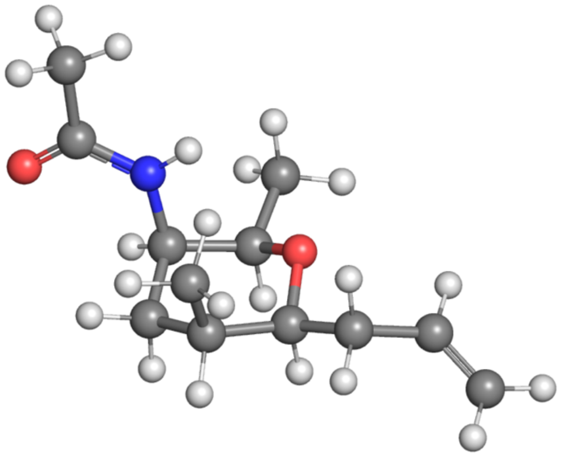

B3LYP/6-31G(d)

0 1

C	-4.87253300	1.61953400	-1.64347300
C	-4.33788500	0.30867300	-2.27135800
C	-3.29861200	-0.43319300	-1.40139400
C	-3.84570200	-0.57112500	0.02982300
C	-5.26402000	1.33427600	-0.17482800
H	-3.13179000	-1.42548700	-1.83187100
H	-5.18477700	-0.37609800	-2.42140600
H	-3.90799700	0.49707400	-3.26117700
H	-5.80195300	1.87273600	-2.17410400
H	-4.77275100	-1.16627000	-0.03363800
H	-6.10143500	0.61499500	-0.18545100
O	-4.17355400	0.72865200	0.53558100
C	-2.90262400	-1.23434300	1.02341800
H	-3.38638200	-1.31184100	2.00181700
H	-2.63752900	-2.24068200	0.68184300
H	-1.97870900	-0.66073400	1.14141100
C	-3.92068400	2.81761600	-1.80464300
H	-3.54199400	2.87298800	-2.83118400
H	-4.43354200	3.76142800	-1.59031000
H	-3.05714700	2.75691800	-1.13540900
N	-1.99453000	0.22074300	-1.38288300
C	-1.01596500	-0.06841100	-2.29165100
O	-1.16062800	-0.90674400	-3.17485500
C	0.27826800	0.71857800	-2.13479400
H	0.46183100	1.28377200	-3.05401300
H	0.27699600	1.41089800	-1.28676800

H	1.10634200	0.01309000	-2.01668500
H	-1.84629800	0.94885700	-0.69919200
H	-6.44169700	3.12647100	0.05445200
H	-4.82216800	3.21585800	0.75716800
C	-5.69785000	2.56406700	0.63328200
C	-6.25619000	2.20656400	1.98445400
H	-5.59868800	1.61341900	2.61859200
C	-7.46507000	2.55386900	2.42686000
H	-8.15060800	3.14393500	1.82088600
H	-7.81732400	2.26714600	3.41428700

Figure S17. Cell Cytotoxicity Assays

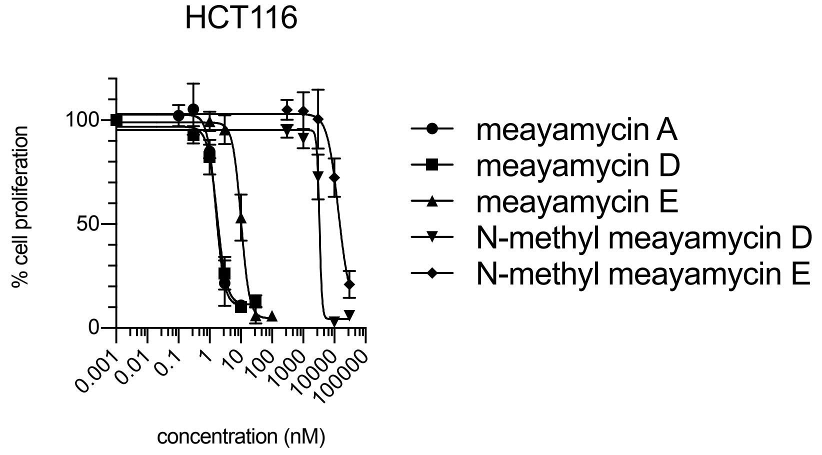

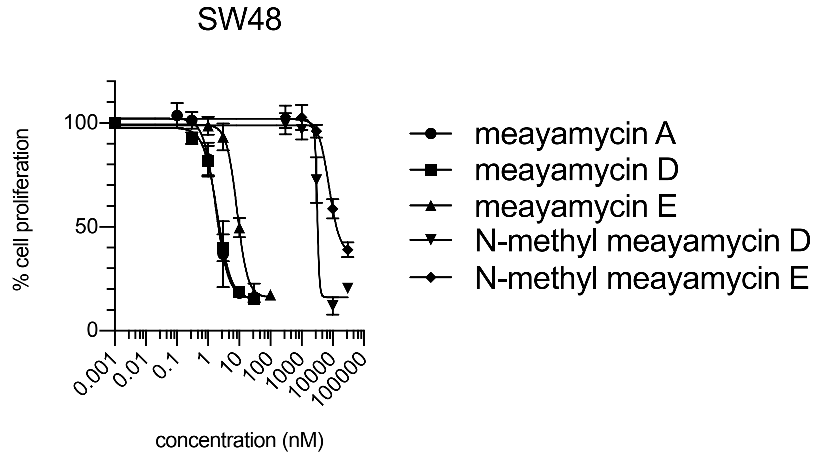

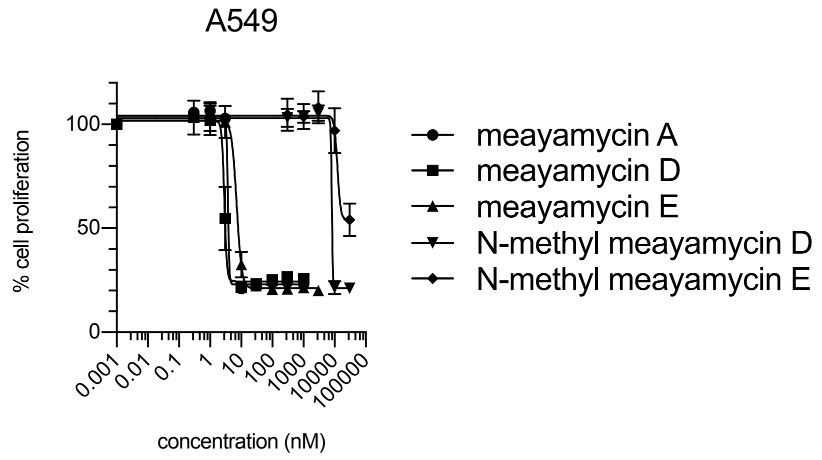

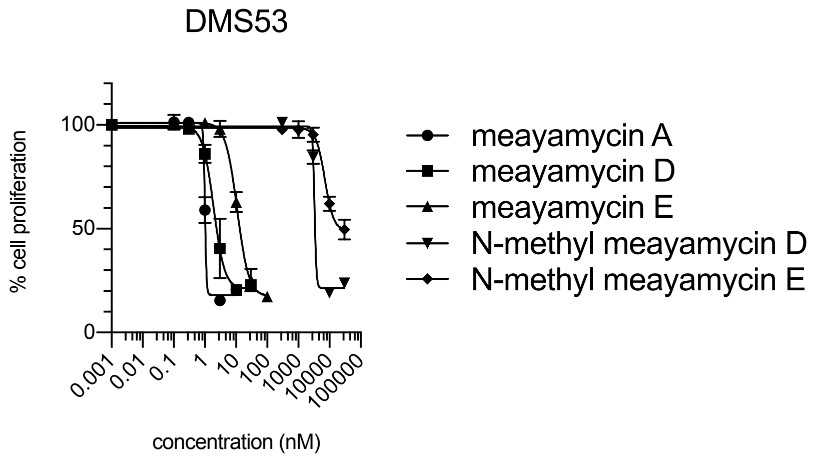
DMS53
% cell proliferation
100
50
0
0.001
0.01
0.1
1
10
100
1000
10000
100000
concentration (nM)
meayamycin A
meayamycin D
meayamycin E
N-methyl meayamycin D
N-methyl meayamycin E

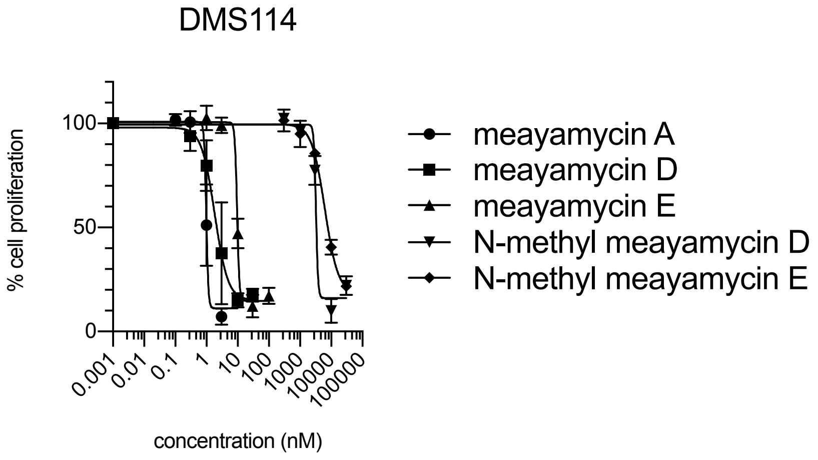
DMS114
% cell proliferation
100
50
0
0.001
0.01
0.1
1
10
100
1000
10000
100000
concentration (nM)
meayamycin A
meayamycin D
meayamycin E
N-methyl meayamycin D
N-methyl meayamycin E

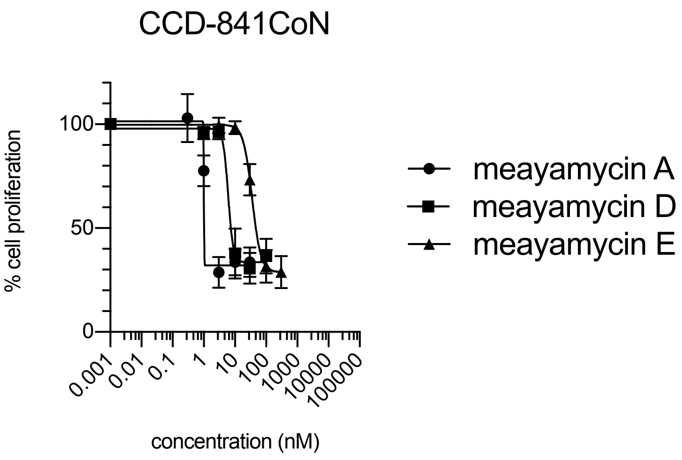
CCD-841CoN
% cell proliferation
100
50
0
0.001
0.01
0.1
1
10
100
1000
10000
100000
concentration (nM)
meayamycin A
meayamycin D
meayamycin E

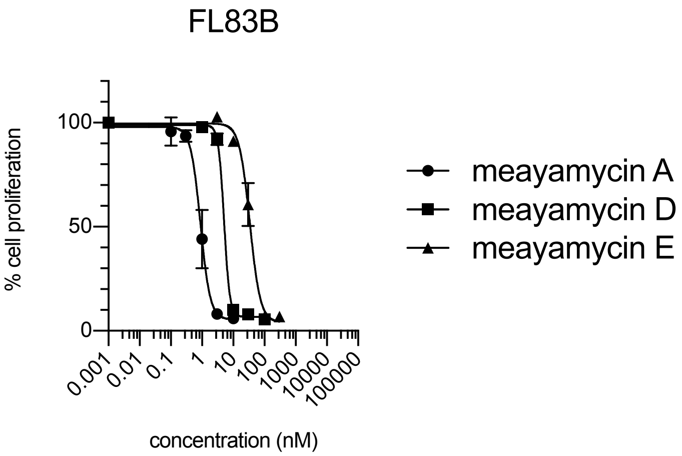
FL83B
100
50
0
% cell proliferation
0.001
0.01
0.1
1
10
100
1000
10000
100000
concentration (nM)
meayamycin A
meayamycin D
meayamycin E

Figure S18. RT-PCR

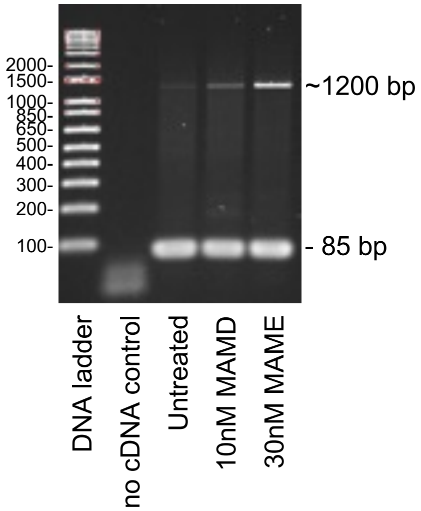

Figure 18S. RT-PCR. DMS114 cells were treated for 8 hr with 10 nM MAMD and 30 nM MAME. Cellular RNA was extracted using RNeasy kit. cDNA was synthesized and PCR was performed. PCR primer sequences were obtained from ref 45.

LCMS traces for meayamycin analogs

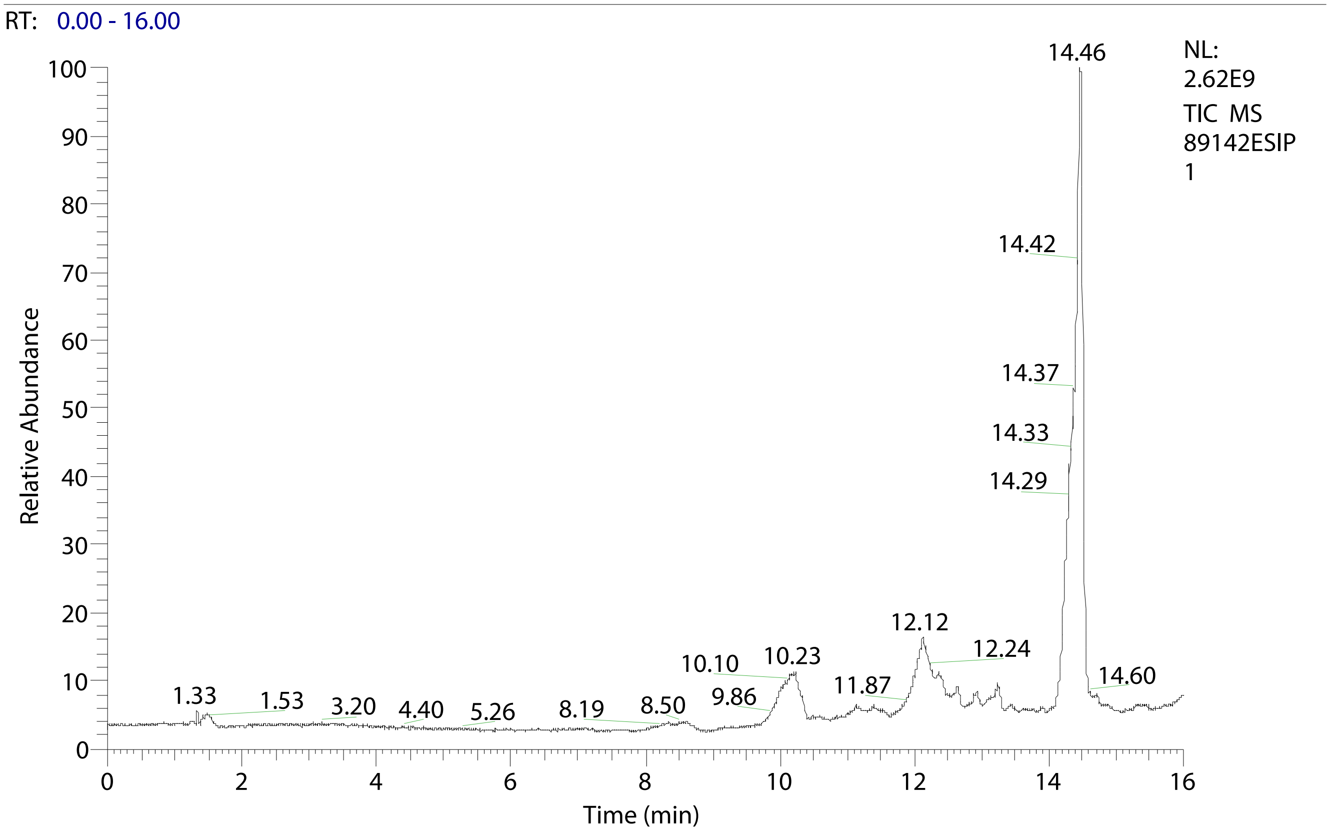

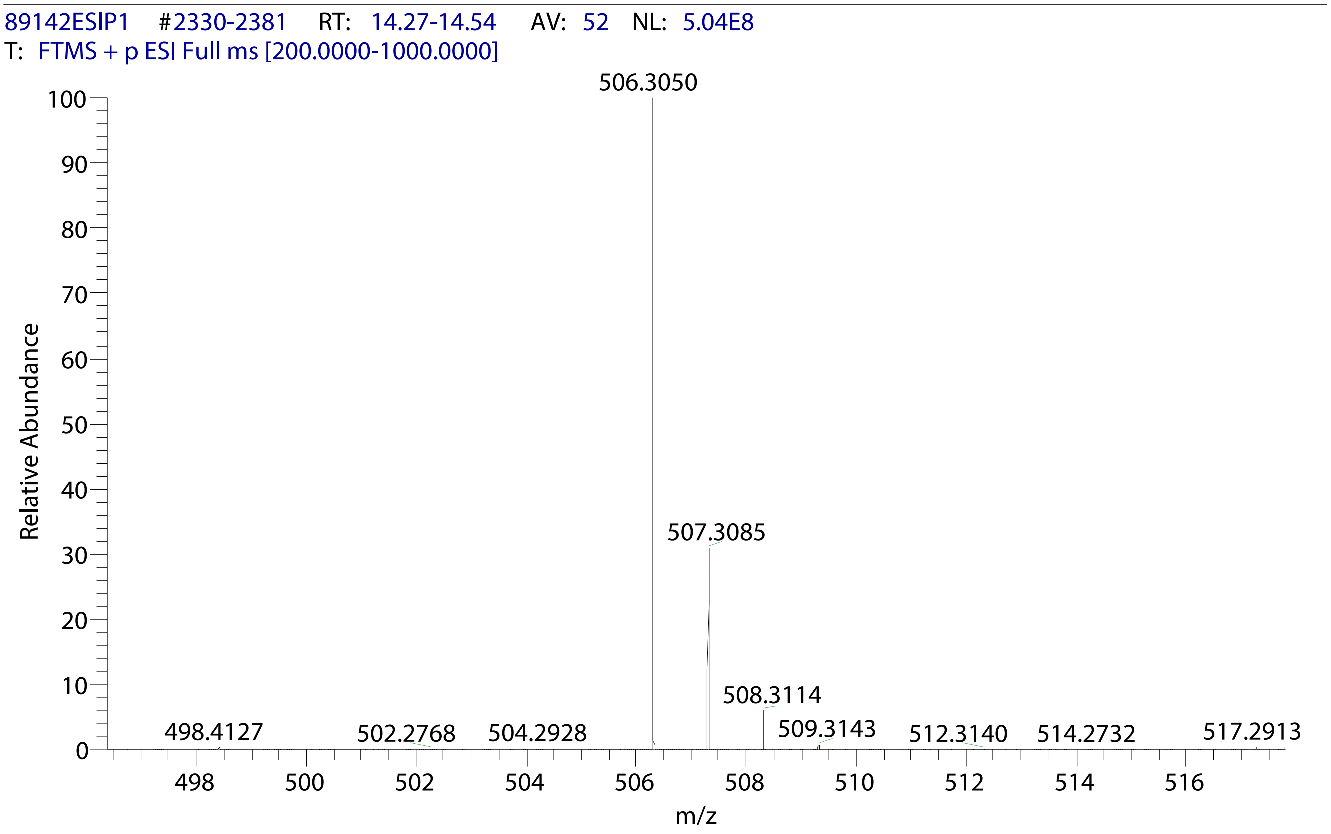

LCMS trace of meayamycin A

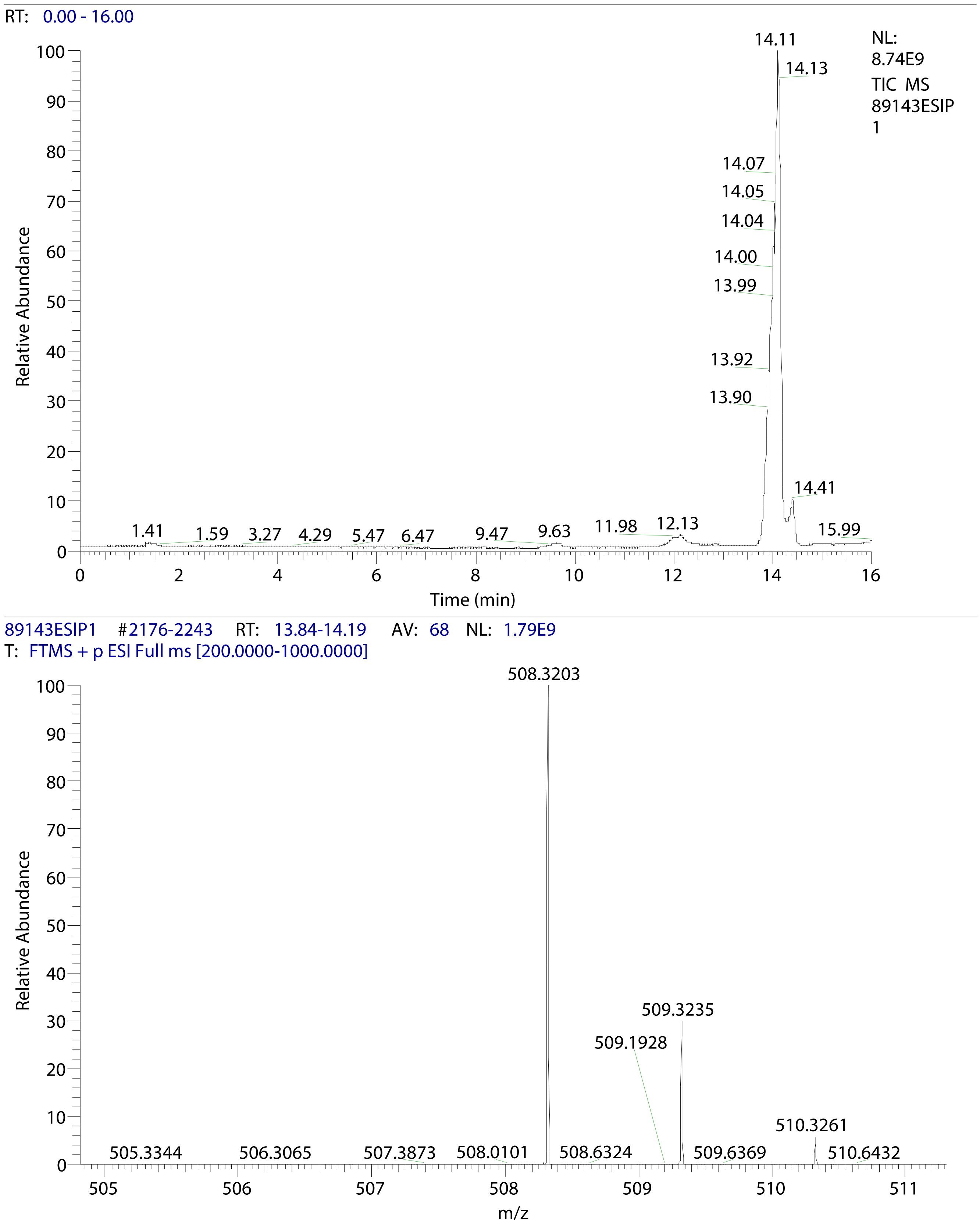

LCMS trace of meayamycin D

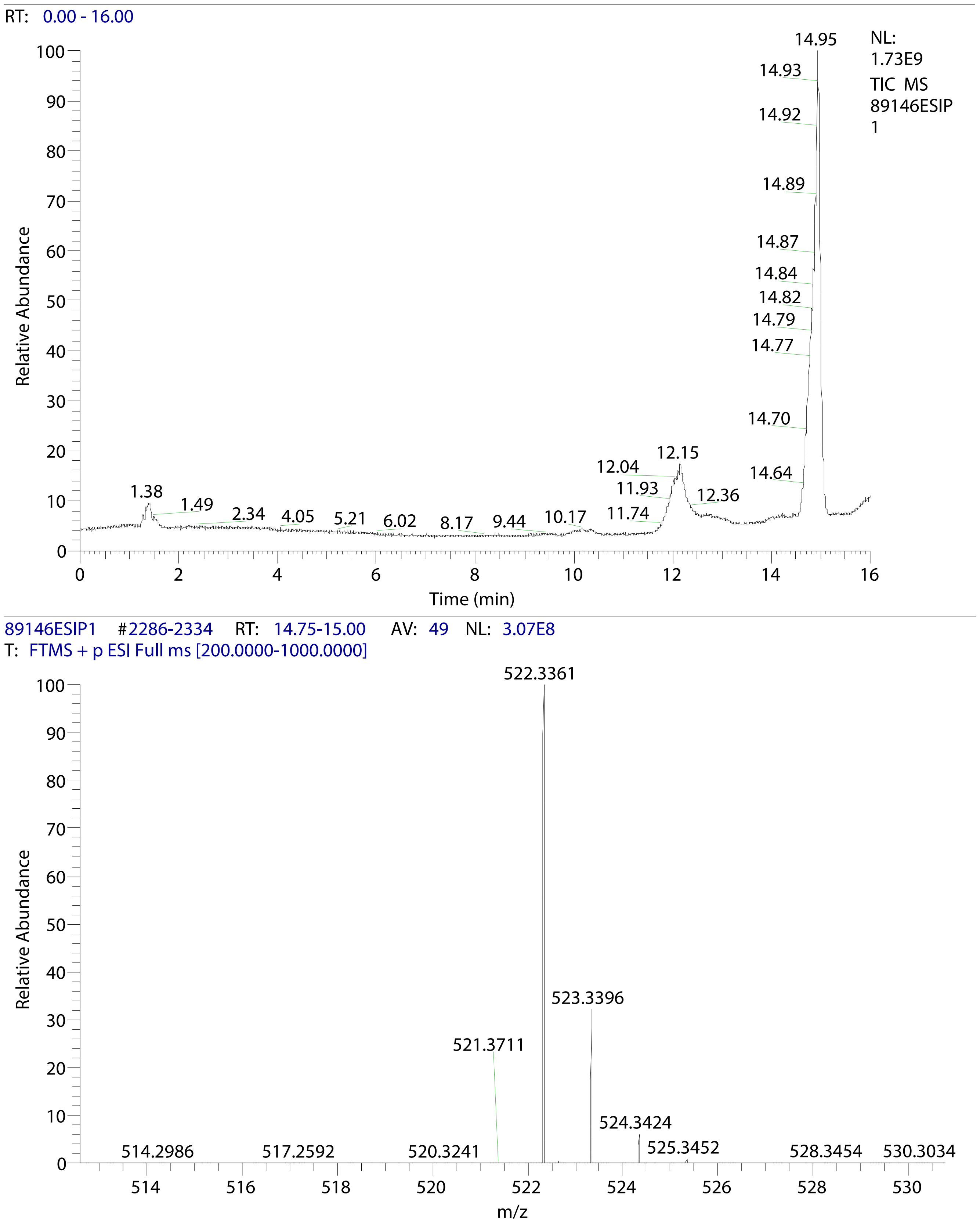

LCMS trace of N-methyl meayamycin D

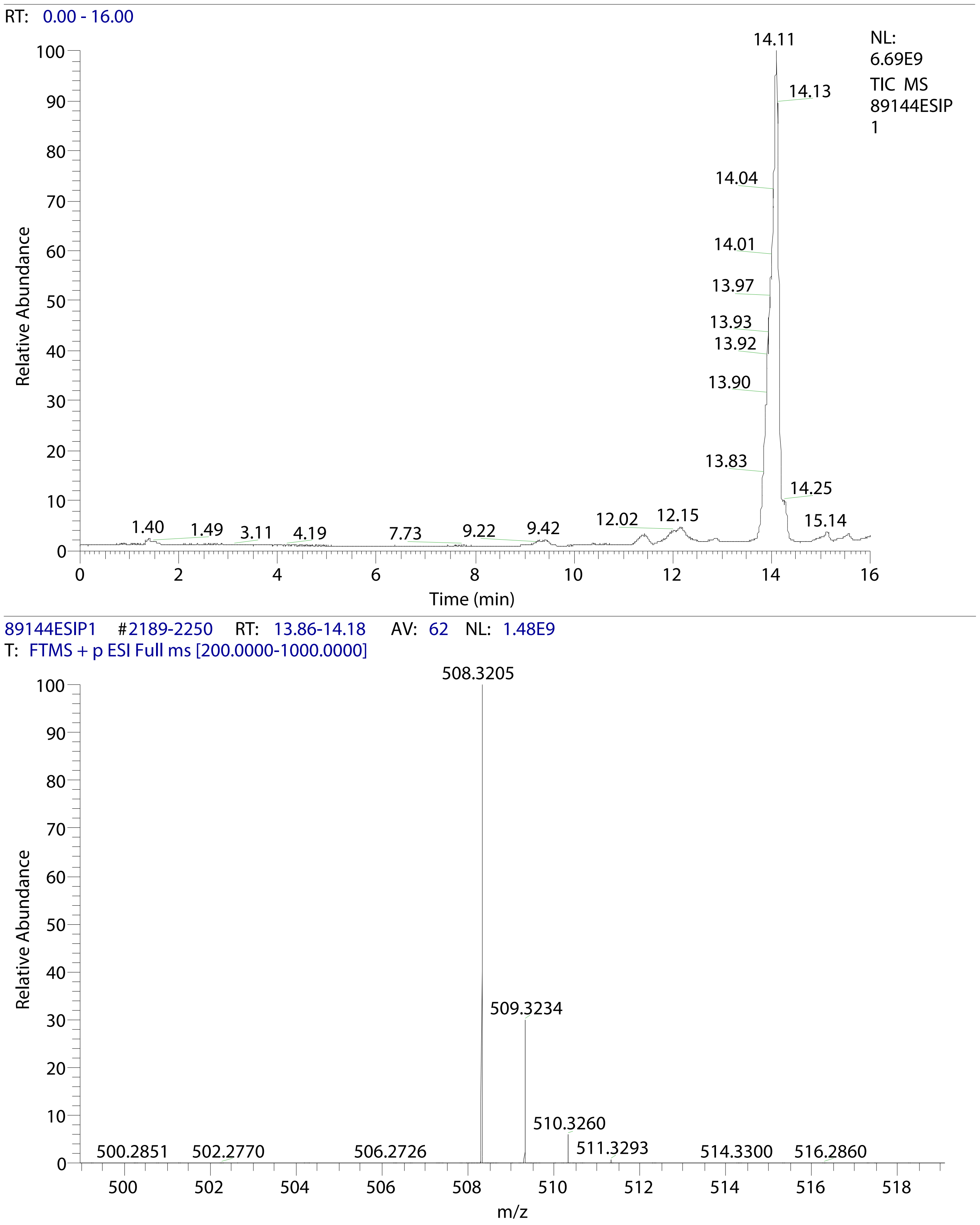

LCMS trace of N-methyl meayamycin E

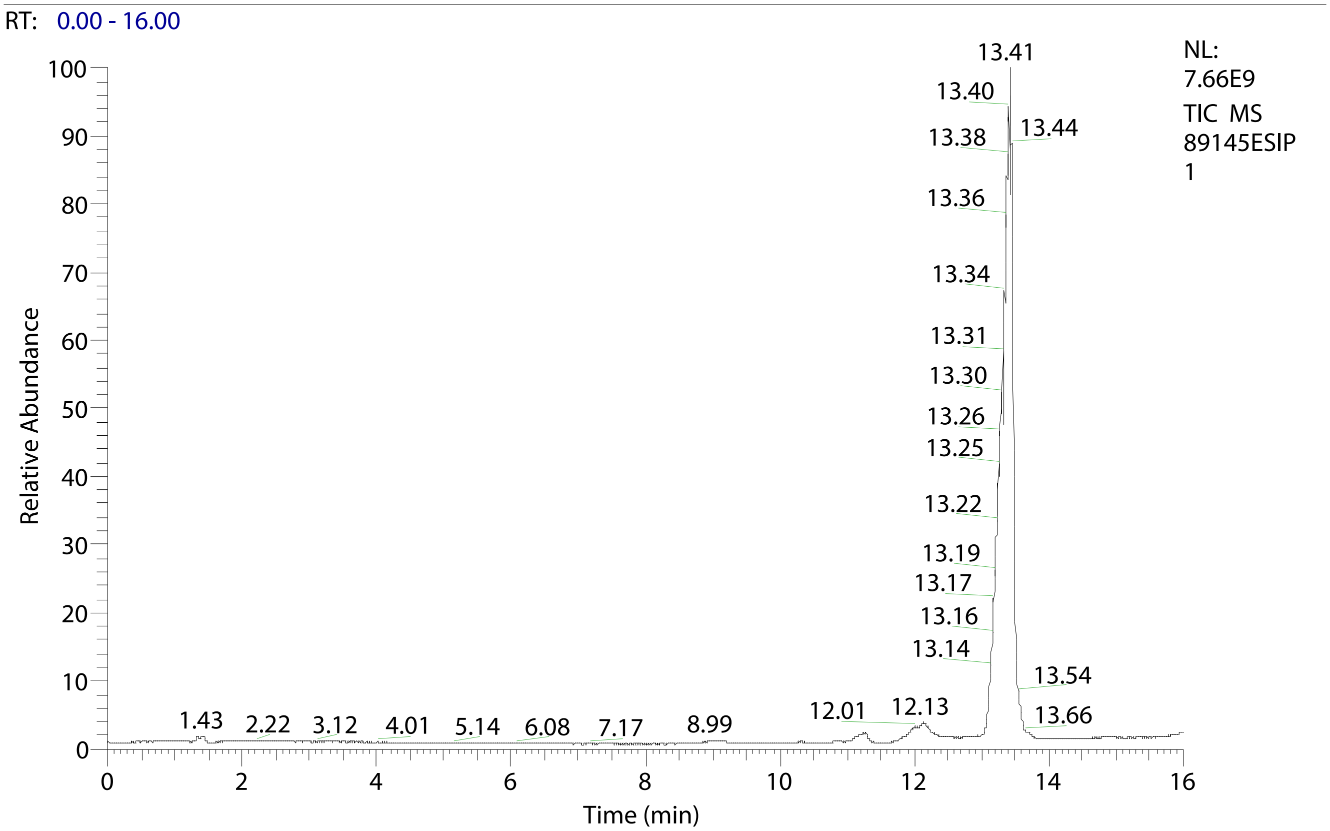

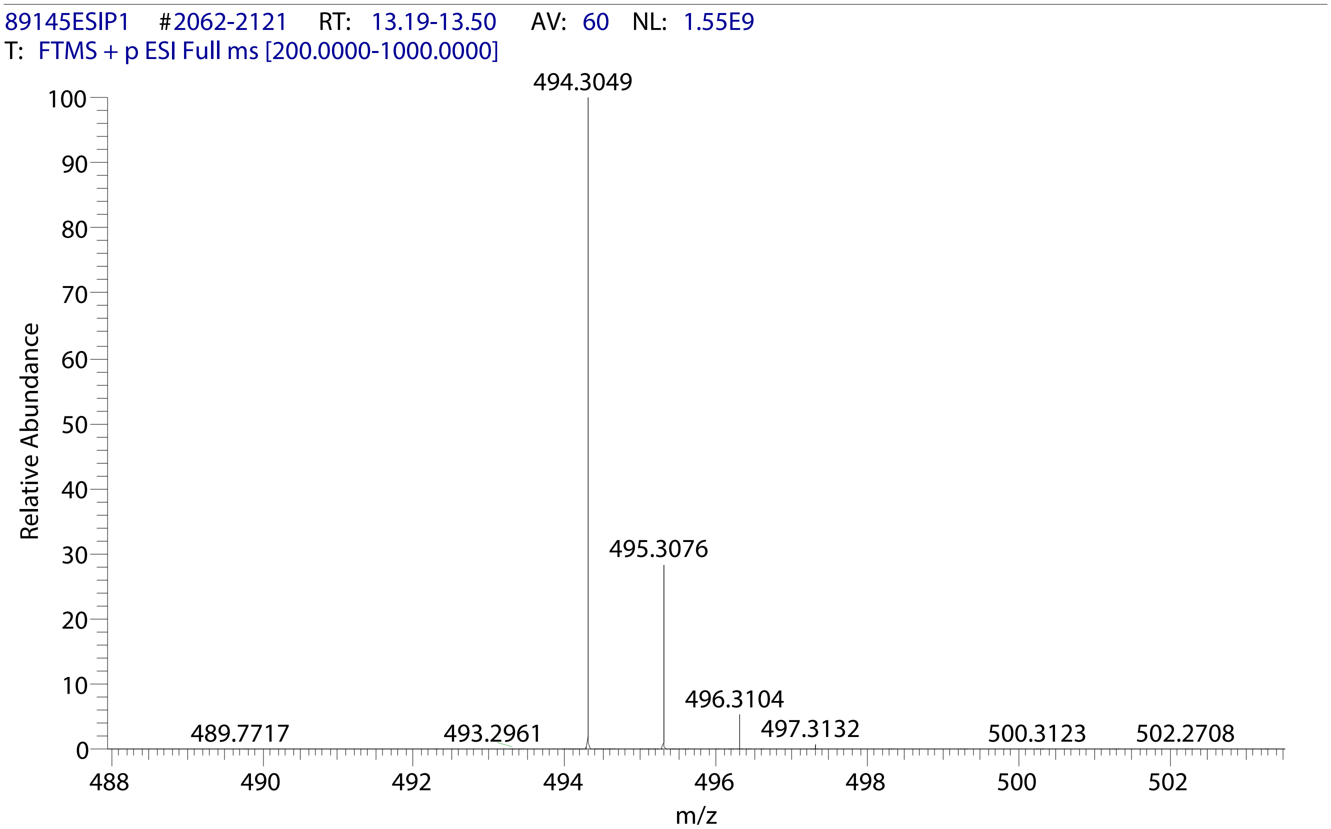

LCMS trace of meayamycin E

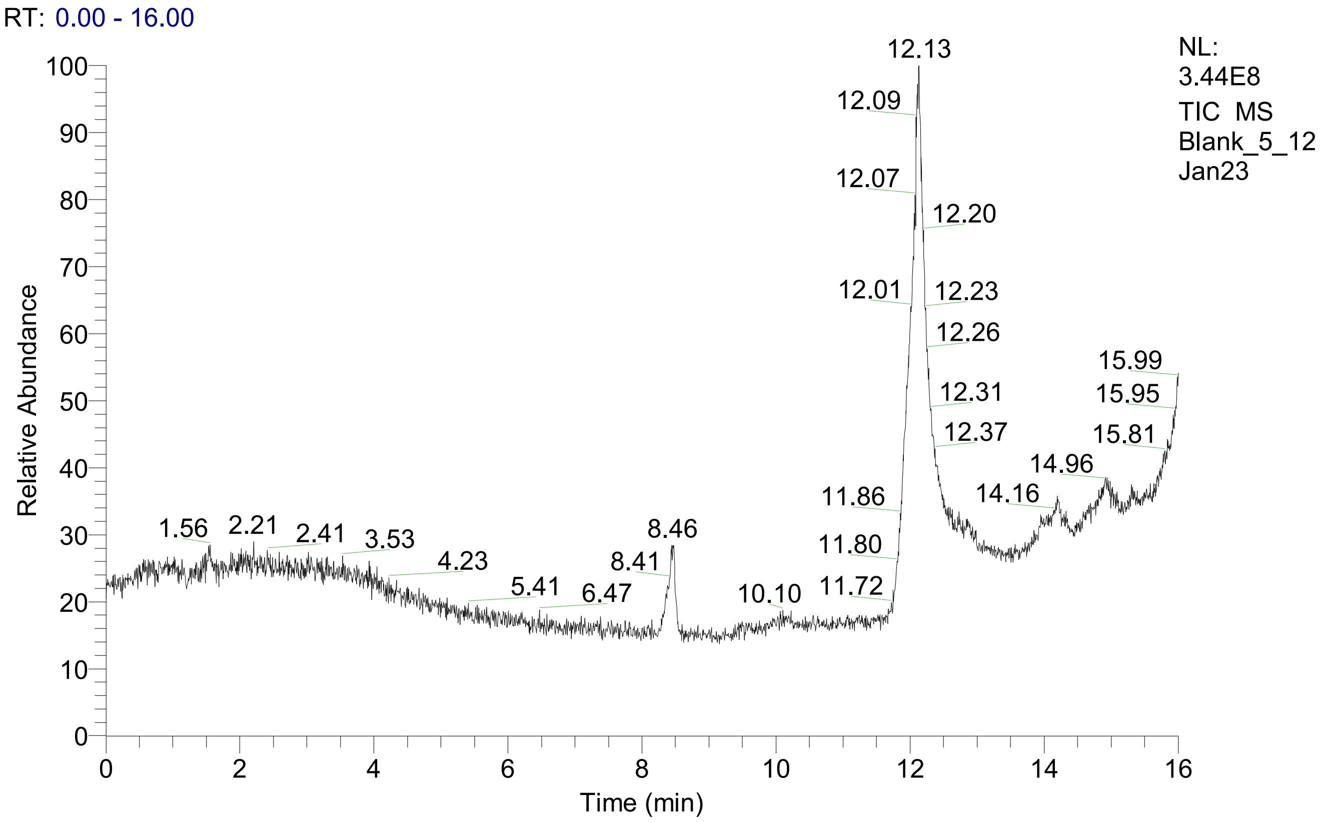

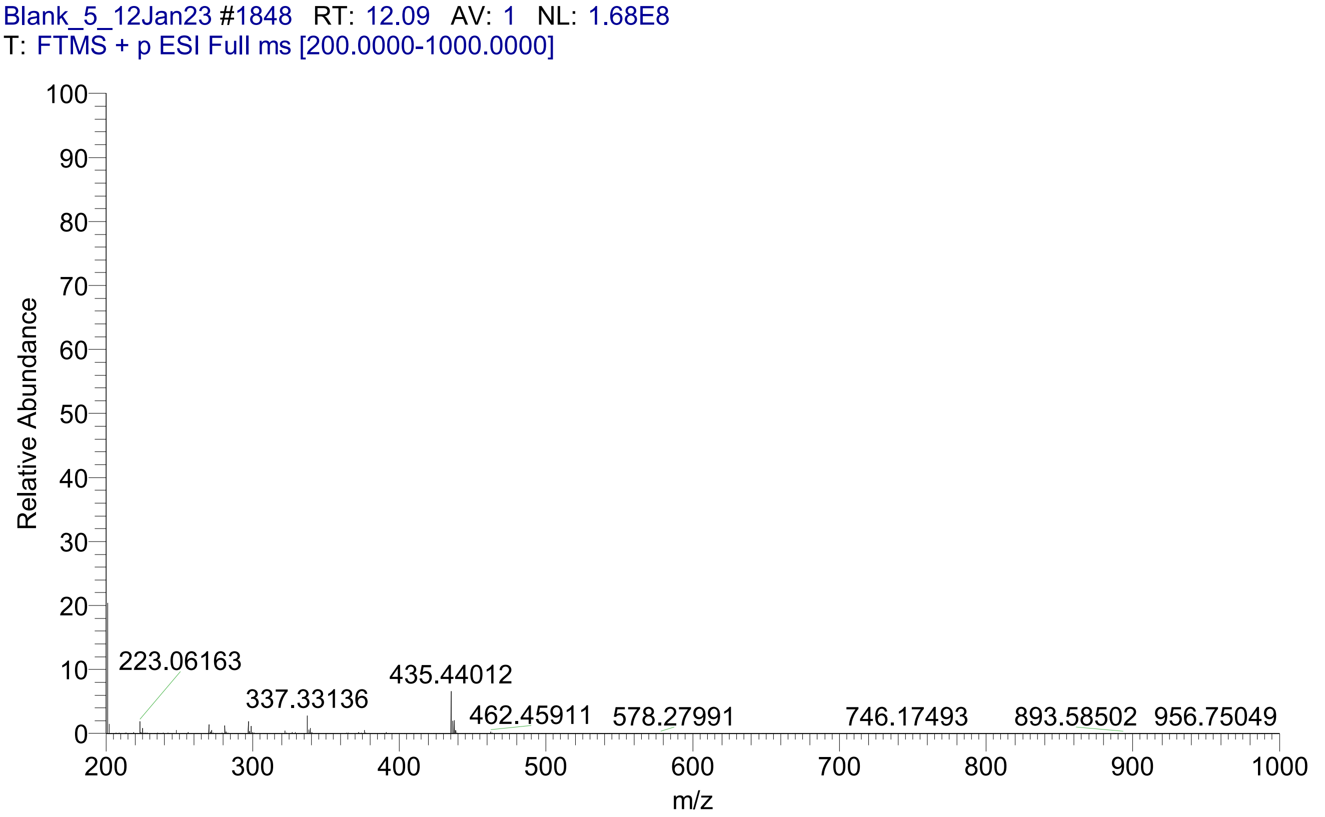

LCMS trace of water blank

NMR Spectra

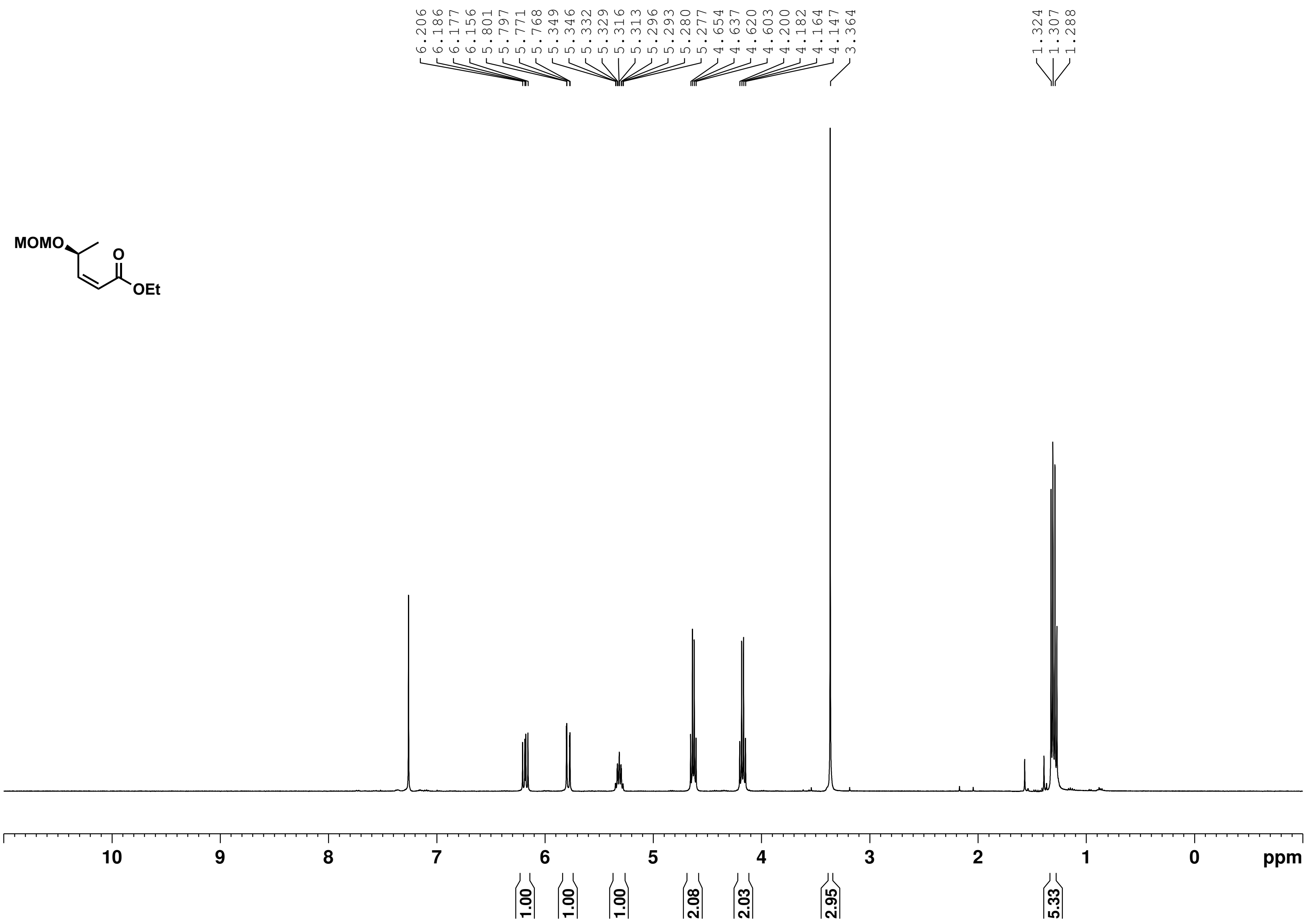

^{1}H NMR spectrum of ethyl (*S*,*Z*)-4-(methoxymethoxy)pent-2-enoate **3** (400 MHz, $CDCl_3$, 293K)

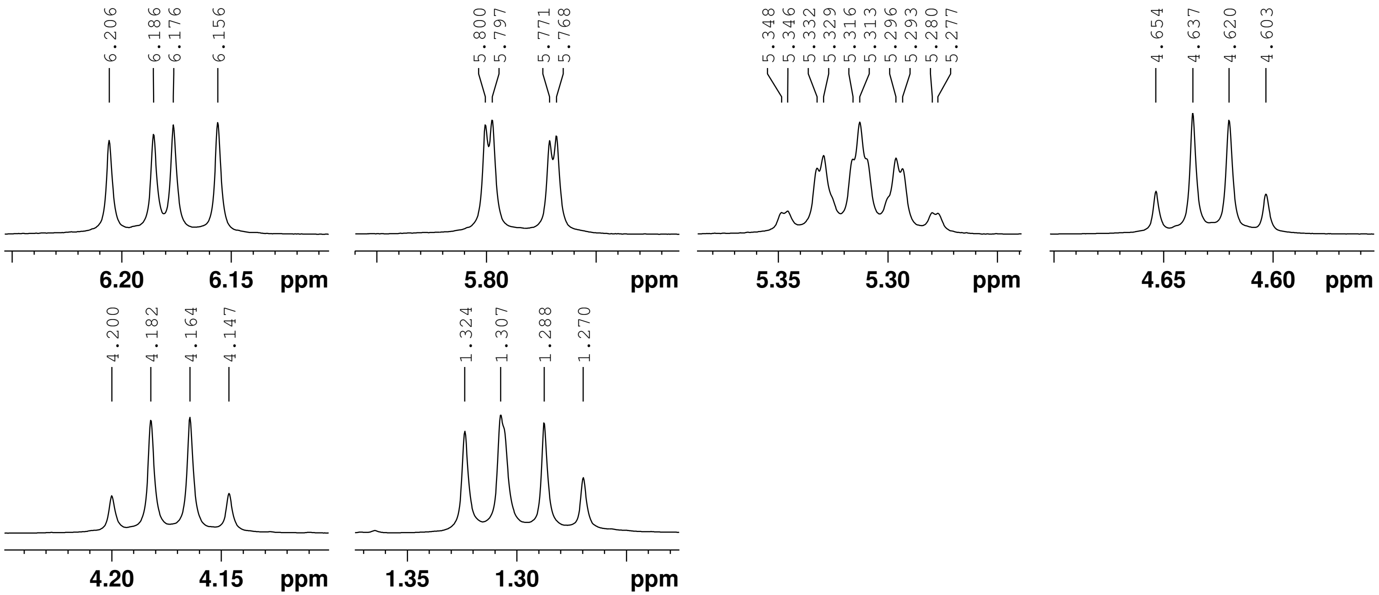

^{1}H NMR spectrum of ethyl (*S*,*Z*)-4-(methoxymethoxy)pent-2-enoate **3** (400 MHz, $CDCl_3$, 293K)

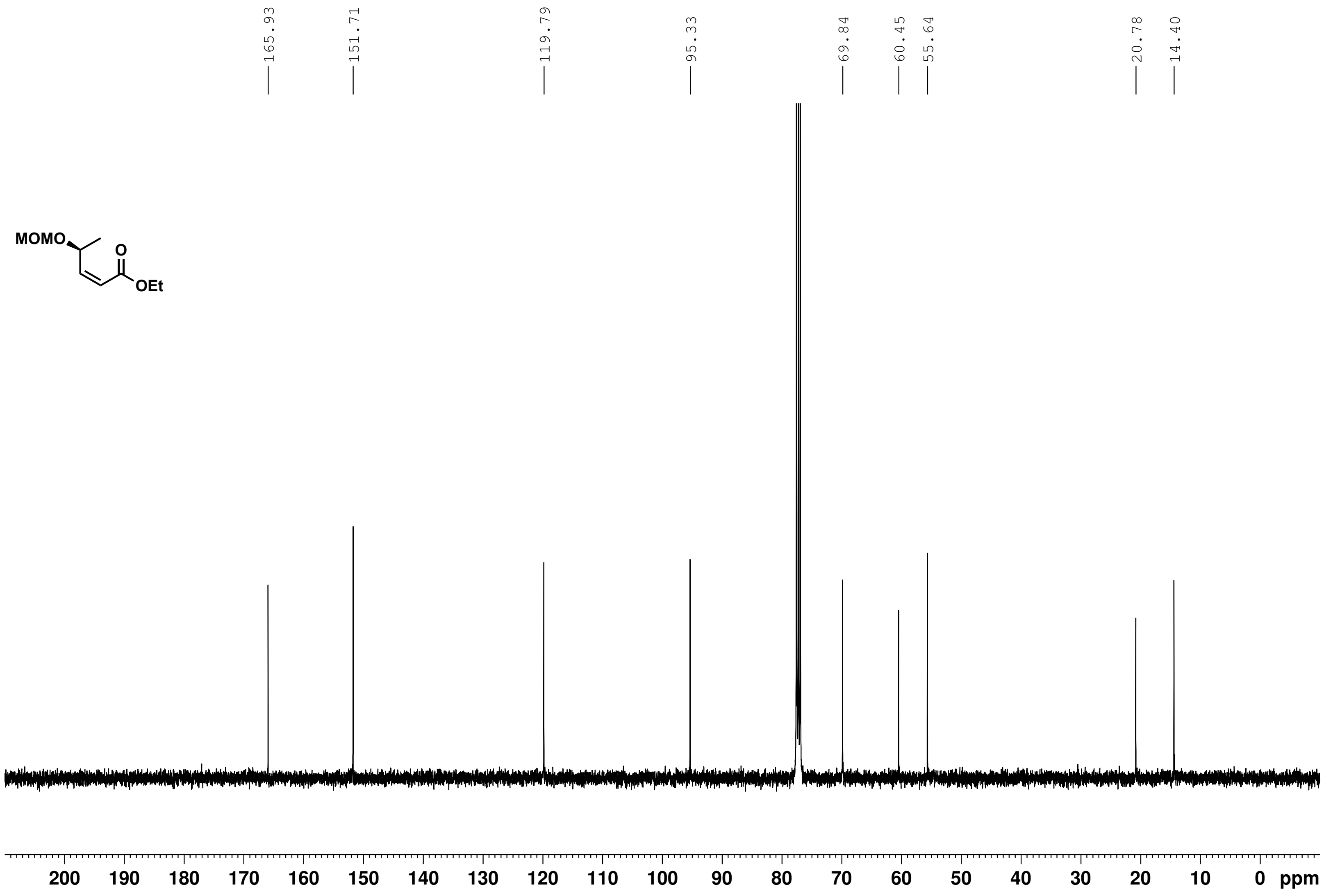

^{13}C NMR spectrum of ethyl (*S*,*Z*)-4-(methoxymethoxy)pent-2-enoate **3** (100 MHz, $CDCl_3$, 293K)

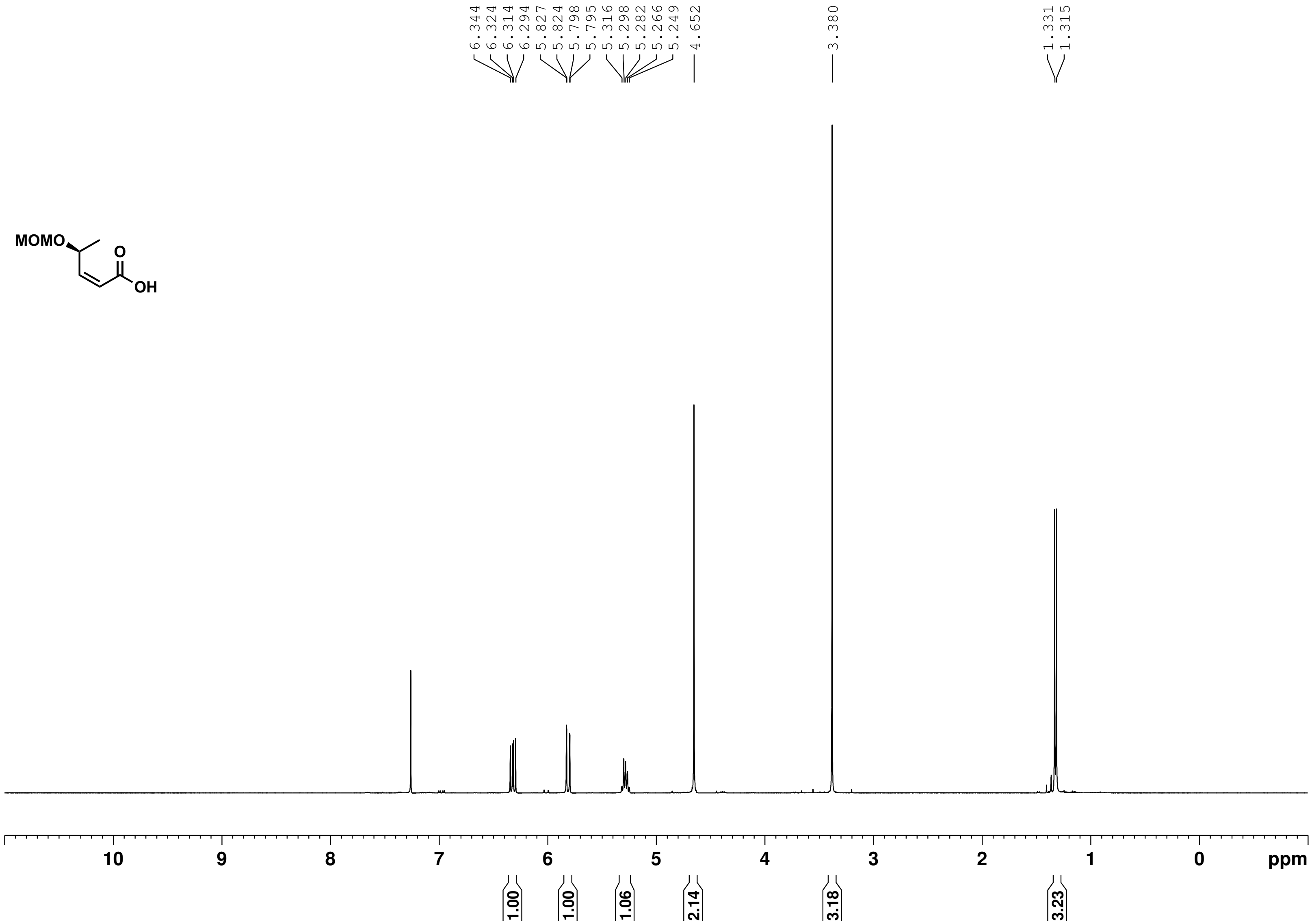

^{1}H NMR spectrum of (*S*,*Z*)-4-(methoxymethoxy)pent-2-enoic acid **4** (400 MHz, 1% CD_3OD in $CDCl_3$, 293K)

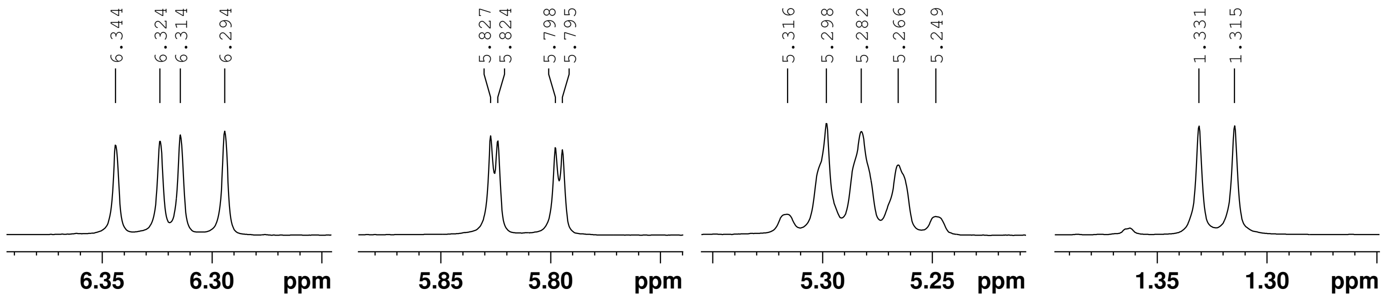

^{1}H NMR spectrum of (*S*,*Z*)-4-(methoxymethoxy)pent-2-enoic acid **4** (400 MHz, 1% CD_3OD in $CDCl_3$, 293K)

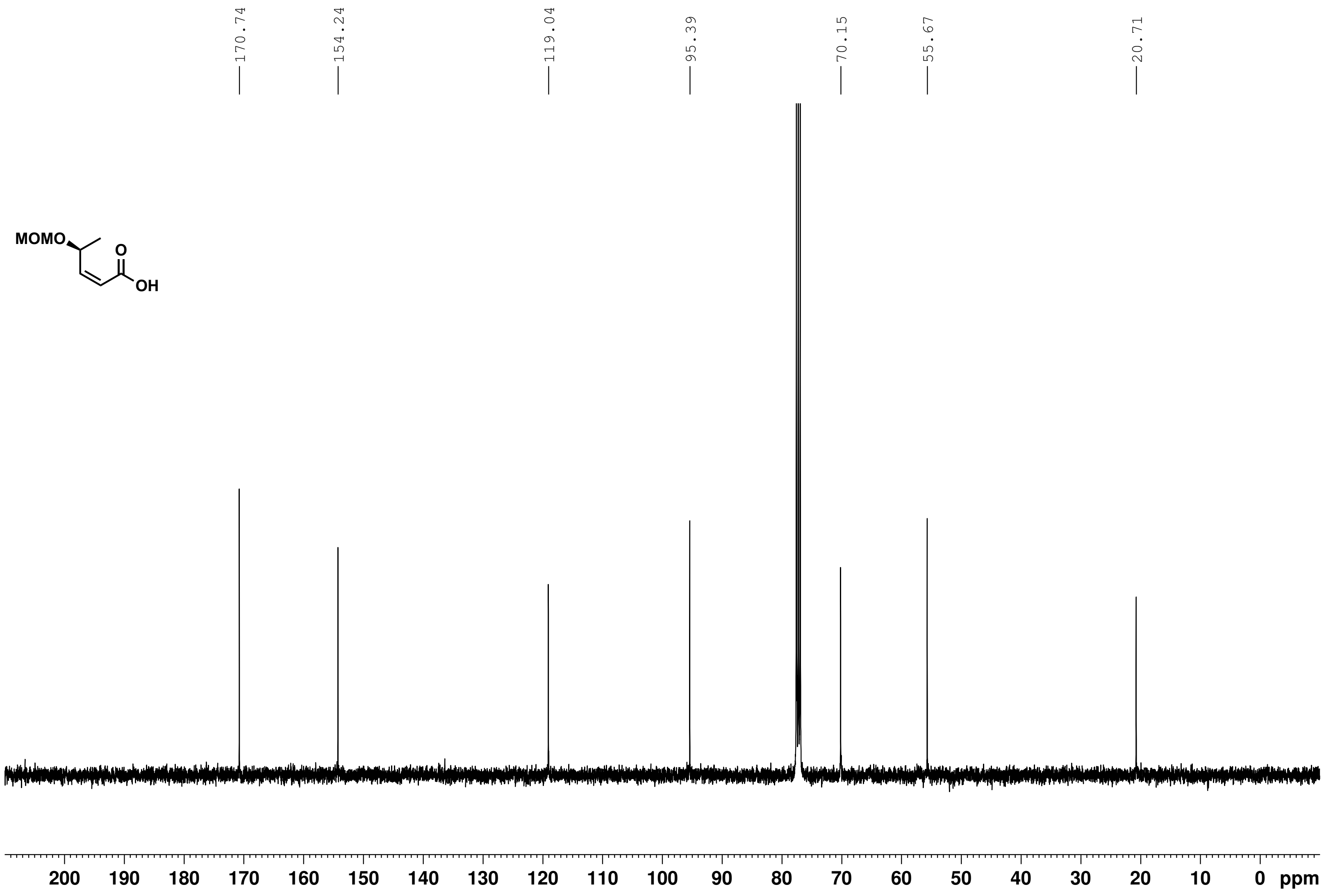

^{13}C NMR spectrum of (*S*,*Z*)-4-(methoxymethoxy)pent-2-enoic acid **4** (100 MHz, 1% CD_3OD in $CDCl_3$, 293K)

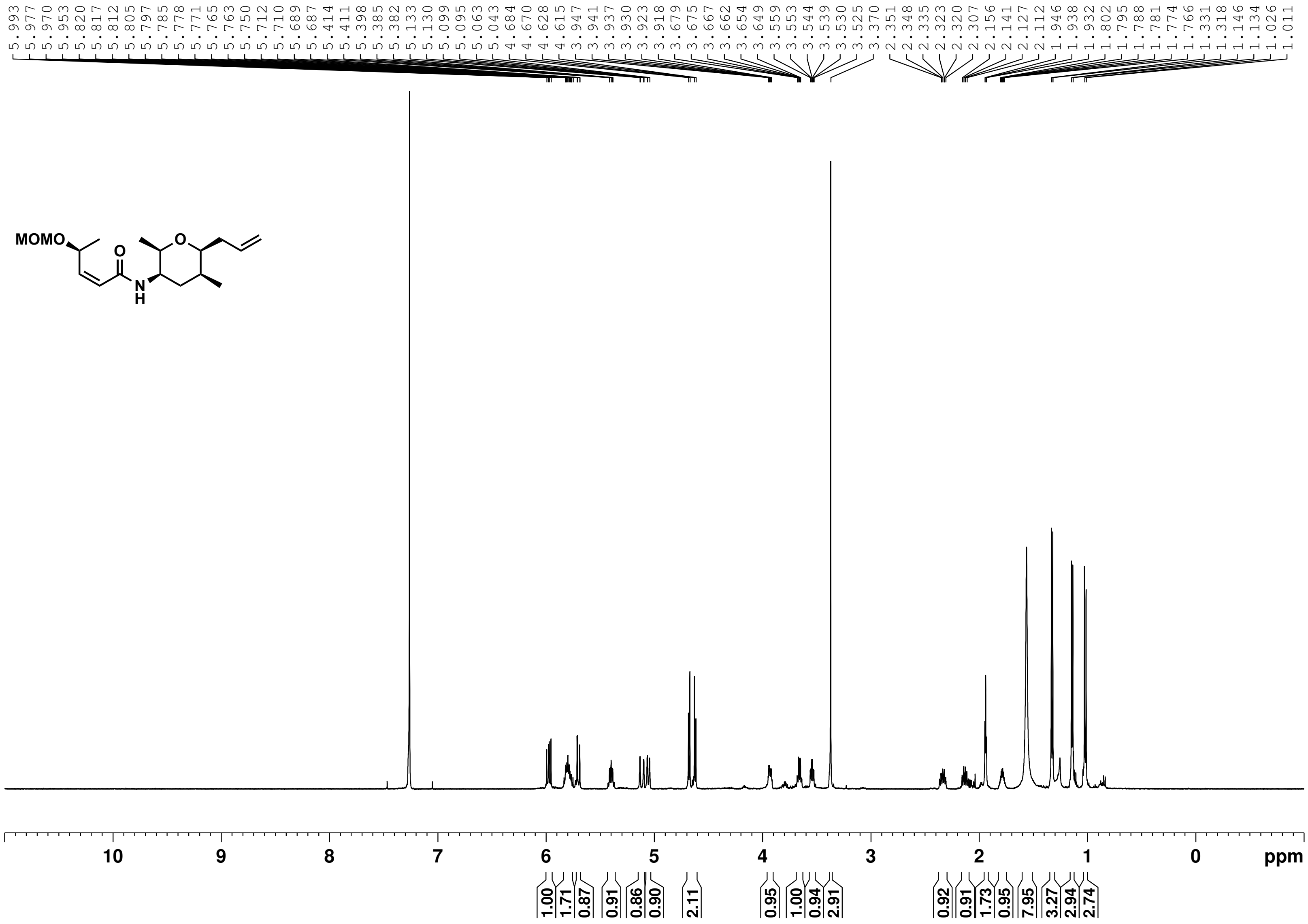

^{1}H NMR spectrum of (*S*,*Z*)-*N*-((2*R*,3*R*,5*S*,6*S*)-6-allyl-2,5-dimethyltetrahydro-2*H*-pyran-3-yl)-4-(methoxymethoxy)pent-2-enamide **8**
(500 MHz, $CDCl_3$, 293K)

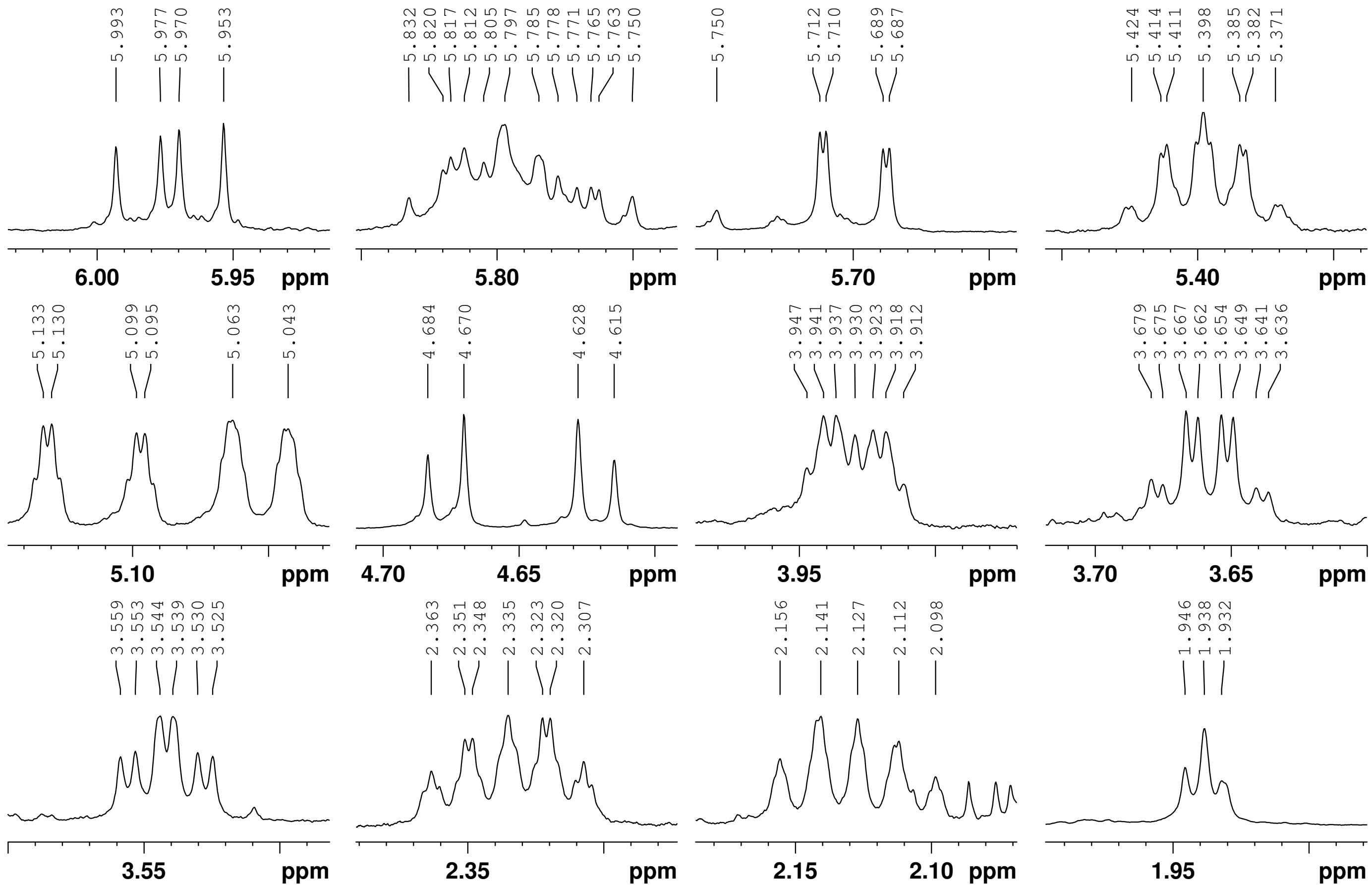

^{1}H NMR spectrum of (*S*,*Z*)-*N*-((2*R*,3*R*,5*S*,6*S*)-6-allyl-2,5-dimethyltetrahydro-2*H*-pyran-3-yl)-4-(methoxymethoxy)pent-2-enamide **8**
(500 MHz, $CDCl_3$, 293K)

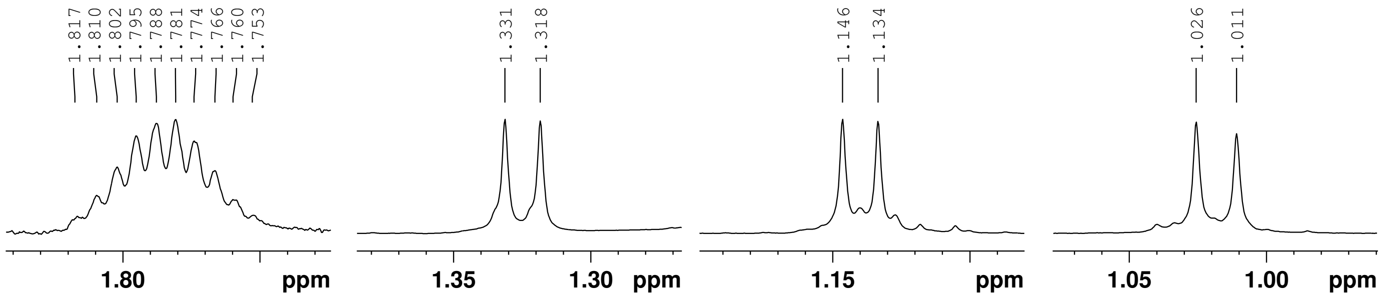

^{1}H NMR spectrum of (*S*,*Z*)-*N*-((2*R*,3*R*,5*S*,6*S*)-6-allyl-2,5-dimethyltetrahydro-2*H*-pyran-3-yl)-4-(methoxymethoxy)pent-2-enamide **8** (500 MHz, $CDCl_3$, 293K)

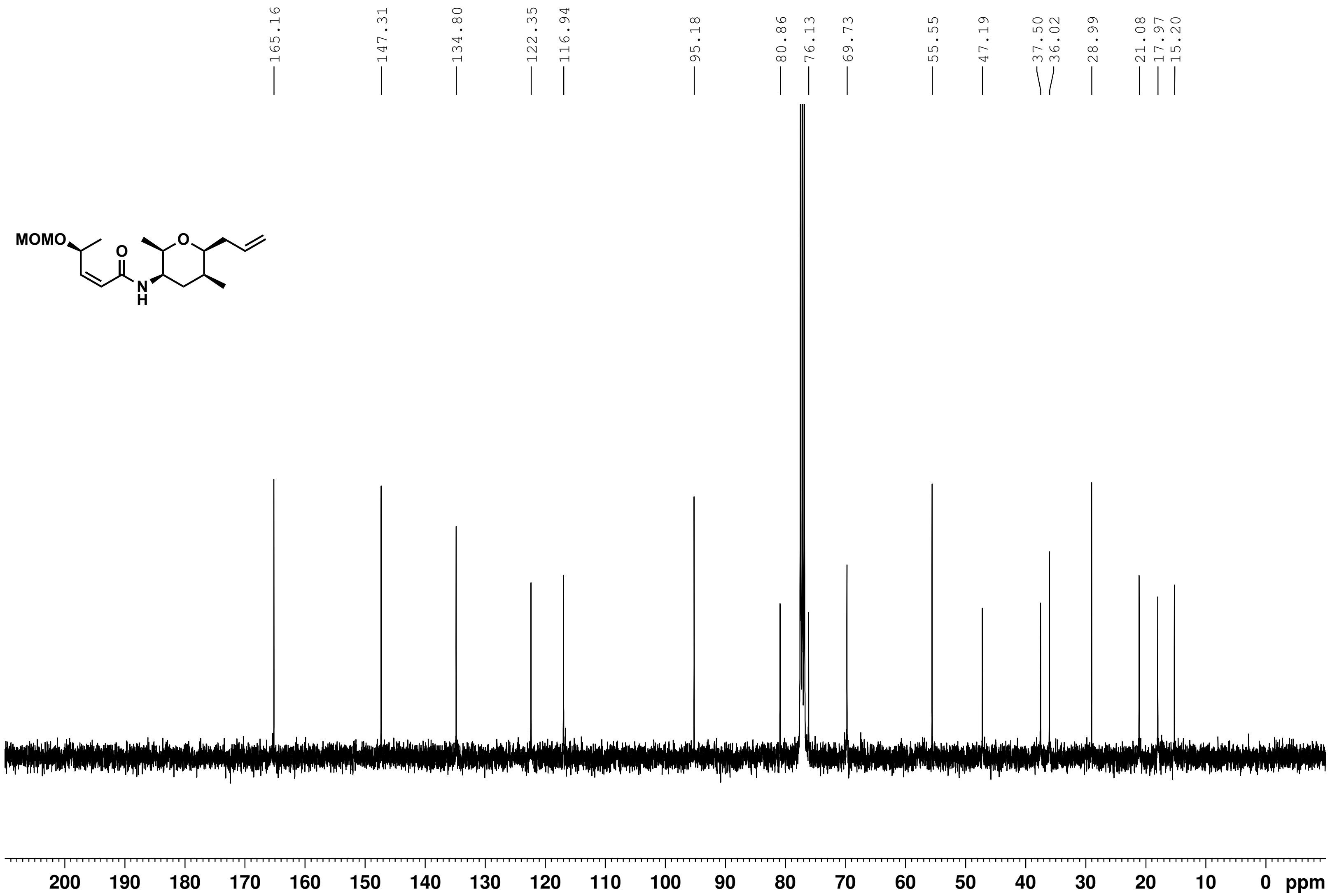

^{13}C NMR spectrum of (*S*,*Z*)-*N*-((2*R*,3*R*,5*S*,6*S*)-6-allyl-2,5-dimethyltetrahydro-2*H*-pyran-3-yl)-4-(methoxymethoxy)pent-2-enamide **8** (100 MHz, $CDCl_3$, 293K)

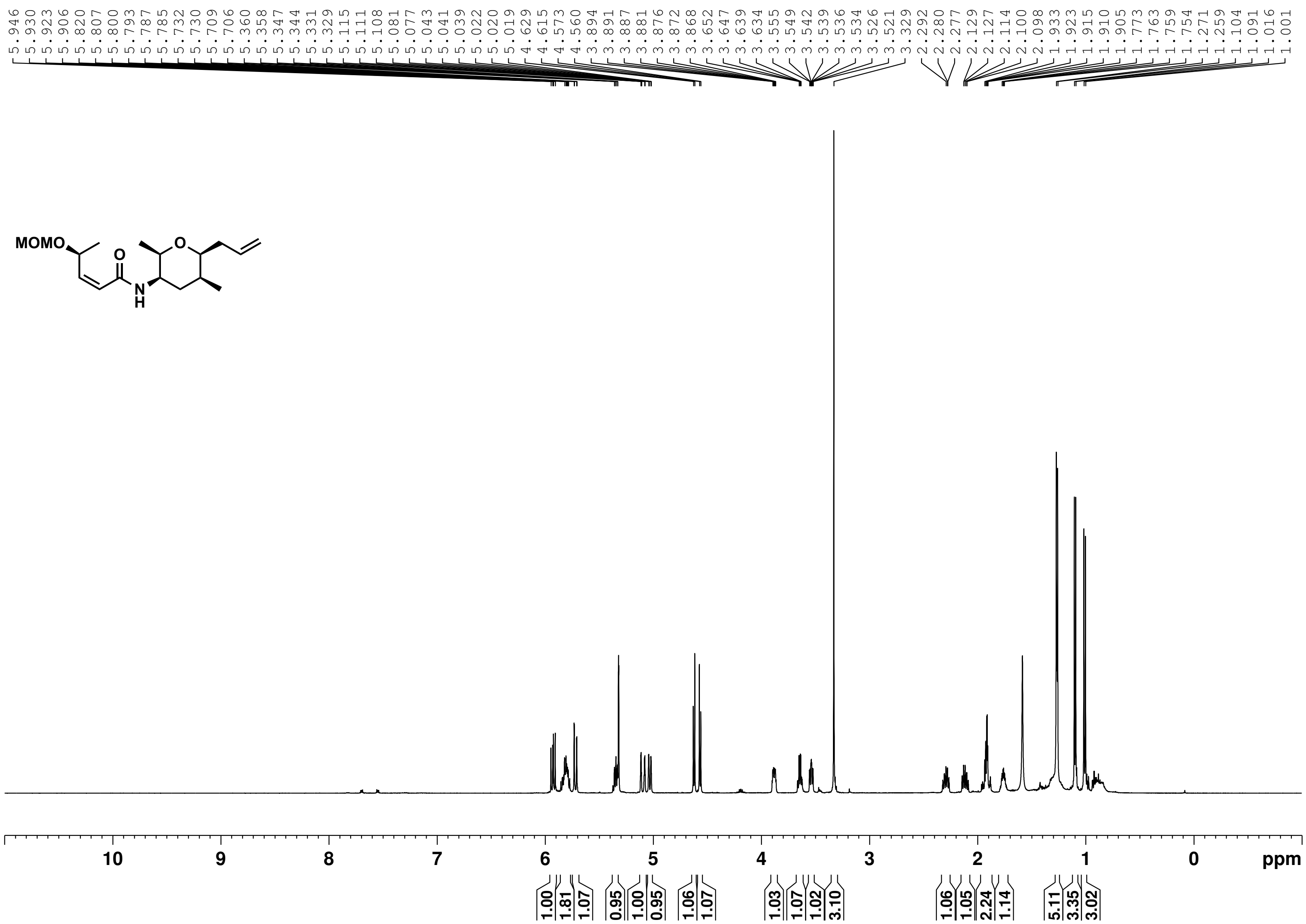

^{1}H NMR spectrum of (*S*,*Z*)-*N*-((2*R*,3*R*,5*S*,6*S*)-6-allyl-2,5-dimethyltetrahydro-2*H*-pyran-3-yl)-4-(methoxymethoxy)pent-2-enamide **8**
(500 MHz, CD_2Cl_2, 293K)

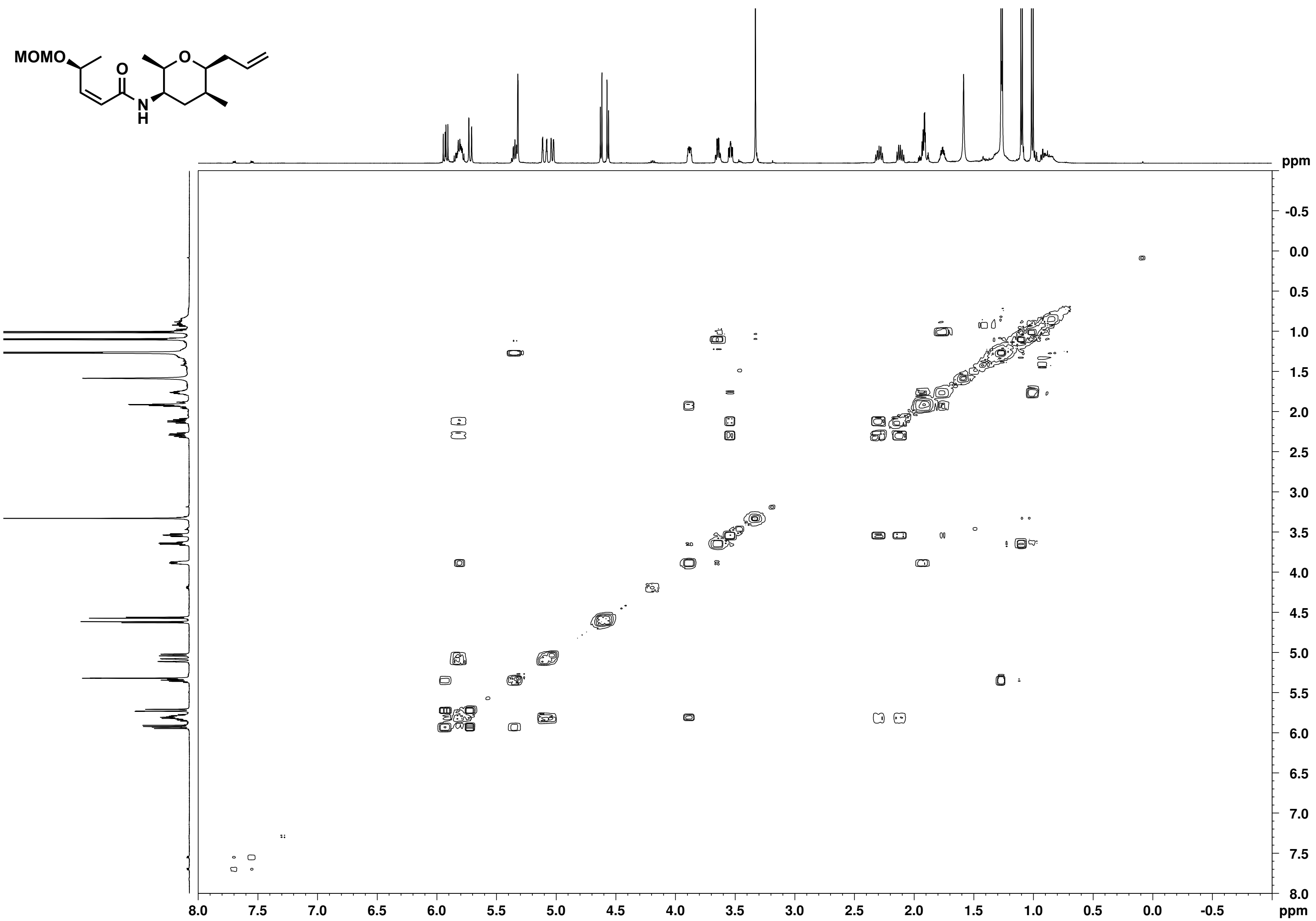

COSY NMR spectrum of (*S*,*Z*)-*N*-((2*R*,3*R*,5*S*,6*S*)-6-allyl-2,5-dimethyltetrahydro-2*H*-pyran-3-yl)-4-(methoxymethoxy)pent-2-enamide **8** (500 MHz, CD_2Cl_2, 293K)

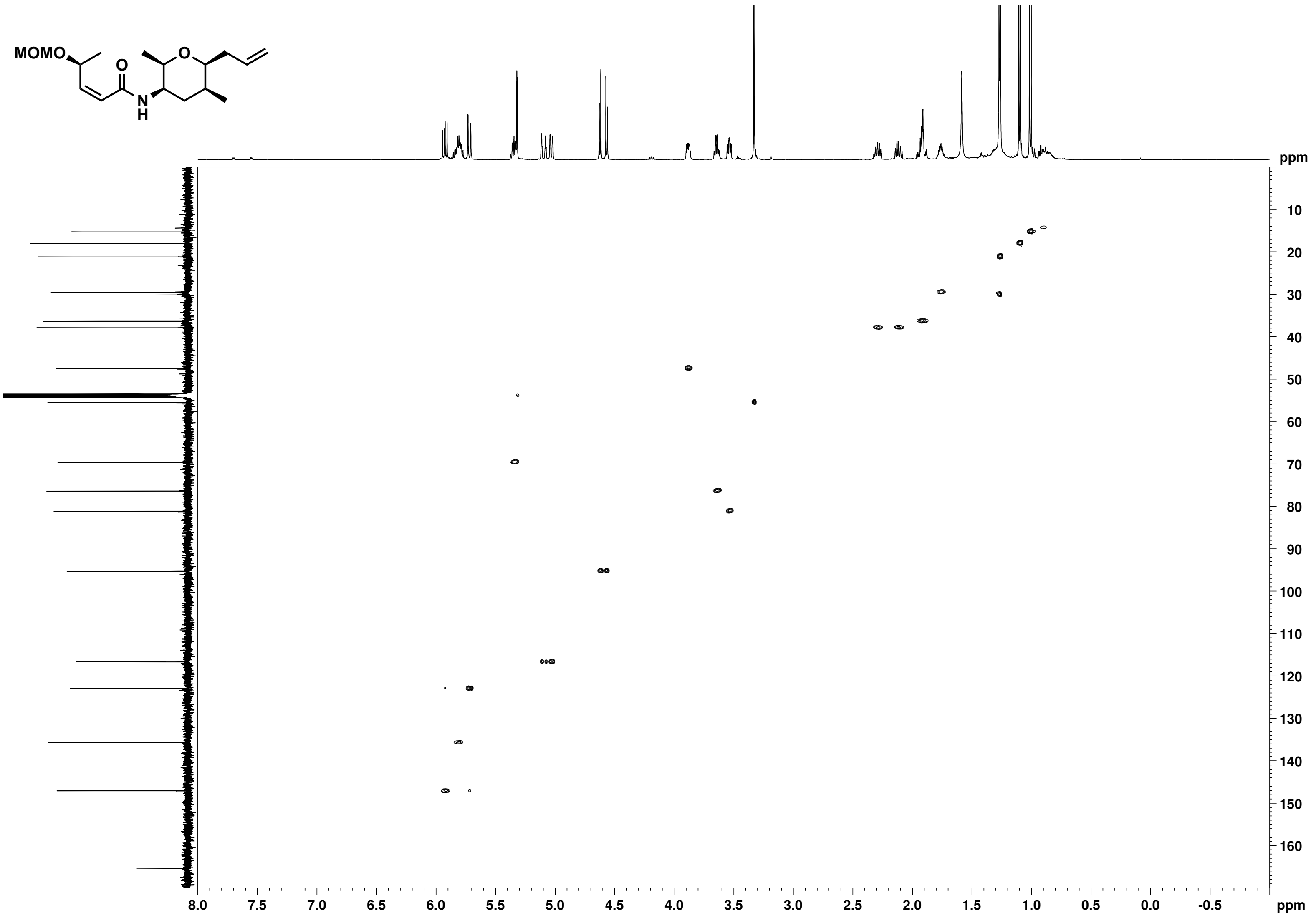

HSQC NMR spectrum of (*S*,*Z*)-*N*-((2*R*,3*R*,5*S*,6*S*)-6-allyl-2,5-dimethyltetrahydro-2*H*-pyran-3-yl)-4-(methoxymethoxy)pent-2-enamide **8** (500 MHz, CD_2Cl_2, 293K)

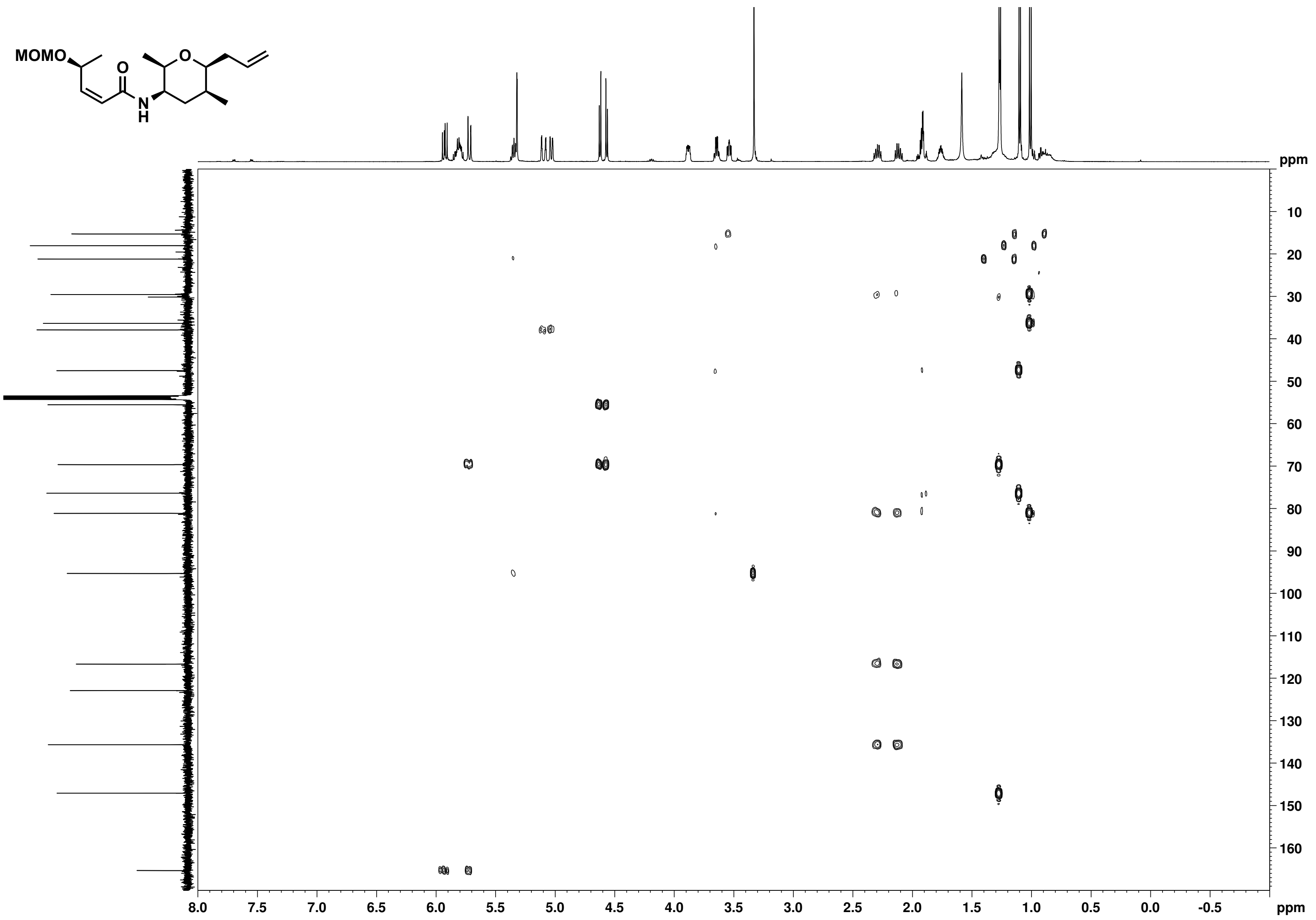

HMBC NMR spectrum of (*S*,*Z*)-*N*-((2*R*,3*R*,5*S*,6*S*)-6-allyl-2,5-dimethyltetrahydro-2*H*-pyran-3-yl)-4-(methoxymethoxy)pent-2-en-amide **8** (500 MHz, CD_2Cl_2, 293K)

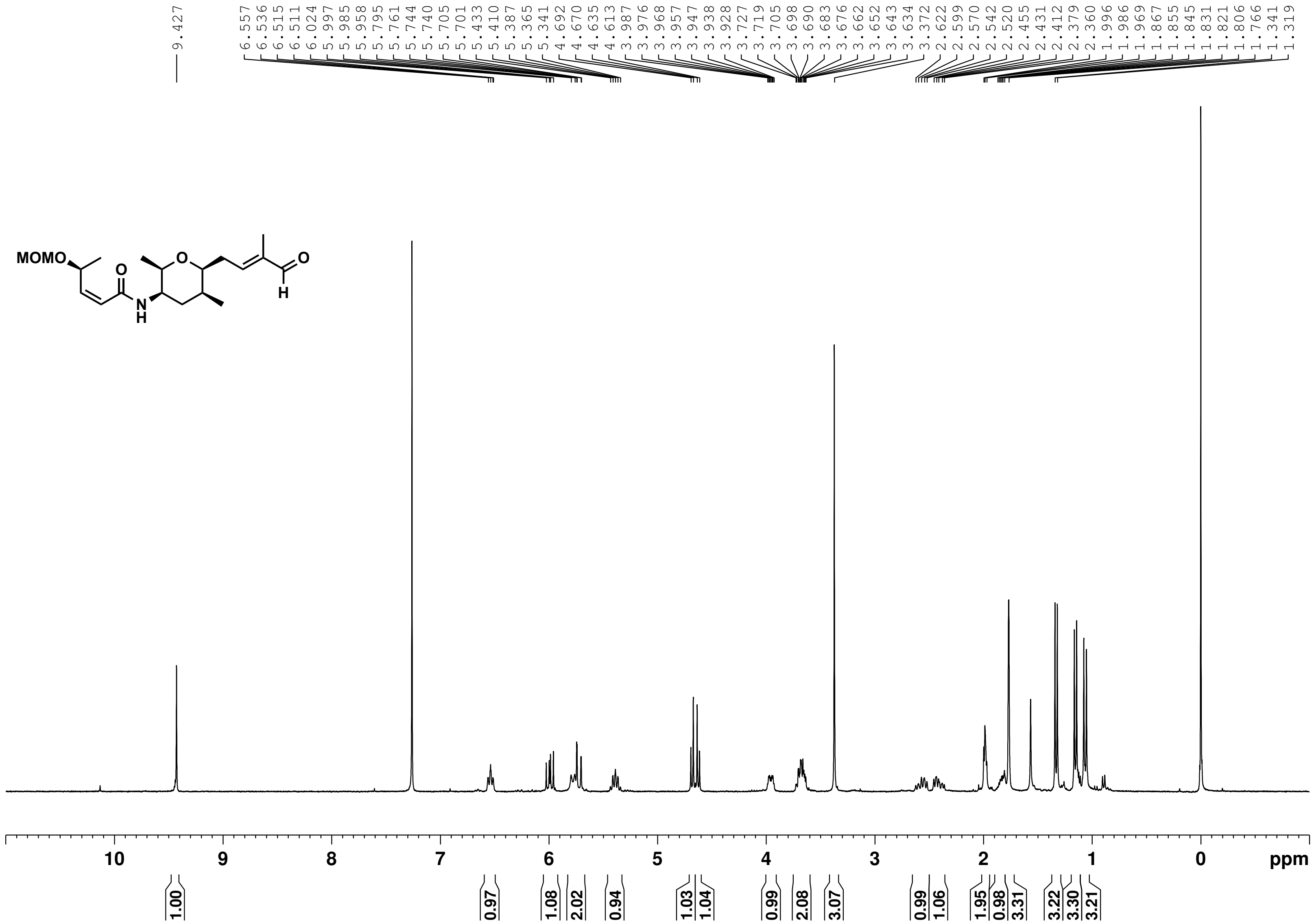

^{1}H NMR spectrum of (*S*,*Z*)-*N*-((2*R*,3*R*,5*S*,6*S*)-2,5-dimethyl-6-((*E*)-3-methyl-4-oxobut-2-en-1-yl)tetrahydro-2*H*-pyran-3-yl)-4-(methoxymethoxy)pent-2-enamide **9** (300 MHz, $CDCl_3$, 293K)

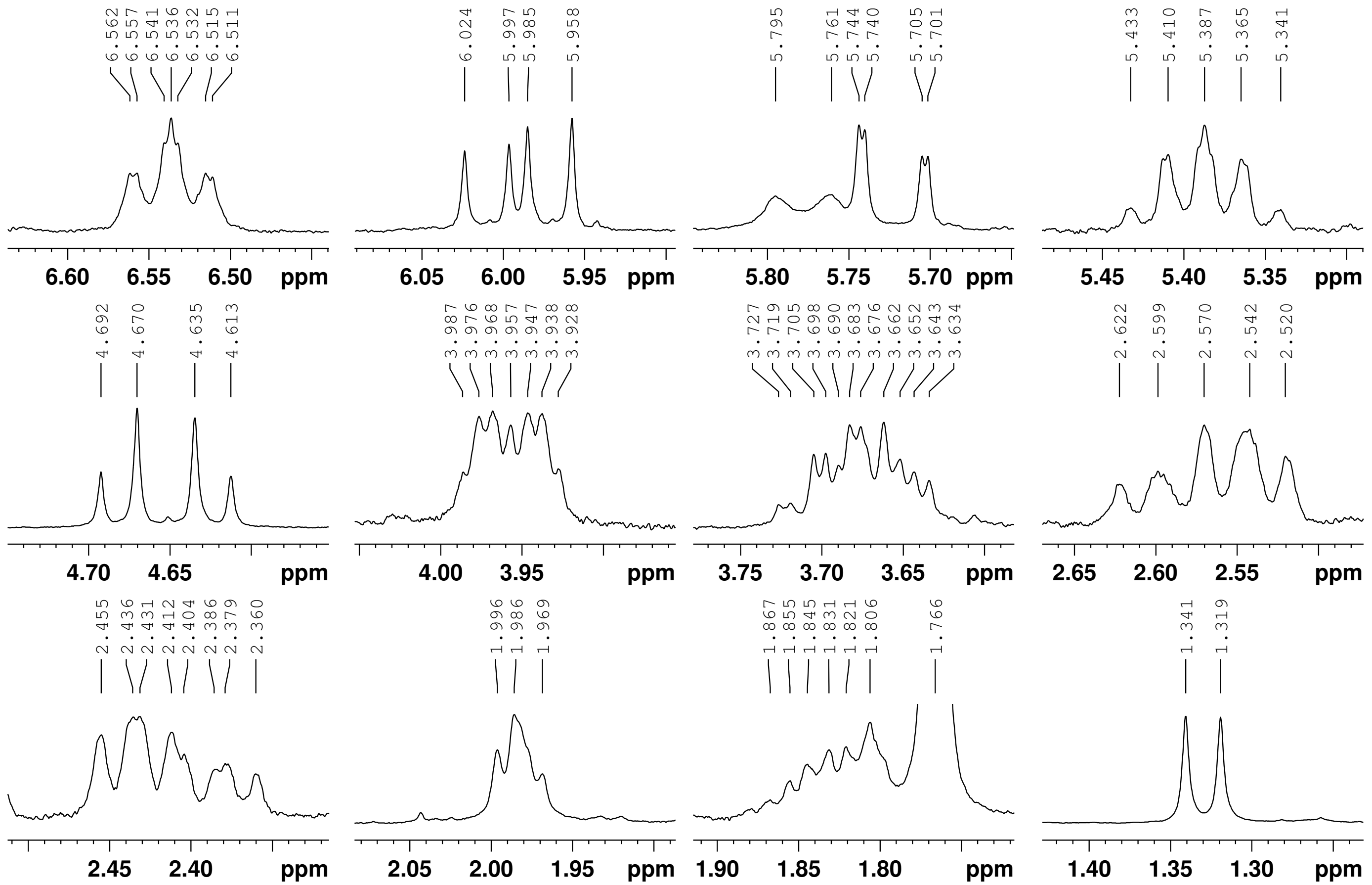

^{1}H NMR spectrum of (*S*,*Z*)-*N*-((2*R*,3*R*,5*S*,6*S*)-2,5-dimethyl-6-((*E*)-3-methyl-4-oxobut-2-en-1-yl)tetrahydro-2*H*-pyran-3-yl)-4-(methoxymethoxy)pent-2-enamide **9** (300 MHz, $CDCl_3$, 293K)

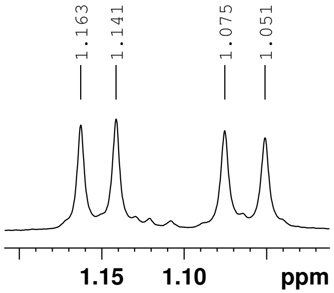

^{1}H NMR spectrum of (*S*,*Z*)-*N*-((2*R*,3*R*,5*S*,6*S*)-2,5-dimethyl-6-((*E*)-3-methyl-4-oxobut-2-en-1-yl)tetrahydro-2*H*-pyran-3-yl)-4-(methoxymethoxy)pent-2-enamide **9** (300 MHz, $CDCl_3$, 293K)

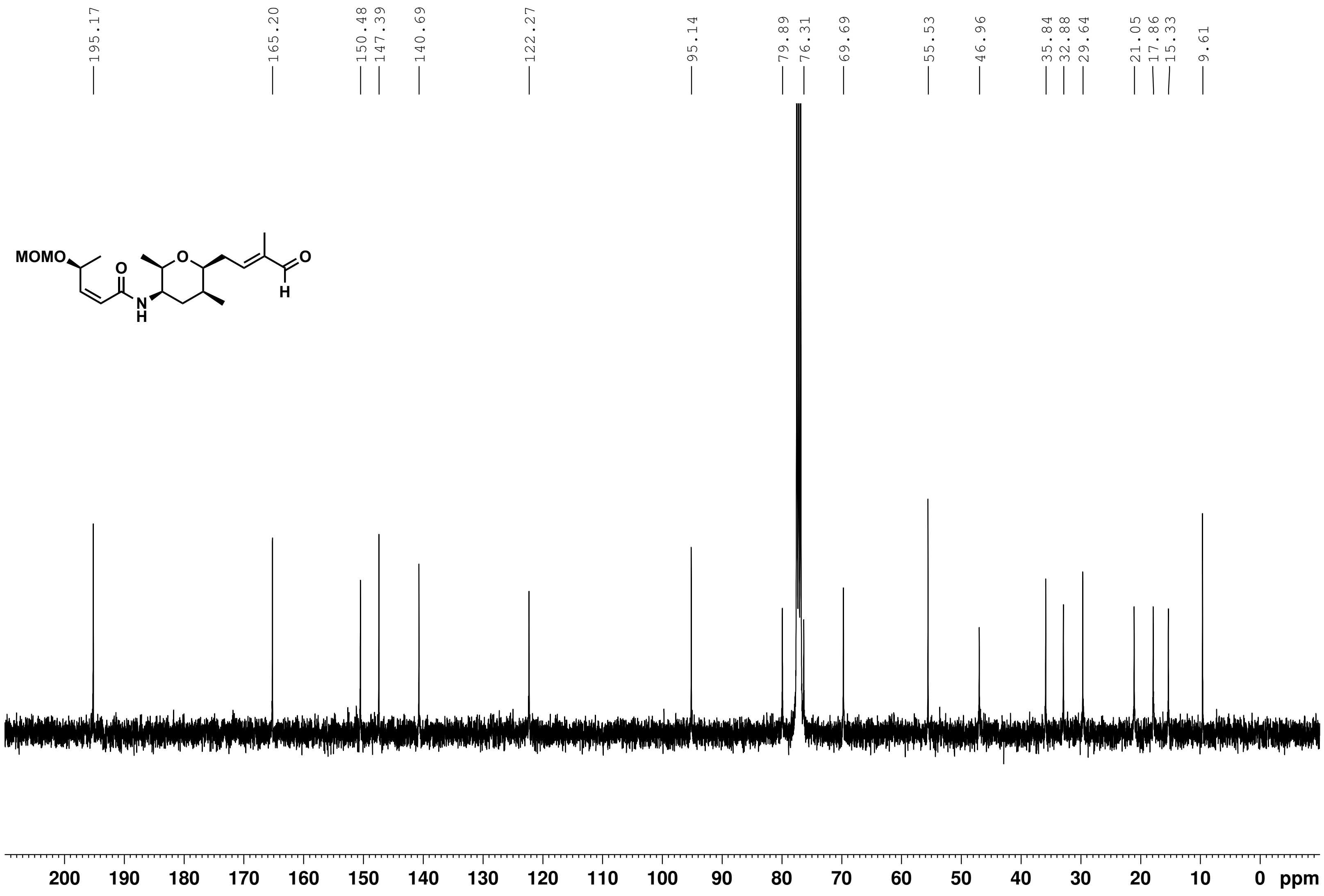

^{13}C NMR spectrum of (*S*,*Z*)-*N*-((2*R*,3*R*,5*S*,6*S*)-2,5-dimethyl-6-((*E*)-3-methyl-4-oxobut-2-en-1-yl)tetrahydro-2*H*-pyran-3-yl)-4-(methoxymethoxy)pent-2-enamide **9** (100 MHz, $CDCl_3$, 293K)

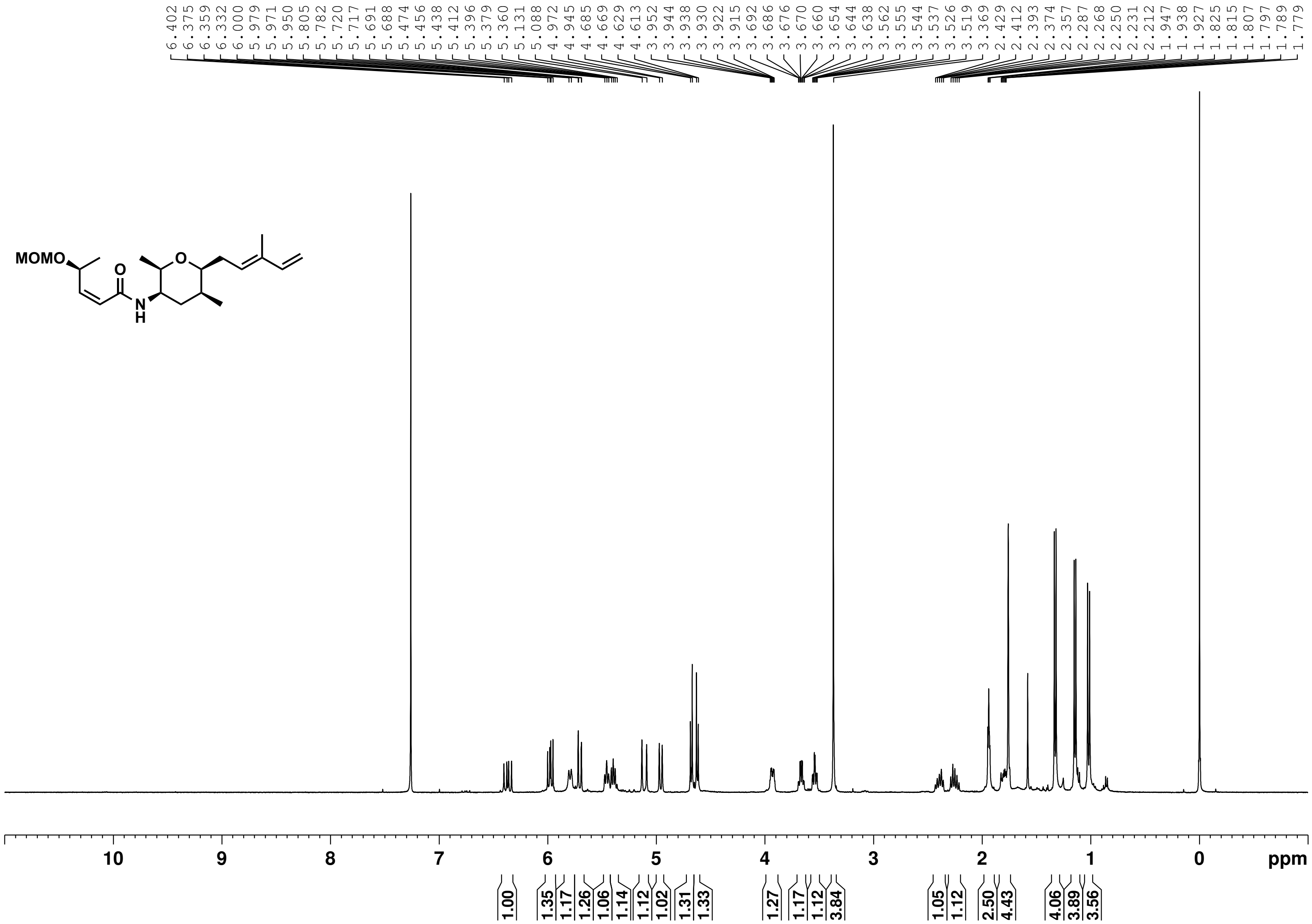

^{1}H NMR spectrum of (*S*,*Z*)-*N*-((2*R*,3*R*,5*S*,6*S*)-2,5-dimethyl-6-((*E*)-3-methylpenta-2,4-dien-1-yl)tetrahydro-2*H*-pyran-3-yl)-4-(methoxymethoxy)pent-2-enamide **10** (400 MHz, $CDCl_3$, 293K)

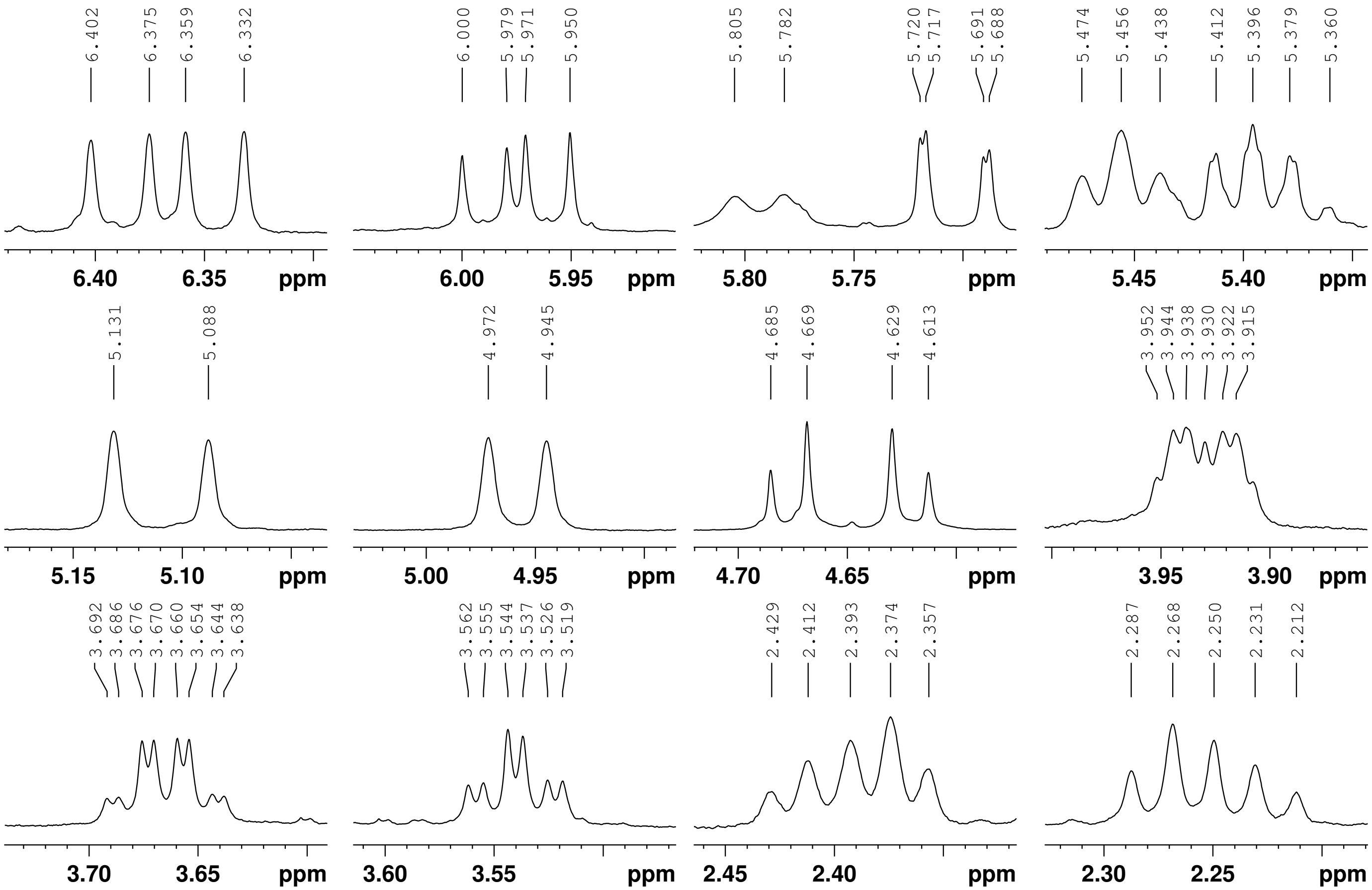

^{1}H NMR spectrum of (*S*,*Z*)-*N*-((2*R*,3*R*,5*S*,6*S*)-2,5-dimethyl-6-((*E*)-3-methylpenta-2,4-dien-1-yl)tetrahydro-2*H*-pyran-3-yl)-4-(methoxymethoxy)pent-2-enamide **10** (400 MHz, $CDCl_3$, 293K)

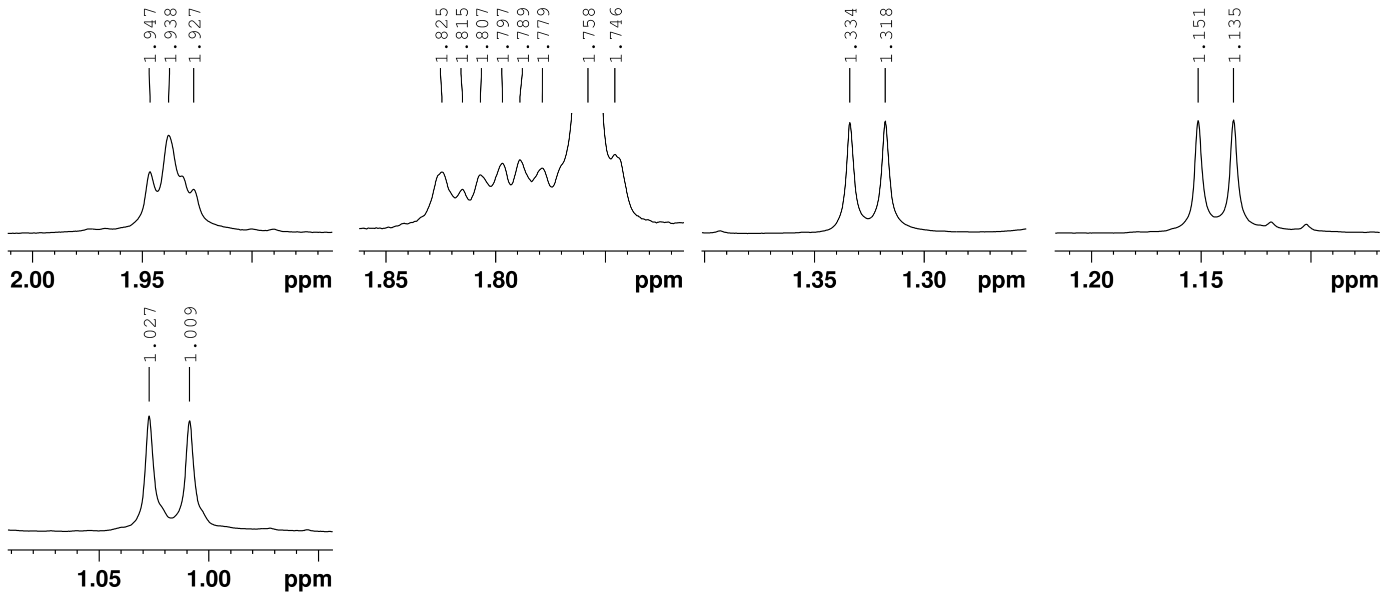

^{1}H NMR spectrum of (*S*,*Z*)-*N*-((2*R*,3*R*,5*S*,6*S*)-2,5-dimethyl-6-((*E*)-3-methylpenta-2,4-dien-1-yl)tetrahydro-2*H*-pyran-3-yl)-4-(methoxymethoxy)pent-2-enamide **10** (400 MHz, $CDCl_3$, 293K)

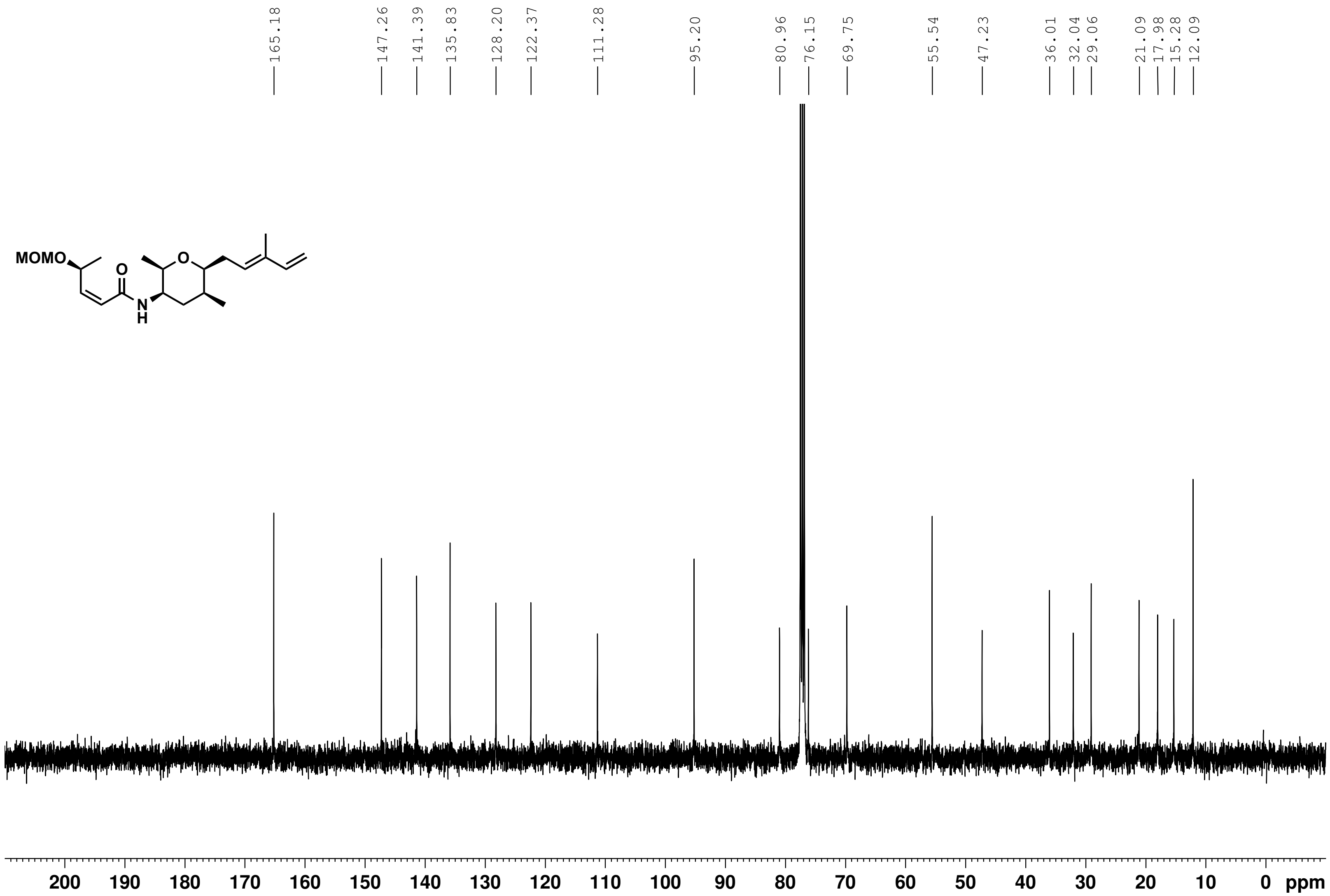

^{13}C NMR spectrum of (*S*,*Z*)-*N*-((2*R*,3*R*,5*S*,6*S*)-2,5-dimethyl-6-((*E*)-3-methylpenta-2,4-dien-1-yl)tetrahydro-2*H*-pyran-3-yl)-4-(methoxymethoxy)pent-2-enamide **10** (100 MHz, $CDCl_3$, 293K)

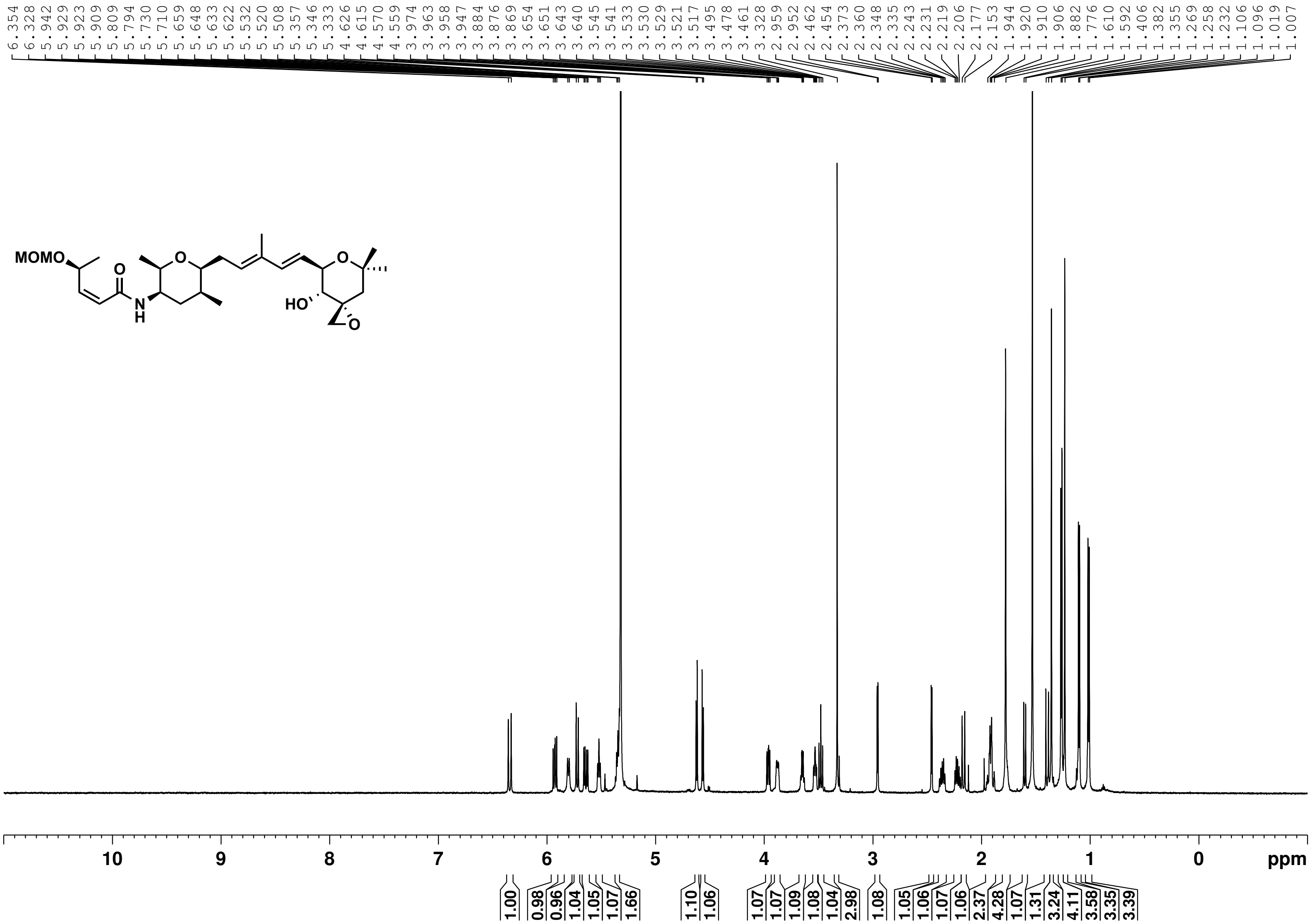

^{1}H NMR spectrum of meayamycin D (600 MHz, CD_2Cl_2, 293K)

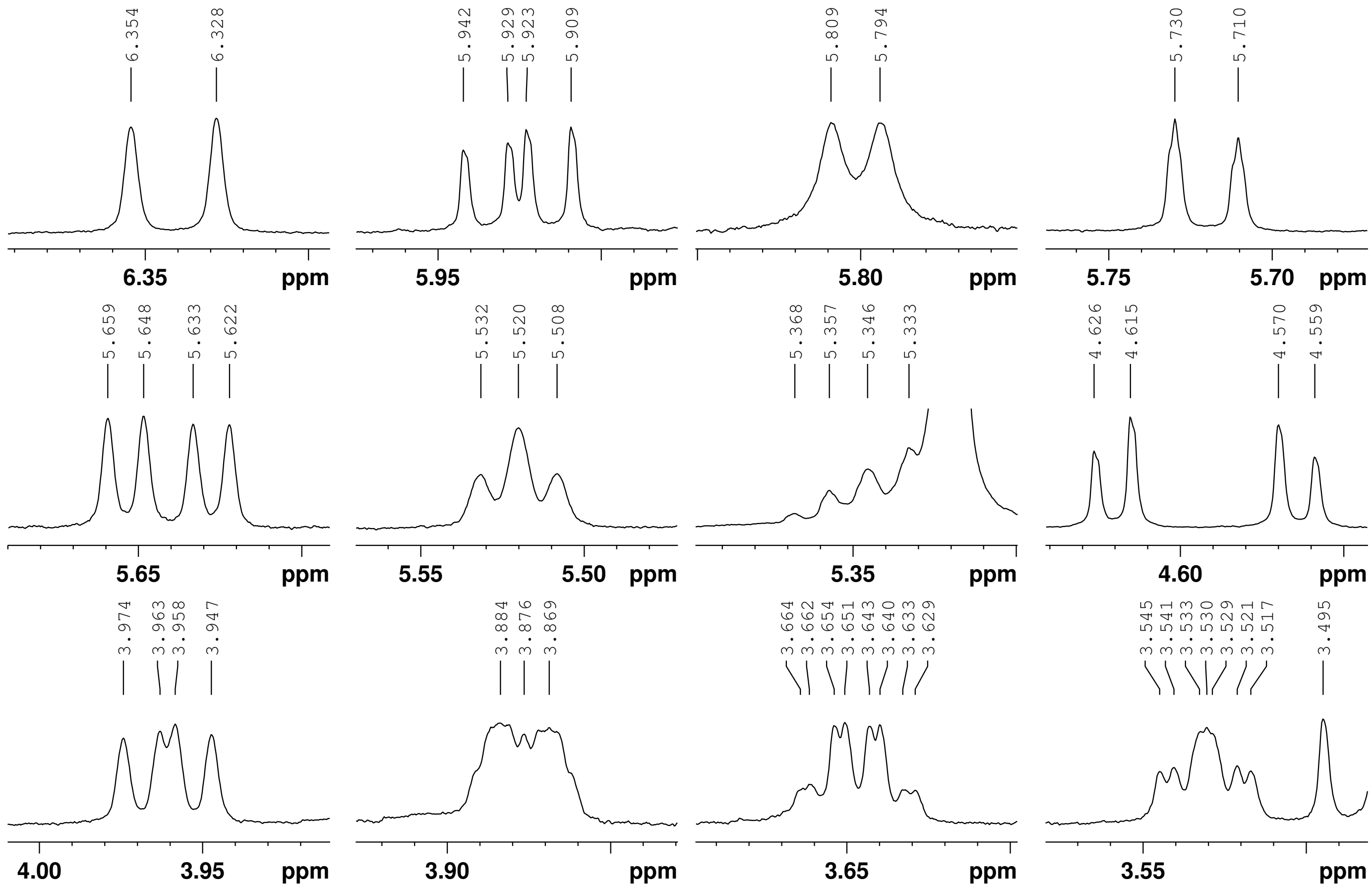

^{1}H NMR spectrum of meayamycin D (600 MHz, CD_2Cl_2, 293K)

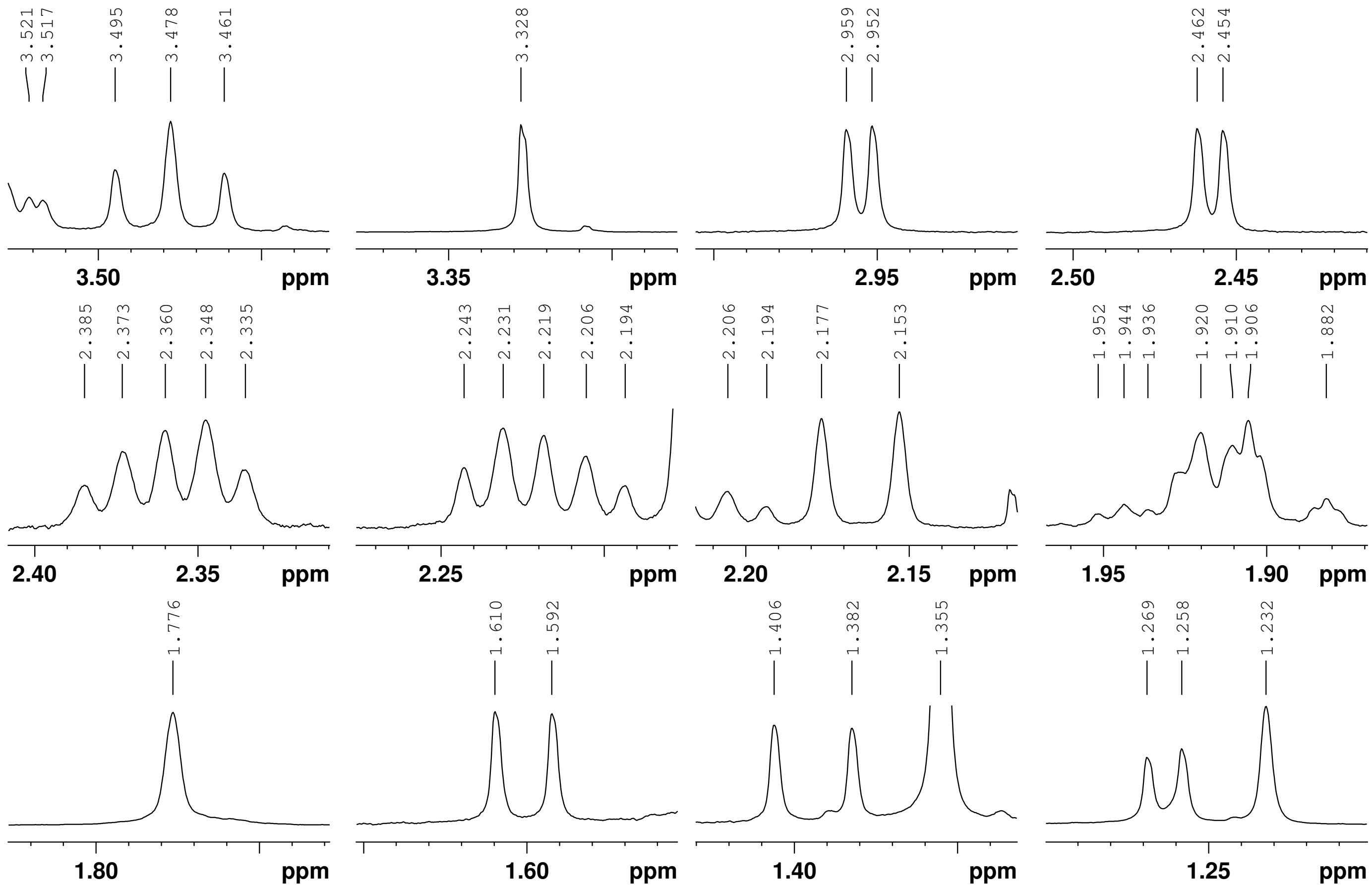

^{1}H NMR spectrum of meayamycin D (600 MHz, CD_2Cl_2, 293K)

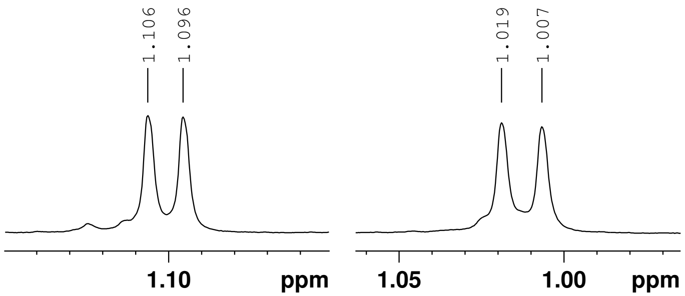

^{1}H NMR spectrum of meayamycin D (600 MHz, CD_2Cl_2, 293K)

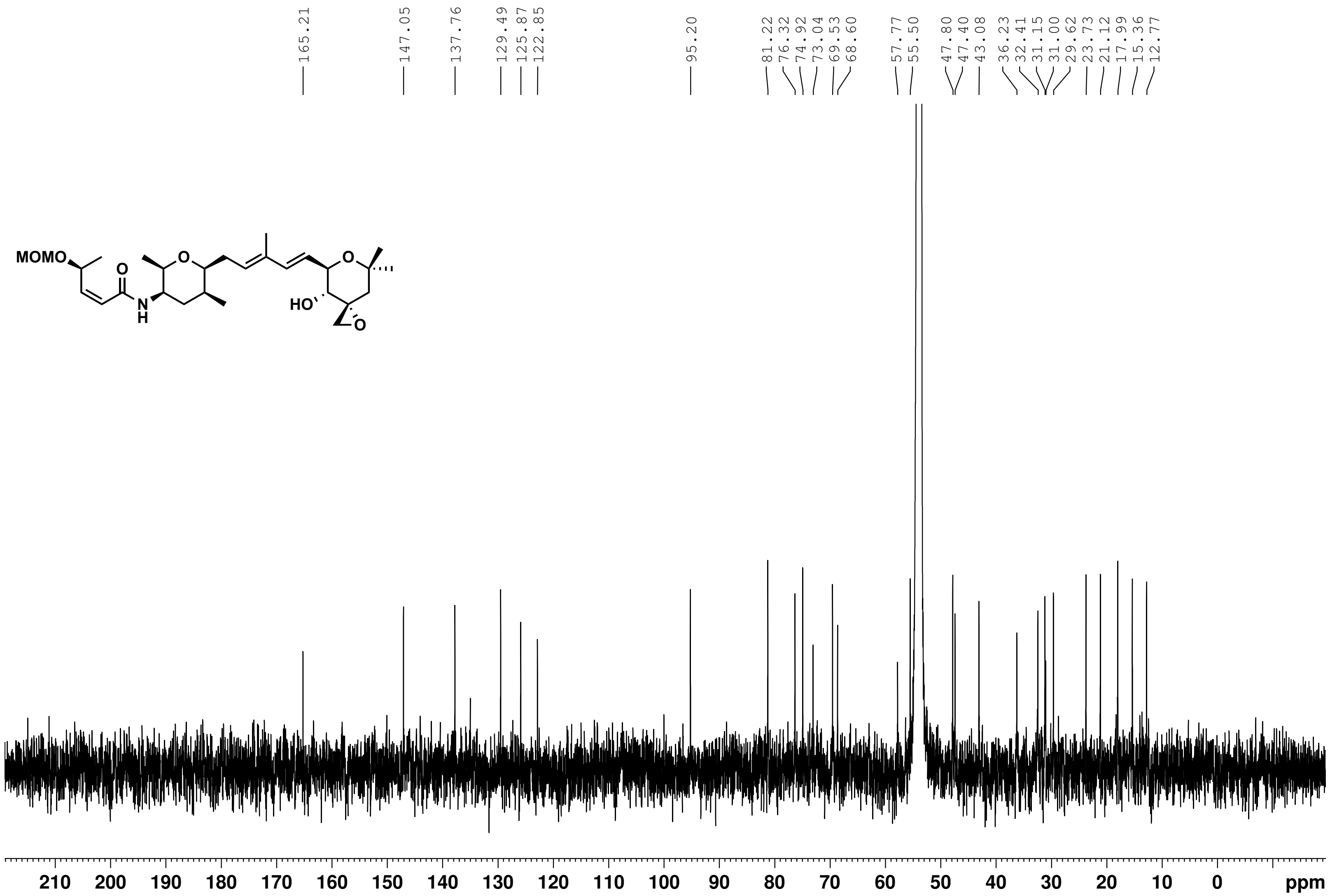

^{13}C NMR spectrum of meayamycin D (150 MHz, CD_2Cl_2, 293K)

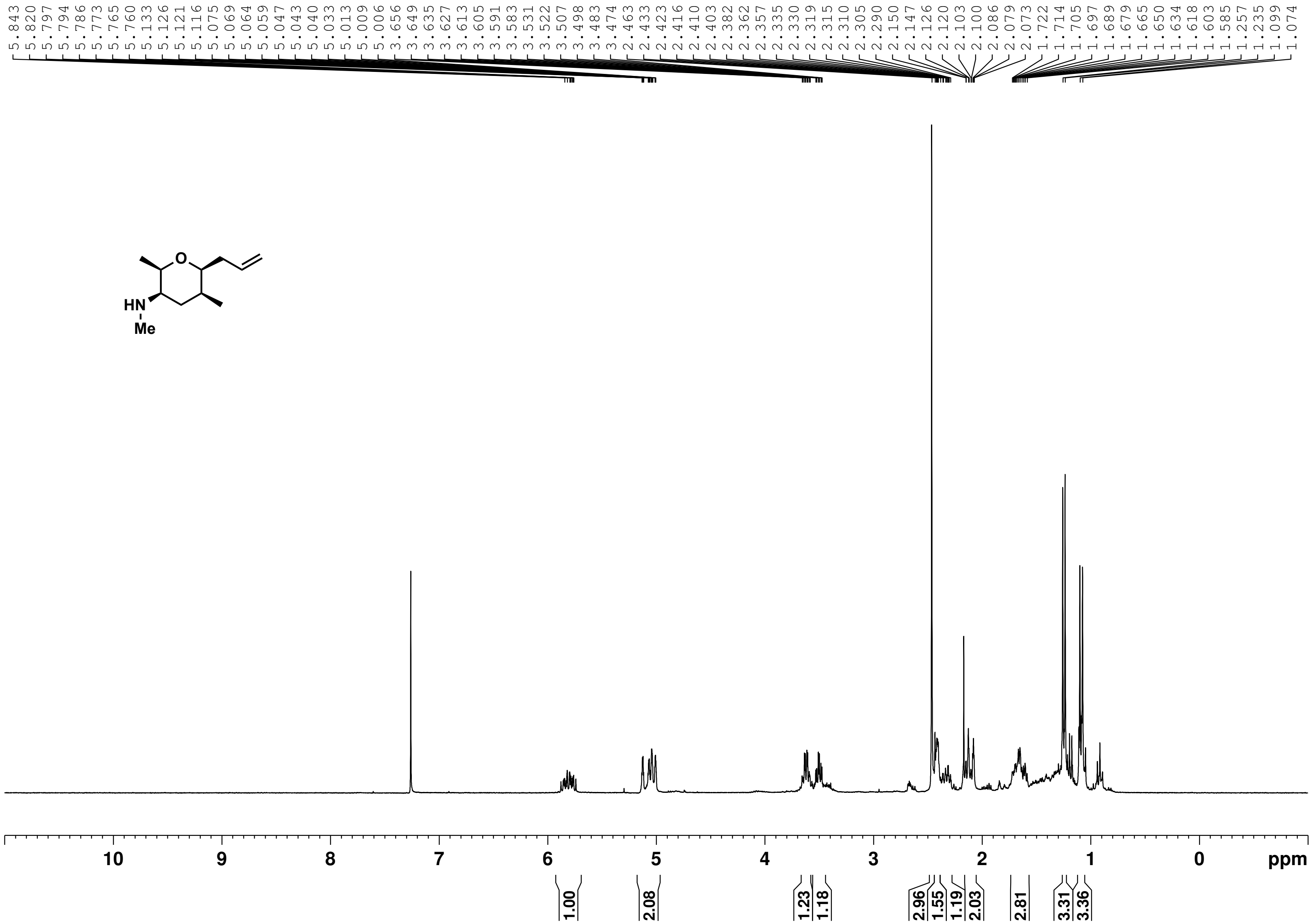

^{1}H NMR spectrum of (2*R*,3*R*,5*S*,6*S*)-6-allyl-*N*,2,5-trimethyltetrahydro-2*H*-pyran-3-amine **16** (300 MHz, 1% CD_3OD in $CDCl_3$, 293K)

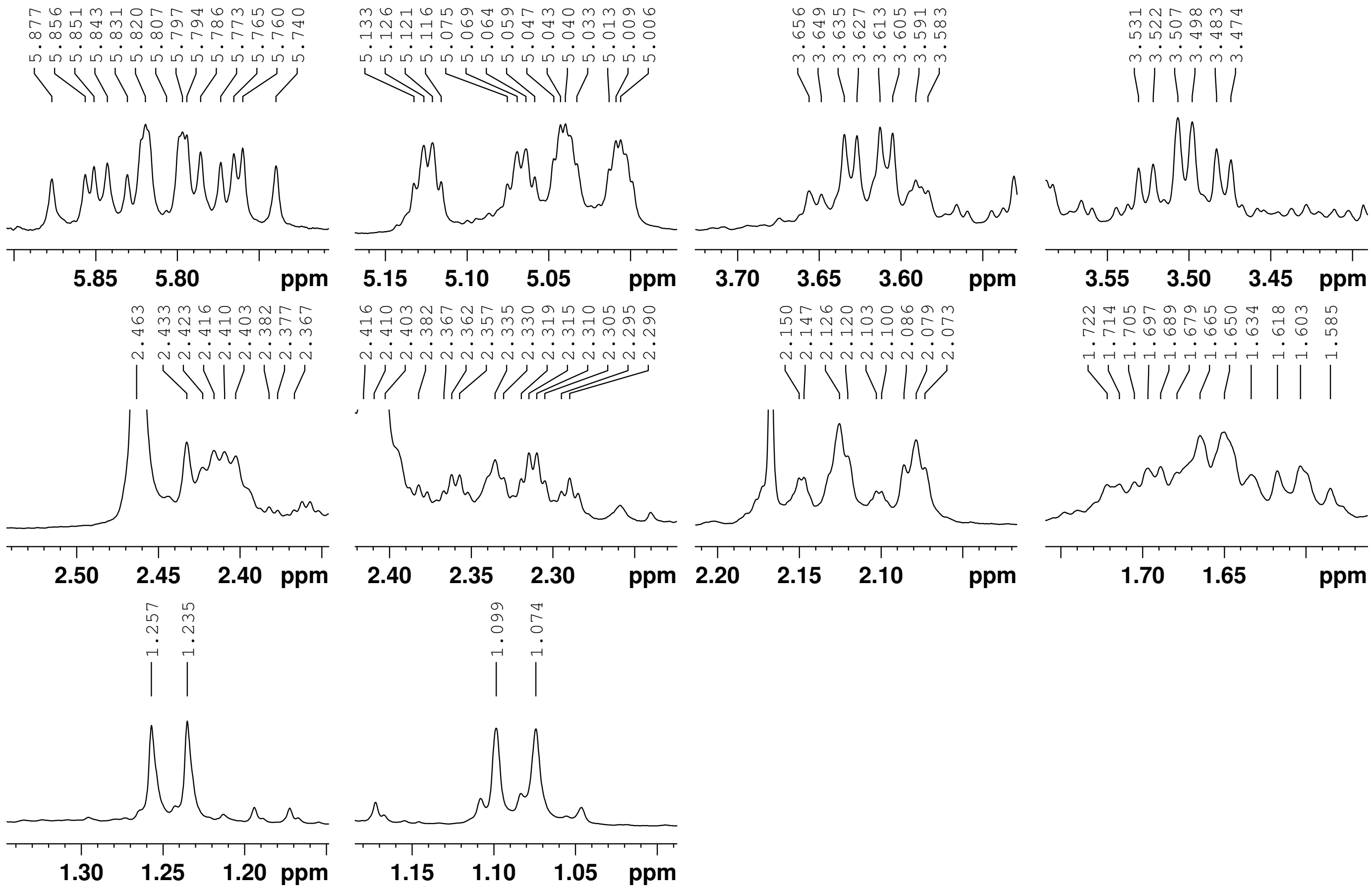

^{1}H NMR spectrum of (2*R*,3*R*,5*S*,6*S*)-6-allyl-*N*,2,5-trimethyltetrahydro-2*H*-pyran-3-amine **16** (300 MHz, 1% CD_3OD in $CDCl_3$, 293K)

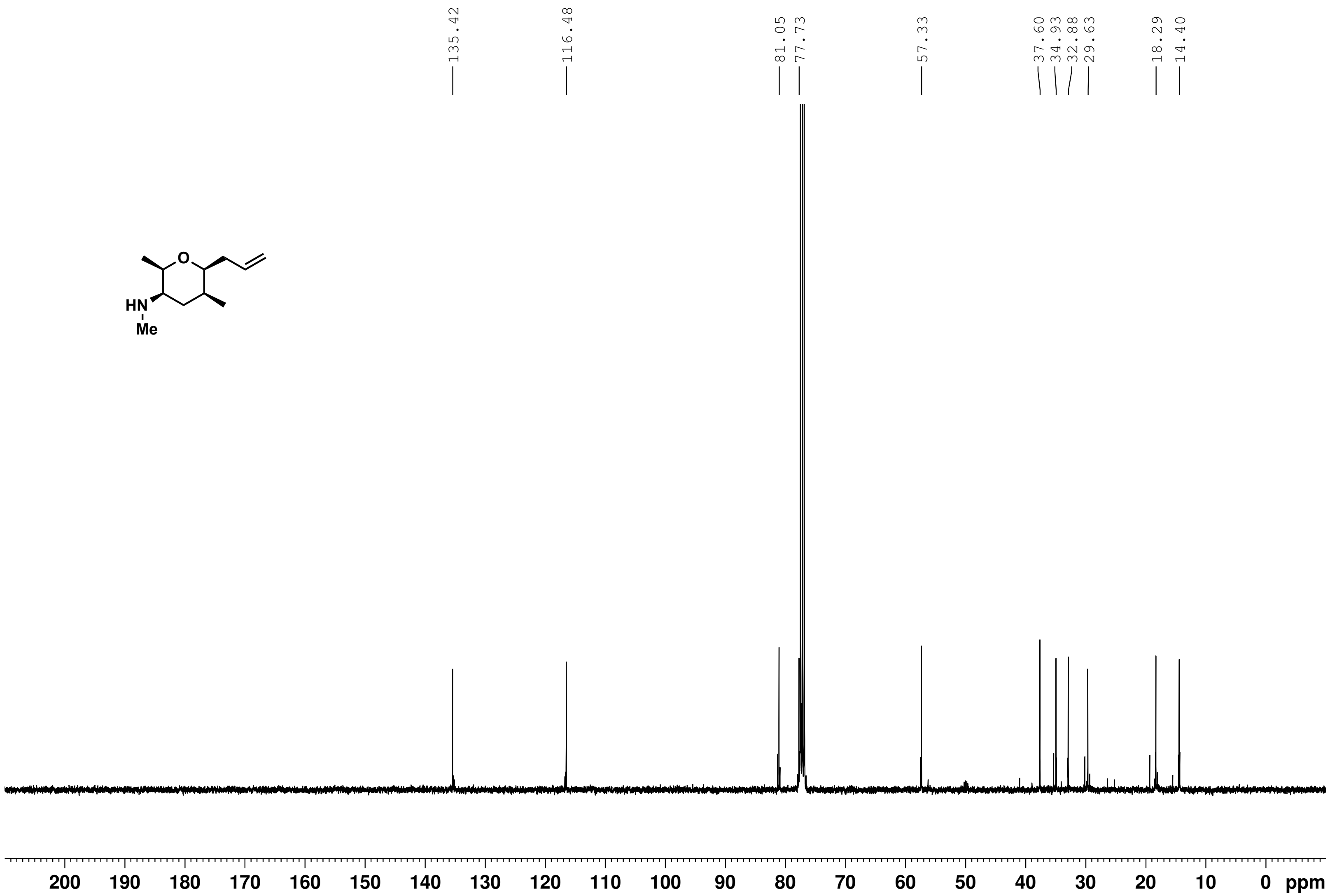

^{13}C NMR spectrum of (2*R*,3*R*,5*S*,6*S*)-6-allyl-*N*,2,5-trimethyltetrahydro-2*H*-pyran-3-amine **16** (100 MHz, 1% CD_3OD in $CDCl_3$, 293K)

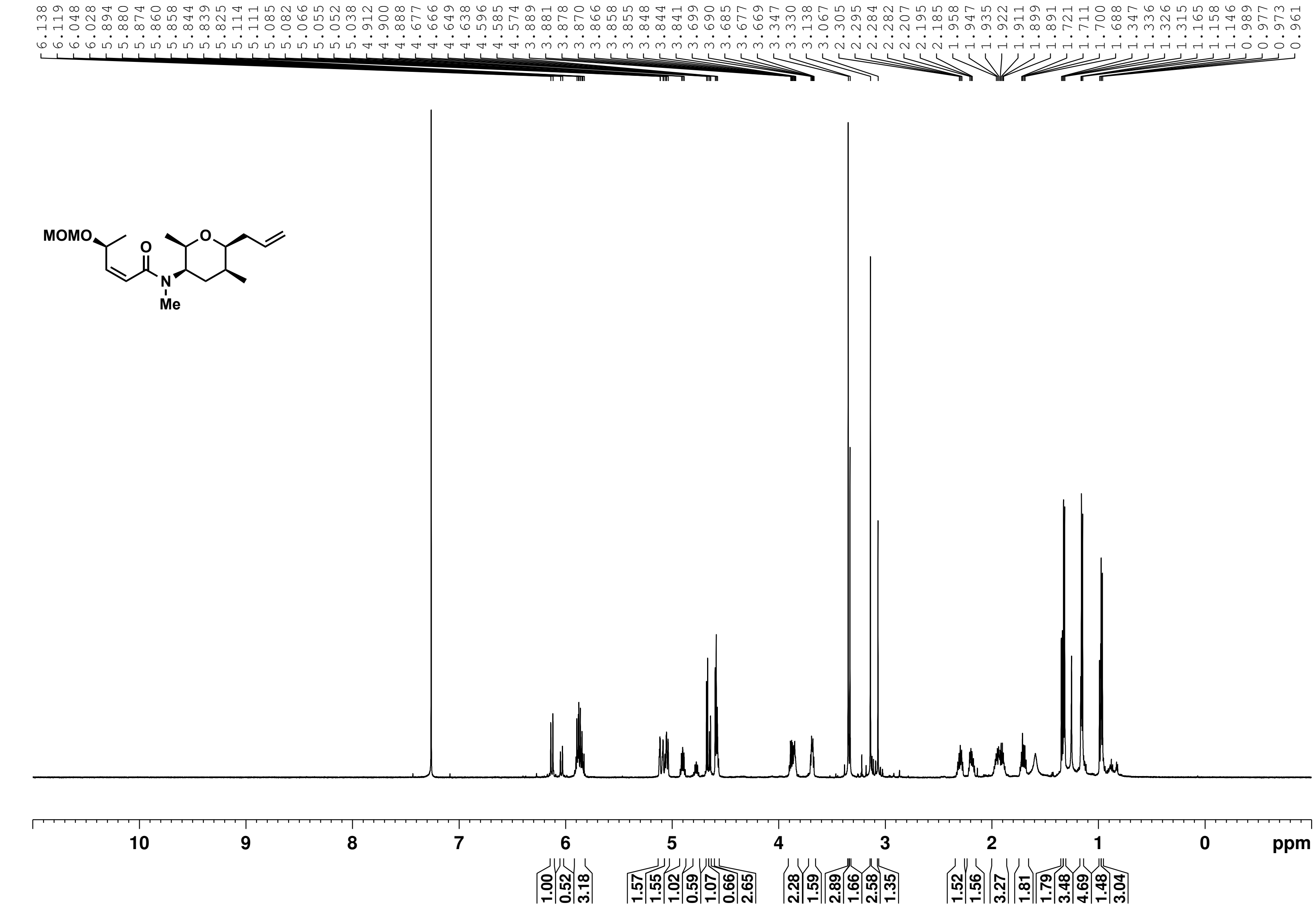

^{1}H NMR spectrum of (*S*,*Z*)-*N*-((2*R*,3*R*,5*S*,6*S*)-6-allyl-2,5-dimethyltetrahydro-2*H*-pyran-3-yl)-4-(methoxymethoxy)-*N*-methylpent-2-enamide **17** (600 MHz, $CDCl_3$, 293K)

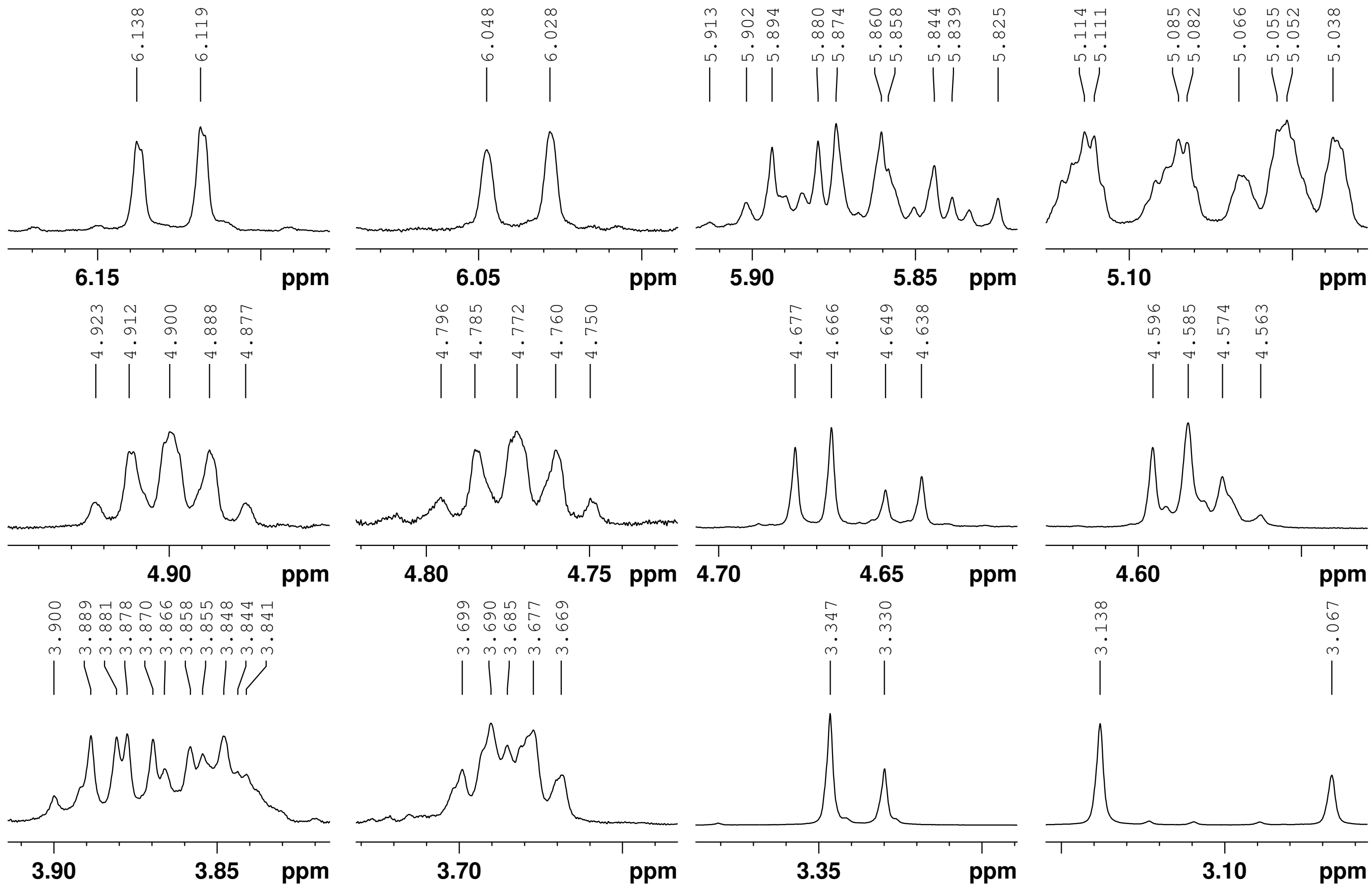

^{1}H NMR spectrum of (*S*,*Z*)-*N*-((2*R*,3*R*,5*S*,6*S*)-6-allyl-2,5-dimethyltetrahydro-2*H*-pyran-3-yl)-4-(methoxymethoxy)-*N*-methylpent-2-enamide **17** (600 MHz, $CDCl_3$, 293K)

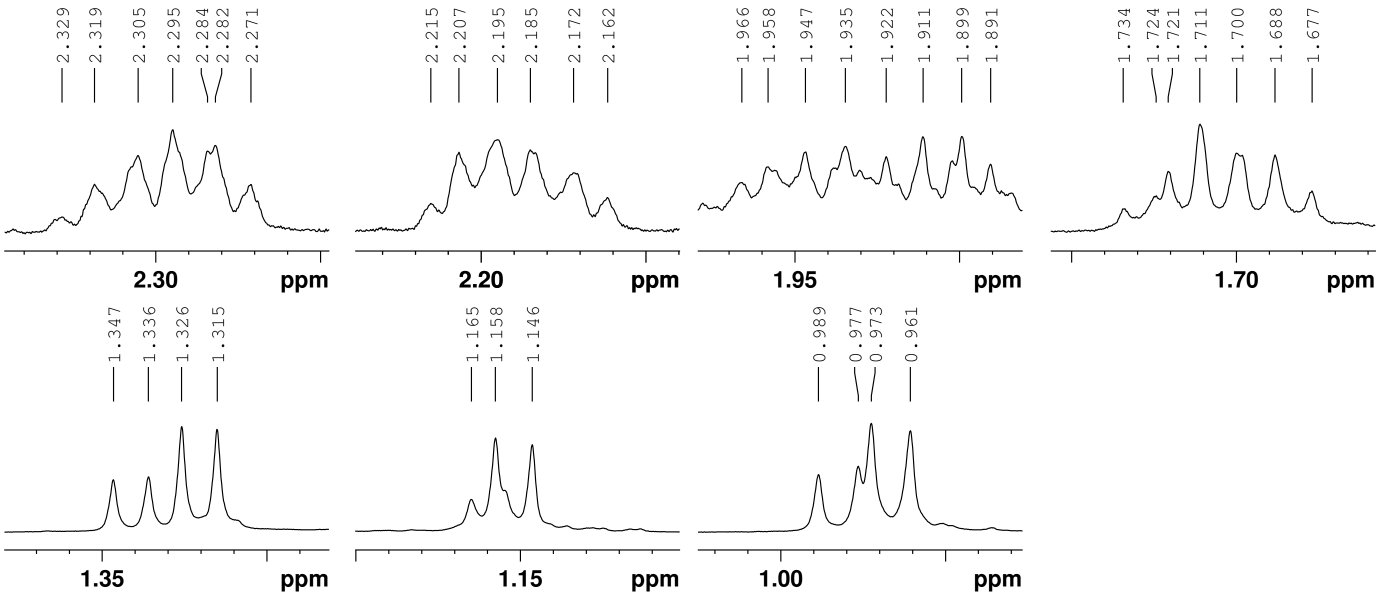

^{1}H NMR spectrum of (*S*,*Z*)-*N*-((2*R*,3*R*,5*S*,6*S*)-6-allyl-2,5-dimethyltetrahydro-2*H*-pyran-3-yl)-4-(methoxymethoxy)-*N*-methylpent-2-enamide **17** (600 MHz, $CDCl_3$, 293K)

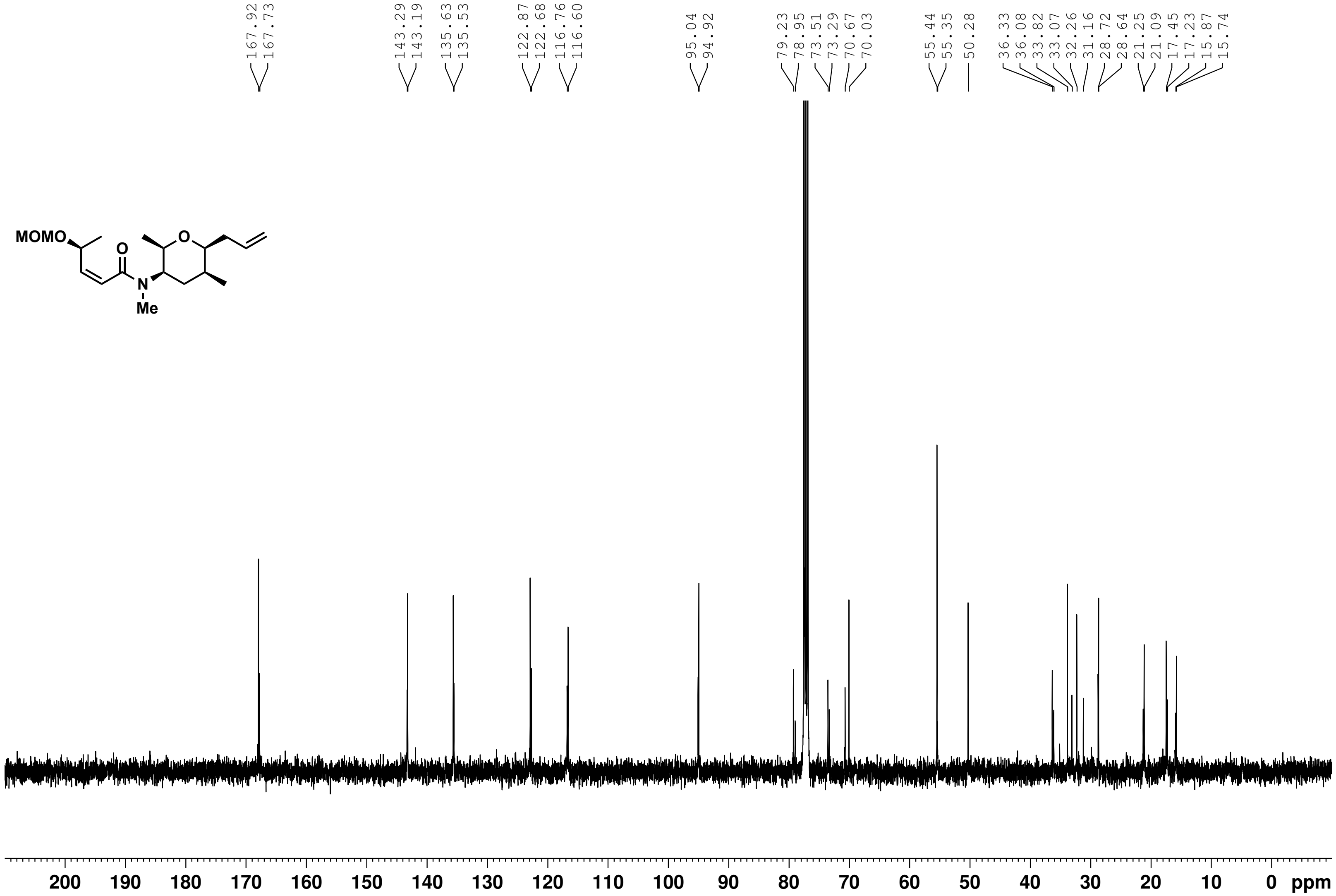

^{13}C NMR spectrum of (*S*,*Z*)-*N*-((2*R*,3*R*,5*S*,6*S*)-6-allyl-2,5-dimethyltetrahydro-2*H*-pyran-3-yl)-4-(methoxymethoxy)-*N*-methylpent-2-enamide **17** (100 MHz, $CDCl_3$, 293K)

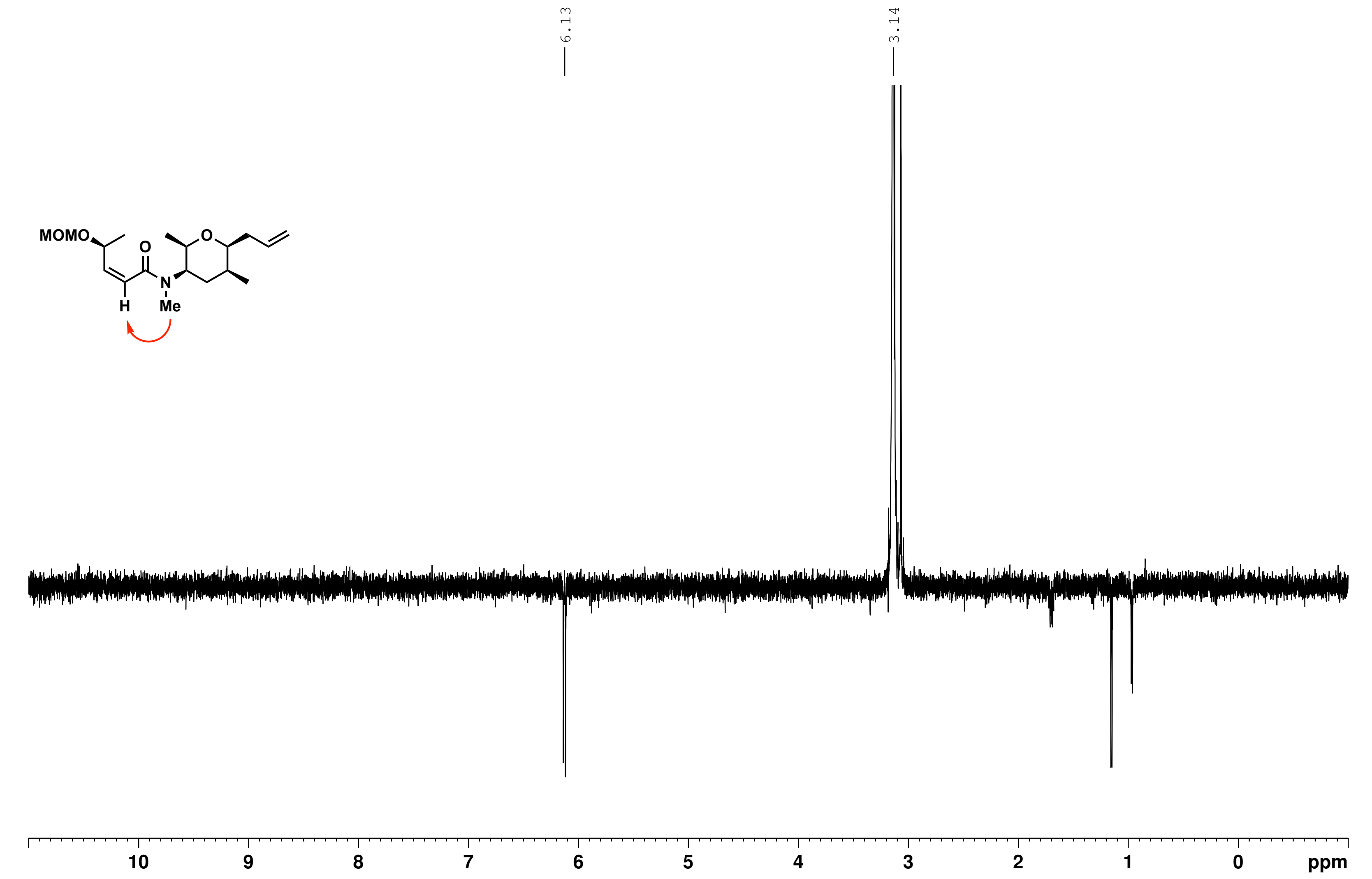

Selective 1D NOESY spectrum of (*S*,*Z*)-*N*-((2*R*,3*R*,5*S*,6*S*)-6-allyl-2,5-dimethyltetrahydro-2*H*-pyran-3-yl)-4-(methoxymethoxy)-*N*-methylpent-2-enamide **17** (600 MHz, $CDCl_3$, 293K, major rotamer)

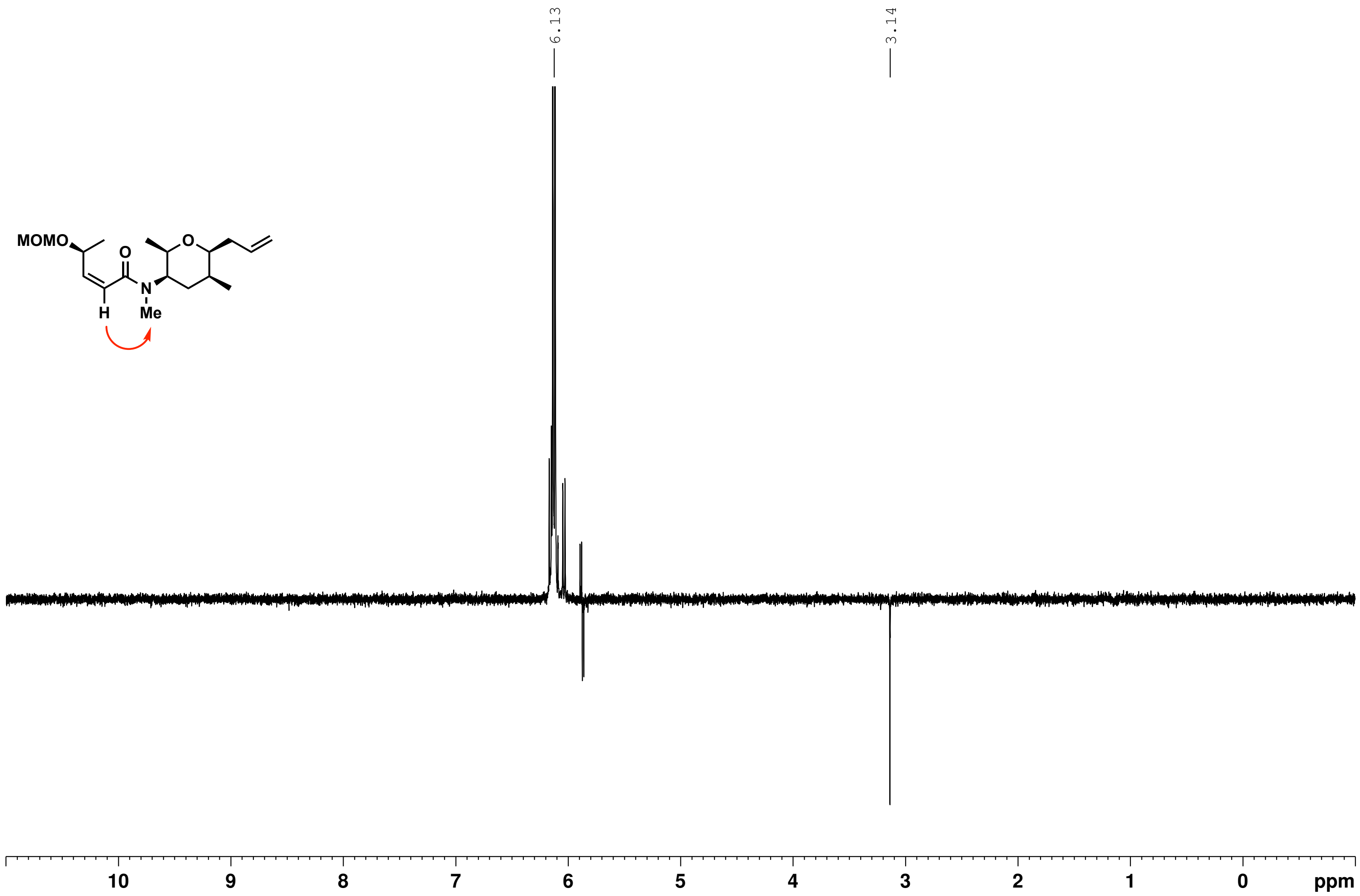

Selective 1D NOESY spectrum of (*S*,*Z*)-*N*-((2*R*,3*R*,5*S*,6*S*)-6-allyl-2,5-dimethyltetrahydro-2*H*-pyran-3-yl)-4-(methoxymethoxy)-*N*-methylpent-2-enamide **17** (600 MHz, $CDCl_3$, 293K, major rotamer)

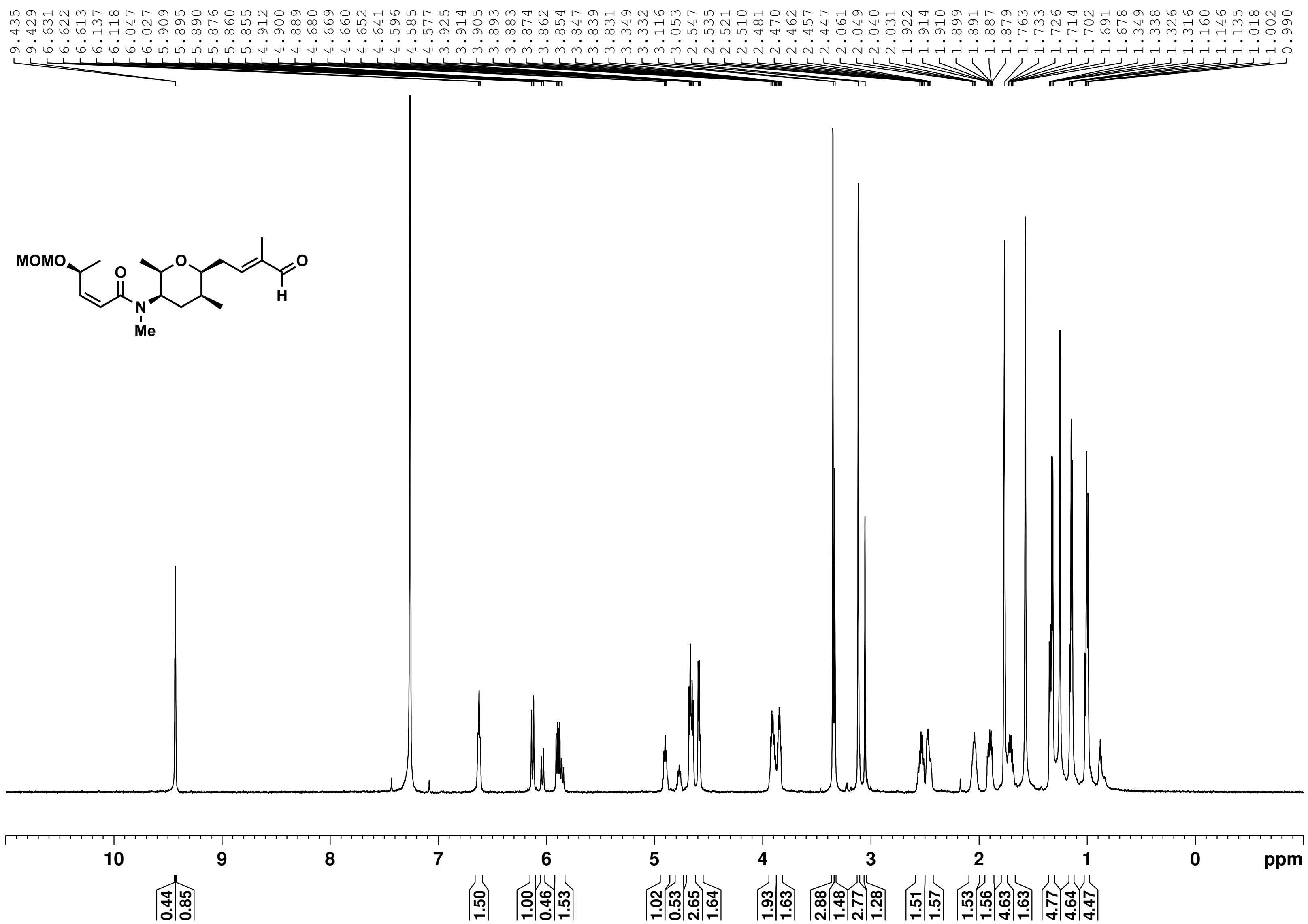

^{1}H NMR spectrum of (*S*,*Z*)-*N*-((2*R*,3*R*,5*S*,6*S*)-2,5-dimethyl-6-((*E*)-3-methyl-4-oxobut-2-en-1-yl)tetrahydro-2*H*-pyran-3-yl)-4-(methoxymethoxy)-*N*-methylpent-2-enamide **18** (600 MHz, $CDCl_3$, 293K)

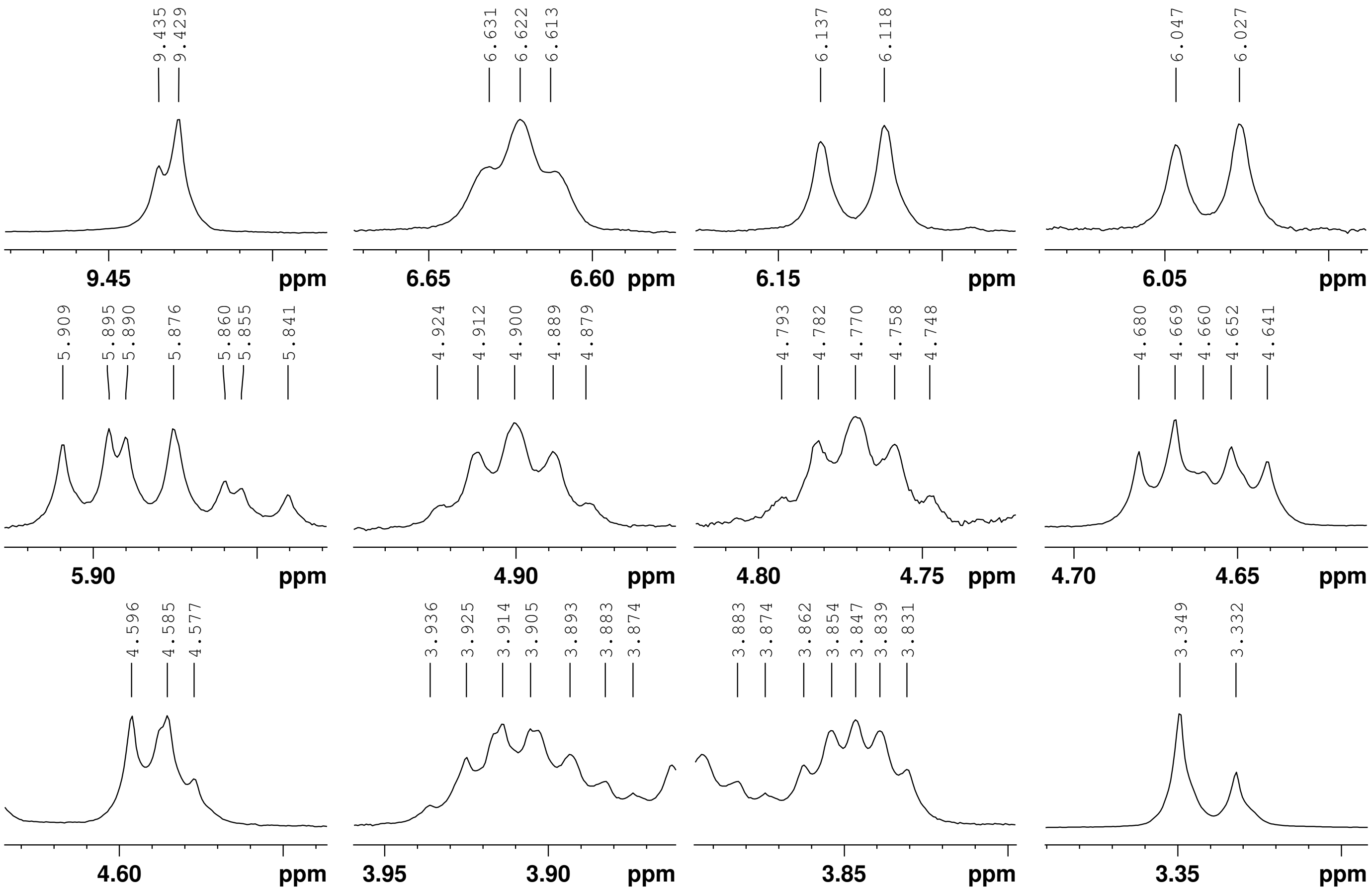

^{1}H NMR spectrum of (*S*,*Z*)-*N*-((2*R*,3*R*,5*S*,6*S*)-2,5-dimethyl-6-((*E*)-3-methyl-4-oxobut-2-en-1-yl)tetrahydro-2*H*-pyran-3-yl)-4-(methoxymethoxy)-*N*-methylpent-2-enamide **18** (600 MHz, $CDCl_3$, 293K)

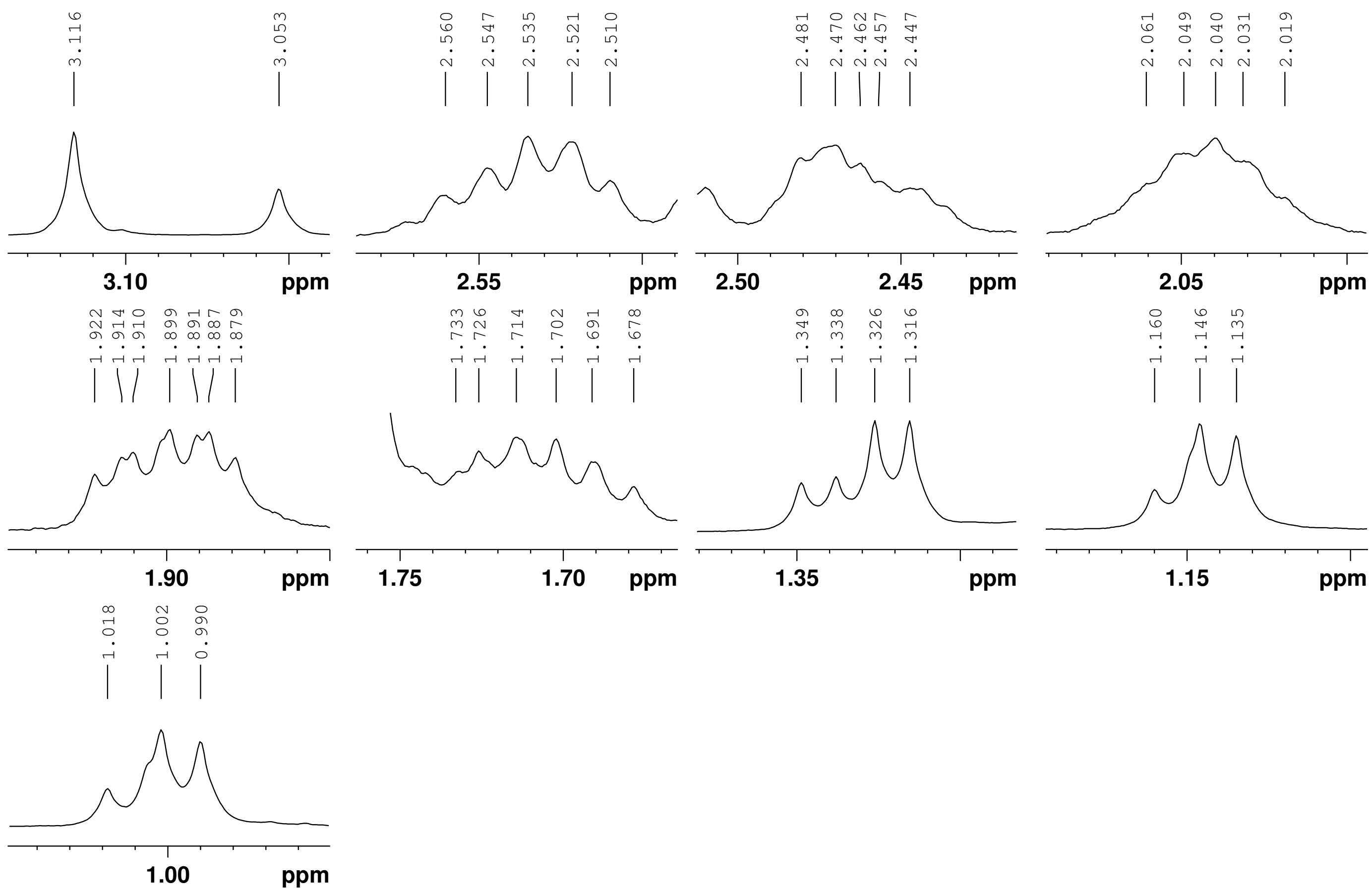

^{1}H NMR spectrum of (*S*,*Z*)-*N*-((2*R*,3*R*,5*S*,6*S*)-2,5-dimethyl-6-((*E*)-3-methyl-4-oxobut-2-en-1-yl)tetrahydro-2*H*-pyran-3-yl)-4-(methoxymethoxy)-*N*-methylpent-2-enamide **18** (600 MHz, $CDCl_3$, 293K)

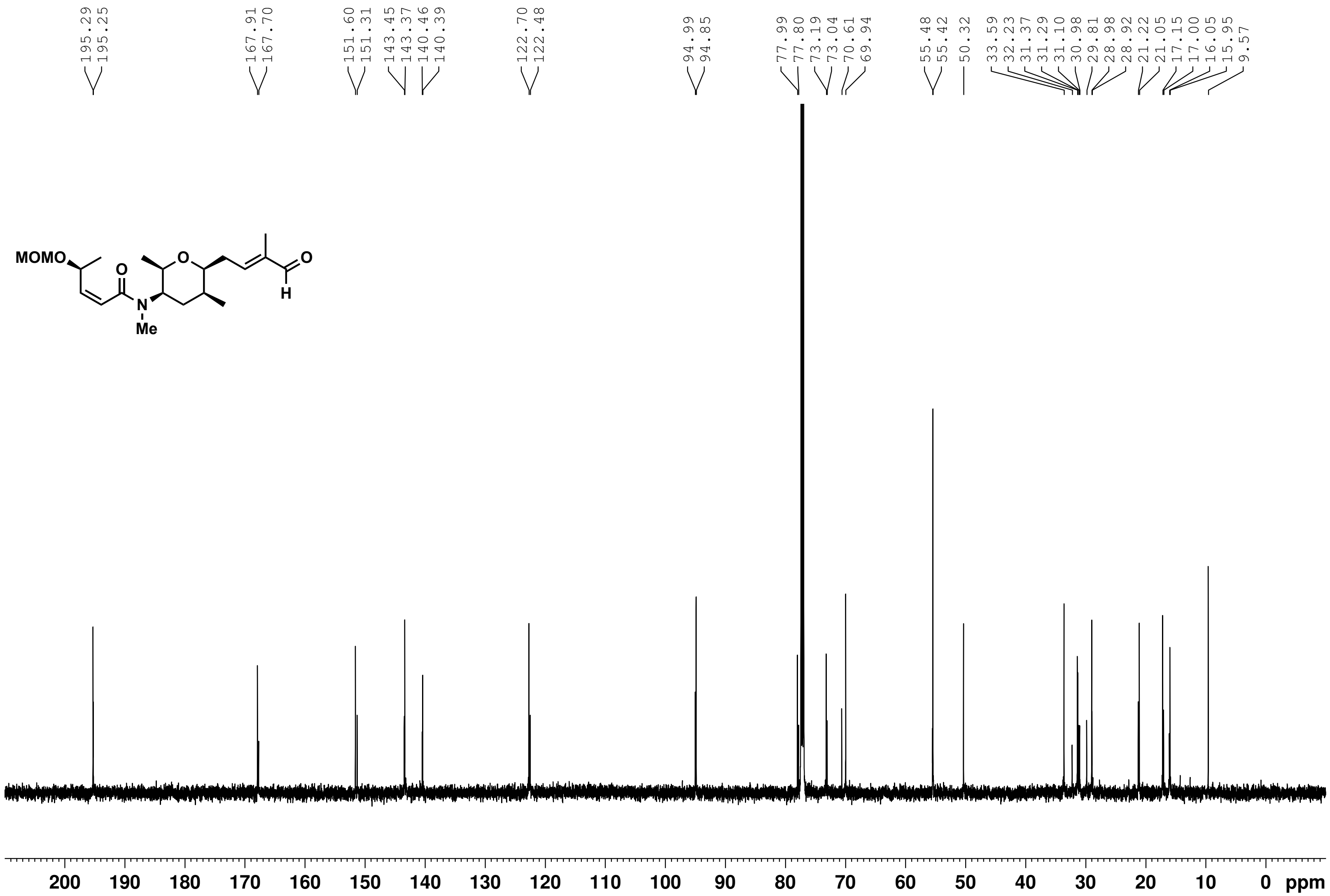

^{13}C NMR spectrum of (*S*,*Z*)-*N*-((2*R*,3*R*,5*S*,6*S*)-2,5-dimethyl-6-((*E*)-3-methyl-4-oxobut-2-en-1-yl)tetrahydro-2*H*-pyran-3-yl)-4-(methoxymethoxy)-*N*-methylpent-2-enamide **18** (150 MHz, $CDCl_3$, 293K)

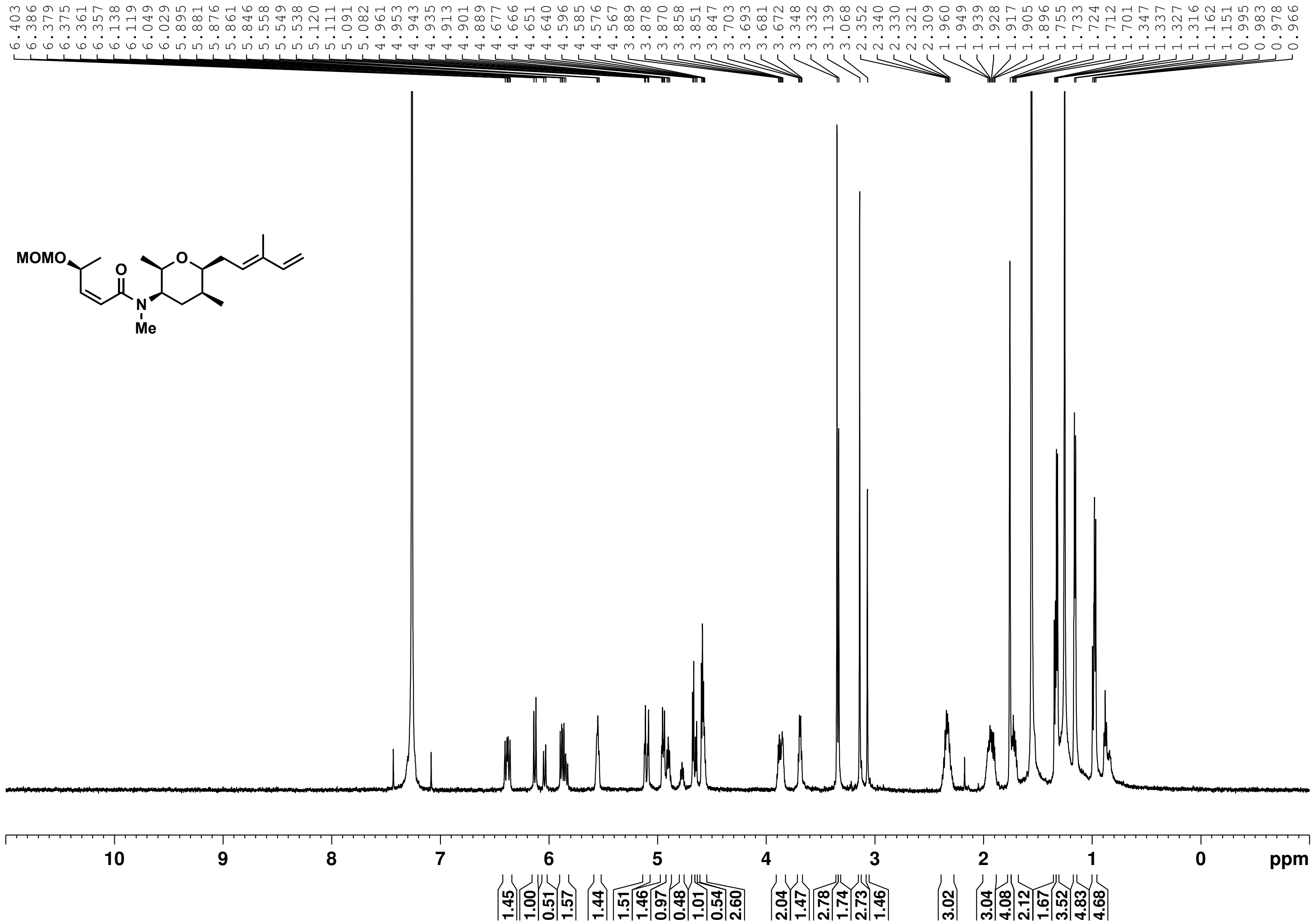

^{1}H NMR spectrum of (*S*,*Z*)-*N*-((2*R*,3*R*,5*S*,6*S*)-2,5-dimethyl-6-((*E*)-3-methylpenta-2,4-dien-1-yl)tetrahydro-2*H*-pyran-3-yl)-4-(methoxymethoxy)-*N*-methylpent-2-enamide **19** (600 MHz, $CDCl_3$, 293K)

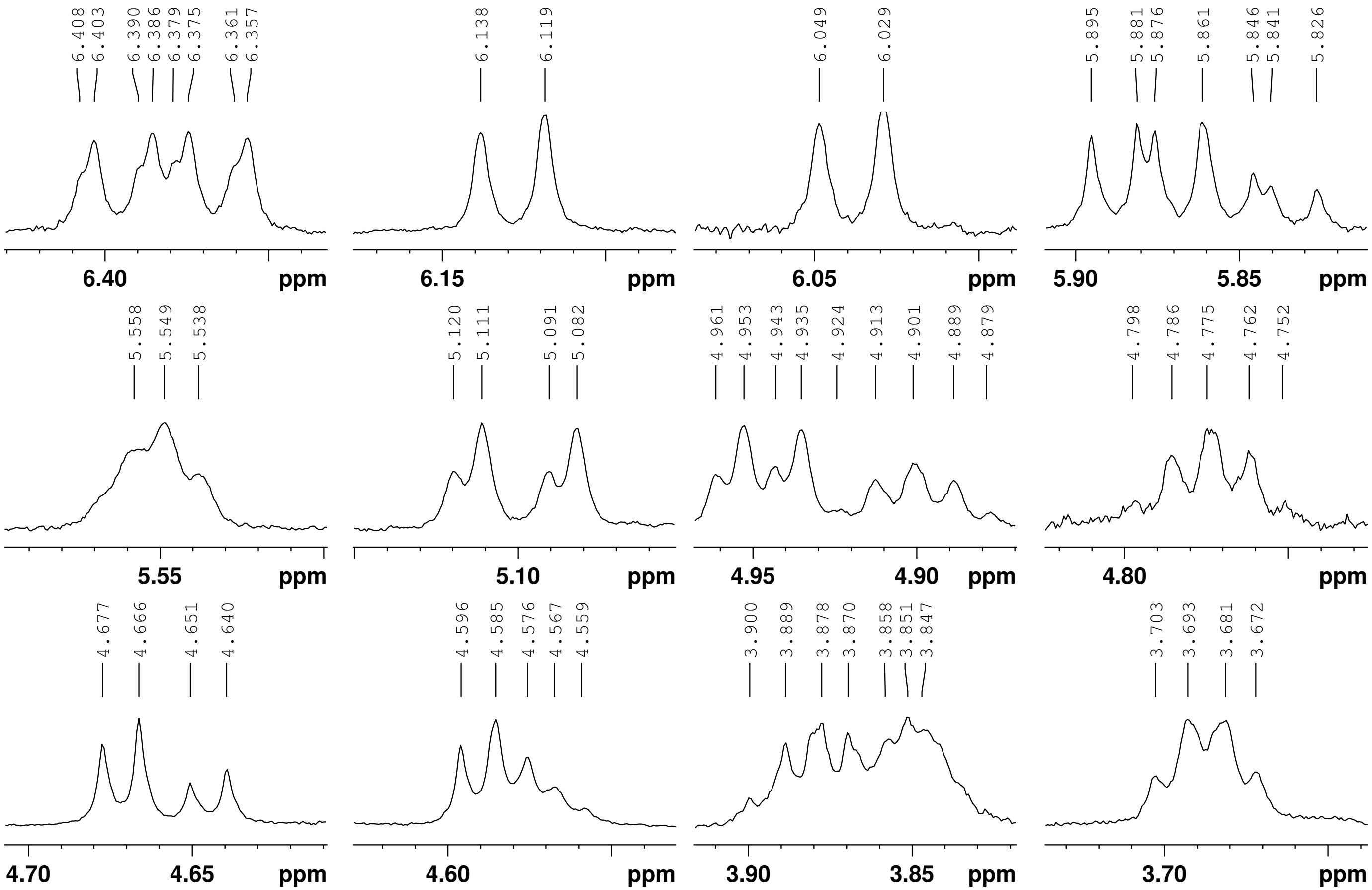

^{1}H NMR spectrum of (*S*,*Z*)-*N*-((2*R*,3*R*,5*S*,6*S*)-2,5-dimethyl-6-((*E*)-3-methylpenta-2,4-dien-1-yl)tetrahydro-2*H*-pyran-3-yl)-4-(methoxymethoxy)-*N*-methylpent-2-enamide **19** (600 MHz, $CDCl_3$, 293K)

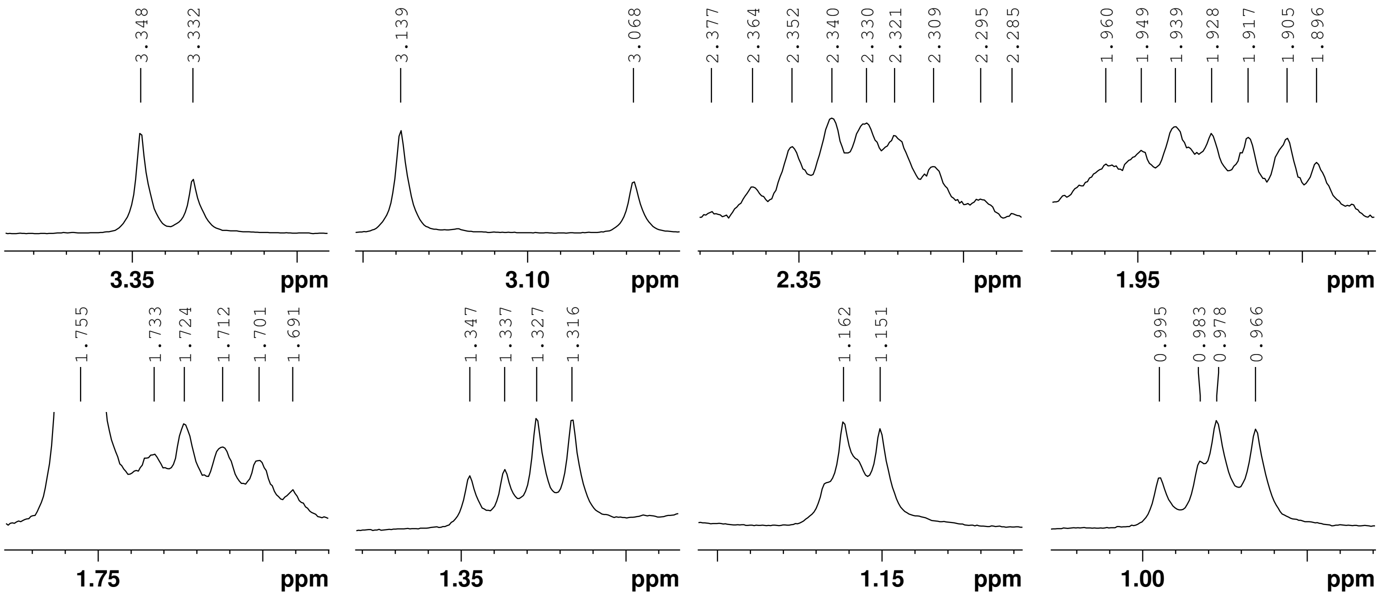

^{1}H NMR spectrum of (*S*,*Z*)-*N*-((2*R*,3*R*,5*S*,6*S*)-2,5-dimethyl-6-((*E*)-3-methylpenta-2,4-dien-1-yl)tetrahydro-2*H*-pyran-3-yl)-4-(methoxymethoxy)-*N*-methylpent-2-enamide **19** (600 MHz, $CDCl_3$, 293K)

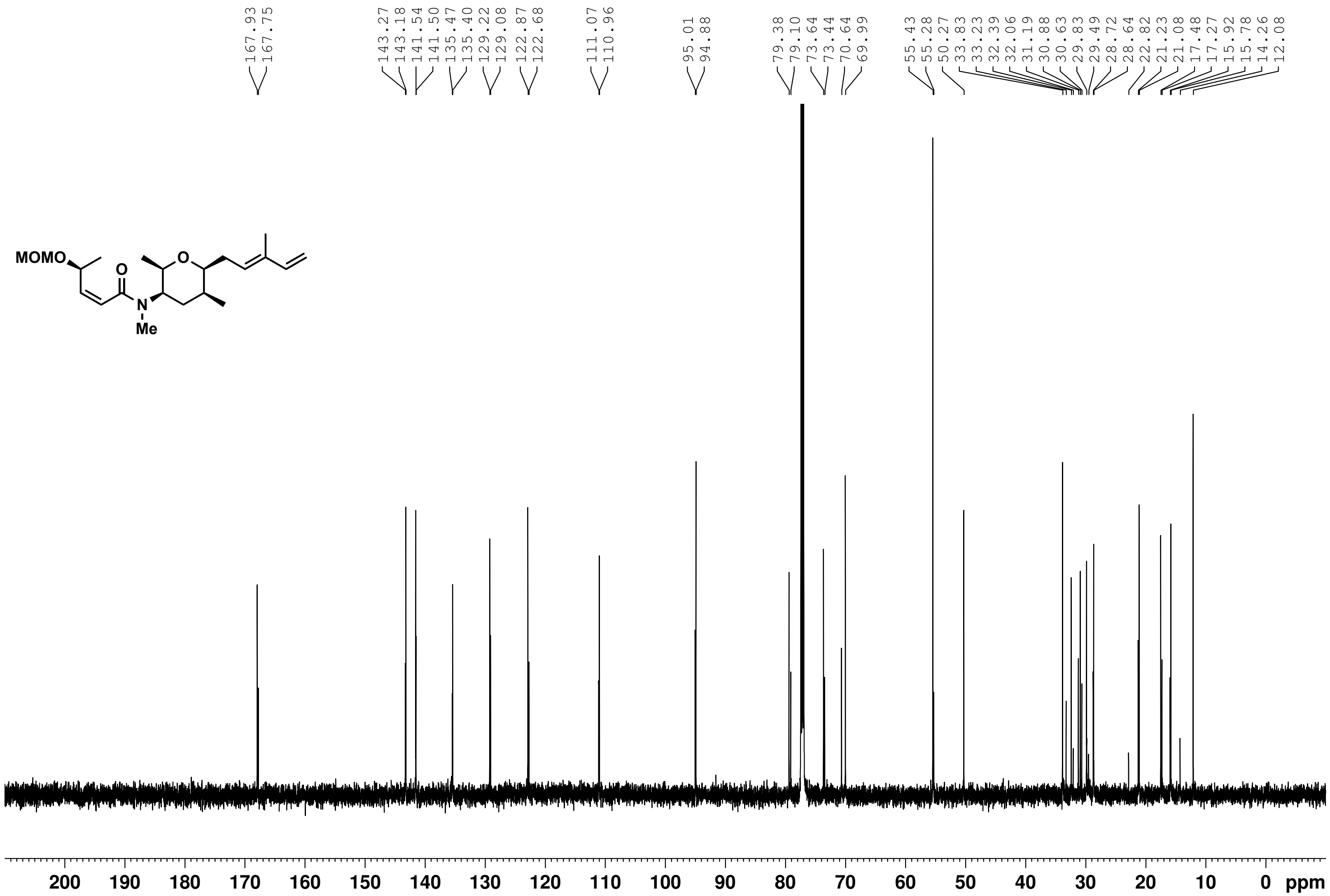

^{13}C NMR spectrum of (*S*,*Z*)-*N*-((2*R*,3*R*,5*S*,6*S*)-2,5-dimethyl-6-((*E*)-3-methylpenta-2,4-dien-1-yl)tetrahydro-2*H*-pyran-3-yl)-4-(methoxymethoxy)-*N*-methylpent-2-enamide **19** (150 MHz, $CDCl_3$, 293K)

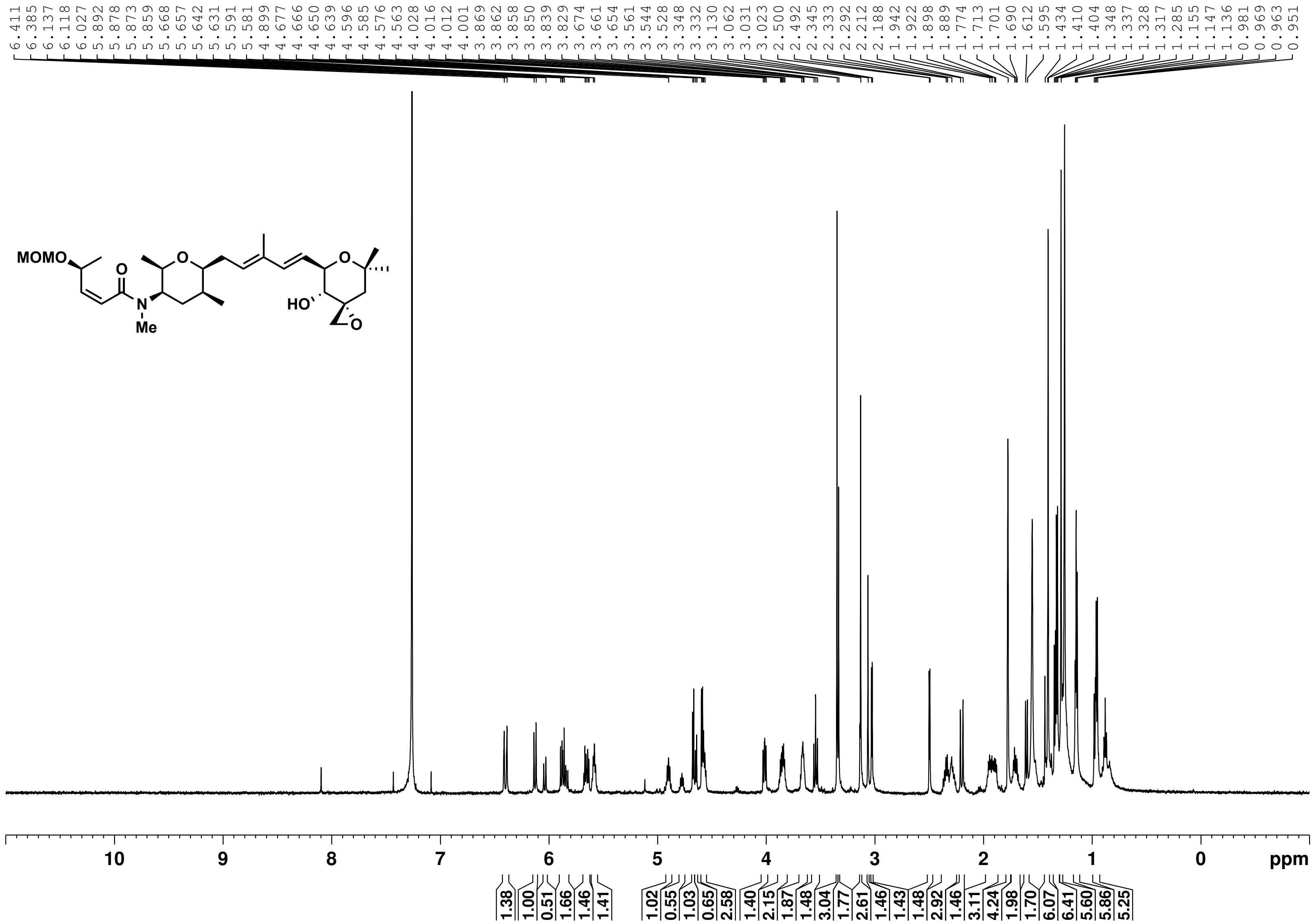

^{1}H NMR spectrum of N-methyl meayamycin D (600 MHz, $CDCl_3$, 293K)

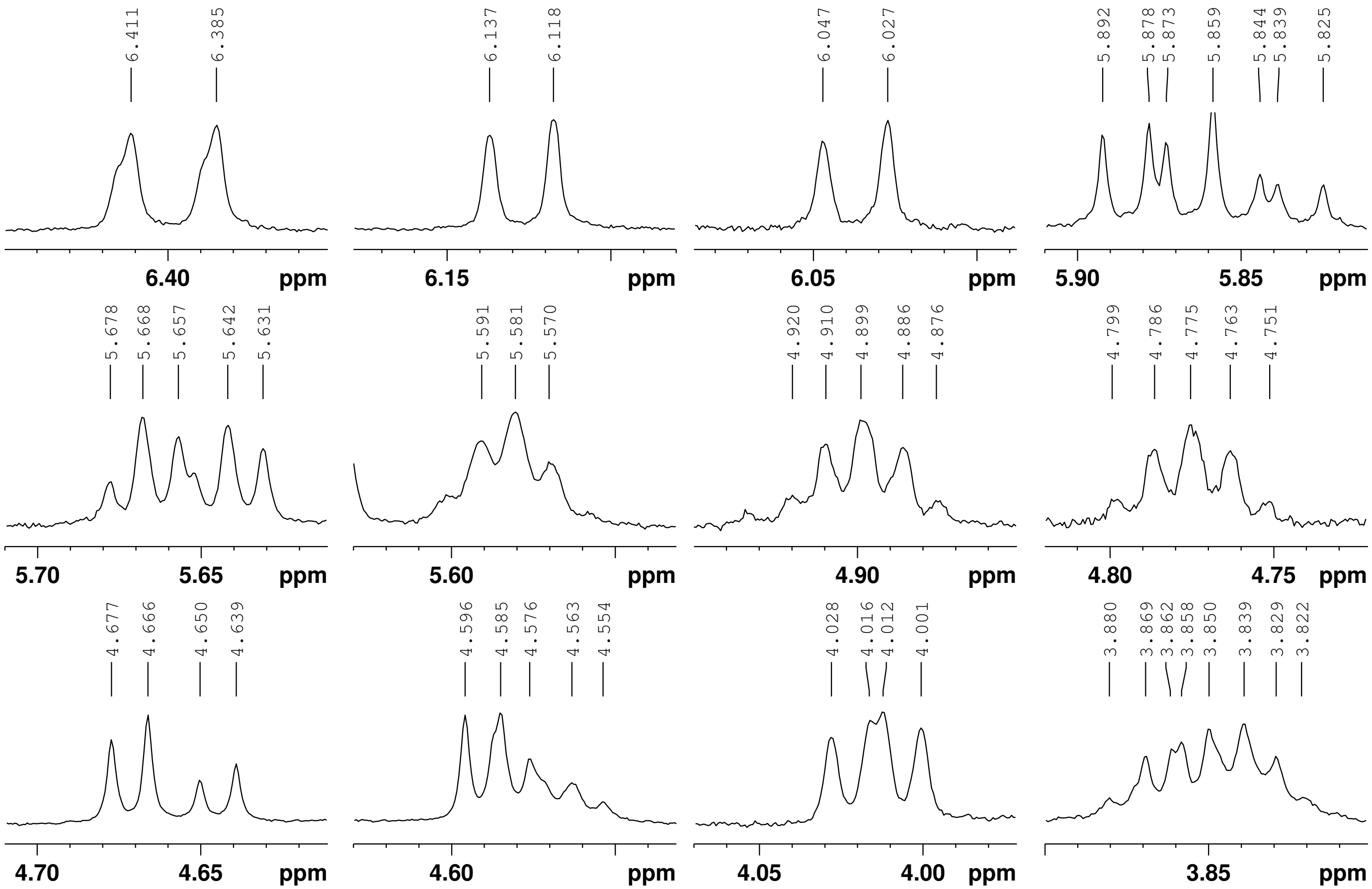

^{1}H NMR spectrum of N-methyl meayamycin D (600 MHz, $CDCl_3$, 293K)

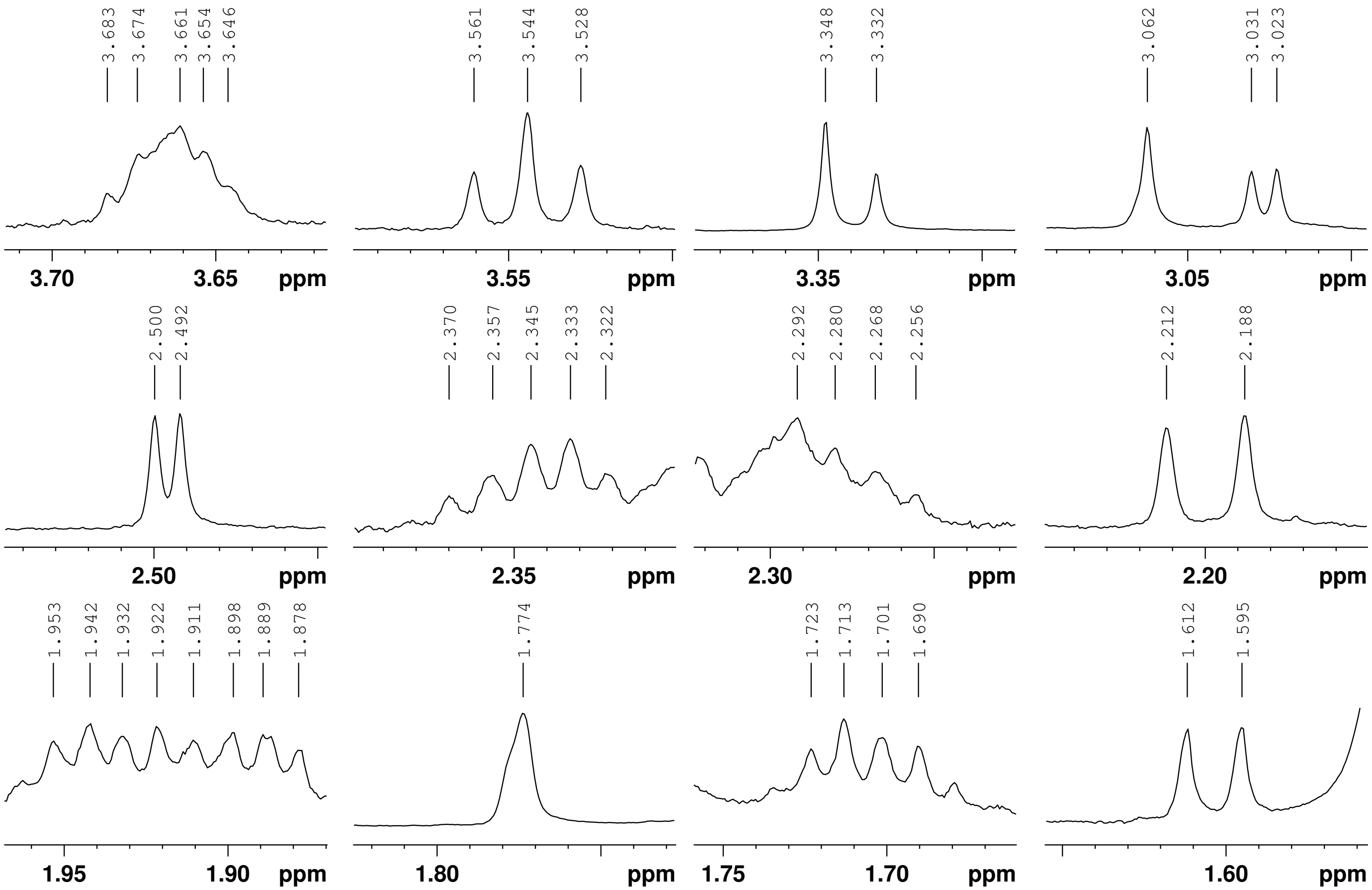

^{1}H NMR spectrum of N-methyl meayamycin D (600 MHz, $CDCl_3$, 293K)

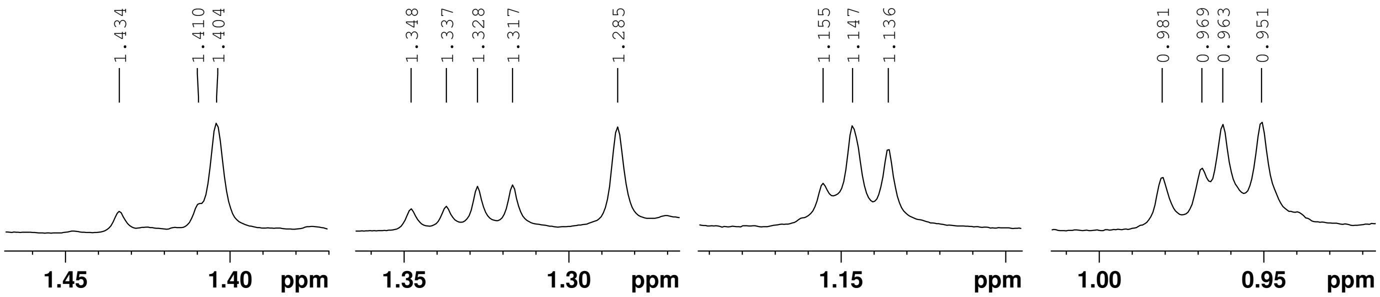

^{1}H NMR spectrum of N-methyl meayamycin D (600 MHz, $CDCl_3$, 293K)

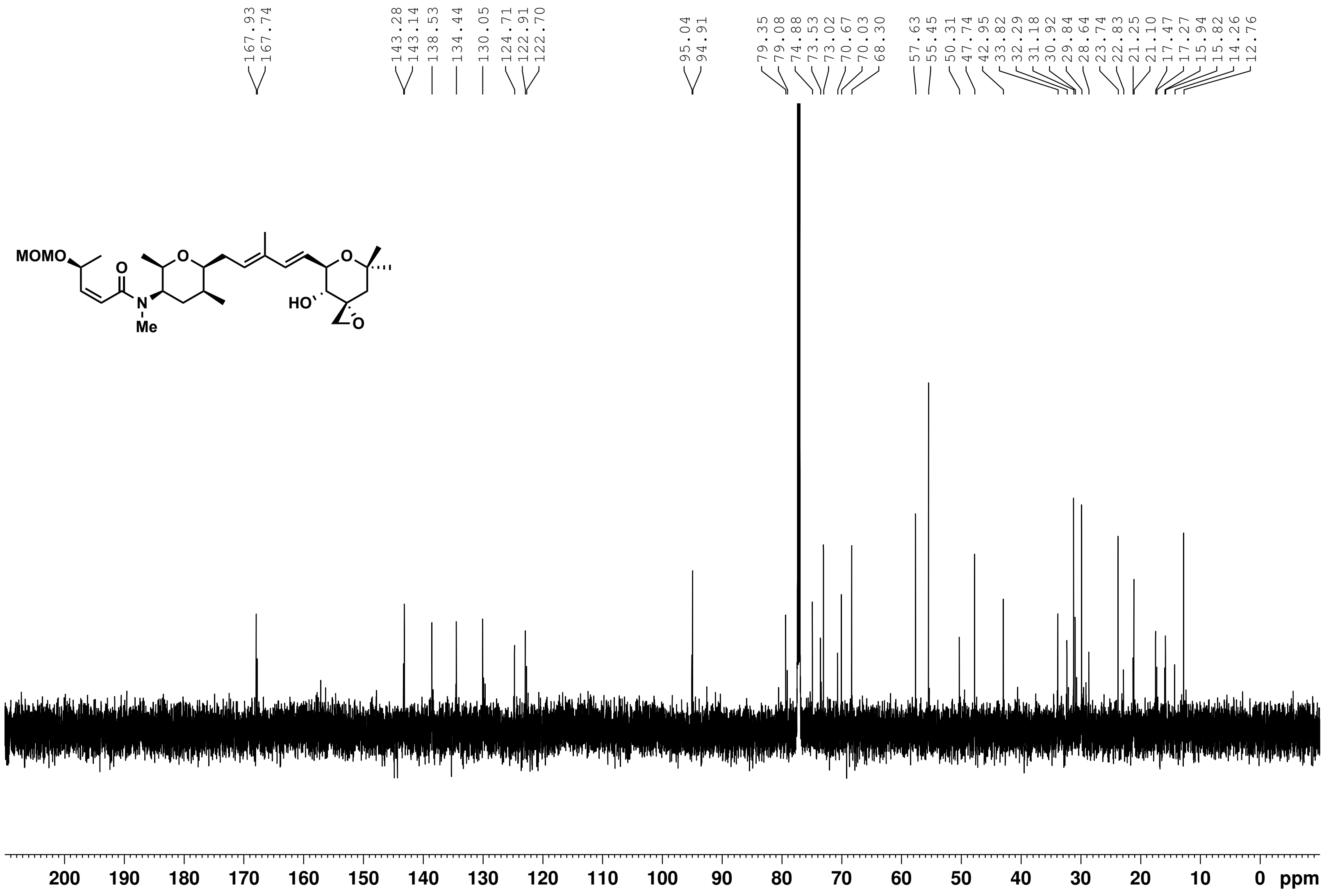

^{13}C NMR spectrum of N-methyl meayamycin D (175 MHz, $CDCl_3$, 293K)

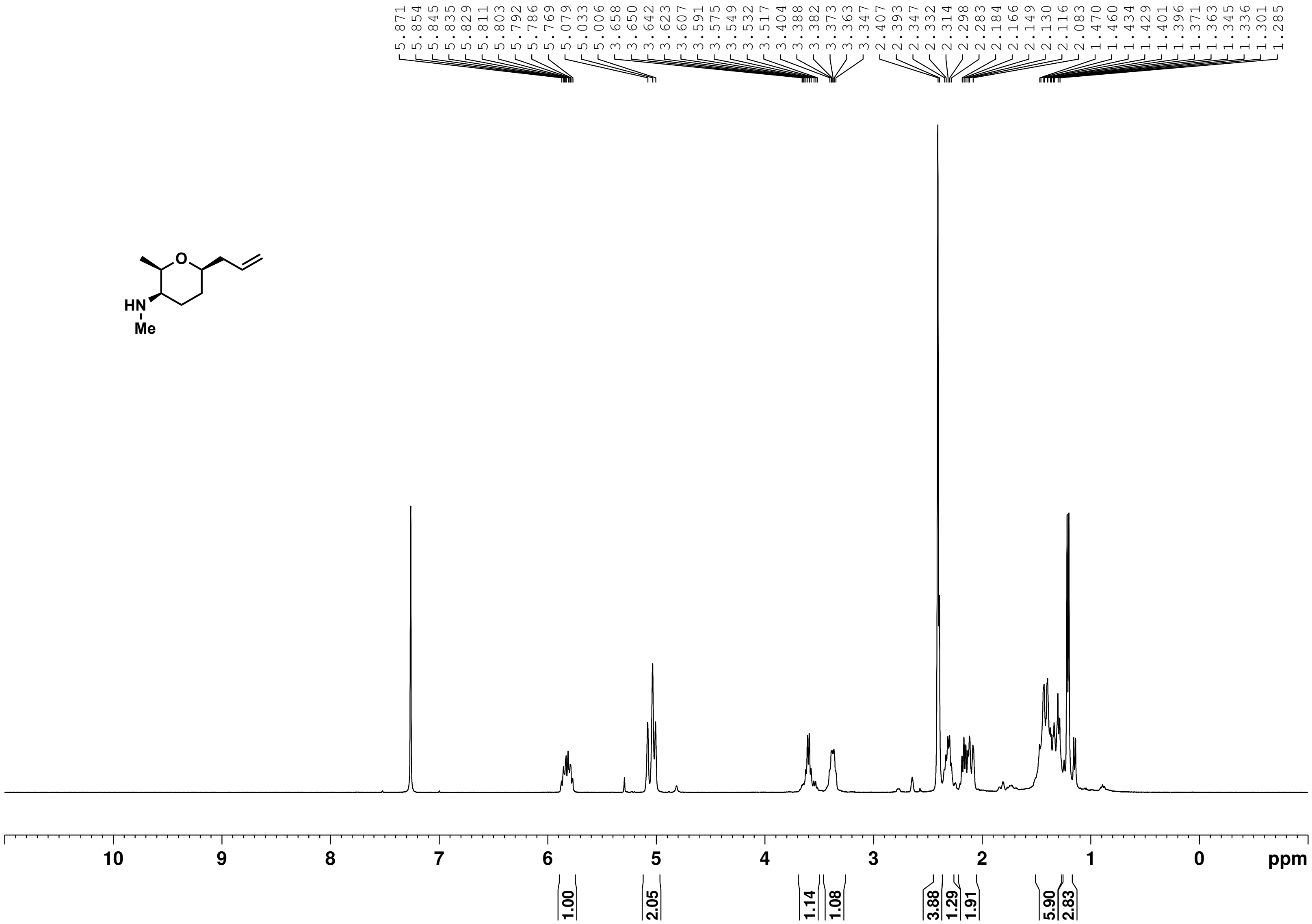

^{1}H NMR spectrum of (2*R*,3*R*,6*R*)-6-allyl-*N*,2-dimethyltetrahydro-2*H*-pyran-3-amine **20** (400 MHz, 1% CD_3OD in $CDCl_3$, 293K)

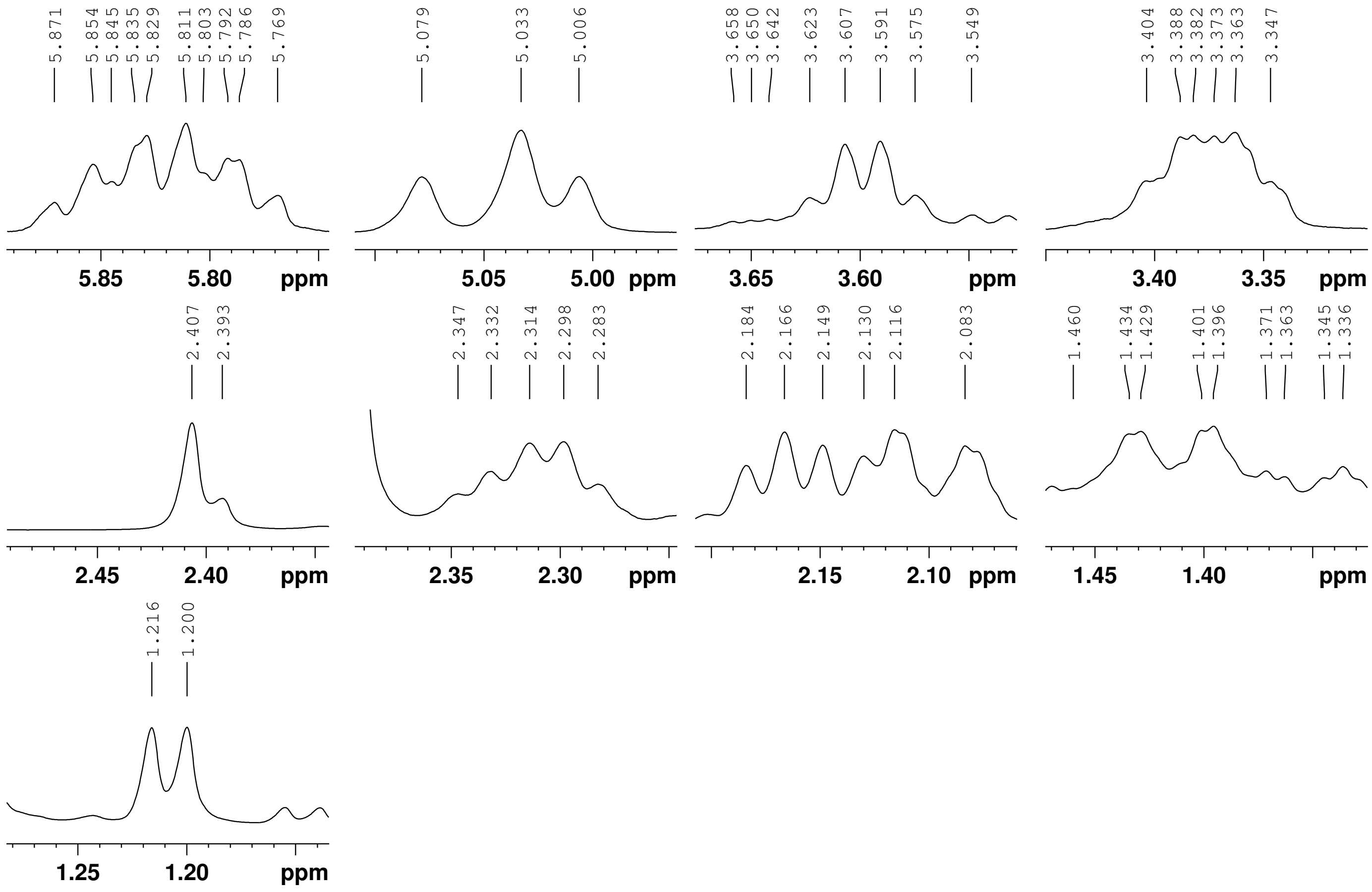

^{1}H NMR spectrum of (2*R*,3*R*,6*R*)-6-allyl-*N*,2-dimethyltetrahydro-2*H*-pyran-3-amine **20** (400 MHz, 1% CD_3OD in $CDCl_3$, 293K)

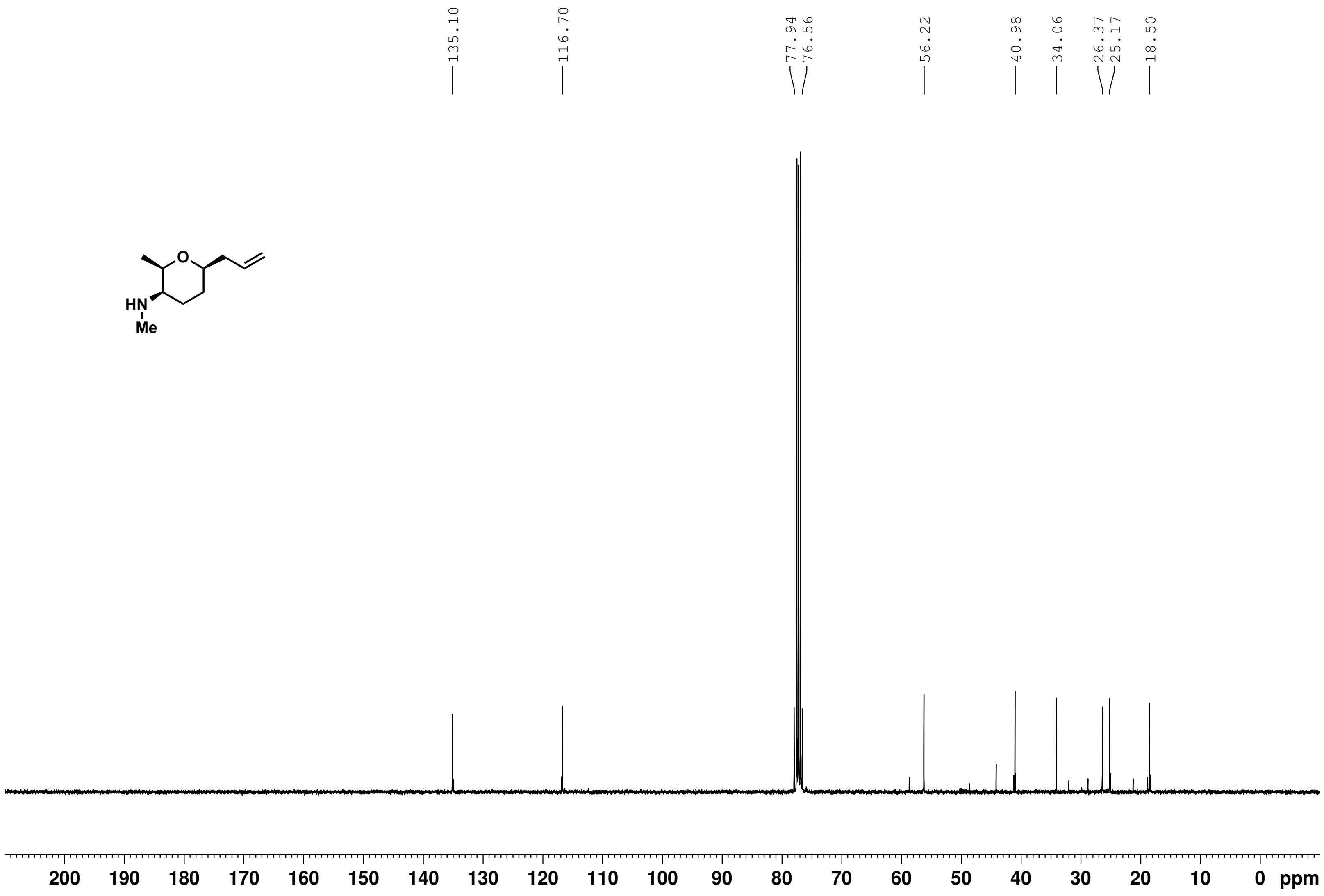

^{13}C NMR spectrum of (2*R*,3*R*,6*R*)-6-allyl-*N*,2-dimethyltetrahydro-2*H*-pyran-3-amine **20** (100 MHz, 1% CD_3OD in $CDCl_3$, 293K)

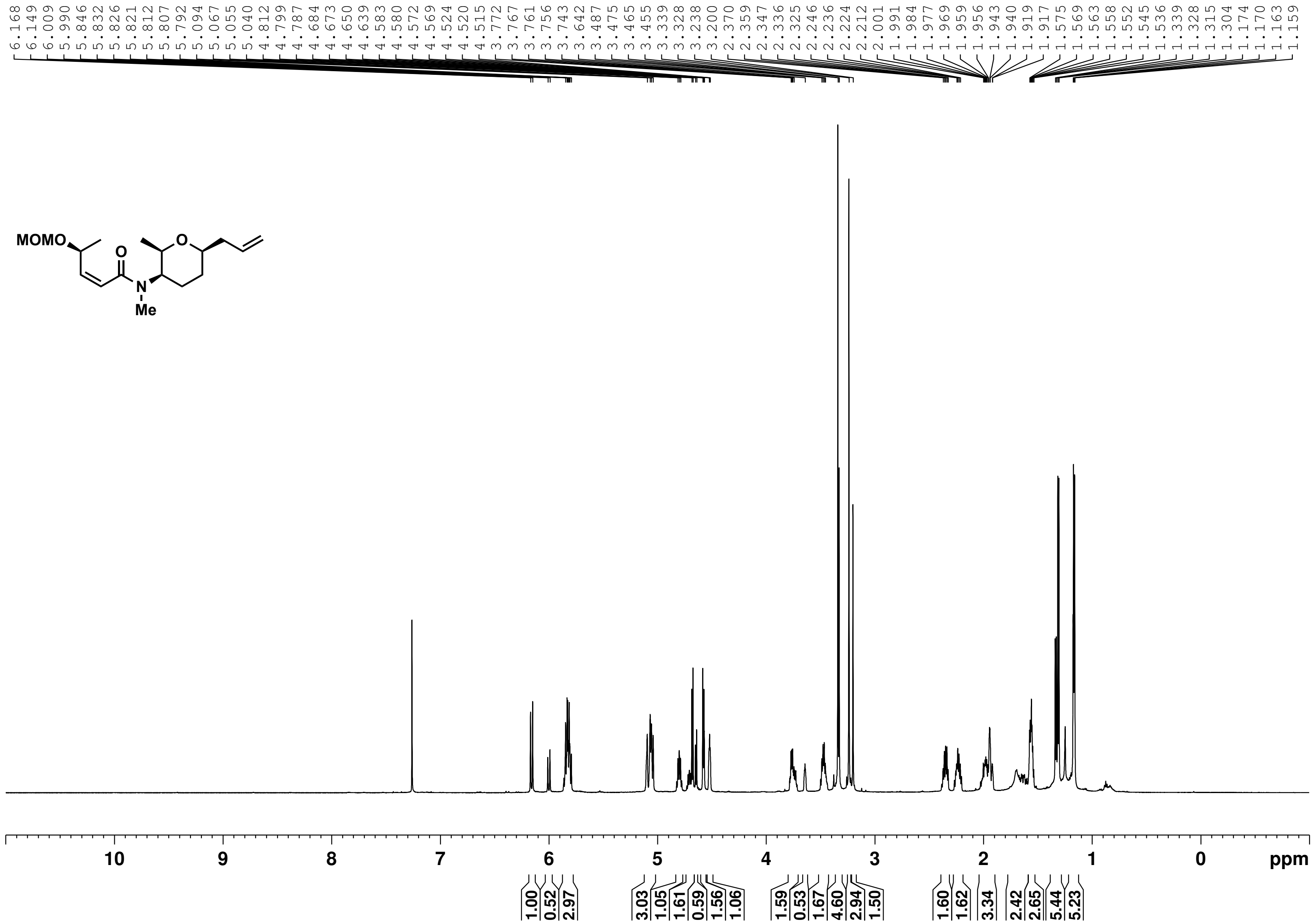

^{1}H NMR spectrum of (*S*,*Z*)-*N*-((2*R*,3*R*,6*R*)-6-allyl-2-methyltetrahydro-2*H*-pyran-3-yl)-4-(methoxymethoxy)-*N*-methylpent-2-enamide **21** (600 MHz, $CDCl_3$, 293K)

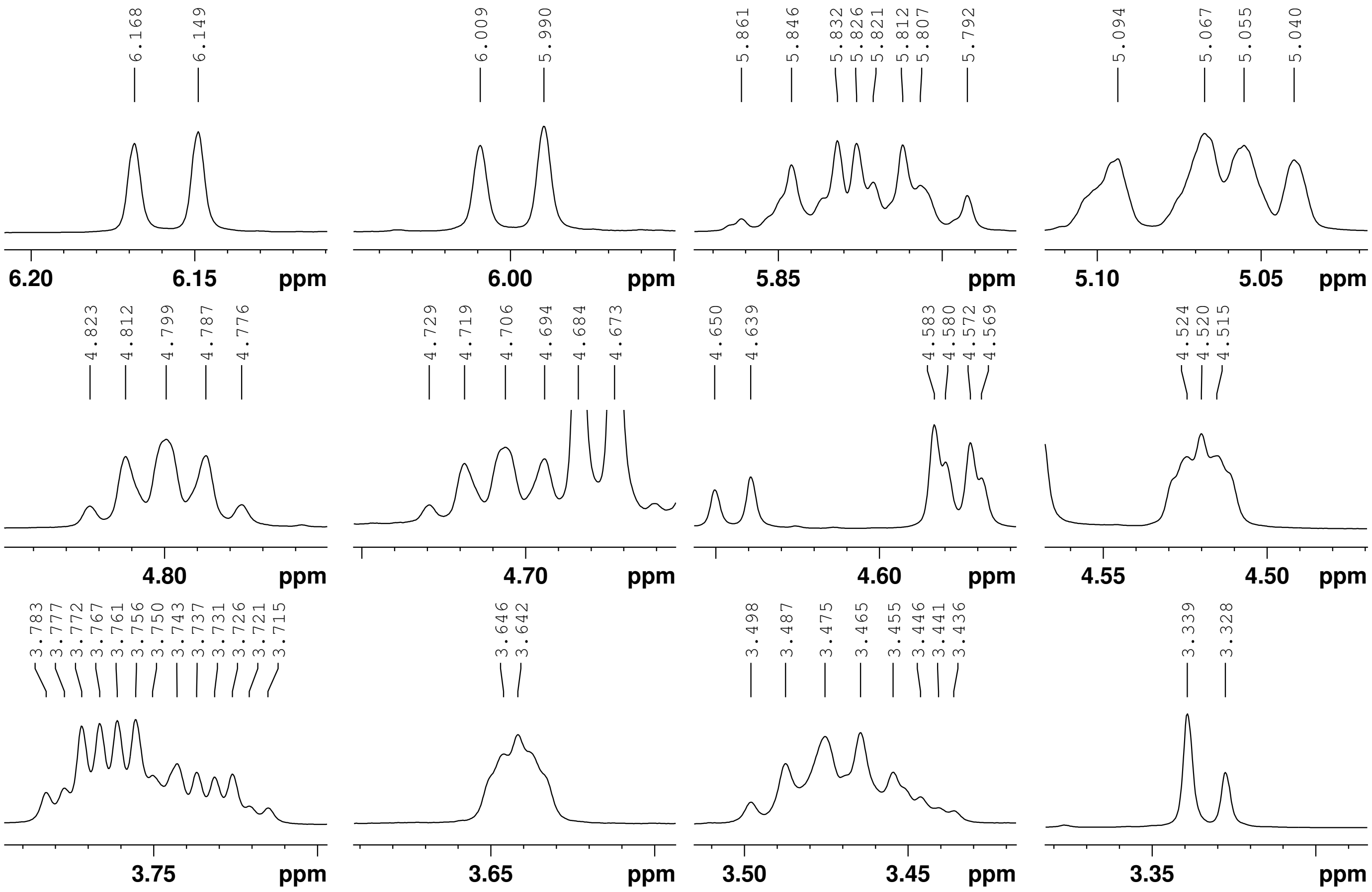

^{1}H NMR spectrum of (*S*,*Z*)-*N*-((2*R*,3*R*,6*R*)-6-allyl-2-methyltetrahydro-2*H*-pyran-3-yl)-4-(methoxymethoxy)-*N*-methylpent-2-enamide **21** (600 MHz, $CDCl_3$, 293K)

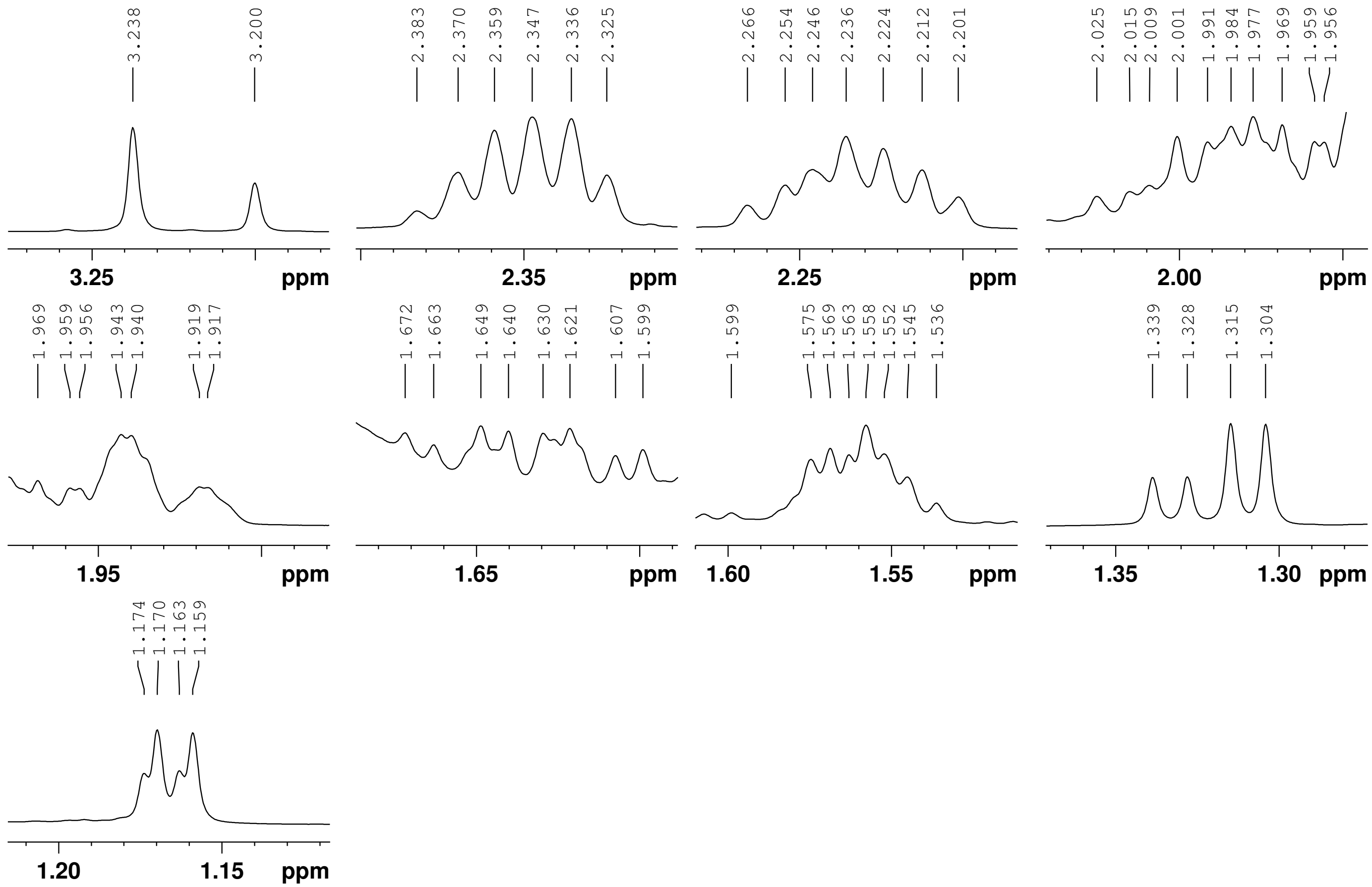

^{1}H NMR spectrum of (*S*,*Z*)-*N*-((2*R*,3*R*,6*R*)-6-allyl-2-methyltetrahydro-2*H*-pyran-3-yl)-4-(methoxymethoxy)-*N*-methylpent-2-enamide **21** (600 MHz, $CDCl_3$, 293K)

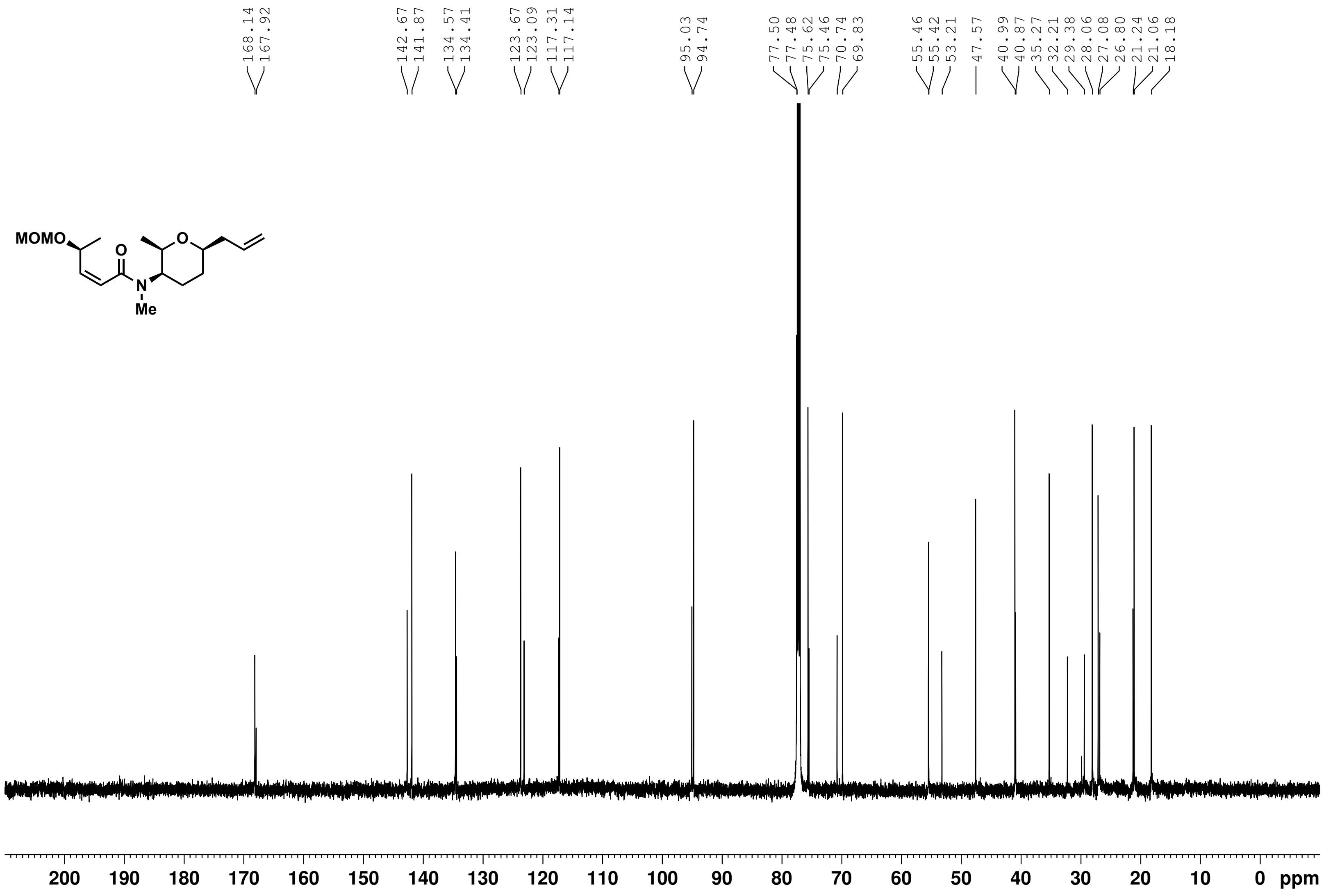

^{13}C NMR spectrum of (*S*,*Z*)-*N*-((2*R*,3*R*,6*R*)-6-allyl-2-methyltetrahydro-2*H*-pyran-3-yl)-4-(methoxymethoxy)-*N*-methylpent-2-enamide **21** (150 MHz, $CDCl_3$, 293K)

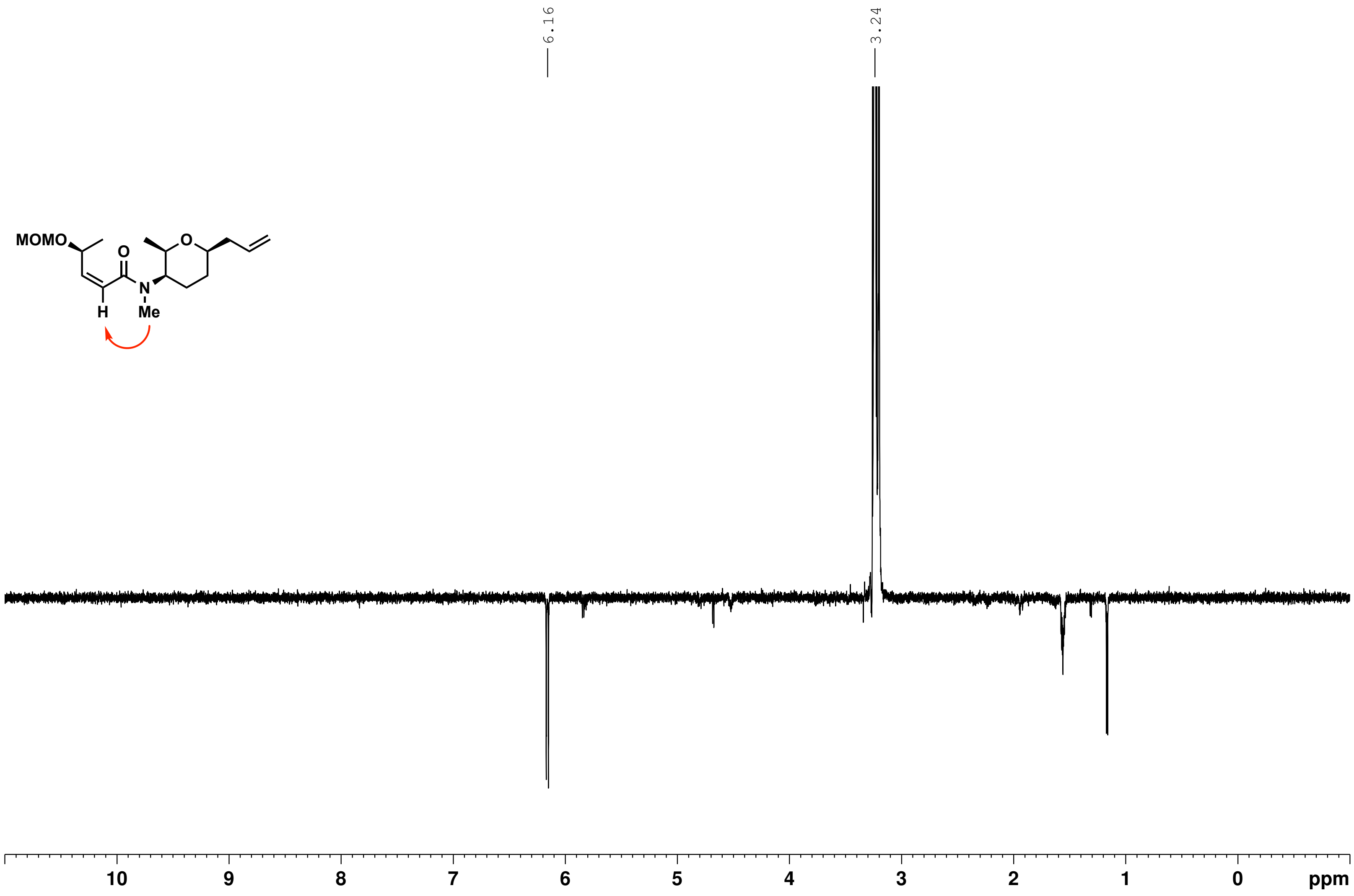

Selective 1D NOESY spectrum of (*S*,*Z*)-*N*-((2*R*,3*R*,6*R*)-6-allyl-2-methyltetrahydro-2*H*-pyran-3-yl)-4-(methoxymethoxy)-*N*-methylpent-2-enamide **21** (600 MHz, $CDCl_3$, 293K, major rotamer)

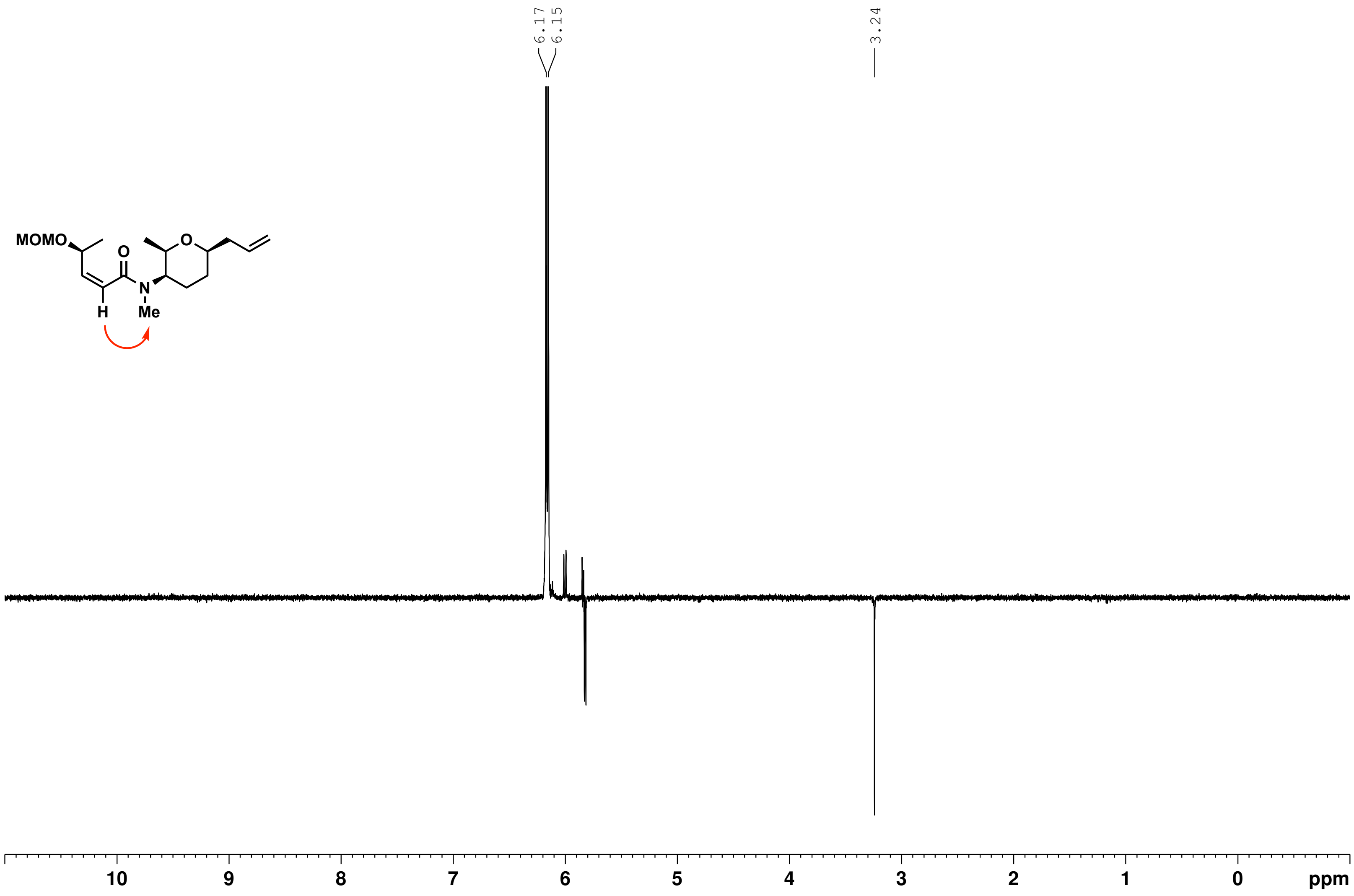

Selective 1D NOESY spectrum of (*S*,*Z*)-*N*-((2*R*,3*R*,6*R*)-6-allyl-2-methyltetrahydro-2*H*-pyran-3-yl)-4-(methoxymethoxy)-*N*-methylpent-2-enamide **21** (600 MHz, $CDCl_3$, 293K, major rotamer)

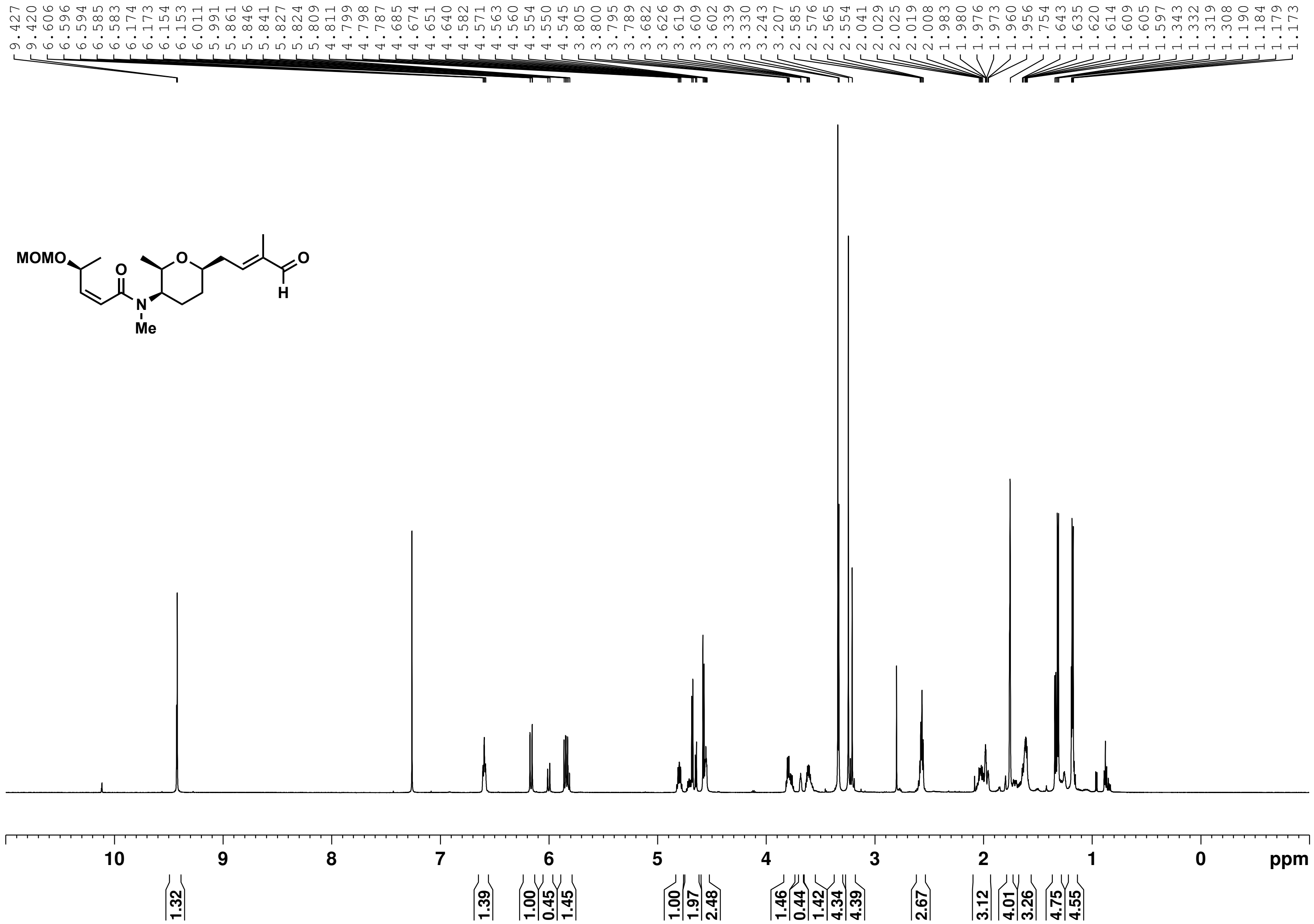

^{1}H NMR spectrum of (*S*,*Z*)-4-(methoxymethoxy)-*N*-methyl-*N*-((2*R*,3*R*,6*R*)-2-methyl-6-((*E*)-3-methyl-4-oxobut-2-en-1-yl)tetrahydro-2*H*-pyran-3-yl)pent-2-enamide **22** (600 MHz, $CDCl_3$, 293K)

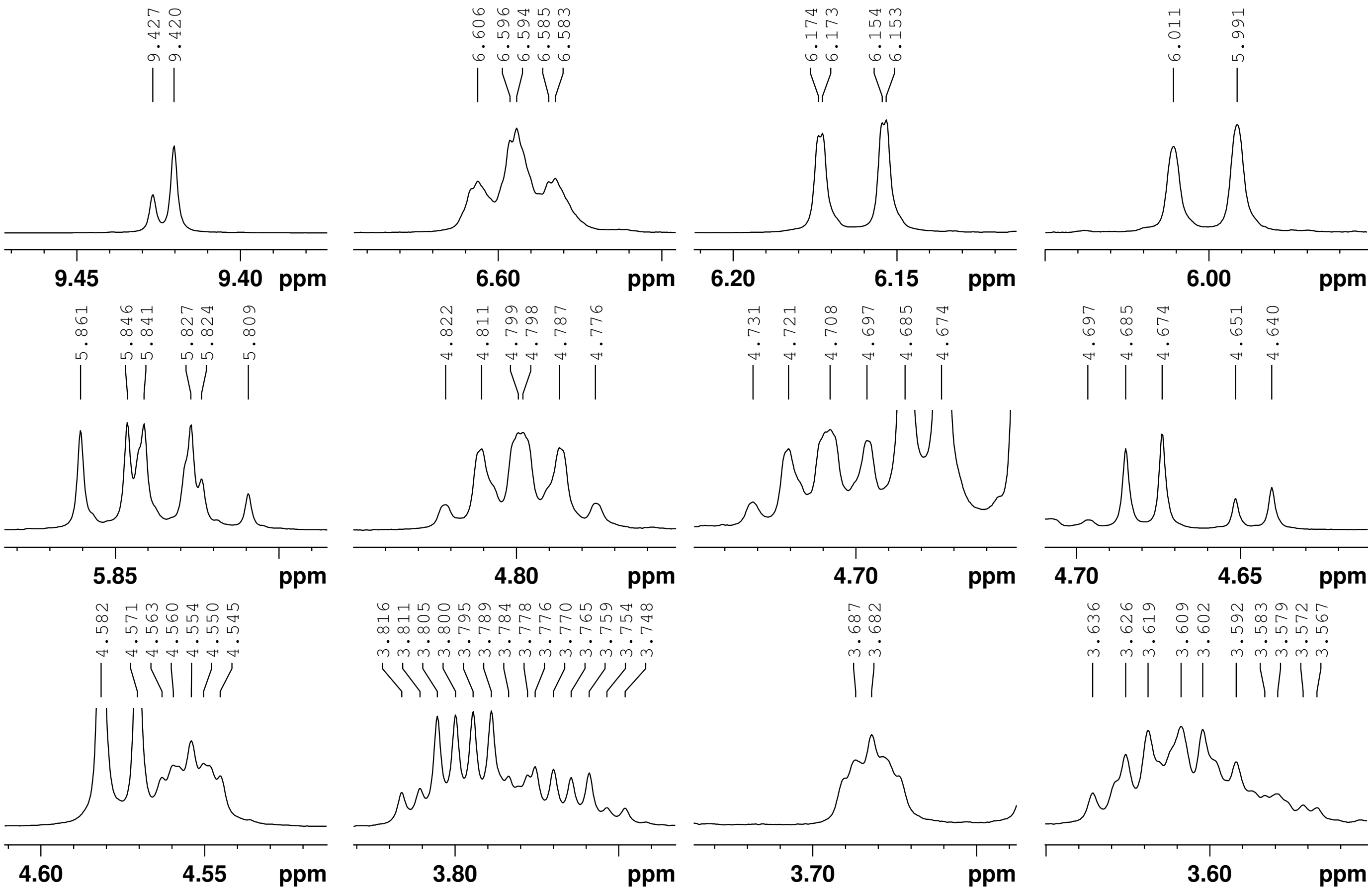

^{1}H NMR spectrum of (*S*,*Z*)-4-(methoxymethoxy)-*N*-methyl-*N*-((2*R*,3*R*,6*R*)-2-methyl-6-((*E*)-3-methyl-4-oxobut-2-en-1-yl)tetrahydro-2*H*-pyran-3-yl)pent-2-enamide **22** (600 MHz, $CDCl_3$, 293K)

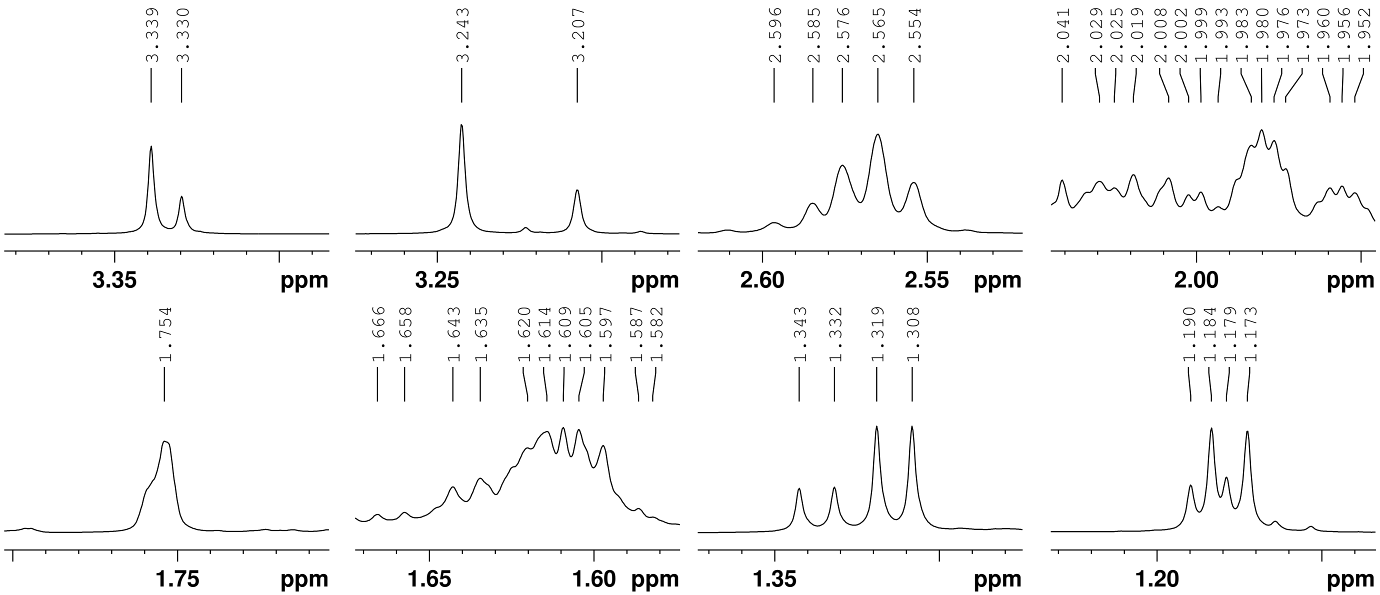

^{1}H NMR spectrum of (*S*,*Z*)-4-(methoxymethoxy)-*N*-methyl-*N*-((2*R*,3*R*,6*R*)-2-methyl-6-((*E*)-3-methyl-4-oxobut-2-en-1-yl)tetrahydro-2*H*-pyran-3-yl)pent-2-enamide **22** (600 MHz, $CDCl_3$, 293K)

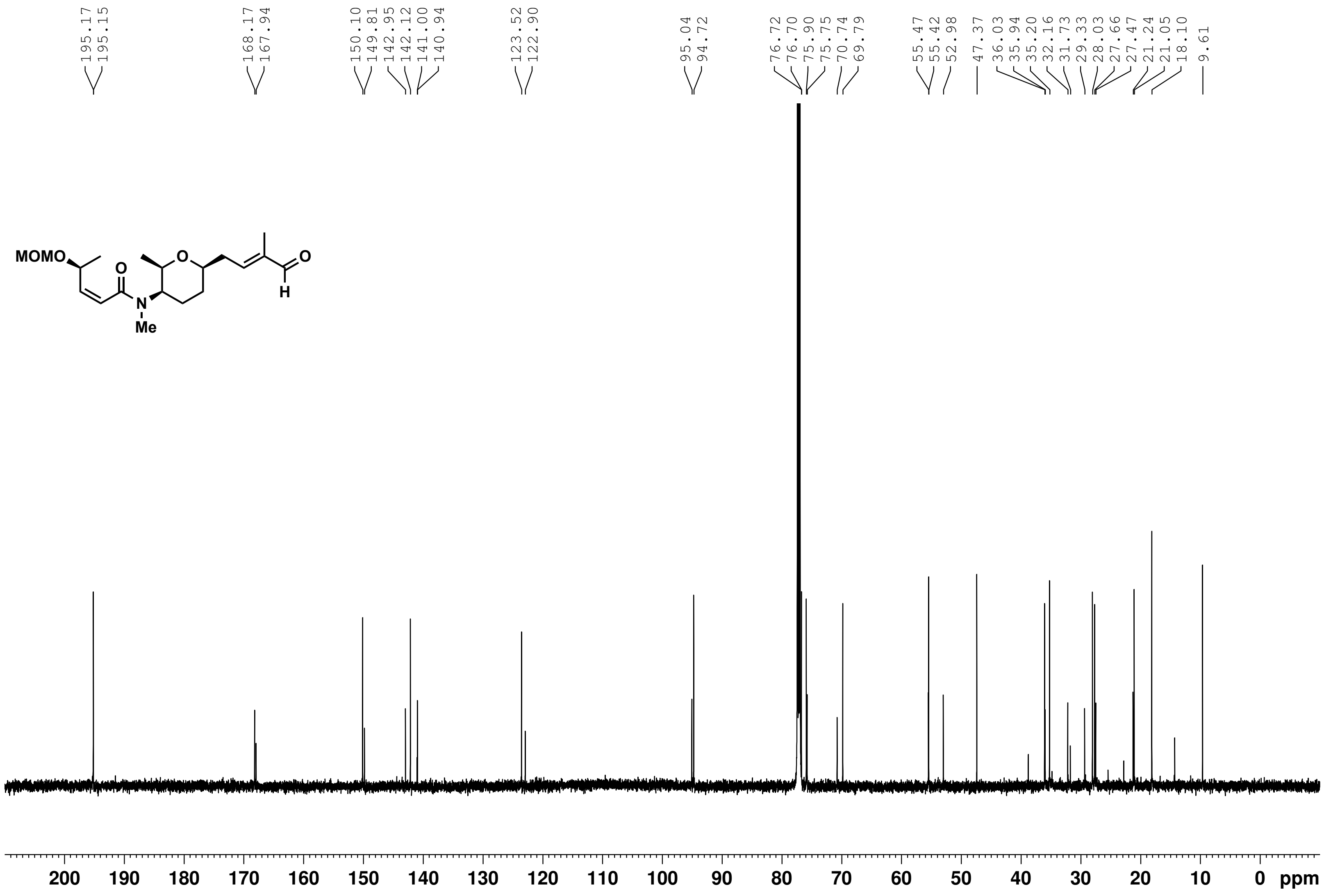

^{13}C NMR spectrum of (*S*,*Z*)-4-(methoxymethoxy)-*N*-methyl-*N*-((2*R*,3*R*,6*R*)-2-methyl-6-((*E*)-3-methyl-4-oxobut-2-en-1-yl)tetrahydro-2*H*-pyran-3-yl)pent-2-enamide **22** (150 MHz, $CDCl_3$, 293K)

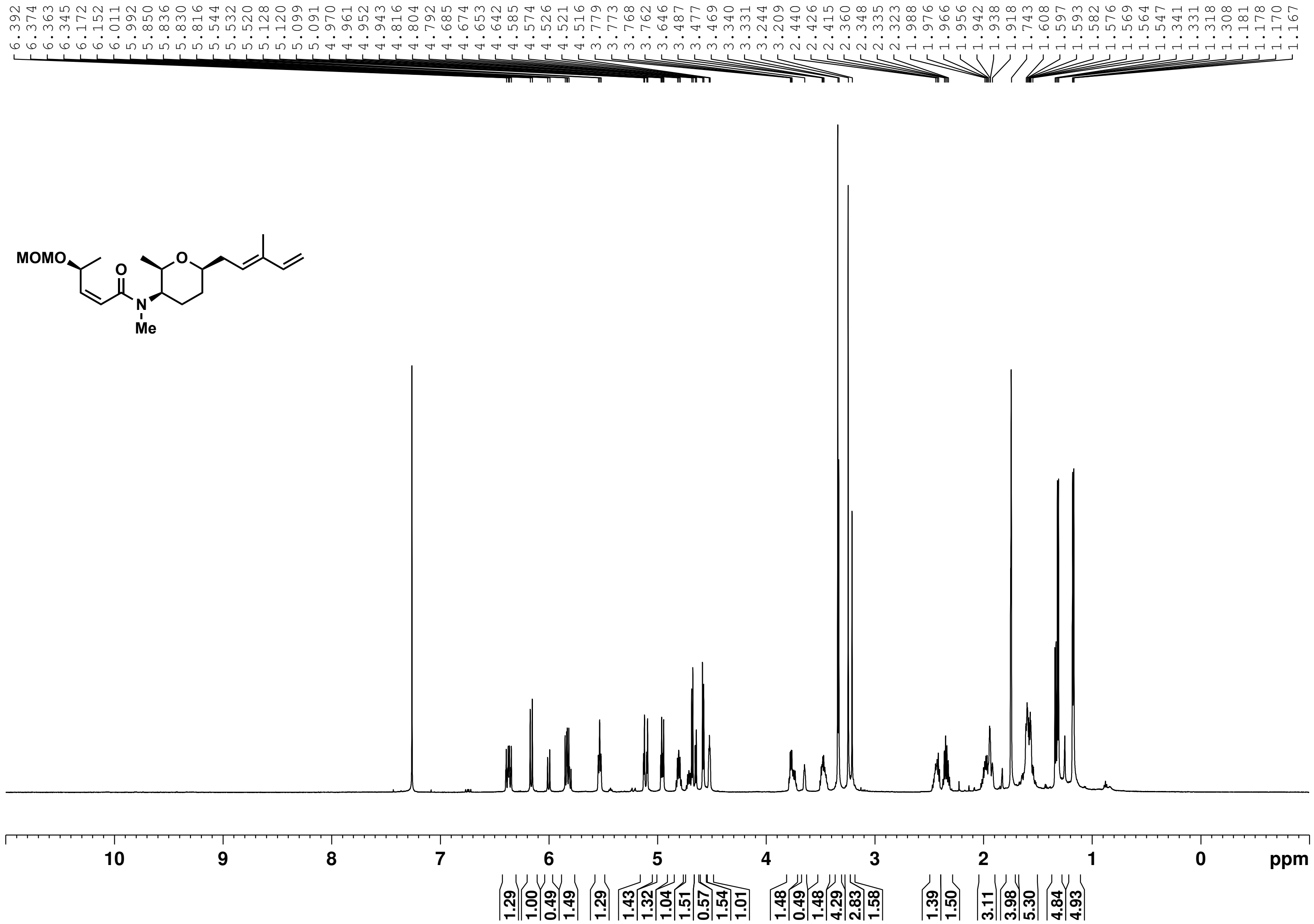

^{1}H NMR spectrum of (*S*,*Z*)-4-(methoxymethoxy)-*N*-methyl-*N*-((2*R*,3*R*,6*R*)-2-methyl-6-((*E*)-3-methylpenta-2,4-dien-1-yl)tetrahydro-2*H*-pyran-3-yl)pent-2-enamide **23** (600 MHz, $CDCl_3$, 293K)

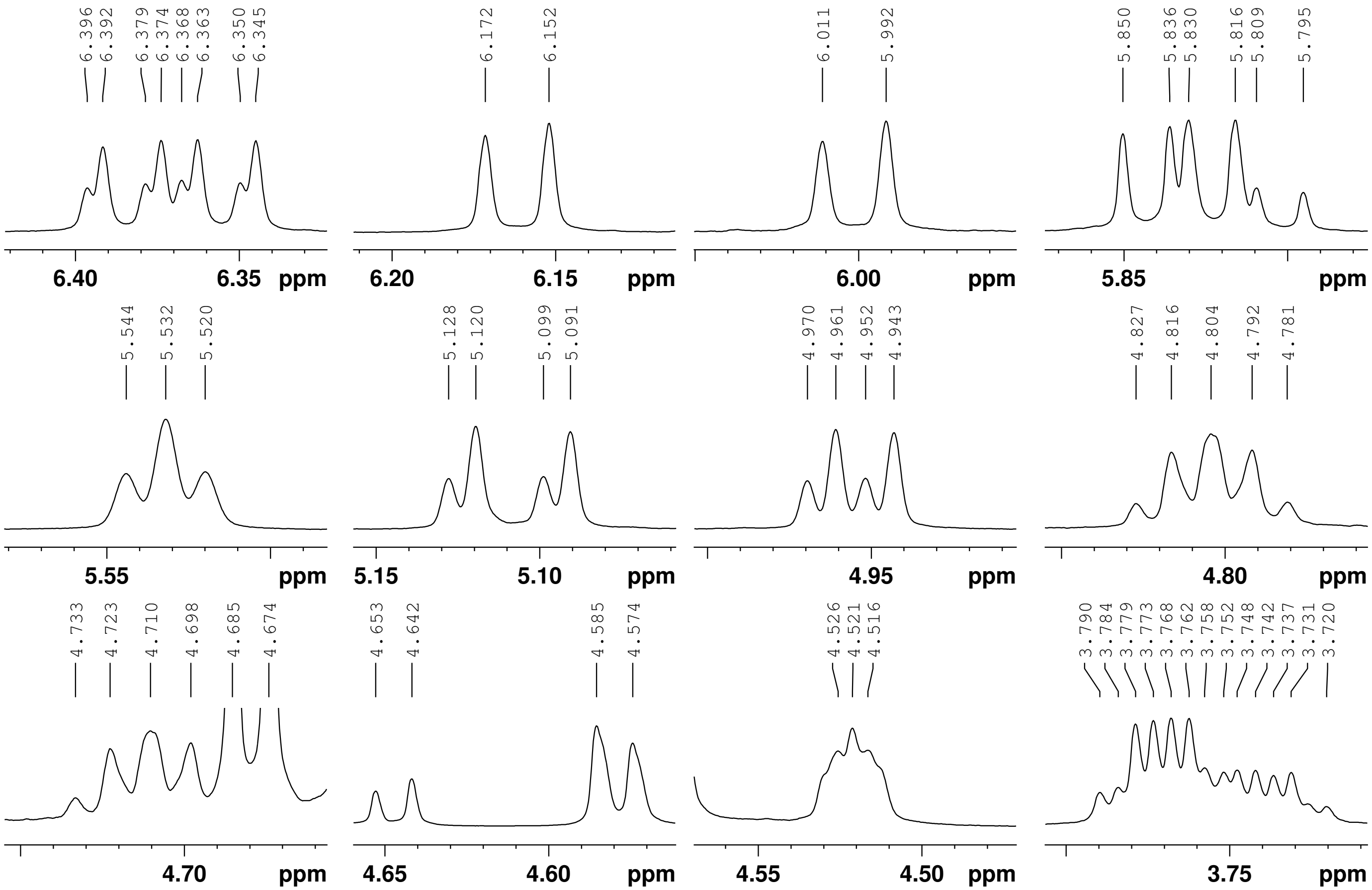

^{1}H NMR spectrum of (*S*,*Z*)-4-(methoxymethoxy)-*N*-methyl-*N*-((2*R*,3*R*,6*R*)-2-methyl-6-((*E*)-3-methylpenta-2,4-dien-1-yl)tetrahydro-2*H*-pyran-3-yl)pent-2-enamide **23** (600 MHz, $CDCl_3$, 293K)

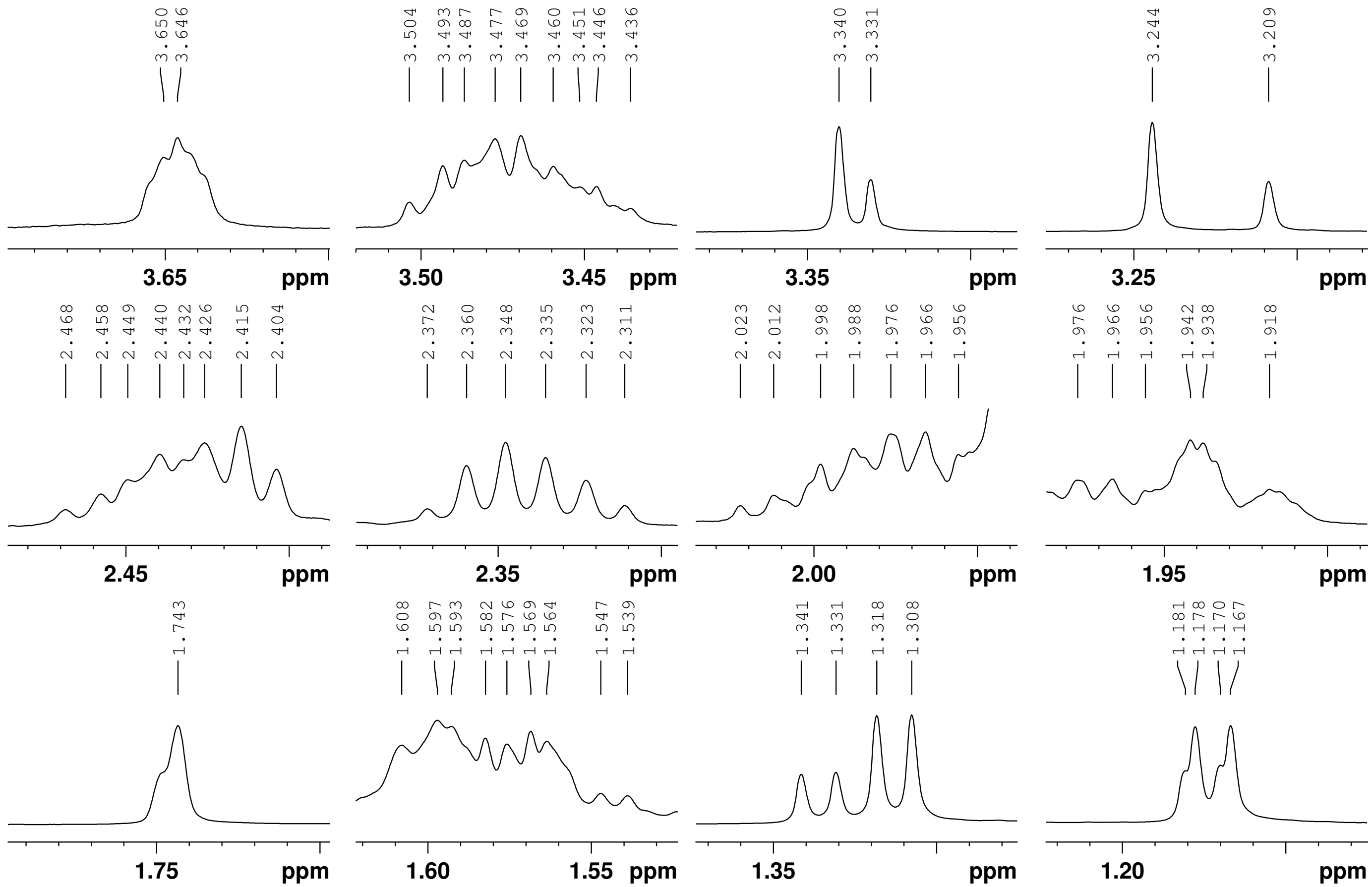

^{1}H NMR spectrum of (*S*,*Z*)-4-(methoxymethoxy)-*N*-methyl-*N*-((2*R*,3*R*,6*R*)-2-methyl-6-((*E*)-3-methylpenta-2,4-dien-1-yl)tetrahydro-2*H*-pyran-3-yl)pent-2-enamide **23** (600 MHz, $CDCl_3$, 293K)

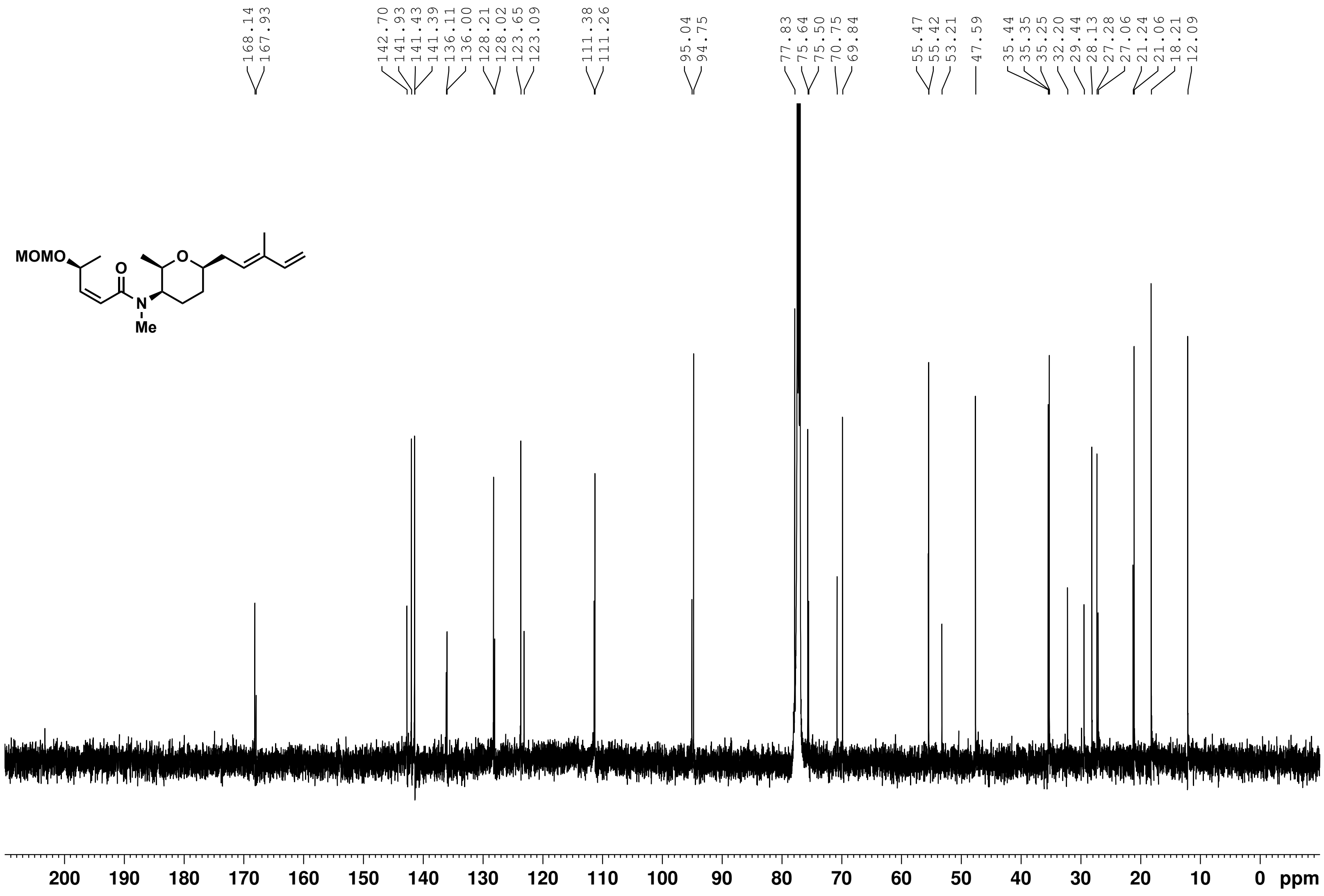

^{13}C NMR spectrum of (*S*,*Z*)-4-(methoxymethoxy)-*N*-methyl-*N*-((2*R*,3*R*,6*R*)-2-methyl-6-((*E*)-3-methylpenta-2,4-dien-1-yl)tetrahydro-2*H*-pyran-3-yl)pent-2-enamide **23** (150 MHz, $CDCl_3$, 293K)

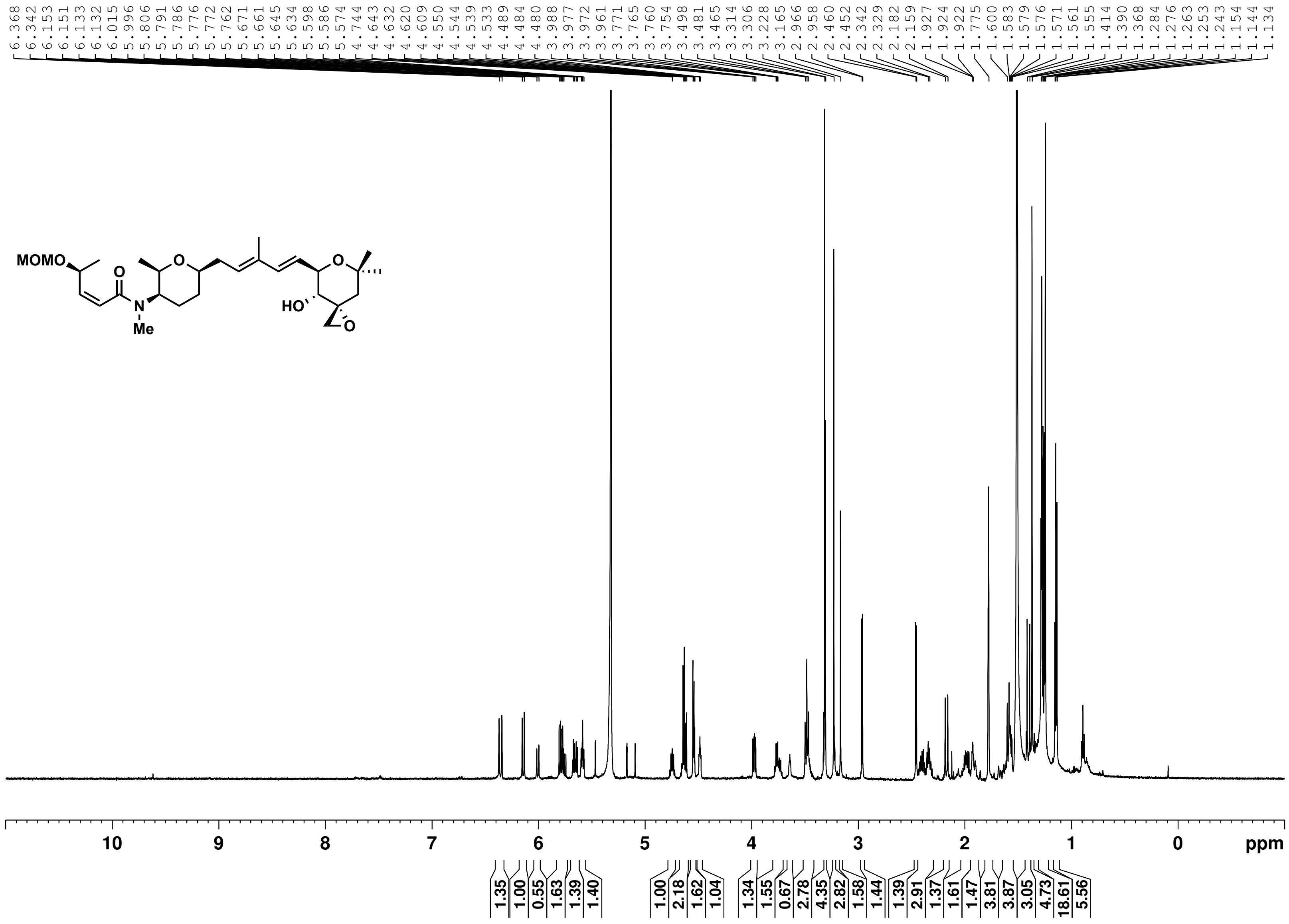

^{1}H NMR spectrum of N-methyl meayamycin E (600 MHz, CD_2Cl_2, 293K)

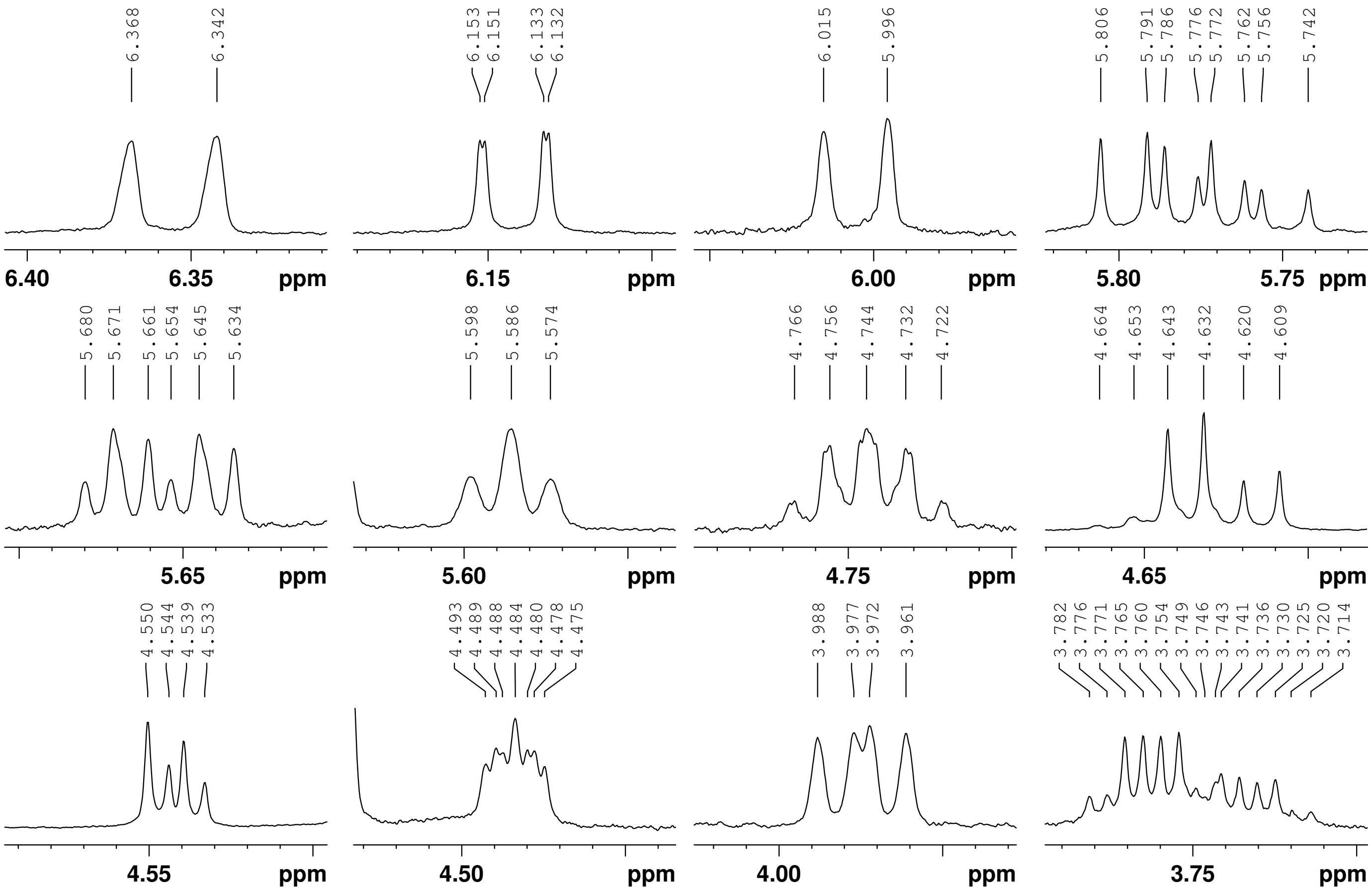

^{1}H NMR spectrum of N-methyl meayamycin E (600 MHz, CD_2Cl_2, 293K)

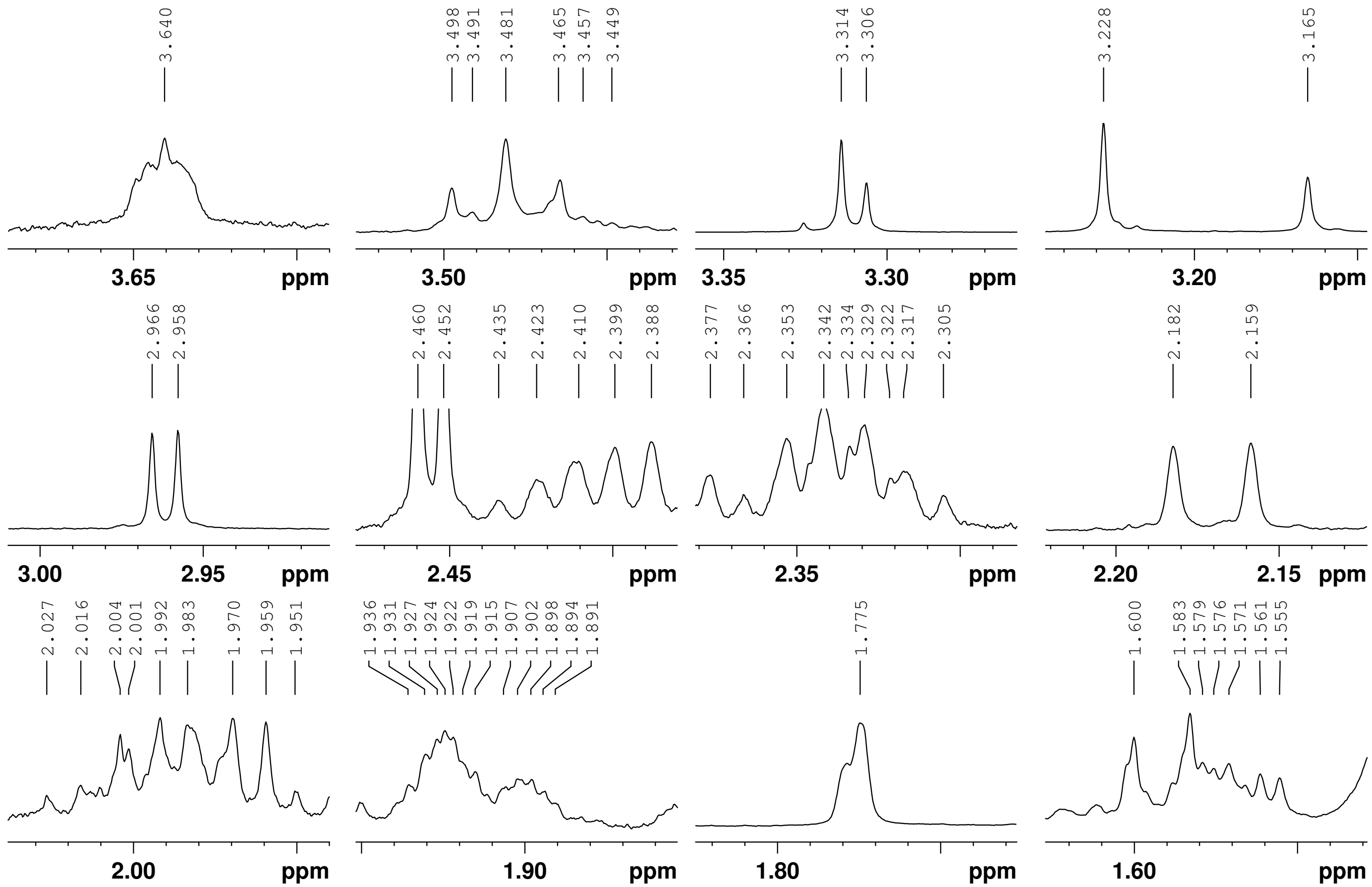

^{1}H NMR spectrum of N-methyl meayamycin E (600 MHz, CD_2Cl_2, 293K)

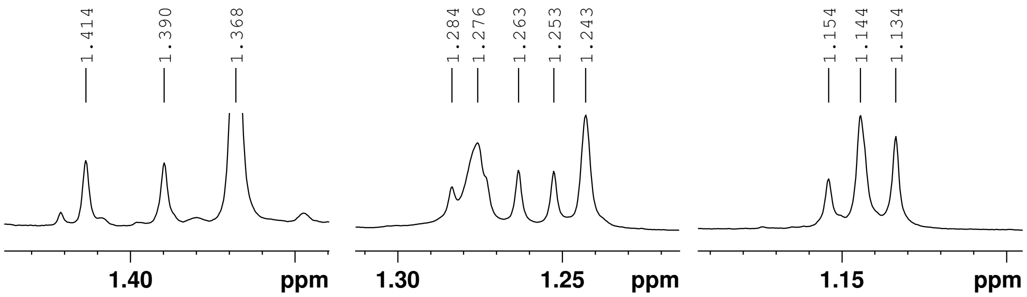

^{1}H NMR spectrum of N-methyl meayamycin E (600 MHz, CD_2Cl_2, 293K)

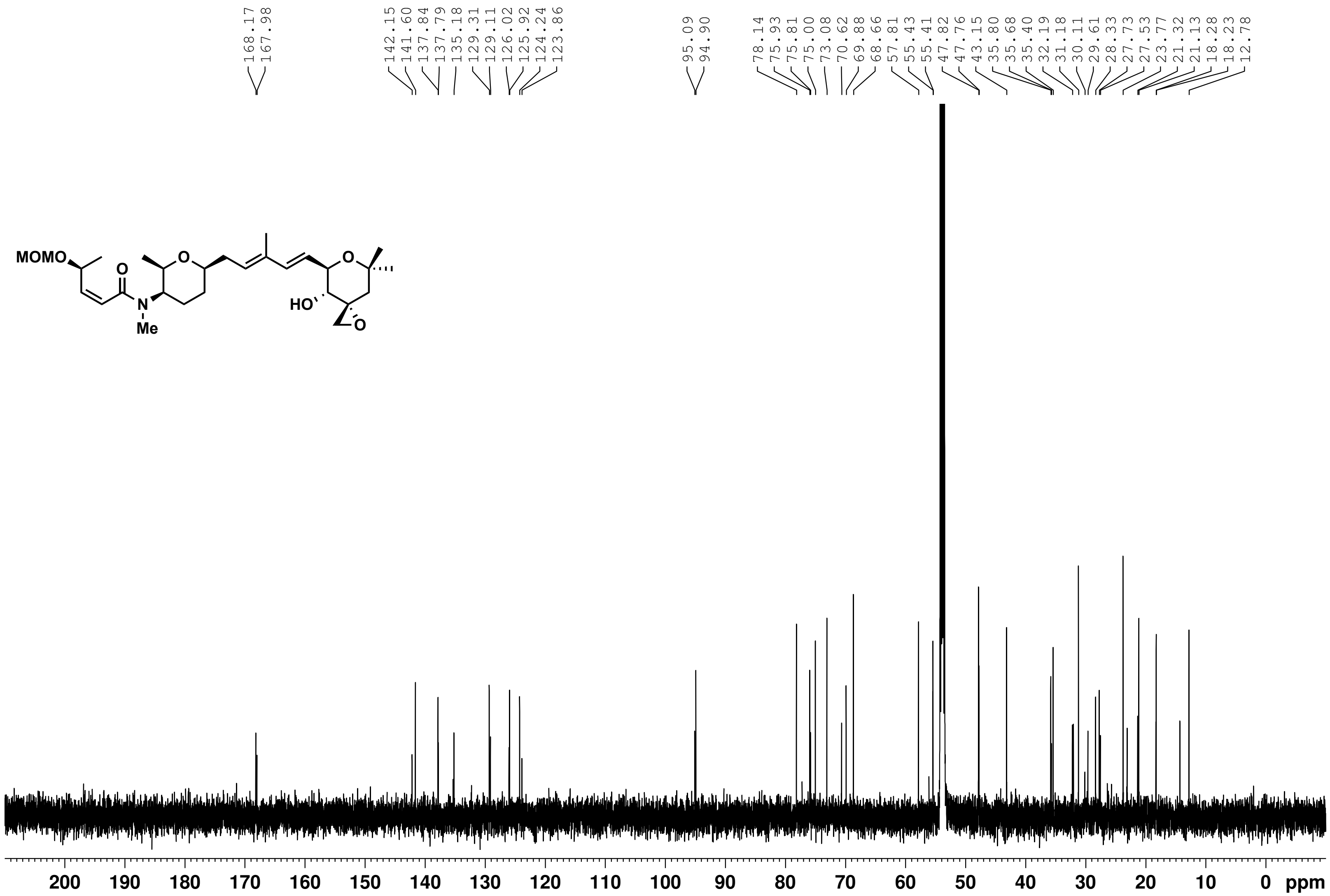

^{13}C NMR spectrum of N-methyl meayamycin E (150 MHz, CD_2Cl_2, 293K)

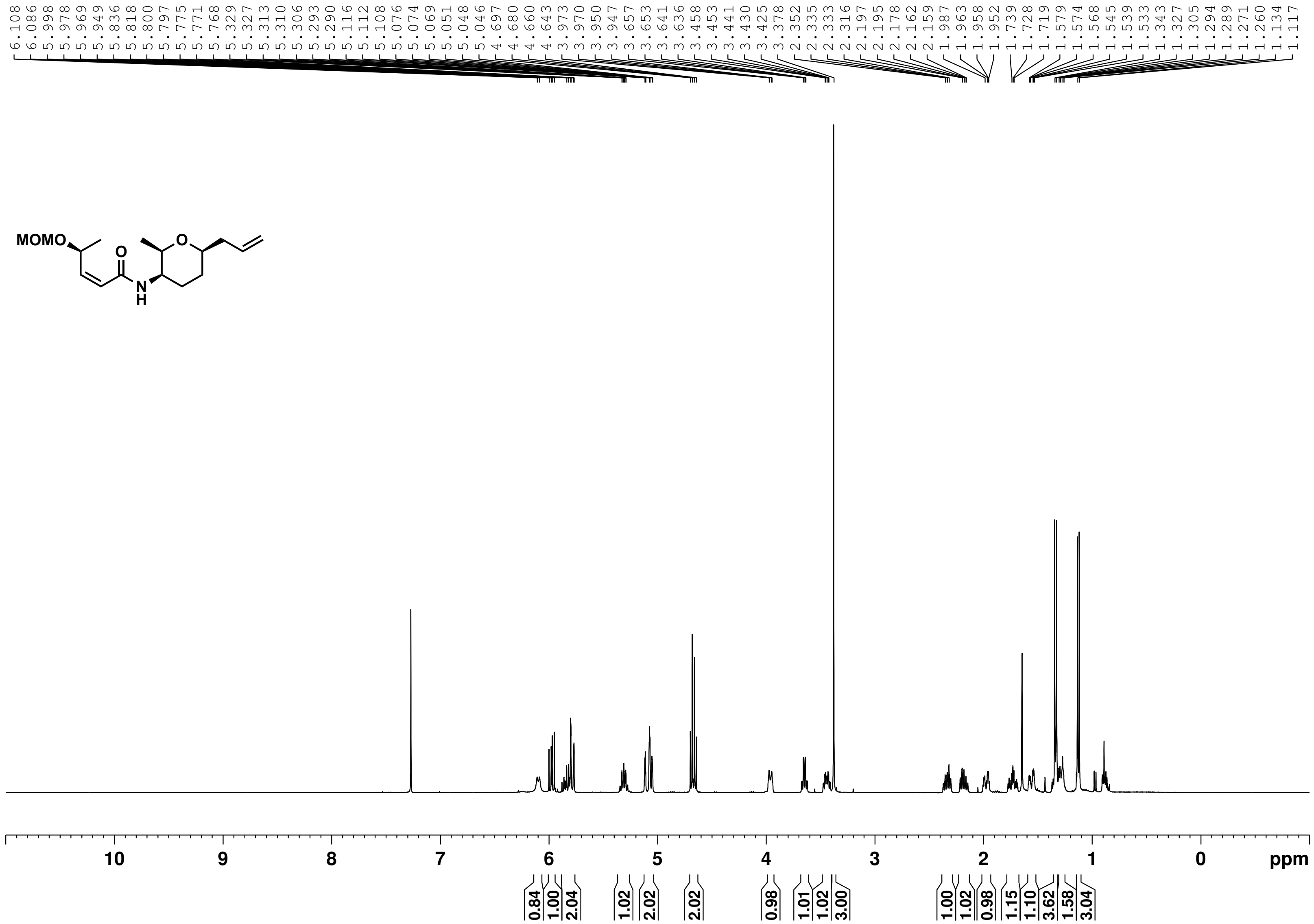

^{1}H NMR spectrum of (*S*,*Z*)-*N*-((2*R*,3*R*,6*R*)-6-allyl-2-methyltetrahydro-2*H*-pyran-3-yl)-4-(methoxymethoxy)pent-2-enamide **13** (400 MHz, $CDCl_3$, 293K)

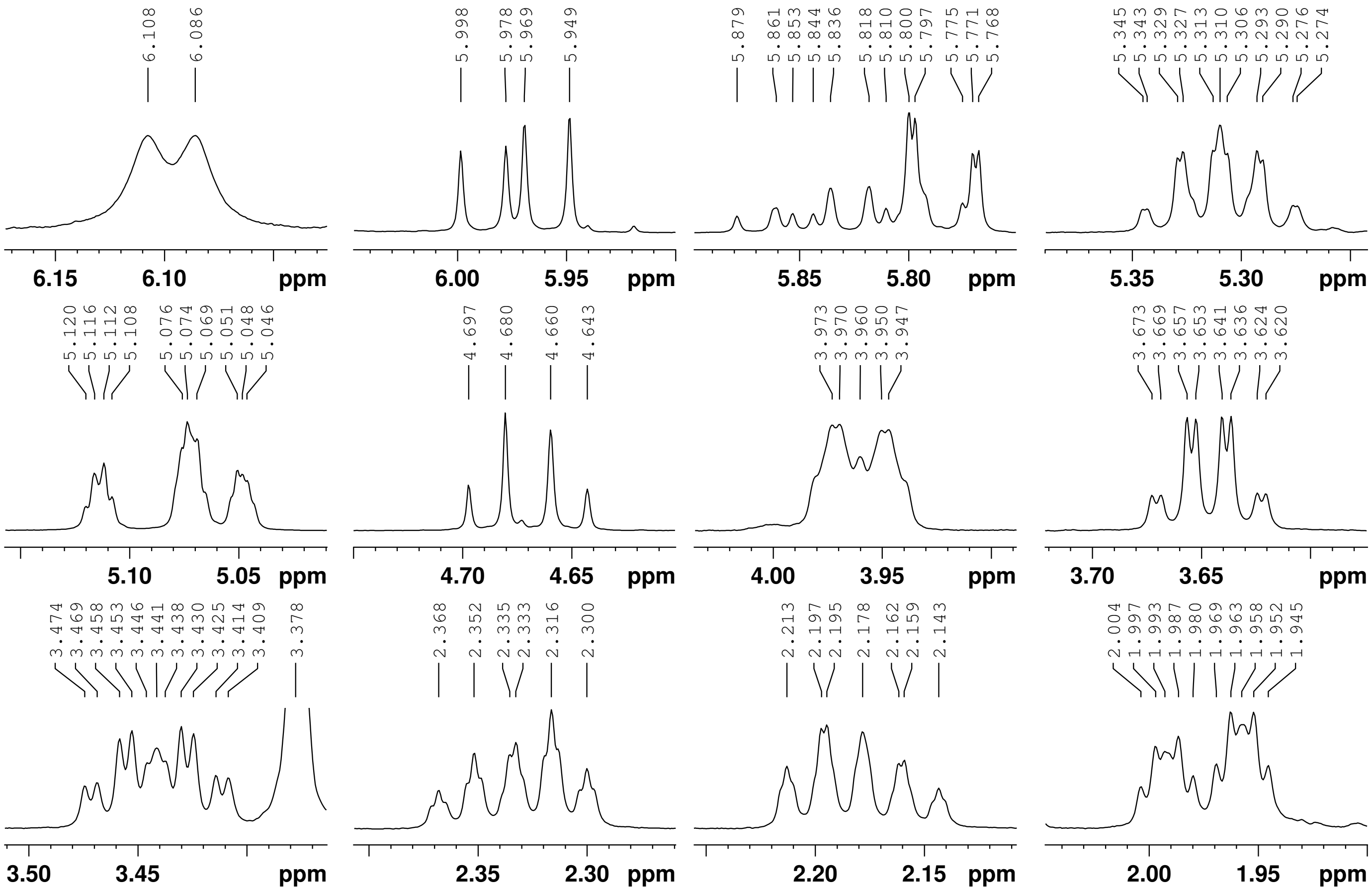

^{1}H NMR spectrum of (*S*,*Z*)-*N*-((2*R*,3*R*,6*R*)-6-allyl-2-methyltetrahydro-2*H*-pyran-3-yl)-4-(methoxymethoxy)pent-2-enamide **13** (400 MHz, $CDCl_3$, 293K)

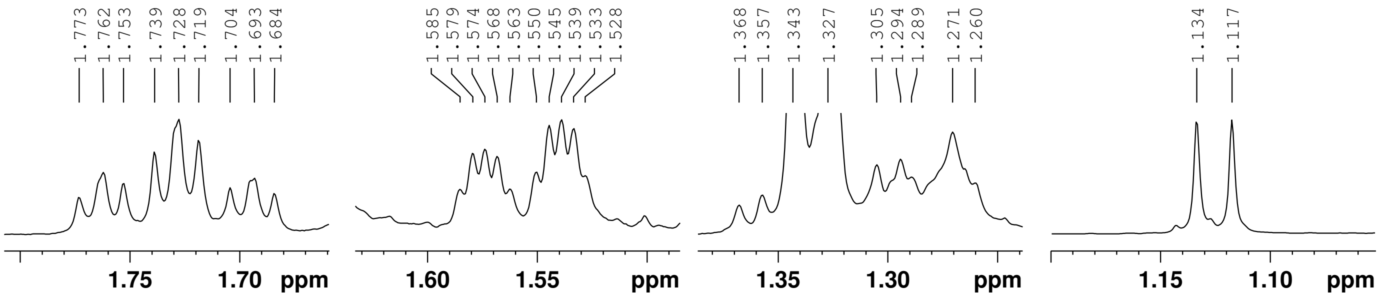

^{1}H NMR spectrum of (*S*,*Z*)-*N*-((2*R*,3*R*,6*R*)-6-allyl-2-methyltetrahydro-2*H*-pyran-3-yl)-4-(methoxymethoxy)pent-2-enamide **13** (400 MHz, $CDCl_3$, 293K)

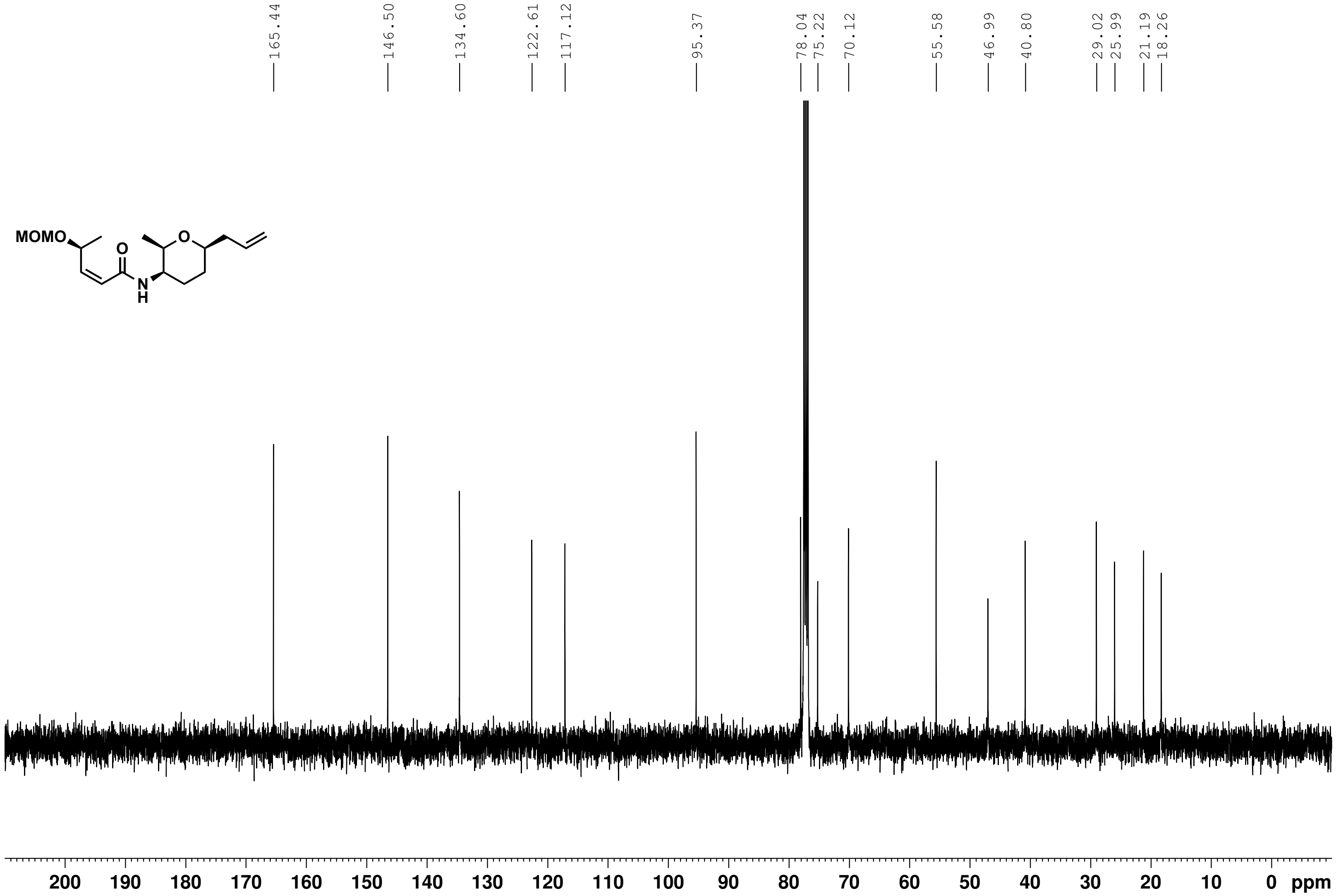

^{13}C NMR spectrum of (*S*,*Z*)-*N*-((2*R*,3*R*,6*R*)-6-allyl-2-methyltetrahydro-2*H*-pyran-3-yl)-4-(methoxymethoxy)pent-2-enamide **13** (100 MHz, $CDCl_3$, 293K)

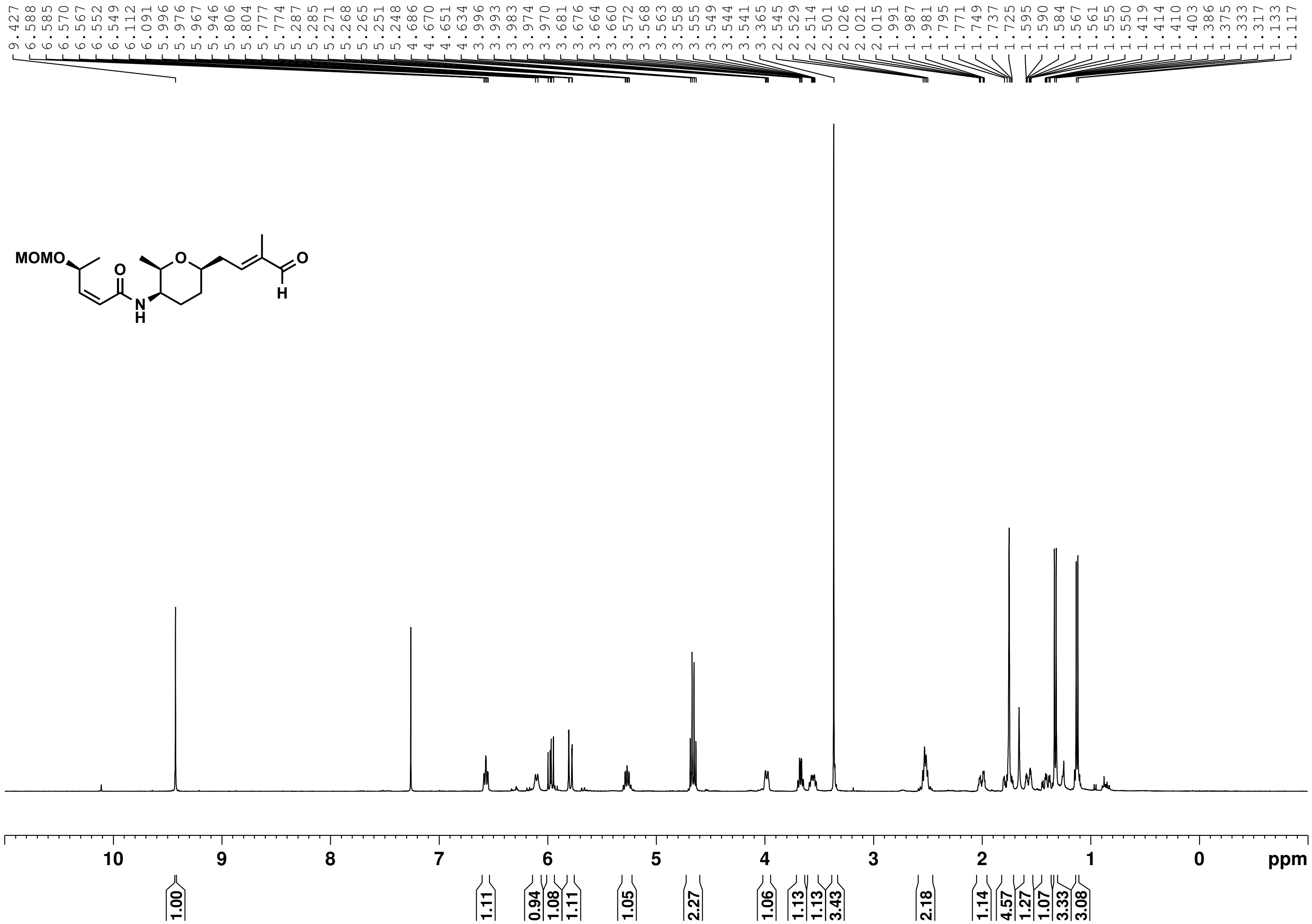

^{1}H NMR spectrum of (*S*,*Z*)-4-(methoxymethoxy)-*N*-((2*R*,3*R*,6*R*)-2-methyl-6-((*E*)-3-methyl-4-oxobut-2-en-1-yl)tetrahydro-2*H*-pyran-3-yl)pent-2-enamide **14** (400 MHz, $CDCl_3$, 293K)

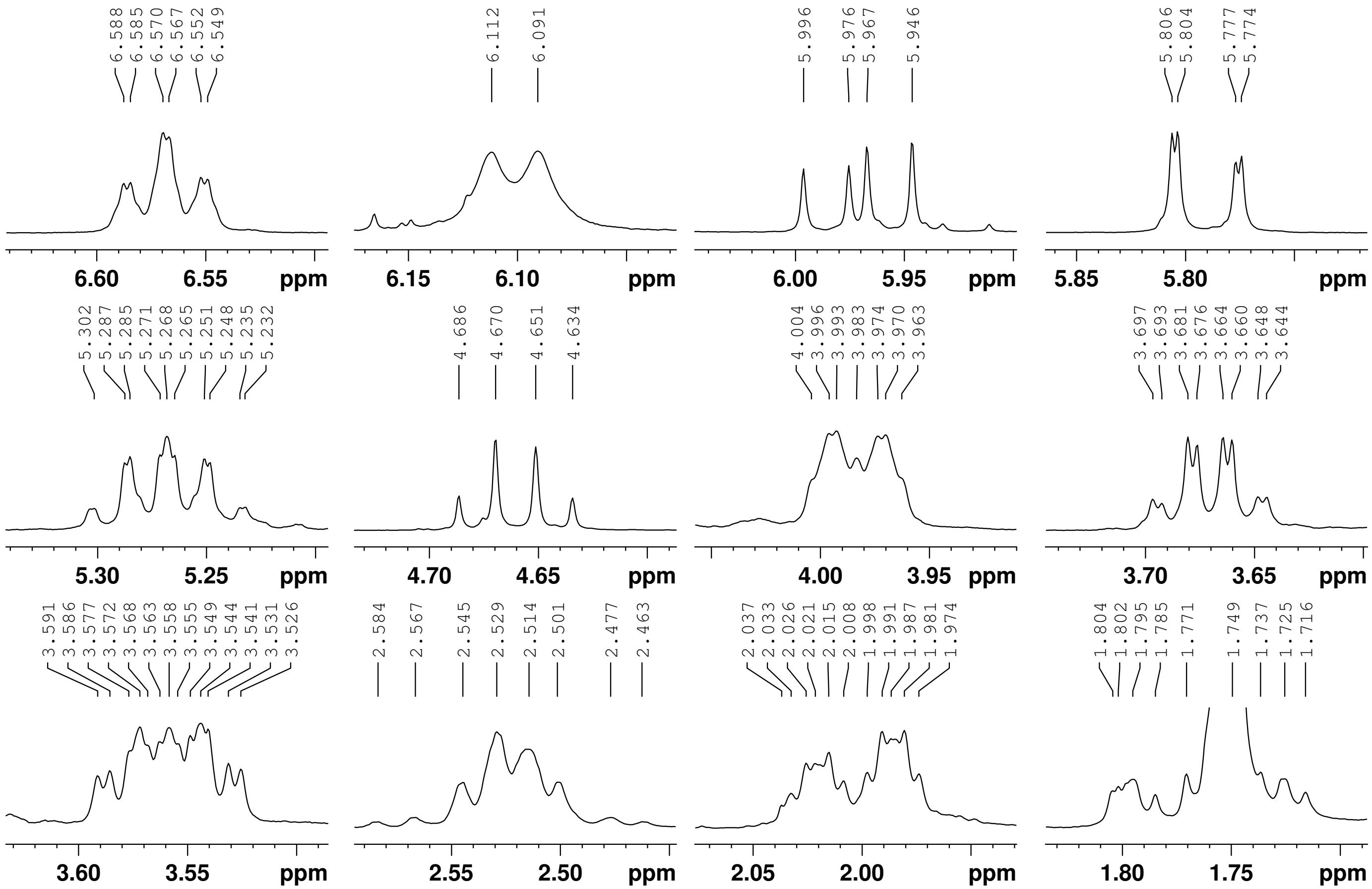

^{1}H NMR spectrum of (*S*,*Z*)-4-(methoxymethoxy)-*N*-((2*R*,3*R*,6*R*)-2-methyl-6-((*E*)-3-methyl-4-oxobut-2-en-1-yl)tetrahydro-2*H*-pyran-3-yl)pent-2-enamide **14** (400 MHz, $CDCl_3$, 293K)

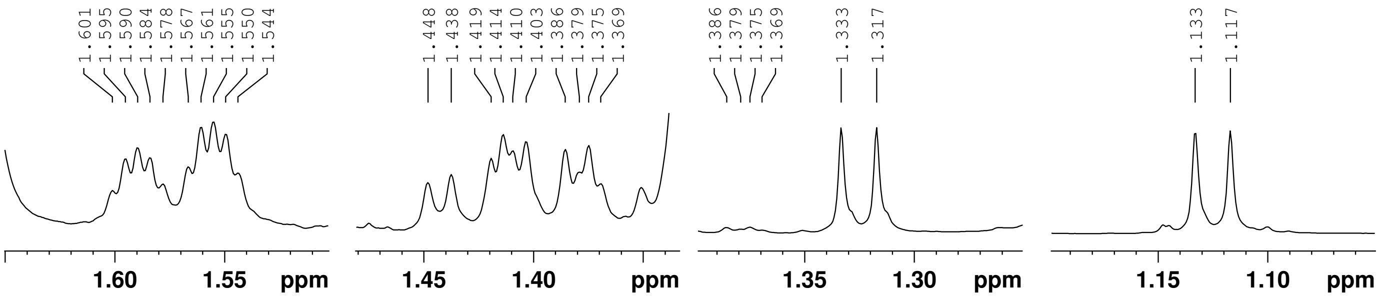

^{1}H NMR spectrum of (*S*,*Z*)-4-(methoxymethoxy)-*N*-((2*R*,3*R*,6*R*)-2-methyl-6-((*E*)-3-methyl-4-oxobut-2-en-1-yl)tetrahydro-2*H*-pyran-3-yl)pent-2-enamide **14** (400 MHz, $CDCl_3$, 293K)

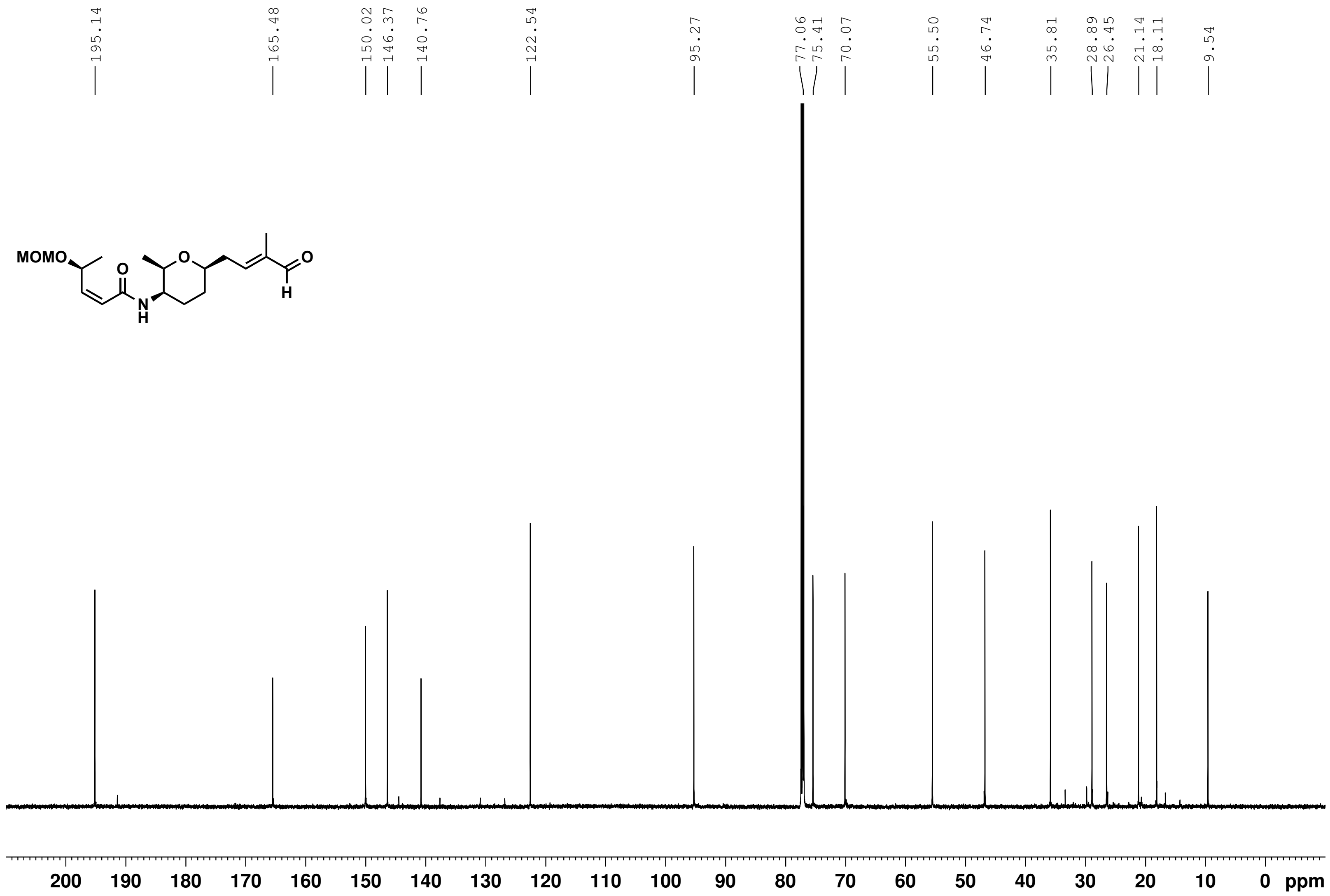

^{13}C NMR spectrum of (*S*,*Z*)-4-(methoxymethoxy)-*N*-((2*R*,3*R*,6*R*)-2-methyl-6-((*E*)-3-methyl-4-oxobut-2-en-1-yl)tetrahydro-2*H*-pyran-3-yl)pent-2-enamide **14** (150 MHz, $CDCl_3$, 293K)

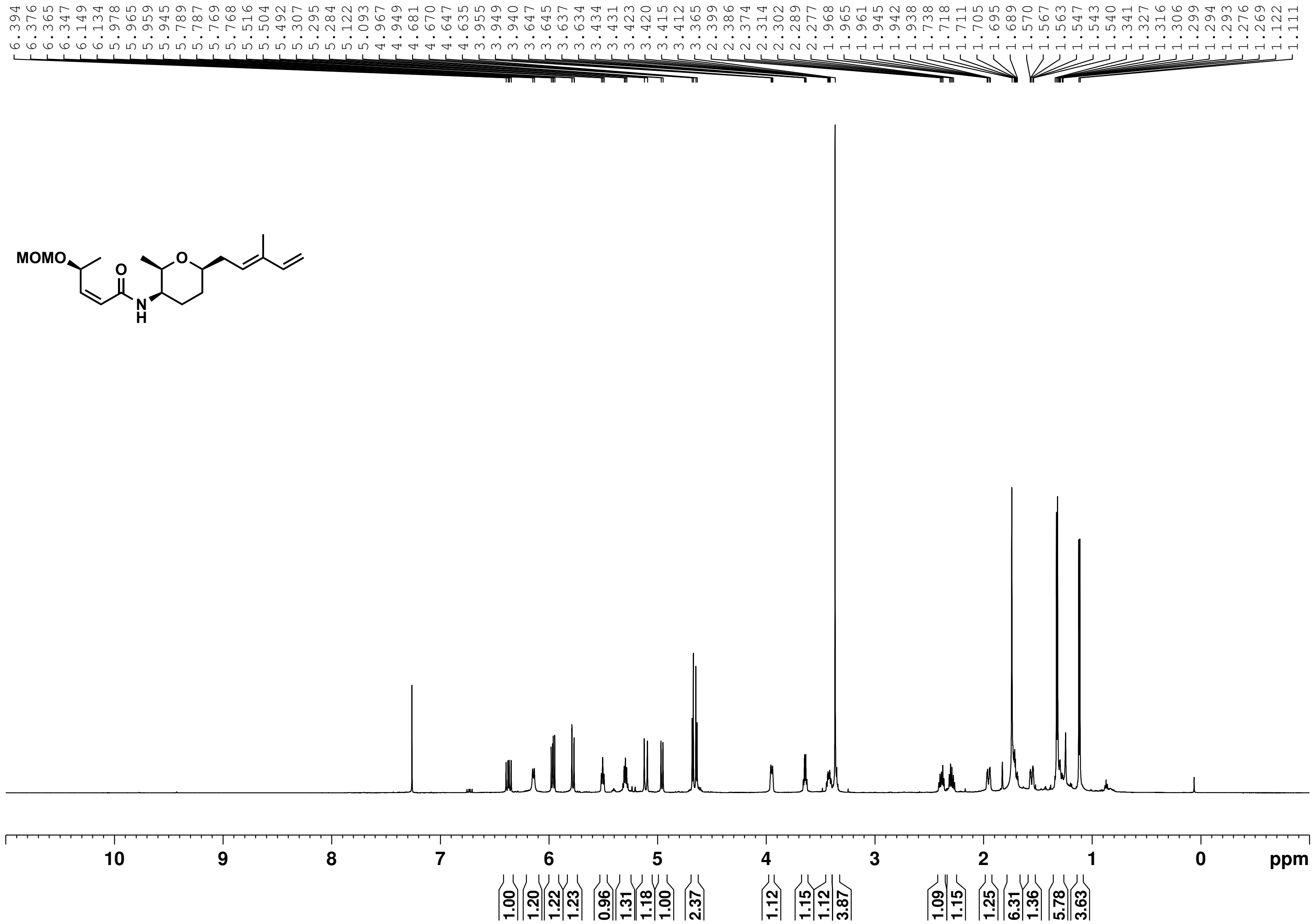

^{1}H NMR spectrum of (*S*,*Z*)-4-(methoxymethoxy)-*N*-((2*R*,3*R*,6*R*)-2-methyl-6-((*E*)-3-methylpenta-2,4-dien-1-yl)tetrahydro-2*H*-pyran-3-yl)pent-2-enamide **15** (600 MHz, $CDCl_3$, 293K)

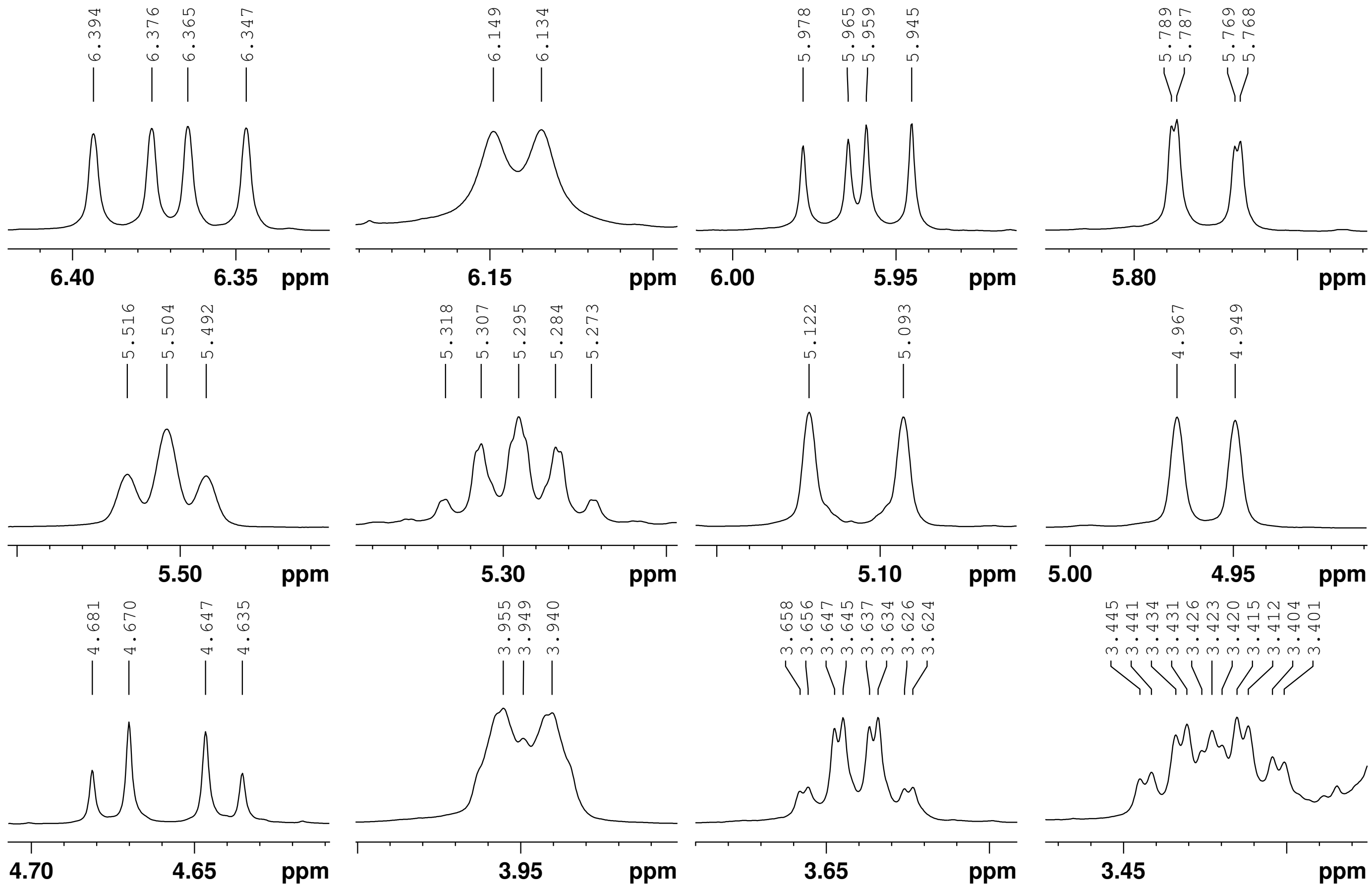

^{1}H NMR spectrum of (*S*,*Z*)-4-(methoxymethoxy)-*N*-((2*R*,3*R*,6*R*)-2-methyl-6-((*E*)-3-methylpenta-2,4-dien-1-yl)tetrahydro-2*H*-pyran-3-yl)pent-2-enamide **15** (600 MHz, $CDCl_3$, 293K)

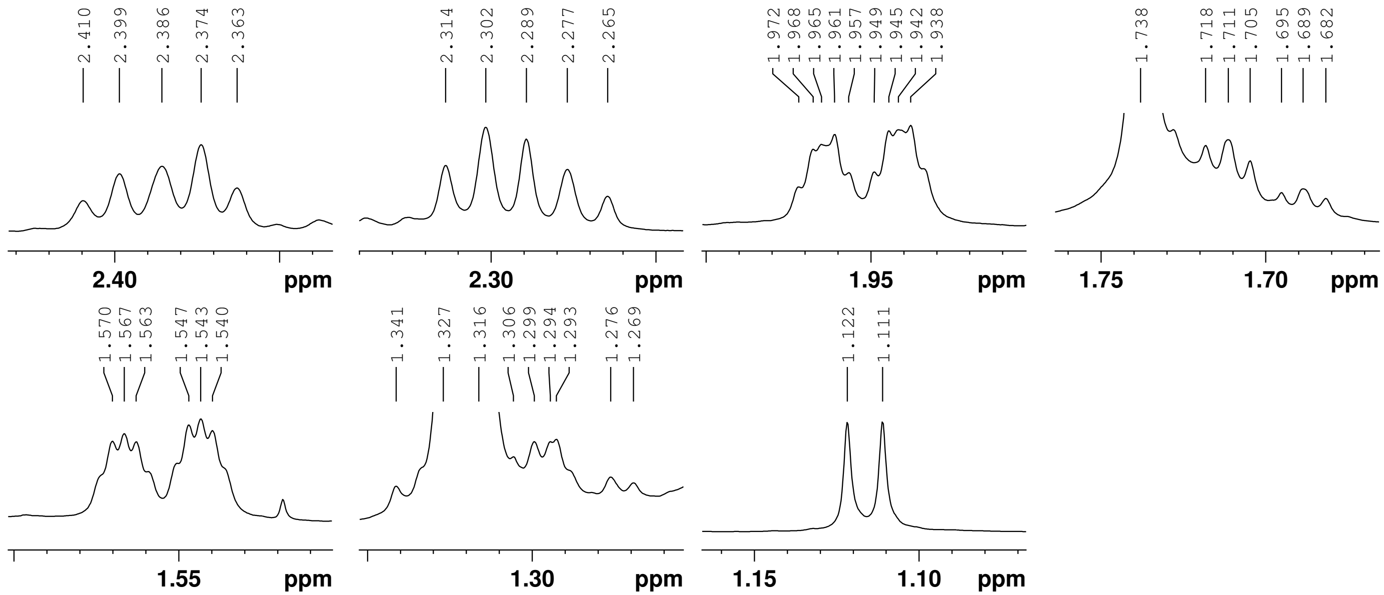

^{1}H NMR spectrum of (*S*,*Z*)-4-(methoxymethoxy)-*N*-((2*R*,3*R*,6*R*)-2-methyl-6-((*E*)-3-methylpenta-2,4-dien-1-yl)tetrahydro-2*H*-pyran-3-yl)pent-2-enamide **15** (600 MHz, $CDCl_3$, 293K)

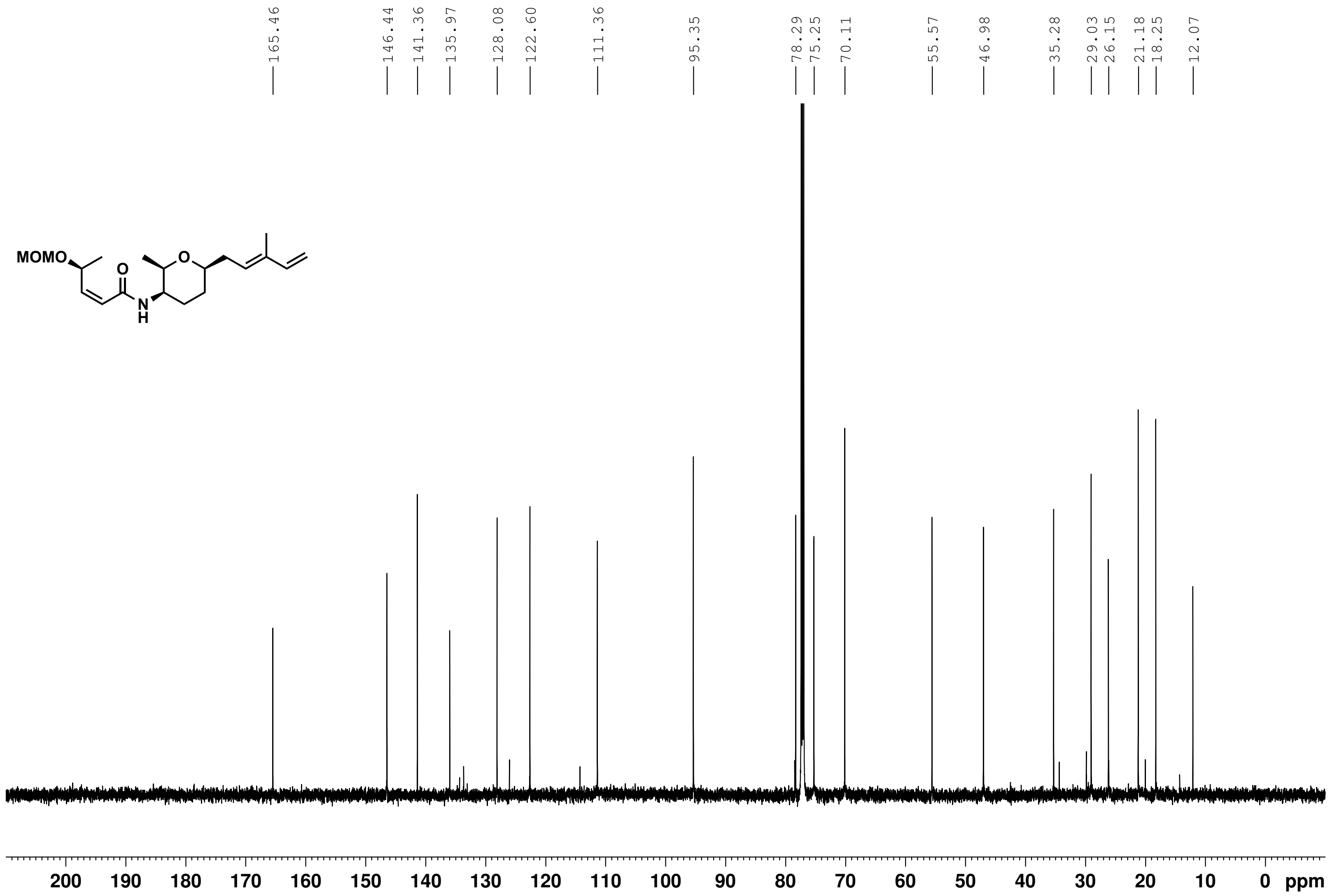

^{13}C NMR spectrum of (*S*,*Z*)-4-(methoxymethoxy)-*N*-((2*R*,3*R*,6*R*)-2-methyl-6-((*E*)-3-methylpenta-2,4-dien-1-yl)tetrahydro-2*H*-pyran-3-yl)pent-2-enamide **15** (150 MHz, $CDCl_3$, 293K)

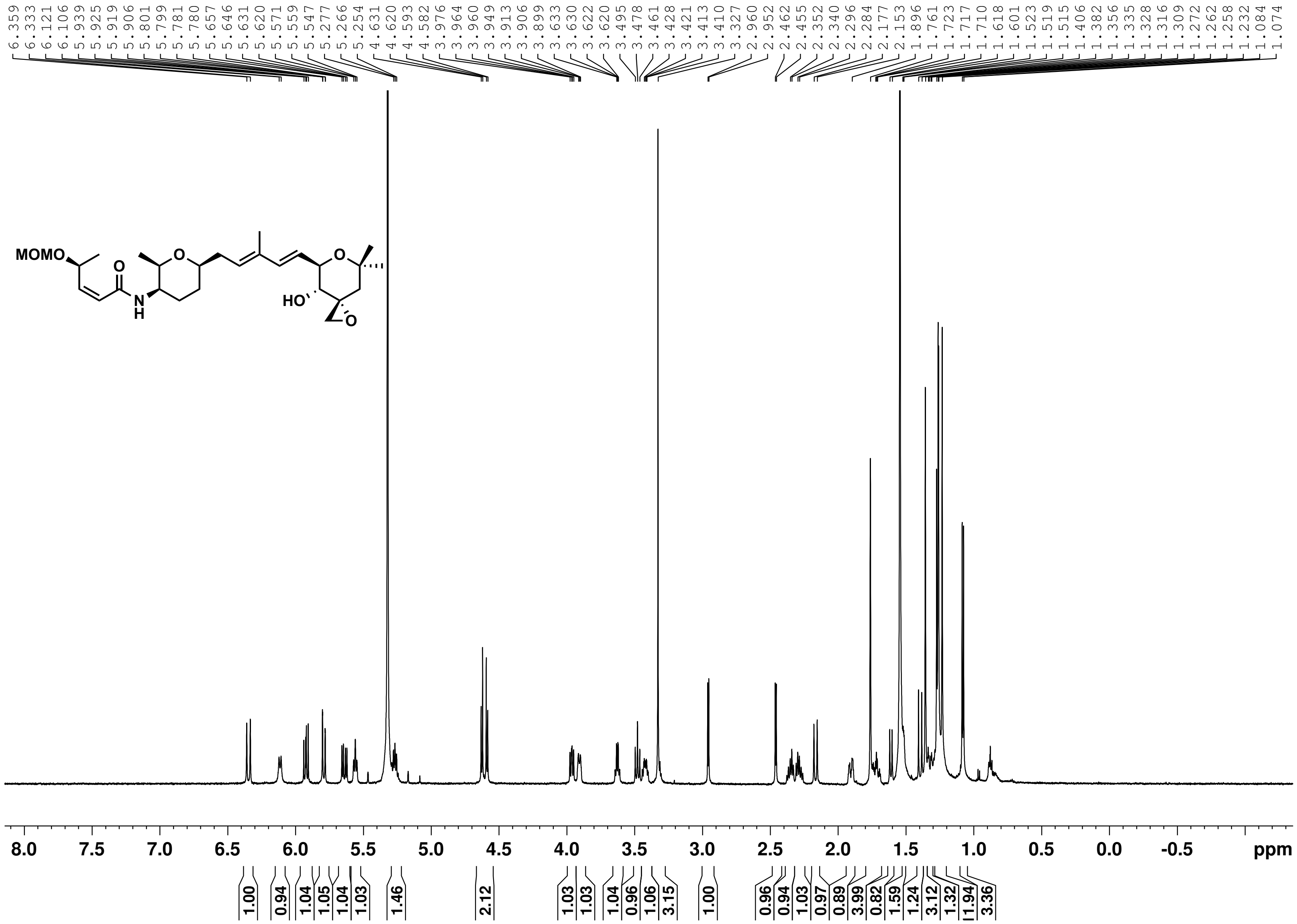

^{1}H NMR spectrum of meayamycin E (600 MHz, CD_2Cl_2, 293K)

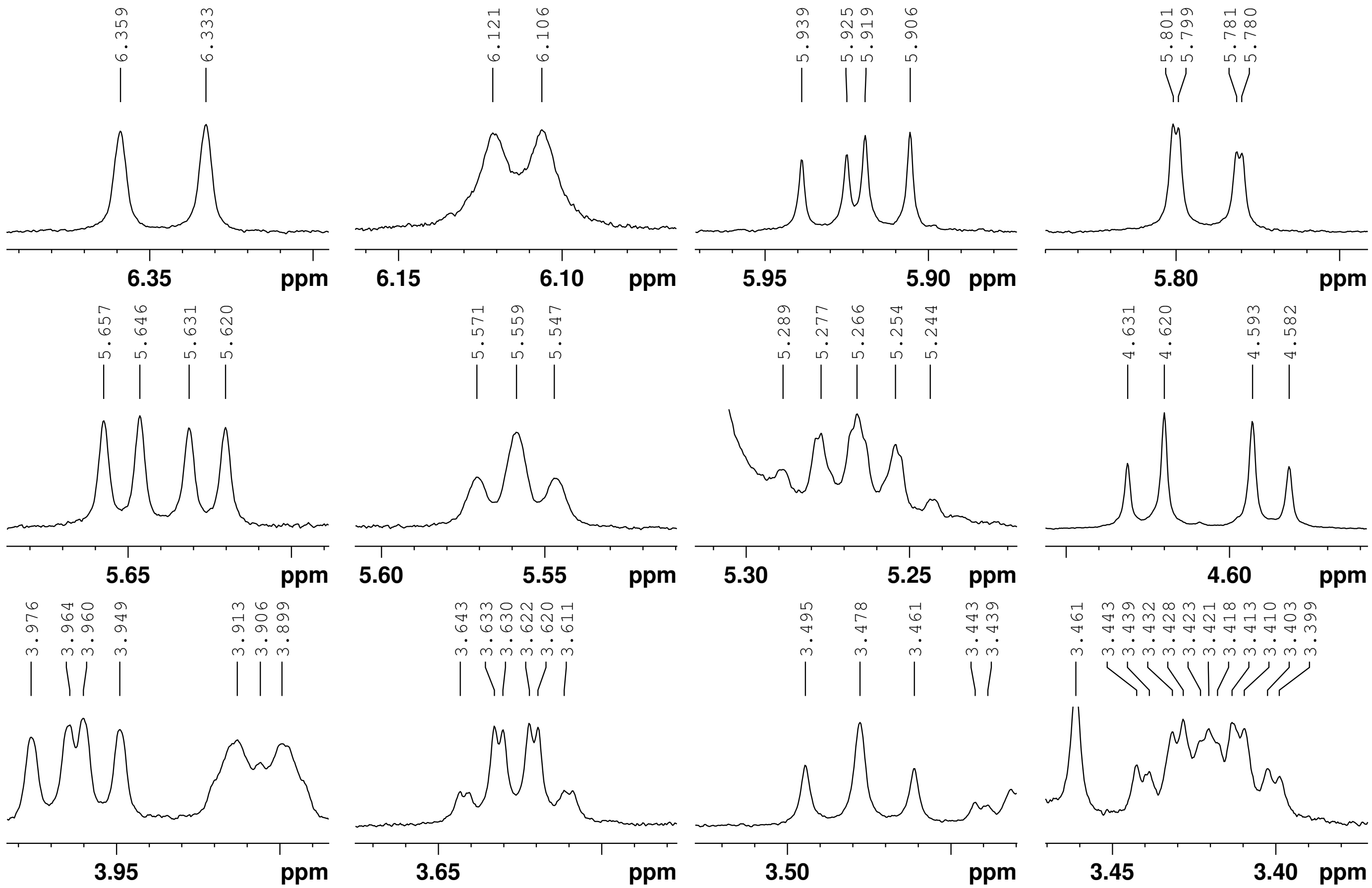

^{1}H NMR spectrum of meayamycin E (600 MHz, CD_2Cl_2, 293K)

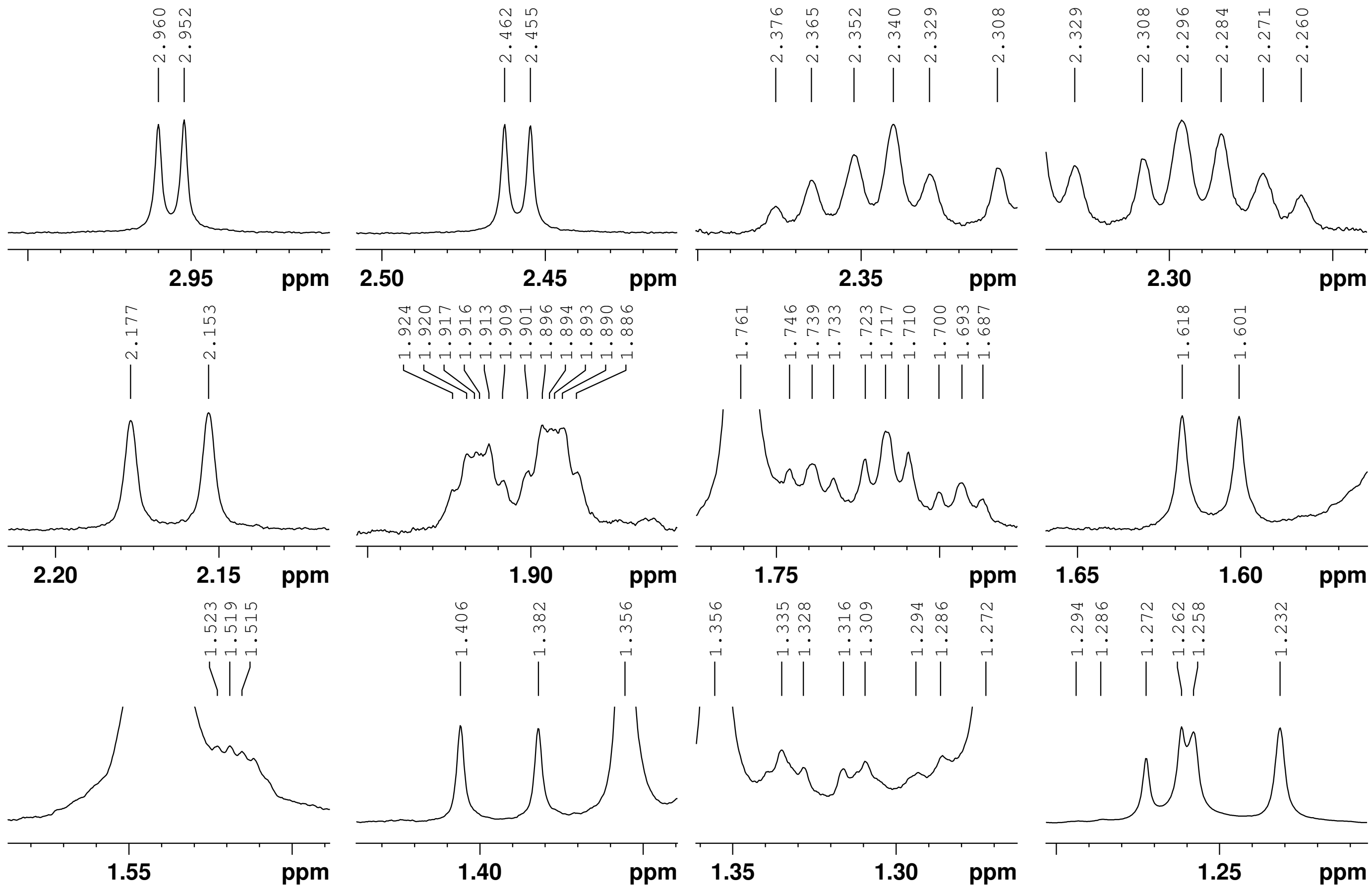

^{1}H NMR spectrum of meayamycin E (600 MHz, CD_2Cl_2, 293K)

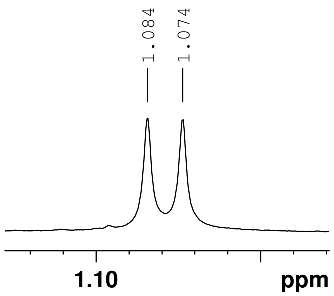

^{1}H NMR spectrum of meayamycin E (600 MHz, CD_2Cl_2, 293K)

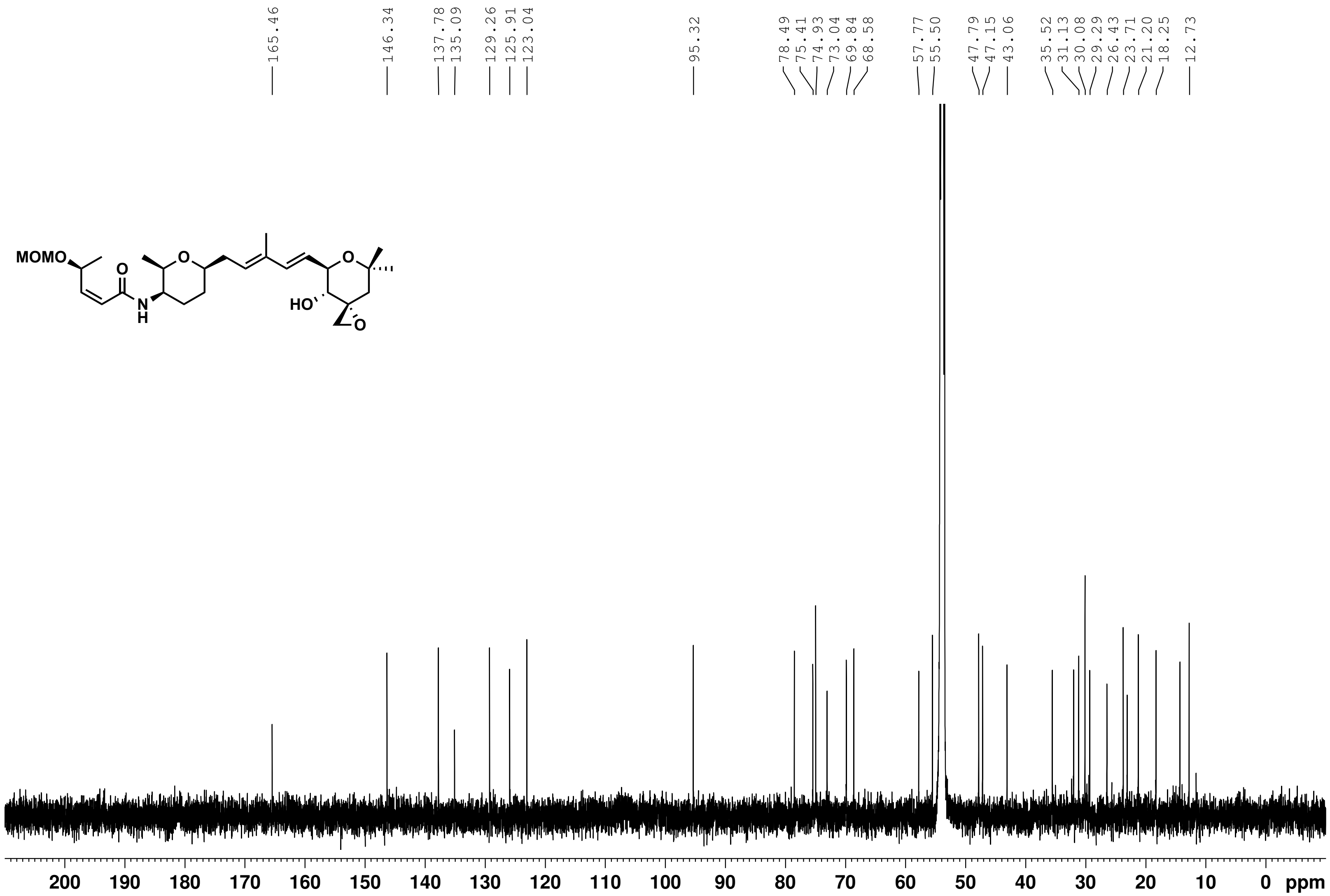

^{13}C NMR spectrum of meayamycin E (150 MHz, CD_2Cl_2, 293K)
